# Callipeltosides A, B and C: Total Syntheses and Structural Confirmation

**DOI:** 10.1002/chem.201501877

**Published:** 2015-07-31

**Authors:** James R Frost, Colin M Pearson, Thomas N Snaddon, Richard A Booth, Richard M Turner, Johan Gold, David M Shaw, Matthew J Gaunt, Steven V Ley

**Affiliations:** a Department of Chemistry, University of Cambridge Lensfield Road, Cambridge, CB2 1EW (UK); b Current address: Department of Chemistry, Indiana University 800 E. Kirkwood Ave., Bloomington, IN 47405 (USA)

**Keywords:** callipeltosides, cross-coupling, glycosidation, gold catalysis, organocatalysis

## Abstract

Since their isolation almost 20 years ago, the callipeltosides have been of long standing interest to the synthetic community owing to their unique structural features and inherent biological activity. Herein we present our full research effort that has led to the synthesis of these molecules. Key aspects of our final strategy include 1) synthesis of the C1–C9 pyran core (**5**) using an AuCl_3_-catalysed cyclisation; 2) formation of C10–C22 vinyl iodide (**55**) by sequential bidirectional Stille reactions and 3) diastereoselective union of these advanced fragments by means of an alkenylzinc addition (d.r.=91:9 at C9). The common callipeltoside aglycon (**4**) was completed in a further five steps. Following this, all three sugar fragments were appended to provide the entire callipeltoside family. In addition to this, D-configured callipeltose B was synthesised and appended to the callipeltoside aglycon. The ^1^H NMR spectrum of this molecule was found to be significantly different to the natural isolate, further supporting our assignment of callipeltoside B (**2**).

## Introduction

**Figure 1:**
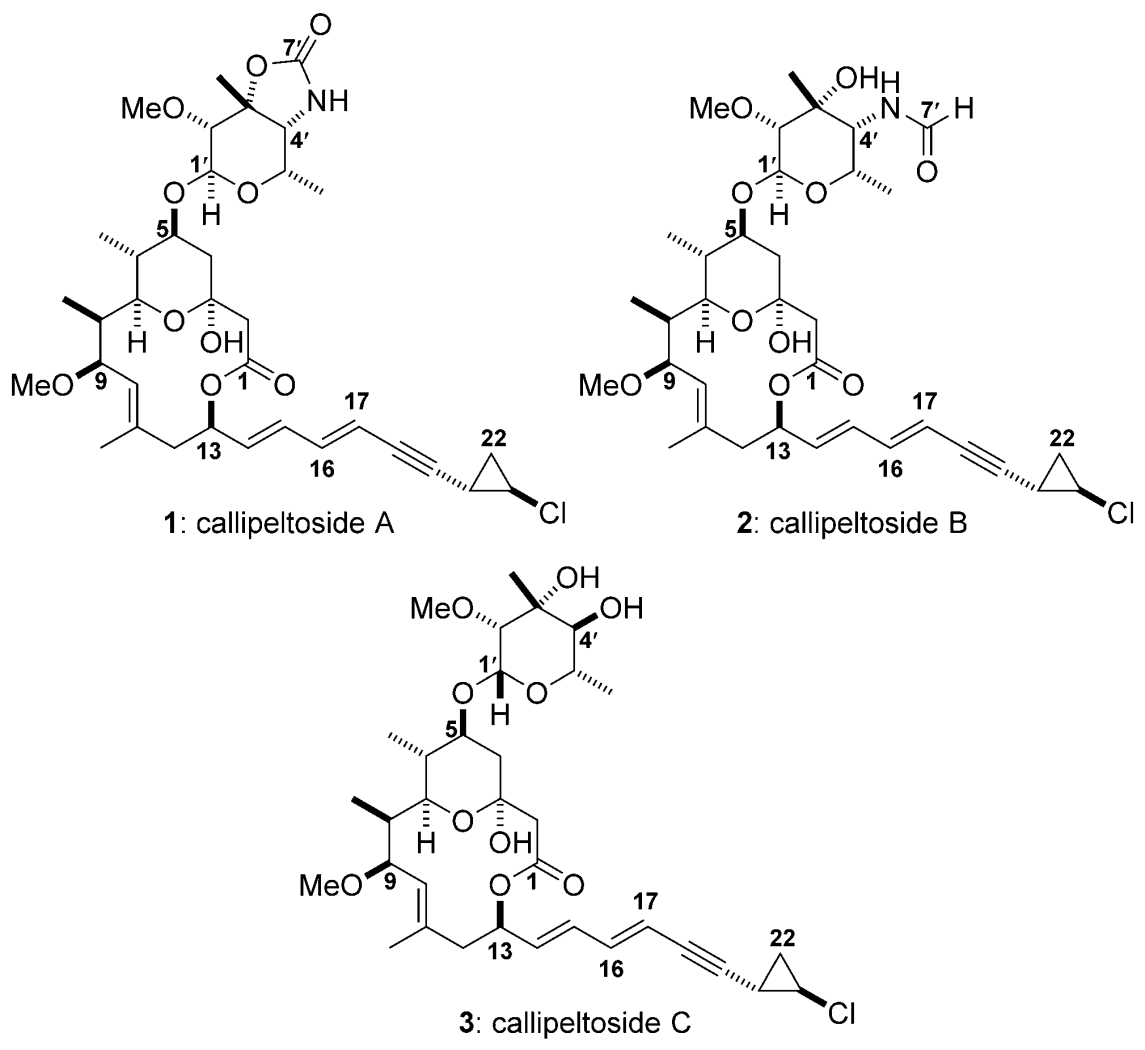
The callipeltoside family of natural products.

Natural product synthesis continues to provide an attractive platform for the discovery of new synthetic methods and further elaboration of novel synthesis pathways. In doing so, this effort importantly not only provides material for biological evaluation but also serves as a tool enabling unambiguous confirmation of structure. This can, in many examples, lead to structural refinement or even complete re-evaluation. A case in point concerns callipeltosides A, B and C (**1**–**3**) (Figure 1, shown in their finally corrected form). These fascinating compounds were first isolated by Minale in 1996 in vanishingly small quantities from the marine sponge *Callipelta sp*.^1^ Preliminary biological assays indicated a degree of cytotoxicity against human bronchopulmonary non-small-cell lung carcinoma (NSCLC-N6 and P388 cell lines).^1^ However, it was their unusual structural features: a 14-membered macrolide incorporating a tetrahydropyran hemiacetal together with a di-ene-yne attached to a *trans*-configured chlorocyclopropane ring that intrigued the synthesis community. The *trans*-configured chlorocyclopropane ring was of particular interest, since this feature is extremely scarce even now, with the phorbasides^2^ and the recently isolated muironolide A^3^ being the only other examples. Given the low availability of callipeltosides (0.8–3.5 mg) and their lack of suitable crystallinity for X-ray studies their complete structural assignment has been challenging.

While Minale correctly deduced the connectivity of the callipeltosides, several stereochemical features remained unclear and could not be resolved. Although the relative stereochemistry of the C1–C19 and C1′–C8’ fragments of these molecules could be deduced, the main concerns centred on the relative configuration of the *trans*-chlorocyclopropane ring with respect to the rest of the molecule, the stereochemical assignment of each glycoside moiety (D or L) and the stereochemistry of the glycosidic linkage. To solve these structural uncertainties and unambiguously deduce the absolute stereochemistry of the callipeltosides, total synthesis of these molecules was clearly necessary.

The synthesis of callipeltoside A was first achieved by the groups of Trost^4^ and Evans^5^ in 2002, following Paterson’s^6^ enantiomeric synthesis of the callipeltoside aglycon in 2001. Further syntheses of callipeltoside A were completed thereafter by the groups of Paterson,^7^ Panek,^8^ and Hoye^9^ as well as the preparation of several advanced fragments by others. In 2008, the synthesis of callipeltoside C was disclosed by MacMillan,^10^ and in so doing confirmed the absolute structure and provided evidence suggesting that the sugar moiety was L-configured as in callipeltoside A.

Prior to our recent report,^11^ the synthesis of callipeltoside B had not been achieved, whilst the glycosidic linkage of callipeltoside C had only been tentatively assigned on the basis of ^1^H NMR coupling constants.^10^ Whilst there has clearly been a considerable amount of effort dedicated to the syntheses of these molecules,^12^ we sought to develop an approach that would allow access to not only one, but all three of the callipeltosides in a highly convergent manner. Our synthetic strategy to complete various fragments of these molecules has evolved considerably over time, with certain methodologies, often developed in our own lab, superceded by more efficient and scalable alternatives. Herein we present our full research effort which has resulted in the realisation of our goal. We also provide further evidence to aid in the stereochemical assignment of the glycosidic linkages present in callipeltosides B and C.

### Synthetic plan

**Scheme 1:**
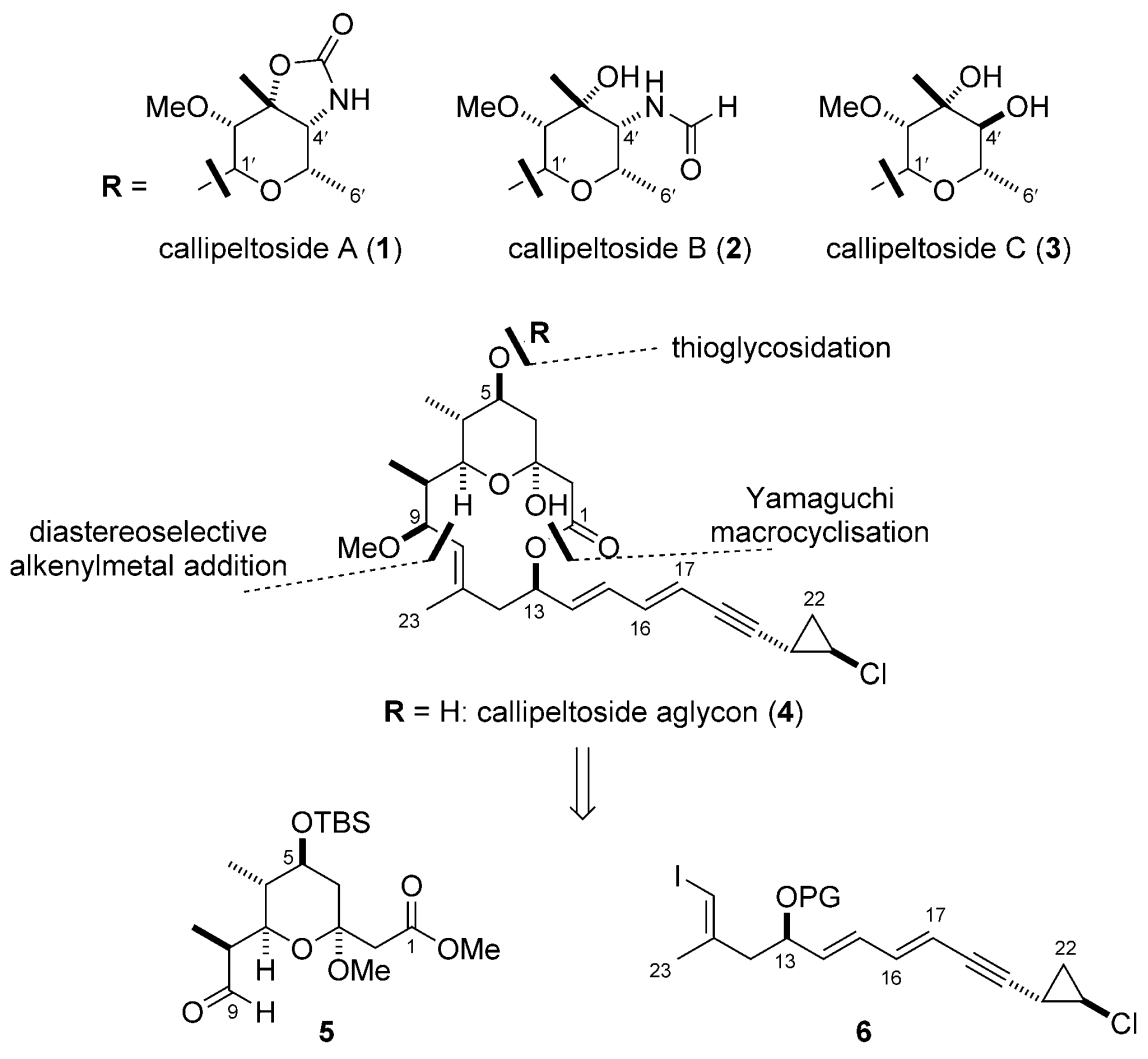
Retrosynthesis of callipeltosides A, B and C.

Identical to previous approaches to the callipeltosides, we chose to firstly disconnect the glycosidic linkage to reveal the common callipeltoside aglycon **4** (Scheme 1). With the knowledge that callipeltosides A and C both contained L-configured sugars it was considered likely that callipeltose B was also of the same configuration and, as such, we set ourselves the additional target of synthesising each callipeltoside sugar from a common, easily accessible precursor. In order to assemble the callipeltoside aglycon, we chose to use a Yamaguchi process^13^ to form the macrocyclic ring but also committed ourselves to a bold coupling strategy using similar-sized fragments **5** and **6**. At the start of the synthetic campaign, we initially considered the use of an asymmetric organocatalytic cyclopropanation method,^14^ developed in our laboratory, to prepare the *trans*-chlorocyclopropane ring. We anticipated that the C13 stereocentre could be set by use of a pyrrolidine tetrazole catalysed oxyamination,^15^ and a double dithiol conjugate addition strategy would provide access to the core pyran fragment (**5**).^16^ While these were later abandoned in favour of more efficient, alternative methodology, they nicely illustrate how a complex synthesis evolves.

## Results and Discussion

### Synthesis of pyran 5

**Scheme 2:**
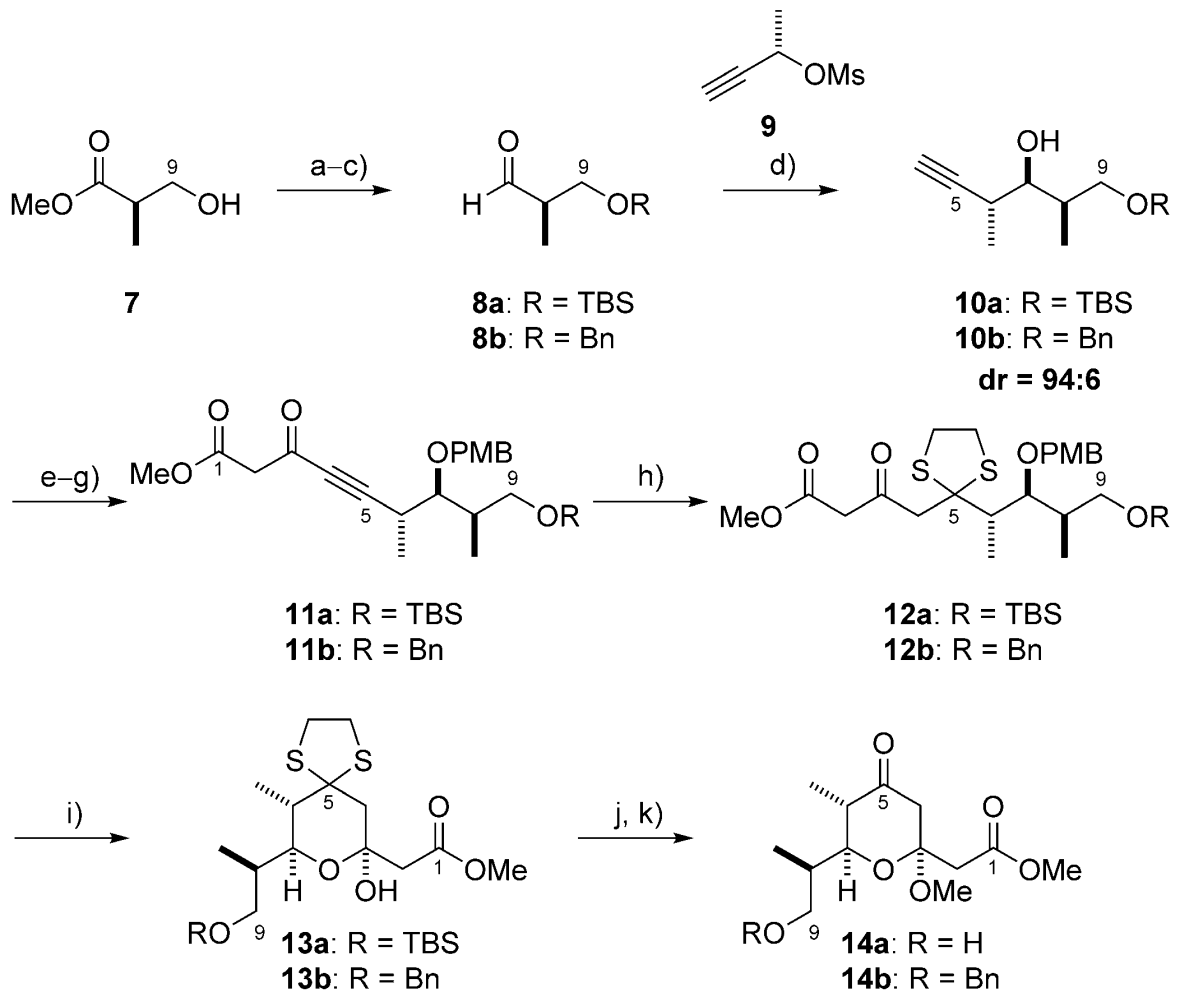
a) TBSCl, Et_3_N, DMAP, CH_2_Cl_2_, RT, 99 %; or benzyl trichloroacetimidate, TfOH, CH_2_Cl_2_, RT, 81 %; b) LiBH_4_, Et_2_O, MeOH, 0 °C, TBS/Bn=99 %; c) oxalyl chloride, DMSO, Et_3_N, CH_2_Cl_2_, −78 °C, 8 a/8 b=quant.; d) Pd(OAc)_2,_ PPh_3_, Et_2_Zn, THF, −78→−25 °C, 10 a=70 %, 10 b=71 %; e) NaH, PMBBr, DMF/THF (1:1), 0 °C, TBS=82 %, Bn=86 %; f) *n*BuLi, ethyl chloroformate, THF, −78 °C→RT, TBS=95 %, Bn=95 %; g) LDA, methyl acetate, THF, −78 °C→RT; h) 1,2-ethanedithiol, NaOMe, CH_2_Cl_2_/MeOH (1:1), −10 °C→RT; i) DDQ, pH 7 phosphate buffer, CH_2_Cl_2_, RT, 13 a=68 % over 3 steps, 13 b=60 % over 3 steps; j) PPTS, trimethylorthoformate, MeOH, RT, TBS=92 %, Bn=81 %; k) [bis(trifluoroacetoxy)iodo]benzene, MeCN, H_2_O, 0 °C, 14 a=86 %, 14 b=87 %. DDQ=2,3-dichloro-5,6-dicyano-1,4-benzoquinone; DMAP=4-dimethylaminopyridine; LDA=lithium diisopropylamide; PMB=4-methoxybenzyl ether. PPTS=pyridine *p*-toluenesulfonate.

**a) Double dithiol conjugate addition approach to C1–C9 pyran 5**: Our synthetic effort began with the preparation of the pyran aldehyde **5** from (*R*)-configured Roche ester **7**. This was initially protected as its TBS-ether and converted to the corresponding aldehyde in a further two steps. Reaction with mesylate **9** using the conditions developed by Marshall^17^ then enabled formation of the C6 and C7 stereocentres in good yield (70 %) and high diastereoselectivity (d.r.=94:6). The C7 alcohol was protected as the *p*-methoxybenzyl (PMB) ether and the scaffold further elaborated in two steps to give alkyne **11 a**. This provided the opportunity to perform a double dithiol conjugate addition which, as anticipated, smoothly furnished **12 a** containing a masked ketone at the C5-position.^16^ Deprotection of the PMB group using DDQ resulted in spontaneous cyclisation and simultaneously gave the desired functionalised pyran system as a single diastereoisomer. Subsequent ketal formation and removal of the dithiane provided deprotected ketone **14 a** in 29 % overall yield over 11 steps (Scheme 2).

**Scheme 3:**
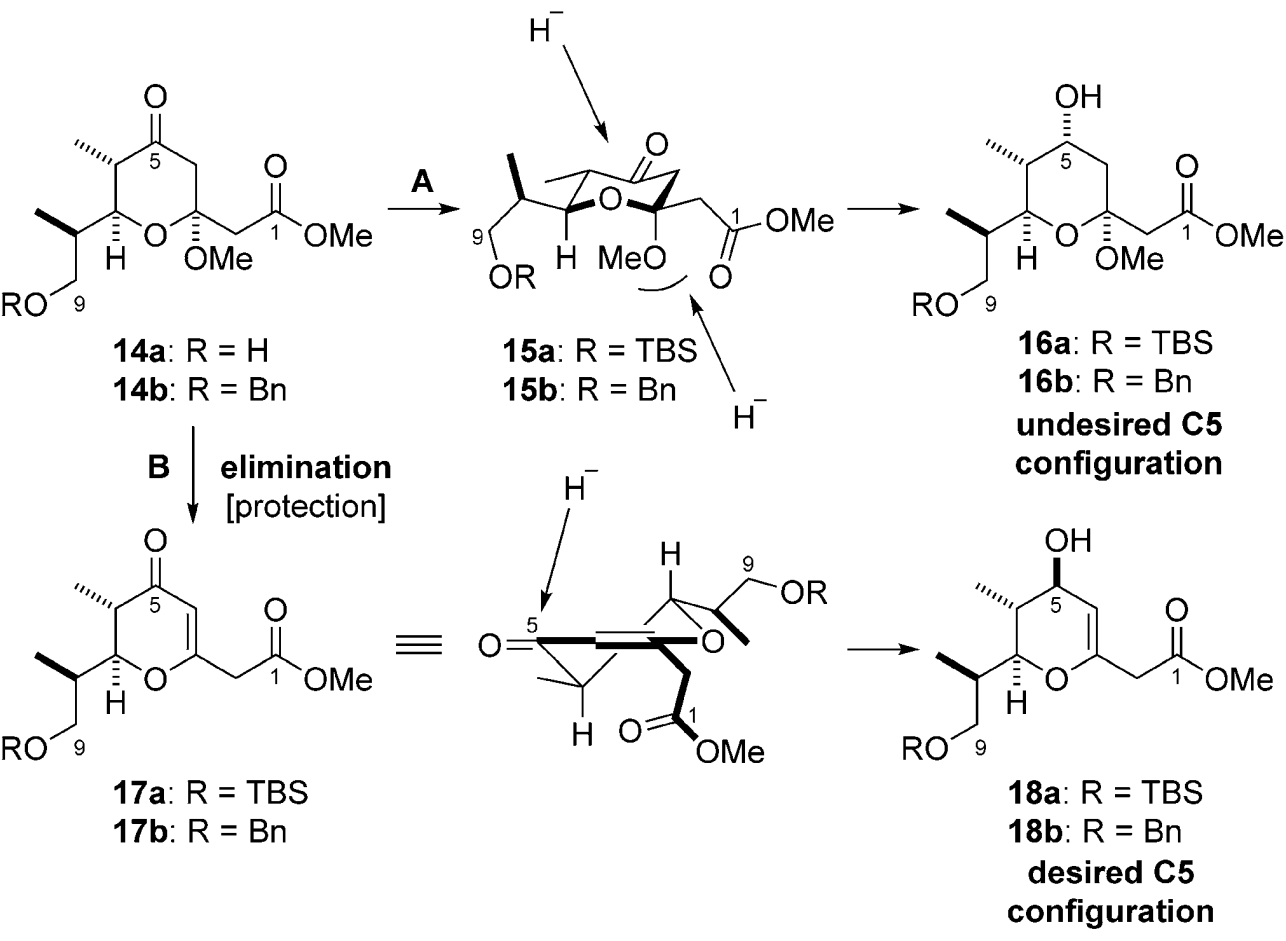
Installation of the C5 stereocentre.

**Scheme 4:**
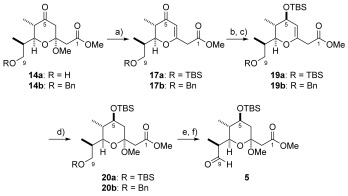
a) TfOH, CH_2_Cl_2_, RT, 17 b=88 %, (for 17 a (ii) TBSCl, imidazole, CH_2_Cl_2_, RT, 83 % over 2 steps); b) NaBH_4_, CeCl_3_⋅7 H_2_O, MeOH, −78 °C; c) TBSCl, imidazole, DMAP, CH_2_Cl_2_, RT, 19 a=90 % over 2 steps, 19 b=97 % over 2 steps; d) (±)-CSA, MeOH, RT, 20 a=20–68 %, 20 b=20–74 %; e) TBAF, THF, RT, TBS=67 % (+11 % recovered 20 a), for Bn: H_2_, Pd/C (10 wt. %), EtOAc, RT, 96 %; f) Dess–Martin periodinane, CH_2_Cl_2_, RT, 87 %. (±)-CSA=(±)-camphorsulfonic acid; TBAF=tetrabutylammonium fluoride.

At this stage a diastereoselective reduction of the ketone was required to set the C5 stereocentre. However, under a variety of common conditions it was not possible to obtain the desired configuration with good control. Further investigation suggested that the C3-ketal functionality was responsible, apparently influencing the trajectory of the incoming hydride source and hence the observed stereochemistry.^8^ This being the case, we chose to remove the troublesome C3-ketal through elimination to the pyranone **17 a**.^18^ Following this, the desired C5 stereochemistry could be set by Luche reduction^19^ to give **18 a** as a single diastereoisomer (Schemes 3 and 4).

This slight detour in our sequence therefore required that the C3-ketal had to be re-installed in order to complete the preparation of the key pyran aldehyde **5**. This seemingly trivial transformation was, however, found to be extremely capricious, with parallel batches under apparently identical reaction conditions providing variable yields ranging between 20 and 68 %. Unfortunately the variable nature of this reaction prohibited the synthesis of sufficient quantities of material. Our frustration was then further compounded by the fact that we were also unable to cleanly differentiate between the primary and secondary TBS protecting groups in the next step of the sequence. Optimal conditions required the use of TBAF as the limiting reagent (0.9 equiv), with separation and several recycles to process the material. For this reason, we chose to replace the primary TBS-ether with the benzyl protecting group at an early stage (see Schemes 2 and 3 for yields). This then removed the selectivity issue during the final deprotection step and provided pyran aldehyde **5** in a marginally improved step-count (17 steps) and yield (10.4 % overall yield) relative to the analogous TBS-protected sequence (18 steps, 8.6 % overall yield). However, the issues associated with the elimination and reinstallation of the ketal functionality remained, and therefore an alternative preparation of this fragment was sought.

**b) Gold-catalysed approach to C1–C9 pyran 5**: In 2009, we established a method whereby five and six-membered cyclic acetals could be synthesised by a AuCl_3_-catalysed hydroalkoxylation of appropriate conjugated alkynoates.^20^ This approach resulted in the formation of pyran motifs that were very similar to the desired C1–C9 pyran aldehyde **5**. It was therefore anticipated that this new approach would replace the dithiol conjugate addition methodology^16^ and could be adapted for the scale-up of the C1–C9 pyran aldehyde **5**. In doing so, the important contiguous C5–C8 stereocentres would also be implemented prior to cyclisation so as to overcome the problematic installation of the C5 stereocentre.

**Scheme 5:**
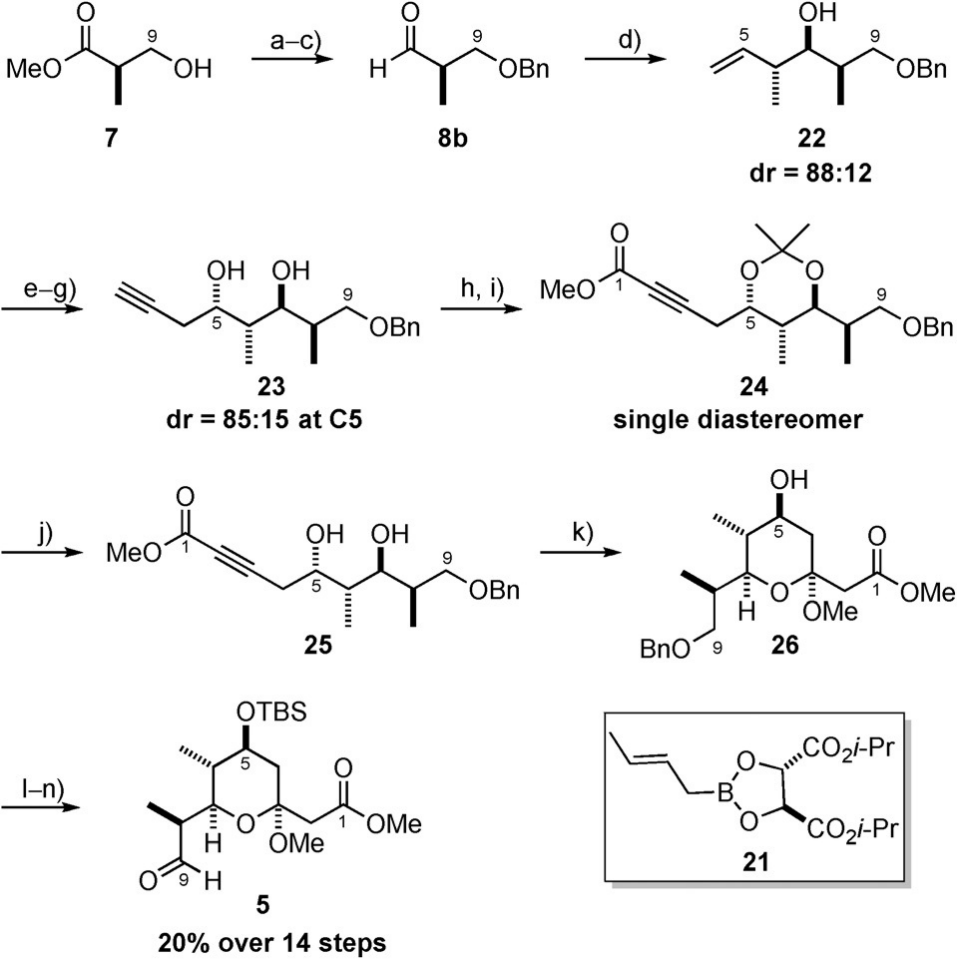
a) Benzyl trichloroacetimidate, TfOH, CH_2_Cl_2_, RT, 81 %; b) LiBH_4_, Et_2_O, MeOH, 0 °C, 99 %; c) oxalyl chloride, DMSO, Et_3_N, CH_2_Cl_2_, −78 °C, quant.; d) crotylborane 21, 4 Å MS, PhMe, −78 °C, 70 %; e) OsO_4_, NMO, acetone/H_2_O (2:1), RT; f) NaIO_4_, THF/H_2_O (10:1), 0 °C→RT; g) Zn, propargyl bromide, THF, 0 °C→−100 °C, 72 % over 3 steps; h) 2,2-dimethoxypropane, (±)-CSA, acetone, RT; i) *n*BuLi, THF, −40→−78 °C, then ClCO_2_Me, 73 % over 2 steps; j) QP-SA, MeOH, RT, 95 %; k) AuCl_3_ (2 mol %), MeOH, RT, 96 %; l) 2,6-lutidine, TBSOTf, CH_2_Cl_2_, −78 °C, 91 %; m) H_2_, Pd/C (10 % wt), EtOAc, RT, 96 %; n) Dess–Martin periodinane, K_2_CO_3_, CH_2_Cl_2_, RT, 87 %. NMO= *N*-methylmorpholine-*N*-oxide.

As before, aldehyde **8 b** was produced using a high-yielding three-step sequence of protection, reduction and oxidation (Scheme 5). With this in hand, we decided to set the C6 and C7 stereocentres by means of a diastereoselective crotylation process. While there have been several methods developed to achieve related transformations, both the Brown^21^ and Roush^22^ procedures have been frequently used in the synthesis of complex natural products. Both of these methods resulted in good levels of diastereoselectivity (Brown=86:14 c.f. Roush=88:12), although the Roush crotylation was found to be higher yielding (70 vs. 37 %).^23^ The remaining crucial C5 stereocentre was then set using a three-step sequence involving dihydroxylation, diol cleavage and propargylzinc addition, which resulted in good diastereoselectivity (85:15) and yield (72 % yield over 3 steps). Protection as the acetonide, followed by reaction with methyl chloroformate then provided ynoate **24**. At this stage all minor diastereoisomers could be removed by column chromatography and importantly we could confirm the relative stereochemistry of the four contiguous C5–C8 stereocentres by X-ray crystallography (see the Supporting Information). Finally removal of the acetonide afforded the diol **25**, ready for the key AuCl_3_-catalysed cyclisation. Treatment of diol **25** with only 2 mol % AuCl_3_ in MeOH at room temperature resulted in clean formation of pyran **26** in an impressive 96 % yield, with product purification requiring simple filtration through celite® to remove metal impurities.^24^ Our previous route could then be intercepted by TBS-protection of the C5 alcohol (Scheme 5), with hydrogenolysis of the benzyl group and oxidation of the resulting primary alcohol completing the synthesis of pyran aldehyde **5**.

This third-generation sequence provided a much-improved overall yield of 20 % in just 14 steps (from (*R*)-Roche ester **7**), and also importantly required only eight chromatographic purifications allowing for the synthesis of multigrams of material. With a high-yielding and scalable route to pyran aldehyde **5** in place, we turned our attention to the preparation of the di-ene-yne containing vinyl iodide fragment **6**.

### Formation of the C16–C17 bond: Horner–Wadsworth–Emmons and Julia–Kocienski approaches to vinyl iodide 6

**Scheme 6:**
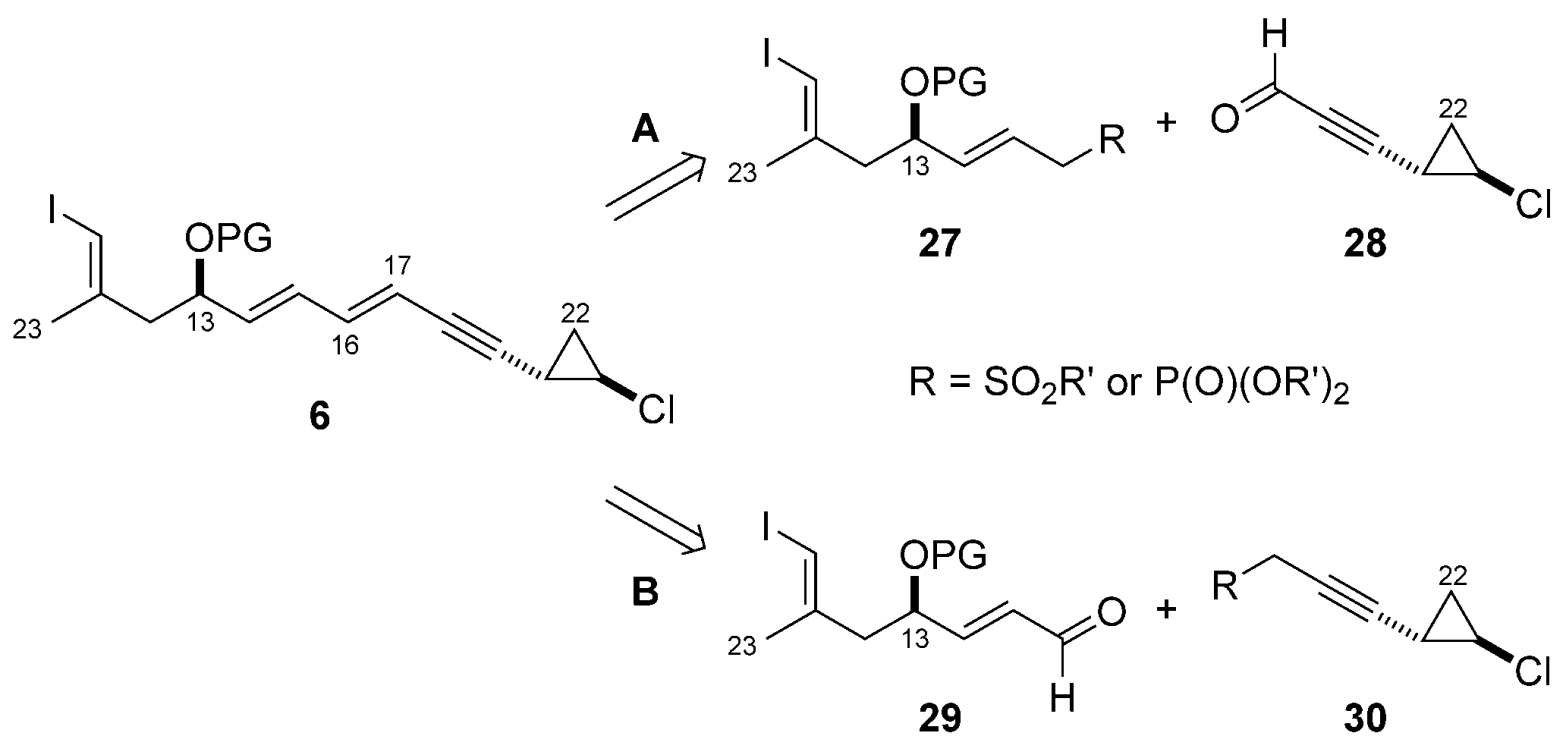
Horner–Wardsworth–Emmons and Julia–Kocienski approaches to vinyl iodide 6.

Our principal retrosynthetic disconnection of the callipeltosides required union of the fully elaborated vinyl iodide fragment **6** (shown with a generic protecting group) with pyran aldehyde **5** by a diastereoselective alkenylmetal addition to form the C9–C10 bond, allowing for maximum convergence. A clear priority in the synthesis of vinyl iodide **6** was the construction of the embedded di-ene-yne system as a single *E*,*E*-isomer. In order to achieve this, we initially chose the C16–C17 bond as the key disconnection, for which there was little literature precedence.^25^ This analysis revealed two potential pathways (Scheme 6, disconnection A and B) from which the fragment could be obtained, namely by a Horner–Wadsworth–Emmons (HWE)^26^ or Julia–Kocienski^27^ coupling partners **27** and **30**, along with their respective aldehydes **28** and **29**. As mentioned earlier, it was anticipated that the organocatalytic methods developed in our laboratory would be key to the formation of the *trans*-chlorocyclopropane ring^14^ and also the C10–C15 sub-fragments.^15^

**Scheme 7:**
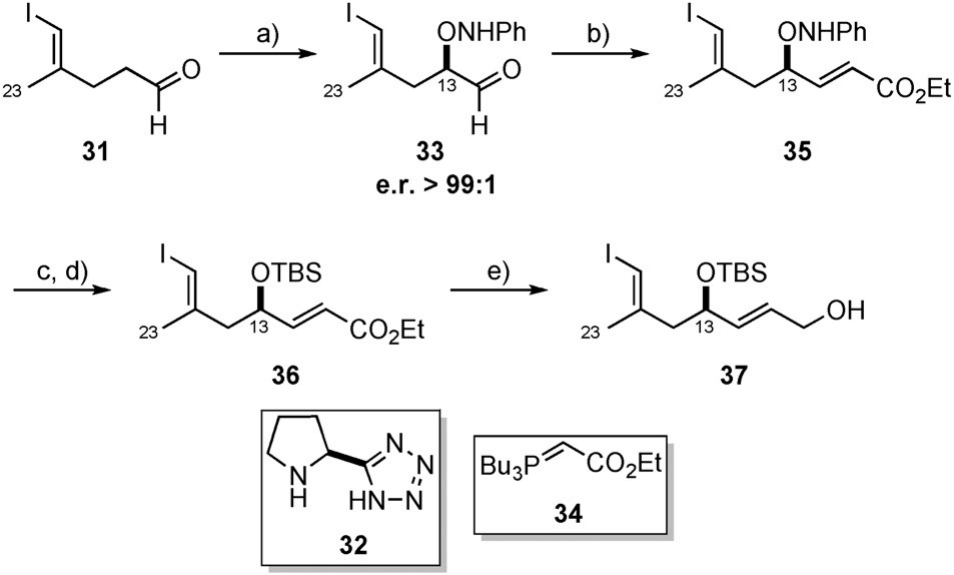
a) 32 (10 mol %), nitrosobenzene, DMSO, RT; b) 34, THF, RT, 66 % over 2 steps; c) CuSO_4_, *i*PrOH/THF (10:1), 40 °C, 52 %; d) TBSOTf, 2,6-lutidine, CH_2_Cl_2_, −78 °C, 93 %; e) DIBAL-H, THF, −78 °C, quant. DIBAL-H=diisobutylaluminium hydride.

**a)** **Synthesis of C10–C15 vinyl iodide 37**: For the C10–C15 unit, we chose to make use of known aldehyde **31**, prepared in two steps from 4-pentyl-1-ol (see the Supporting Information). In a similar fashion to the synthesis of callipeltoside C disclosed by MacMillan,^10^ we chose to apply an enantioselective organocatalytic oxyamination reaction^28^ to set the C13 stereocentre. This was achieved by treatment of aldehyde **31** with nitrobenzene and proline-derived tetrazole catalyst **32** to deliver **33** in excellent enantioselectivity (e.r. determined to be >99:1 following NaBH_4_ reduction). Practically, we found that aldehyde **33** was not easily isolated in pure form, and therefore it was conveniently reacted directly with phosphorane **34** in a one-pot procedure to install the C14/C15 *E*-configured double bond and provide **35** as a single isomer in 66 % yield over two steps (Scheme 7). Cleavage of the N–O bond, TBS protection and reduction provided allylic alcohol **37**, from which both the HWE or Julia–Kocienski coupling partners could be realised.

**Scheme 8:**
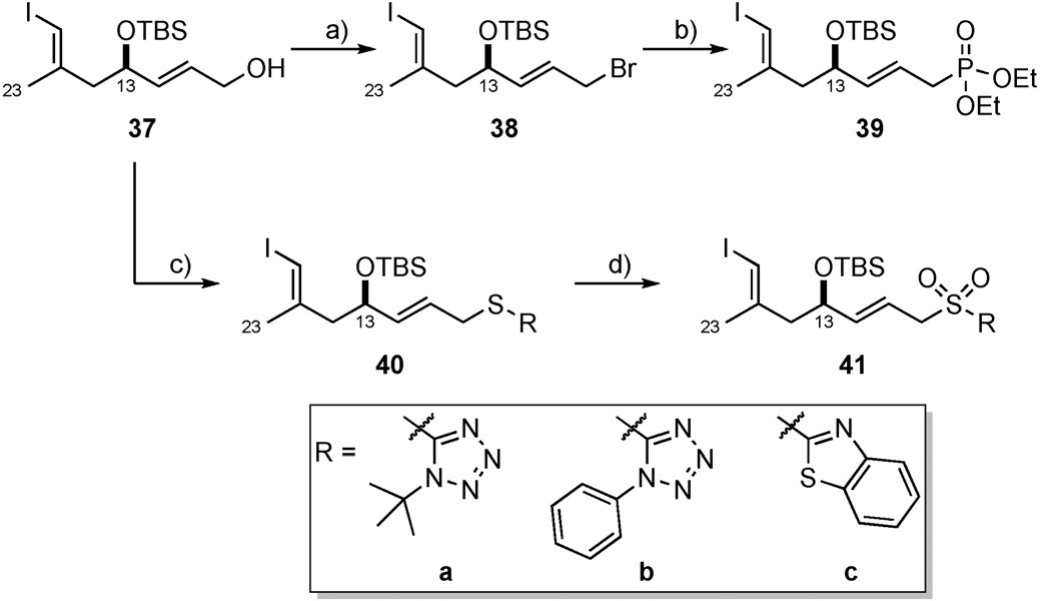
a) PPh_3_, CBr_4_, CH_2_Cl_2_, −40 °C; b) P(OEt)_3_, 100 °C, 80 % over 2 steps; c) 40 a: 1-*tert*-butyl-1*H*-tetrazole-5-thiol, PPh_3_, DIAD, THF, RT, 85 %; 40 b: 1-phenyl-1*H*-tetrazole-5-thiol, PPh_3_, DIAD, THF, RT, 96 %; 40 c: mercaptobenzothiazole, PPh_3_, DIAD, THF, RT, quant.; d) 41 a: Mo_7_O_24_(NH_4_)_6_⋅4 H_2_O, EtOH, H_2_O_2_, 0 °C→RT, 76 %; 41 b: Mo_7_O_24_(NH_4_)_6_⋅4 H_2_O, EtOH, H_2_O_2_, 0 °C→RT, 82 %; 41 c: Mo_7_O_24_(NH_4_)_6_⋅4H_2_O, EtOH, H_2_O_2_, 0 °C→RT, 85 %. DIAD=diisopropylazodicarboxylate.

The allylic alcohol **37** was converted either to ethyl phosphonate **39** by using a standard two-step Appel^29^/Arbuzov^30^ procedure, or directly by a Mitsunobu^31^ reaction to afford a variety of sulfides (**40 a**–**c**) (Scheme 8). Although we could oxidise each TBS-containing sulfide smoothly to the corresponding sulfone (**41 a**–**c**) using ammonium molybdate tetrahydrate and H_2_O_2_, the analogous TES-protected compound (not depicted) resulted in multiple side reactions and decomposition products under the conditions as well as with other oxidants (*m*CPBA, MMPP, oxone).

**b)** **Organocatalytic approach to the**­ ***trans*****-chlorocyclopropane**: As mentioned previously, it was hoped that the *trans*-chlorocyclopropane unit could be accessed by means of an intramolecular organocatalytic asymmetric cyclopropanation process developed in our laboratory. Previous studies had shown that these methods could deliver *trans*-configured cyclopropanes of this type in relatively high yield and in good to excellent diastereo- and enantioselectivity.^14^

**Scheme 9:**
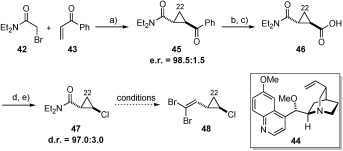
a) 44 (20 mol %), Cs_2_CO_3_, MeCN, 80 °C, 82 %; b) Urea⋅H_2_O_2_, HFIP, TFAA, 0 °C→RT; c) NaOH, H_2_O, MeCN, 50 °C, 48 % over 2 steps; d) SOCl_2_, RT, 98 %; e) 2-mercaptopyridine *N*-oxide sodium salt, TBAI (20 mol %), DMAP (20 mol %), CCl_4_, RT, then AIBN, 80 °C, 57 %. AIBN=2,2′-azobisisobutyronitrile; HFIP=hexafluoroisopropanol; TBAI=tetrabutylammonium iodide.

The synthesis began by reaction of α-bromo amide **42** with acrylphenone (**43**) to generate *trans*-configured cyclopropane **45** (e.r.=98.5:1.5) in good yield (82 %) (Scheme 9). The use of these coupling partners importantly provided synthetic handles to allow differentiation between the C20- and C21-positions of the cyclopropane ring. In order to introduce the chlorine substituent at C21 by a modified Hunsdiecker^32^ reaction (in analogy to Trost^4^, ^33^), an oxidation state change was required to allow formation of a carboxylic acid precursor.

Synthesis of the ester could be achieved using the Baeyer–Villiger reaction;^34^ however, this itself caused a number of issues. The oxidation was often found to be exceptionally sluggish and gave variable reaction times (2–14 days), often with incomplete conversion. Furthermore, following ester saponification, removal of the phenol generated was difficult and tedious.^35^ In an attempt to circumvent these issues, organocatalytic cyclopropanation using benzyl acrylate (correct oxidation level, ester removable by hydrogenolysis) was investigated. Unfortunately, cyclopropanation resulted in significantly reduced yield and enantioselectivity (not depicted).14c

Undeterred, acid chloride formation and Hunsdiecker reaction pleasingly gave the desired *trans*-configured chlorocyclopropane **47** in excellent diastereoselectivity (d.r.=97:3) in moderate yield over two steps. Although reliable, scale-up of this sequence was impacted by the requirement for the high dilution conditions employed (0.02 M, CCl_4_). Having set the desired stereochemistry of this key fragment in high diastereo- and enantioselectivity, we attempted to reduce the amide functionality in preparation for the synthesis of versatile dibromoolefin **48**, which had been used in previous approaches to the callipeltosides.^4^–^10^ Application of a variety of different reducing conditions ([Cp_2_Zr(H)Cl], LiBHEt_3_, DIBAL-H, DIBAL-H/*n*BuLi and NH_3_**⋅**BH_3_/*n*BuLi) gave either no desired product, mixtures, or could not be reproduced in a reliable fashion.^36^ Despite further experimentation, work-arounds, and attempts to deliver significant quantities of material, we were forced to abandon this organocatalytic approach to the *trans*-chlorocyclopropane unit and consider a more scalable alternative.

**Scheme 10:**
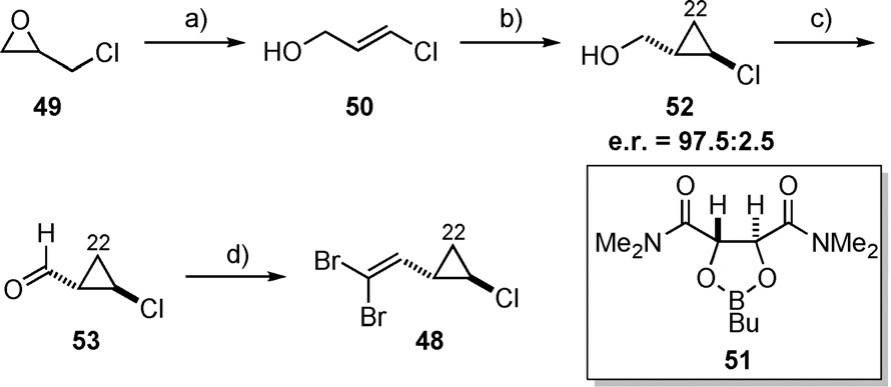
a) *n*BuLi, TMEDA, THF −78 °C, 70 %; b) Et_2_Zn, CH_2_I_2_, CH_2_Cl_2_, 0 °C, then 50, 51, CH_2_Cl_2_, 0 °C→RT, 74 %; c) PCC, celite®, CH_2_Cl_2_, RT; d) PPh_3_, CBr_4_, CH_2_Cl_2_, 0 °C→RT, 70 % over 2 steps. PCC=pyridinium chlorochromate; TMEDA=*N*,*N*,*N*’,*N*’-tetramethylethylenediamine.

**c)** **Alternative approach to the**­ ***trans*****-chlorocyclopropane unit**: Paterson^6^, ^7^ and Panek^8^ have both shown that the asymmetric Simmons–Smith reaction developed by Charette^37^ provided rapid access to dibromoolefin **48** in only four steps from *epi*-chlorohydrin. This being the case, we also chose to assess this sequence for the preparation of this fragment.^38^ As expected, the methodology was robust, delivering the desired cyclopropyl alcohol in excellent levels of enantioselectivity (e.r.=97.5:2.5). This could then elaborated to the desired dibromoolefin (**48**) in a further two steps (Scheme 10).

After having gained access to reasonable quantities of dibromoolefin **48** and also having implemented a versatile approach to ethyl phosphonate **39** and several Julia–Kocienski coupling partners (**41 a**–**c**), we sought to install the C17–C19 portion of key vinyl iodide **6** and investigate the stereoselective formation of the C16–C17 *E*-configured alkene.

**Scheme 11:**
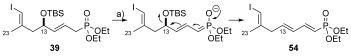
a) LiHMDS, THF, −78 °C; proposed decomposition of phosphonate 38.

**d)** **Initial approach to vinyl iodide 6 (PG=TBS (55))**: Preliminary studies towards the construction of the C16–C17 bond using phosphonate **39** quickly showed this to be an unproductive route to the fragment. Treatment of phosphonate **39** using LiHMDS, perhaps unsurprisingly only led to the elimination of the C13–OTBS group to provide conjugated diene **54** (Scheme 11). In order to make the addition of phosphonate **39** to aldehyde **28** competitive with elimination of the C13–OTBS group, **28** and **39** were pre-mixed prior to the slow addition of LiHMDS. However, this process resulted in recovered phosphonate **39** and decomposition of the aldehyde. Use of a weaker base such as Cs_2_CO_3_ also proved ineffective in producing the desired product **55**.

Our attention turned to the study of the Julia–Kocienski olefination. These investigations began with sulfone **41 a** (Scheme 8) which, in combination with Cs_2_CO_3_ in THF/DMF (3:1) at room temperature gave vinyl iodide **55** in moderate yield (54 %), but disappointingly as a mixture of *Z*/*E* isomers favouring the *Z* form (6:1) (Table 1, entry 1). Changing the solvent to solely DMF afforded a slight improvement in the *Z*/*E* ratio (5:1) (entry 2). In order to improve the observed selectivity further we reasoned that the synthesis of sulfones enabling the stabilisation of the negative charge on the tetrazole unit would lower the energy of the zwitterionic intermediate reaction pathway more than the non-zwitterionic reaction pathway. Therefore we employed phenyltetrazole sulfone **41 b** (entry 3). This led to a much improved, but still unacceptable *Z*/*E* (1:1) ratio. Further modification by use of sulfone **41 c** offered no improvement in terms of selectivity (entry 4) whilst additional experimentation using **41 b** by altering the base, solvent and temperature (entries 5–7) used in the reaction also did not favour the desired *E*-isomer.^39^

**Table 1:** Conditions for the formation of the C16–C17 bond.^[a]^

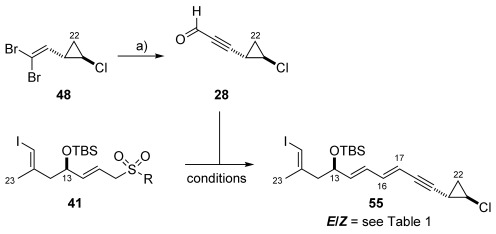				
Entry	Sulfone	Conditions	*Z*/*E*^[b]^	Yield [%]^[c]^
1	**41 a**	Cs_2_CO_3_, THF/DMF (3:1), RT, 15 h	6:1	54
2	**41 a**	Cs_2_CO_3_, DMF, RT, 6 h	5:1	n.d.^[d]^
3	**41 b**	Cs_2_CO_3_, DMF, RT, 15 h	1:1	44
4	**41 c**	Cs_2_CO_3_, DMF, RT, 6 h	1.4:1	n.d.^[d]^
5	**41 b**	KHMDS, 18-crown-6, DMF, −50 °C, 6 h	1.3:1	n.d.^[d]^
6	**41 b**	Cs_2_CO_3_, DMF, 0 °C, 6 h	1.4:1	40^[e]^
7	**41 b**	Cs_2_CO_3_, DMPU, RT, 15 h	1:1	52

[a] Reagents and conditions: a) i) *n*BuLi, THF, −78 °C, (CHO)_*n*_, 71 %; ii) MnO_2_, CH_2_Cl_2_, RT, used directly. [b] Determined by ^1^H NMR spectroscopy of the crude reaction mixture. [c] Yield based on sulfone **41**. [d] Not determined (n.d.). [e] **41 b** recovered (15 %).

**Scheme 12:**
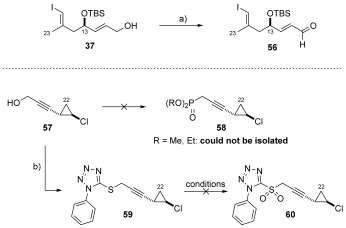
a) MnO_2_, CH_2_Cl_2_, RT, used directly; b) 1-phenyl-1*H*-tetrazole-5-thiol, PPh_3_, DIAD, THF, RT, 89 % (based on 1-phenyl-1*H*-tetrazole-5-thiol).

In an attempt to further improve the *E*/*Z* selectivity, we decided to reverse the phosphonate and sulfone coupling partners such that they were derived from the cyclopropane unit (**58** and **60**), with the aldehyde prepared from the C10–C16 fragment (**37**) (Scheme 12). However, although the aldehyde **56** could be prepared easily from the corresponding alcohol (**37**) using MnO_2_, we found that phosphonate **58** (in which R=Me or Et) could not be isolated. In addition, the preparation of the sulfone **60** by oxidation of sulfide **59** proved problematic (*m*CPBA, oxone, H_2_O_2_/Mo_7_O_24_(NH_4_)_6_**⋅**4 H_2_O) and could not be achieved. These factors, coupled with the poor selectivity observed in the Julia–Kocienski coupling between aldehyde **28** and sulfone(s) **41 a**–**c** dictated that we reassess our approach to vinyl iodide **55**.

### Formation of the C14–C15 bond

**Scheme 13:**
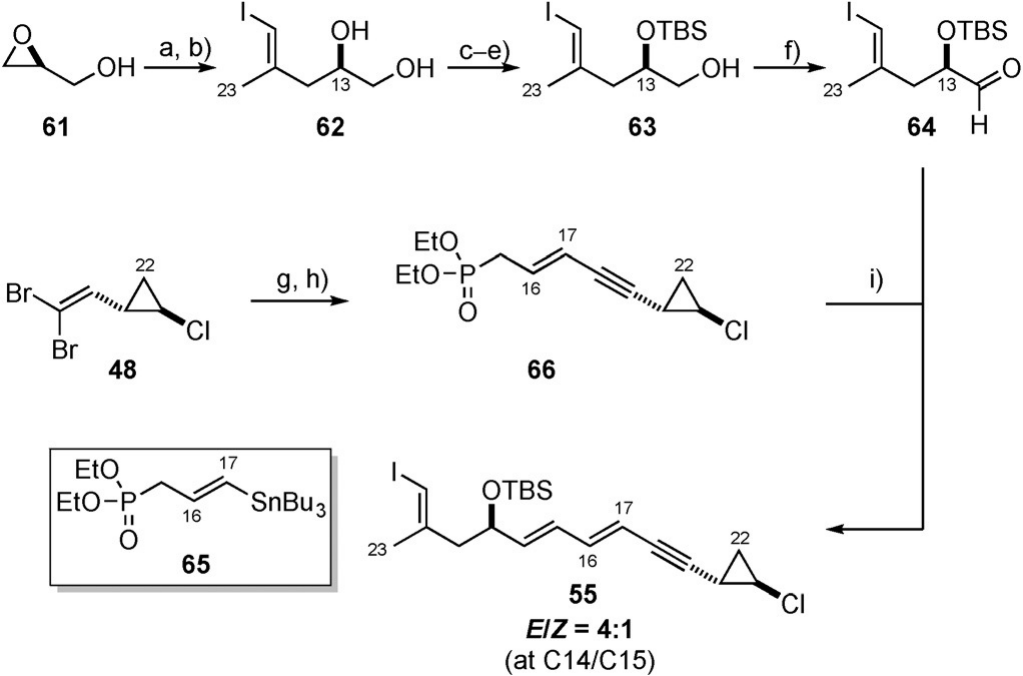
a) Lithium acetylide, ethylene diamine complex, HMPA, THF, RT, 50 %; b) i) [(Cp)_2_ZrCl_2_], Me_3_Al, 1,2-dichloroethane, RT; ii) I_2_, THF, −30 °C, 60 %; c) PivCl, pyr, 0 °C; d) TBSOTf, 2,6-lutidine, −78 °C; e) DIBAL-H, CH_2_Cl_2_, −78 °C, 80 % over 3 steps; f) oxalyl chloride, DMSO, Et_3_N, CH_2_Cl_2_, −78 °C, quant.; g) TBAF, DMF, 65 °C; h) 65, [Pd_2_(dba)_3_] (2 mol %), AsPh_3_ (8 mol %), THF, 60 °C, 56 % over 2 steps; i) LiHMDS, THF, −78 °C, 80 %. dba=dibenzylideneacetone; Cp=cyclopentadienyl; HMPA=hexamethylphosphoramide; LiHMDS=lithium hexamethyldisilazide.

**Horner–Wadsworth–Emmons (HWE) approach**: As our investigations into olefination-based methods to form the C16–C17 bond yielded only poor results (low yields, poor selectivity) we chose to briefly investigate the formation of the C14–C15 alkene using a Horner–Wadsworth–Emmons olefination (Scheme 13). This route required the use of phosphonate **66**, common to several of the published callipeltoside syntheses.^4^, ^9^ In a novel approach, phosphonate **66** was synthesised in two steps from the dibromoolefin, by TBAF-induced elimination and Stille cross-coupling^40^ using stannane **65**^41^ in moderate yield. This reaction was found to be robust and scalable, and was not optimised to improve the yield of the product **66**. The required aldehyde **64** was synthesised from (*S*)-glycidol in 6 steps. Epoxide opening, followed by carboalumination/iodination yielded diol **62**.^42^ Following protection of the primary alcohol (PivCl), TBS protection and pivolate reduction, alcohol **63** was produced in 80 % yield over three steps.^43^ Swern oxidation^44^ followed by HWE olefination using the literature conditions (LiHMDS, THF, −78 °C^8^), yielded the C10–C22 vinyl iodide in good yield as an inseparable 4:1 *E*/*Z* mixture. Unfortunately attempts to improve this ratio (i.e. alternative bases and lower temperature) gave no improvement in selectivity. Enrichment of the isomeric ratio by isomerisation was also attempted (similar to Evans^5^ and MacMillan^10^); however, this generally lead to extensive decomposition (including proto-deiodination) under a range of conditions employed. Although this approach was found to be reasonably scalable and provided significant quantities of vinyl iodide **55** for initial fragment union studies, focus remained on the development of a route to provide the vinyl iodide as a single *E*,*E*-isomer.

### Cross-coupling approach to vinyl iodide 6 (55)

**Scheme 14:**
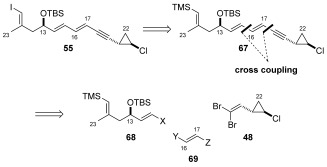
Cross-coupling approach to vinyl iodide 55 (6).

After having observed poor selectivity by disconnection of the C16–C17 bond, we chose therefore to disconnect vinyl iodide **55** at both the C15–C16 and C17–C18 junctions with the idea of performing sequential cross-coupling reactions (Scheme 14). Such an approach should provide vinyl iodide **55** in a stereospecific manner. To facilitate this approach it was necessary to mask the C10 vinyl iodide functionality as a vinyl silane, thereby preventing side reactions during the cross-coupling process. This design feature, as well as the previously noted issues concerning the isolation of aldehyde **33** using the organocatalytic oxyamination procedure, encouraged us to explore an alternate route to the C10–C15 unit.

**a) Synthesis of the C10**–**C15 vinyl silane fragment**: Assembly began from commercially available TMS-propyne **72** which, in the presence of 5 mol % of molybdenum catalyst **71**,^46^ could be regioselectively hydrostannylated to give a single isomer.^45^ Subsequent iodine-tin exchange gave vinyl iodide **73** in 62 % yield over two steps on a large scale (ca. 45 g).

With this material in hand, we elected to form the corresponding alkenylmetal species, with which opening of an appropriate (*S*)-configured epoxide could be achieved. This reaction was first carried out by addition of the corresponding higher-order heterocuprate (of **73**) to the lithium alkoxide of (*S*)-glycidol, affording diol **75** in 72 % yield (Table 1, entry 1).^47^ However, in the first attempt epoxide **61** was used as the limiting reagent, with epoxide ring-opening requiring a large excess (5.0 equiv) of vinyl iodide **73**.^48^ Although substrate **73** could be produced in multigram quantities, we sought to decrease further the number of equivalents of **73** such that greater quantities of **75** could be produced. Unfortunately, reducing the excess of vinyl iodide **73** resulted in lower isolated yields of diol **75** (Table 2, entries 2 and 3), while reaction with PMB-protected (*S*)-glycidol afforded a mixture of products (entry 4). Interestingly, lowering the number of equivalents of vinyl iodide **73** resulted in increasing amounts of byproduct **77**. The formation of compound **77** represented a rather unusual case whereby a *tert*-butyl group (presumably from *t*BuLi) had instead reacted with the lithium alkoxide of (*S*)-glycidol. Although there is literature precendent for the opening of an epoxide with both *t*BuLi and its corresponding lower-order homocuprate,^49^ it is unknown which species is responsible for the formation of **77** in this instance. Unable to prevent the formation of byproduct **77** (or **78**), we turned to the direct opening of epoxide **74** using the organolithium species of **73**.^50^ Adjustment of the stoichiometry of the reaction indicated that an excess of PMB-protected epoxide **74** (2.5 equiv) was optimal, producing the desired product in 53 % yield (following TBS protection) and in multigram quantities (entry 7).

**Table 2:** Conditions for epoxide ring opening.^[a]^

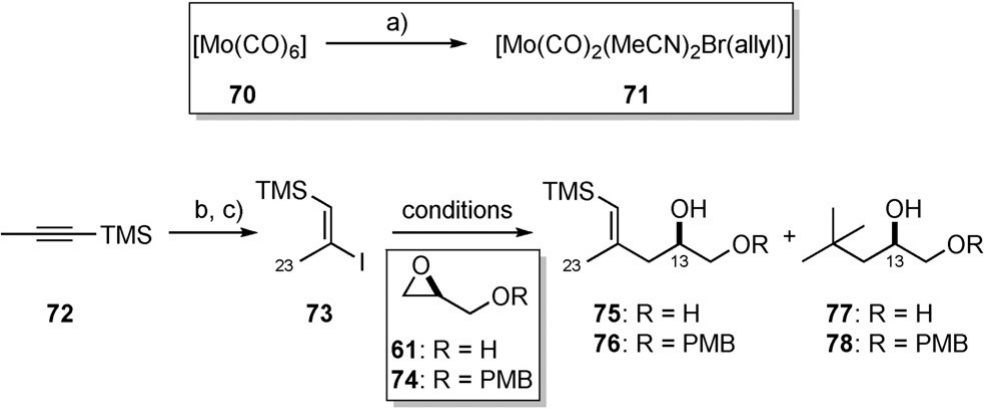				
Entry	Conditions	Epoxide [equiv]	Vinyl iodide73[equiv]	Yield [%]
1	thiophene, *n*BuLi, CuCN, *t*BuLi, THF, BF_3_⋅OEt_2_, −78→−90→−40 °C	**61** (1.0)	5.0	72
2	as for entry 1	**61** (1.0)	3.5	55
3	as for entry 1	**61** (1.0)	2.0	33
4	as for entry 1	**74** (1.0)	5.0	60^[a]^
5	*i*PrMgCl, CuI,^[b]^ THF, −78 °C	**74** (1.0)	1.2	–
6	*t*BuLi, BF_3_**⋅**OEt_2_, PhMe, −78 °C	**74** (1.0)	2.0	11
7	*t*BuLi, BF_3_**⋅**OEt_2_, PhMe, −78 °C	**74** (2.5)	1.0	53^[c]^

[a] Reagents and conditions: a) allyl bromide, MeCN, PhH, 85 °C, 44 %; b) **71** (5 mol %), Bu_3_SnH, THF, RT; c) I_2_, CH_2_Cl_2_, 0 °C, 62 % over 2 steps. [b] Compound could not be purified fully. [c] 25 mol % of CuI was used. [d] Following TBS protection.

**Scheme 15:**
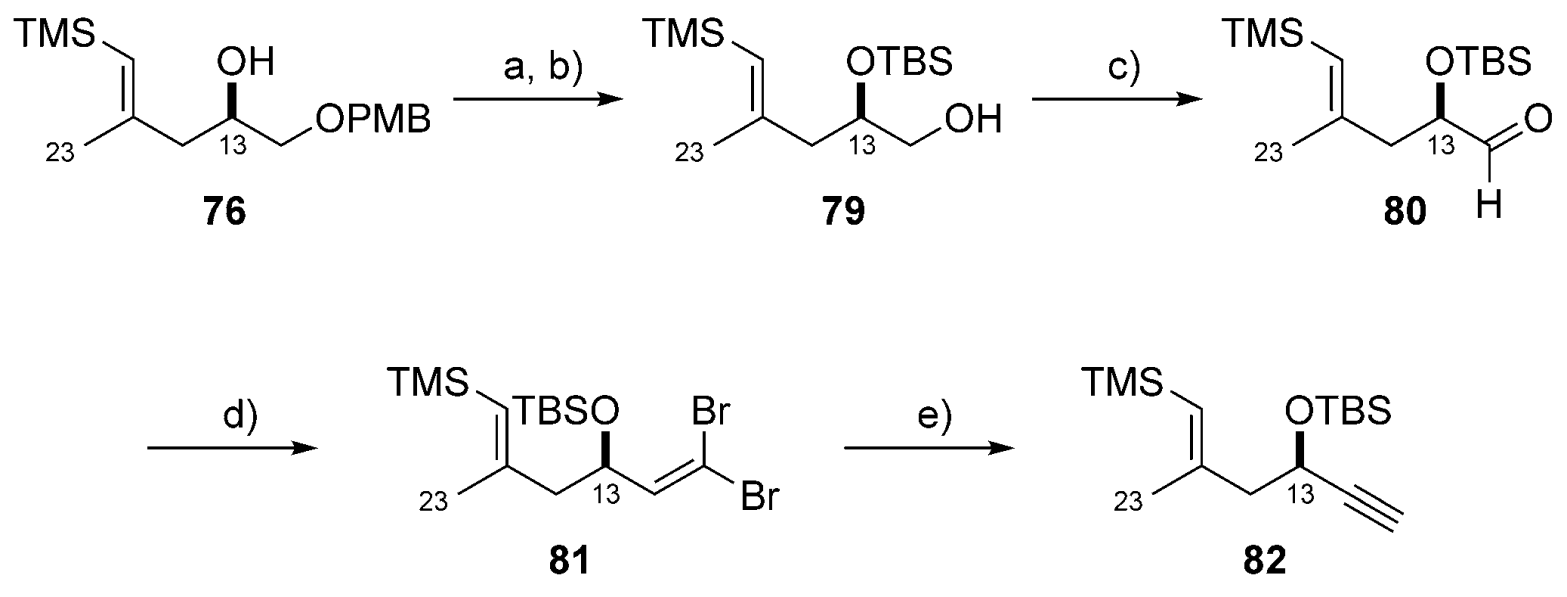
a) TBSOTf, 2,6-lutidine, CH_2_Cl_2_, −78 °C, 53 % over 2 steps from 73; b) DDQ, pH 7 phosphate buffer, CH_2_Cl_2_, 0 °C, 89 %; c) SO_3_⋅pyr, DMSO, Et_3_N, CH_2_Cl_2_, 0 °C→RT; d) PPh_3_, CBr_4_, 2,6-lutidine, 0 °C, 89 % over 2 steps; e) *n*BuLi, THF, −78 °C→RT, then H_2_O, 85 %.

Following ring opening of PMB-protected epoxide **74**, a two step TBS protection/PMB deprotection afforded alcohol **79**. After some optimisation, aldehyde **80** was synthesised using Parikh–Doering conditions,^51^ with subsequent Ramirez dibromoolefination^52^ providing compound **81** in 89 % yield over two steps.^53^ Finally, Corey–Fuchs reaction^54^ delivered terminal alkyne **82**, with which hydrometallation studies could be conducted (Scheme 15).

Firstly, hydrozirconation of alkyne **82** using the Schwartz reagent ([(Cp)_2_Zr(H)Cl])^55^ followed by iodine exchange was investigated. Unfortunately, this approach provided a mixture of desired (*E*)-vinyl iodide **84** as well as protodesilylated compound **85** in a 1:2 ratio (by ^1^H NMR spectroscopy). In a different approach, aldehyde **80** was found to be poorly reactive towards Takai olefination^56^ conditions and only starting material was isolated or trace conversion to a mixture of **84** and **85**. Pleasingly, regioselective Pd^0^-catalysed hydrostannylation of alkyne **82**^57^ could be achieved which, following the dropwise addition of iodine, gave the desired (*E*)-vinyl iodide **84** in 49 % yield over two steps.

**Scheme 16:**
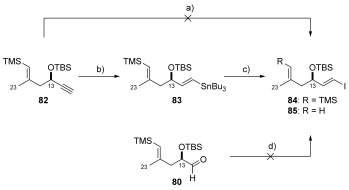
a) [(Cp)_2_Zr(H)Cl], THF, RT, then I_2_; b) Bu_3_SnH, [Pd(PPh_3_)_2_Cl_2_] (3 mol %), THF, 0 °C; c) I_2_, CH_2_Cl_2_, −78 °C, 49 % over 2 steps; d) CrCl_2_, THF, 0 °C, then 80, THF, CHI_3_, RT→50 °C.

Synthesis of vinyl iodide **84** by this method was advantageous since both (*E*)-vinyl iodide **84** and (*E*)-vinyl stannane **83** could be used as coupling partners in the Stille reaction (Scheme 16). This protocol was used for the synthesis of multigram quantities of (*E*)-vinyl stannane **83** and (*E*)-vinyl iodide **84**, which could be straightforwardly isolated and purified.

**Scheme 17:**
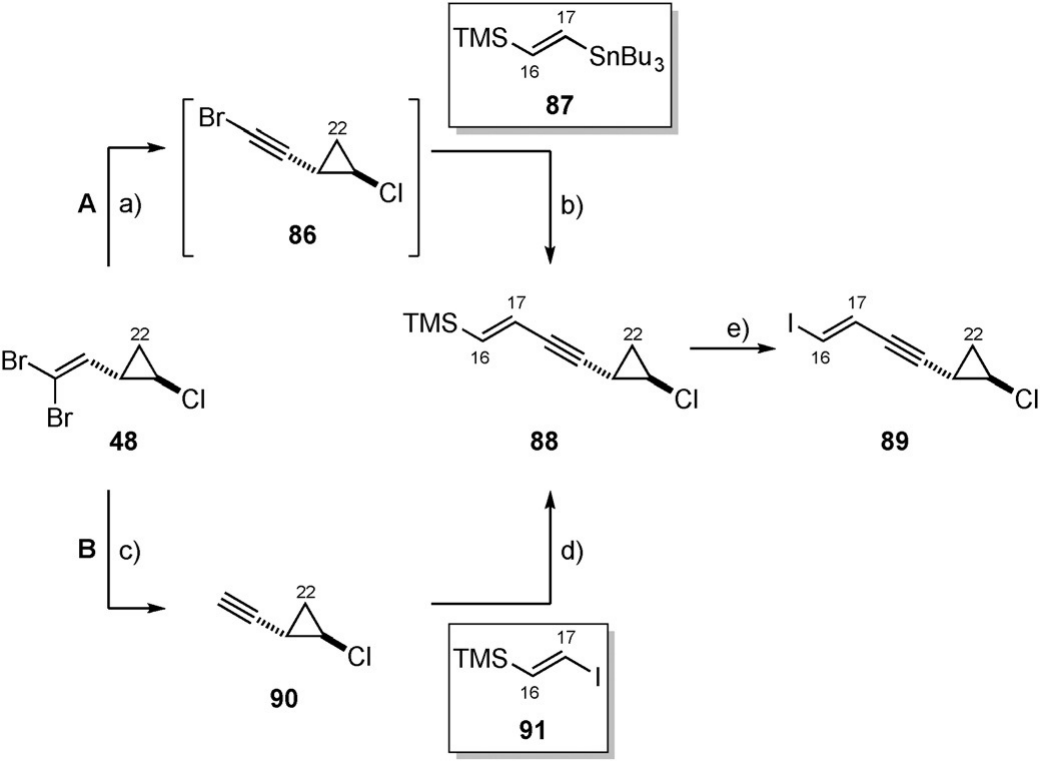
a) TBAF, DMF, 65 °C; b) [Pd(MeCN)_2_Cl_2_] (18 mol %), DMF, RT, 23 % over 2 steps; c) *n*BuLi, Et_2_O, −78 °C then H_2_O; d) [(PPh_3_)_2_PdCl_2_] (7 mol %), CuI (21 mol %), MeCN, RT then Et_3_N, 0 °C, 82 % (from 91); e) Py_2_IBF_4_, HBF_4_⋅OEt_2_, CH_2_Cl_2_, 0 °C, 96 %.

**b) Synthesis of the C16–C22 coupling partners**: With knowledge that both (*E*)-vinyl stannane **83** and (*E*)-vinyl iodide **84** could be accessed straightforwardly, the synthesis of their respective (*E*)-vinyl iodide (**89**) and (*E*)-vinyl stannane (**93**) (shown in Table 3, below) coupling partners began. Initial attempts at generating vinyl iodide **89** focused on a Takai olefination^56^ of propargylic aldehyde **28** (shown in Table 1). Although this strategy delivered the desired vinyl iodide **89**, no selectivity between the *E*- and *Z*-isomers was observed (1:1). Reassessment of the approach led us to target (*E*)-vinyl silane **88**, since silicon-iodine exchange would be expected to proceed with retention of configuration (Scheme 17). Construction of this compound began with the formation of volatile monobromide **86** following elimination using TBAF. Bromoalkyne **86** was then coupled with trimethyl[(*E*)-2-(tributylstannanyl)ethenyl]silane **87**^58^ to give the desired (*E*)-vinyl silane **88** in poor yield (23 %) (Scheme 17, route A). Since this particular Stille coupling was low yielding, a second route to enable the synthesis of compound **88** was explored (Scheme 17, route B). This involved a Sonogashira reaction^59^ between known volatile alkyne **90**^6^, ^7^ and vinyl iodide **91**; itself derived by iodination of **87**. Reassuringly, this gave a reproducible yield of 82 % and was optimised such that only 2.5 equivalents of alkyne **90** was required.^60^ This proved a much more effective way to access vinyl silane **88**. To deliver the desired vinyl iodide **89**, silicon-iodine exchange was initially conducted by treatment of vinyl silane **88** with either I_2_ (CH_2_Cl_2_ and THF solutions) or with NIS. On both occasions the use of I_2_ resulted in the generation of complex mixtures, with NIS surprisingly favouring the formation of the undesired *Z*-isomer (3:1). However, further experimentation revealed that a combination of the Barleunga reagent^61^ with HBF_4_**⋅**OEt_2_ at 0 °C afforded the desired *E*-isomer exclusively in an impressive 96 % yield (Scheme 17).

At this point attention switched to the synthesis of the alternate (*E*)-vinyl stannane coupling partner (**93**, Table 3). The synthesis of vinyl stannane **93** relied on the Stille coupling between monobromide **86** and bis-stannane **92**.^62^ However, despite the investigation of a number of different literature known Stille conditions using a variety of different catalyst systems, [Pd_2_(dba)_3_]/AsPh_3_ (1:4),^63^ [Pd(MeCN)_2_Cl_2_],^64^ [Pd(PPh_3_)_4_],^65^ [Pd(PFur_3_)_2_Cl_2_],^66^ vinyl stannane **93** could only be produced in a disappointing 6–10 % yield over 2 steps (from dibromide **48**). On each occasion only desired vinyl stannane **93** and bis-stannane (**92**) could be isolated from the reaction mixture, with substantial amounts of unidentifiable decomposition products observed. Since no set of conditions had proven superior for this transformation, Farina’s catalyst system^63^ ([Pd_2_(dba)_3_], AsPh_3_) was chosen as a basis for further optimisation, with the amount of bis-stannane **92** maintained at two equivalents throughout (Table 3).

**Table 3:** Conditions for C17–C18 bond formation.^[a]^

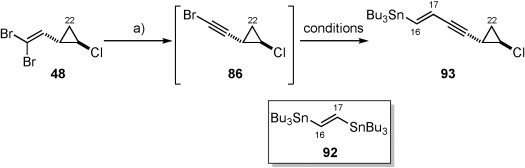					
Entry	[Pd_2_(dba)_3_] [mol %]	AsPh_3_ [mol %]	Additive [mol %]	*T* [°C]	Yield^[a]^ [%]
1	2	8	–	60	8
2	2	8	–	25	6
3	2	8	–	0	7
4	10	40	–	0	15
5	10	40	–	−10	32
6	10	40	–	−20	23
7	10	40	Ag_2_CO_3_ (25)	−10	35
8	10	40	Ag_2_CO_3_ (100)	−10	45^[b]^

[a] Reagents and conditions: a) TBAF, DMF, 65 °C. [b] Yield over 2 steps. [c] Yield reproduced on 9.85 mmol scale.

Initial investigations revealed that the reaction time and temperature had little effect on the isolated yield of (*E*)-vinyl stannane **93** (Table 3, entries 1–3) when [Pd_2_(dba)_3_] and AsPh_3_ were used in 2 and 8 mol % quantities. However, the isolated yield doubled when a five-fold increase in both [Pd_2_(dba)_3_] and AsPh_3_ were administered at 0 °C (entry 4). Further cooling of the reaction to −10 °C proved optimal, producing a much-improved yield of 32 % (entry 5). We then chose to add Ag_2_CO_3_ to the reaction mixture since it could potentially act as both an acid and halide scavenger.^67^ Pleasingly, addition of one equivalent (entry 8) of Ag_2_CO_3_ resulted in an acceptable isolated yield of 45 % over two steps, which could be reproduced on a gram-scale to provide vinyl stannane **93**. In order to assess the effect of alternative additives in the reaction several different bases (organic and inorganic, Table 4, entries 1 and 2), and silver sources (entries 3 and 4) were evaluated, whilst the stoichiometry of Ag_2_CO_3_ was also doubled (entry 5). Disappointingly, however, the isolated yield was found to be reduced in all cases. At this stage the role of the Ag_2_CO_3_ in these reactions remains unclear, and has not been investigated in detail.

**Table 4:** Further conditions for C17–C18 bond formation.

Entry	Additive	Amount [equiv]	Yield [%]^[a]^
1	DIPEA	1.0	25
2	Cs_2_CO_3_	1.0	25
3	AgOAc	1.0	20
4	Ag_2_O	1.0	28
5	Ag_2_CO_3_	2.0	36

[a] Yield over 2 steps. DIPEA=*N*,*N*-diisopropylethylamine.

**Scheme 18:**
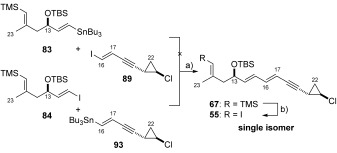
a) [Pd(PFur_3_)_2_Cl_2_] (15 mol %), DMF, RT, 63 %; b) NIS, MeCN, RT, 84 %. NIS=*N*-iodosuccinimide.

**c) Stereospecific synthesis of (*E*)-vinyl iodide 55 (6)**: With both vinyl iodide **89** and vinyl stannane **93** in hand, their Stille reactions with respective coupling partners **83** and **84** were explored Scheme 18. Attempts to construct the C15–C16 bond by a Stille reaction between vinyl stannane **83** and vinyl iodide **89** under several different conditions ([Pd_2_(dba)_3_]/AsPh_3_ (1:4), [Pd(PFur_3_)_2_Cl_2_]) led to either no reaction or only trace conversion to the desired product. As a result of the poor observed reactivity, we were prompted to investigate the Stille cross-coupling between reversed coupling partners **84** and **93**. Indeed reaction between vinyl iodide **84** and vinyl stannane **93** could be achieved in the presence of freshly prepared [Pd(PFur_3_)_2_Cl_2_] (15 mol %).^66^ This gave C10–C22 vinyl silane **67** in 63 % yield and importantly as the desired single (*E*,*E*)-isomer. Having successfully obtained the complete di-ene-yne unit in the required configuration, we were pleased to find that the requisite silicon–iodine exchange occurred with complete retention of configuration (confirmed by no nOe correlation, see the Supporting Information), delivering the fully elaborated C10–C22 vinyl iodide fragment **55** (**6**) as a single isomer. This route provided C10–C22 vinyl iodide **55** in a scalable longest linear sequence of 12 steps (from TMS-propyne (**72**)] and in 5.7 % overall yield.

### Union of pyran 5 with vinyl iodide 554 and completion of the callipeltoside aglycon (4)

**Scheme 19:**
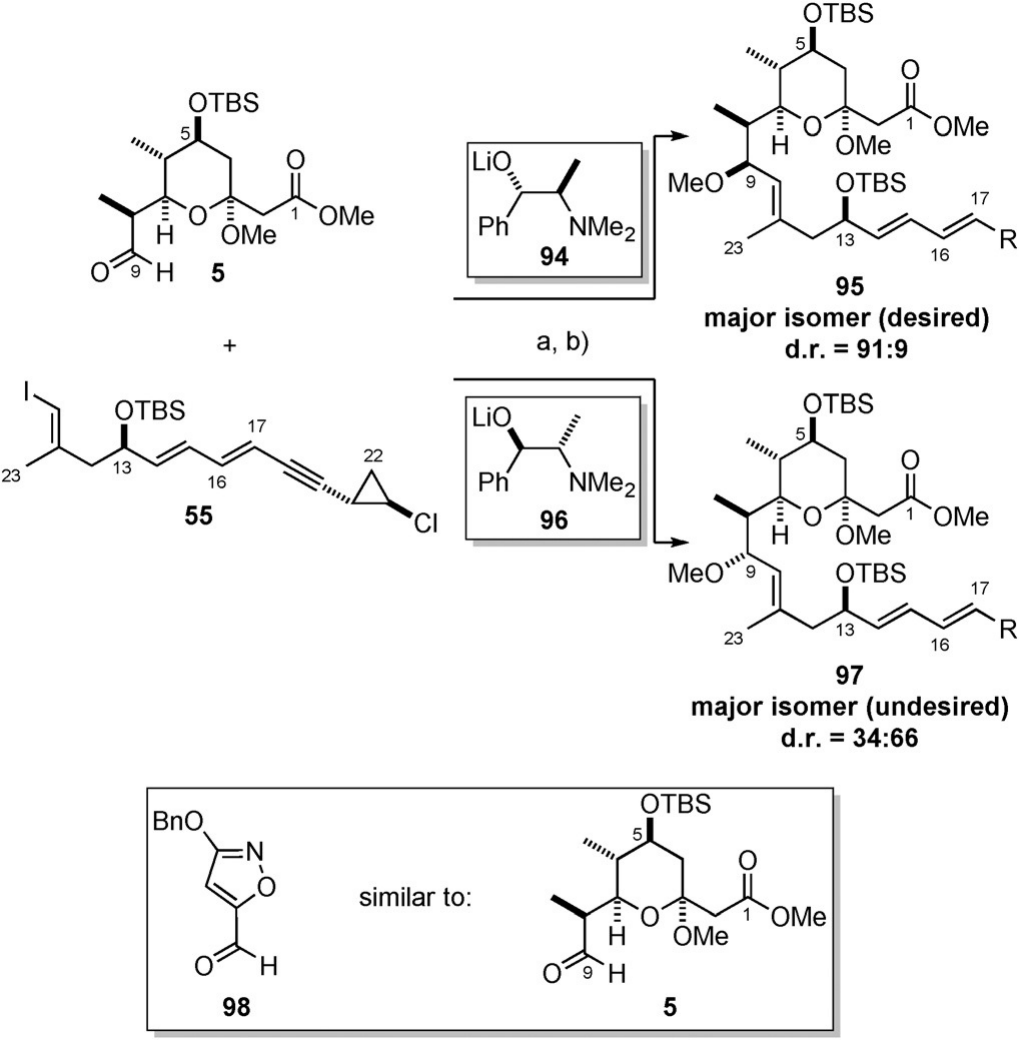
a) i) 55, *t*BuLi (2.8 equiv), Et_2_O, −78 °C; ii) ZnBr_2_ (0.9 M in Et_2_O, 1.35 equiv), 0 °C; iii) 94 (or 96) (1.1 equiv), PhMe, 0 °C; iv) 5, PhMe, 0 °C, 48 %; b) MeOTf, 2,6-di-*tert*-butylpyridine, CH_2_Cl_2_, RT, 73 %.

With routes providing gram-scale quantities of both pyran **5** and vinyl iodide **55** in place, our next major challenge was to address the diastereoselective union of these fragments to form the C9 stereocentre. In the first instance, the coupling of C10–C22 vinyl iodide **55** and pyran aldehyde **5** was attempted in the absence of any chiral additives. Reaction of the corresponding alkenylzinc species of **55** (formed by iodine–lithium exchange followed by transmetallation with ZnBr_2_) with pyran aldehyde **5** afforded a 1:1 mixture of C9 epimeric products. This important result was encouraging as it established for the first time, that a vinyl metal addition of the entire C10–C22 fragment to aldehyde **5** could be achieved. Encouraged by this result, we sought a method that would enable this key coupling to be conducted in a stereocontrolled manner. In order to achieve this, we investigated the elegant work by Oppolzer and Radinov,^68^ as well as the studies of Marshall,^69^ who had previously shown that the stereochemical information present in an appropriate enantioenriched lithio-*N*-methylephedrine alkoxide could be transferred to reactions of this type. Analysis of the proposed model revealed (1*R*,2*S*)-(−)-*N*-methylephedrine **96** to be the reagent of choice; but in practice, disappointing diastereoselectivity at C9 was observed (34:66). Undeterred, we chose to perform this reaction with the enantiomeric ligand, (1*S*,2*R*)-(+)-*N*-methylephedrine **94**, this time observing a much-improved diastereomeric ratio of 91:9 at the crucial C9 stereogenic centre. Methylation of both diastereomeric mixtures resulted in known compound **95**; which was previously described in the MacMillan synthesis of callipeltoside C.^10^ Comparison with the literature therefore provided rapid determination of the stereochemical outcome of these reactions (Scheme 19).

Pleasingly, the major product resulting from the matched addition of (1*S*,2*R*)-(+)-*N*-methylephedrine **94** resulted in the correct C9 stereochemistry for the callipeltosides. This result was welcome, but gave the opposite result to that predicted by the model’s suggested by Oppolzer^68^ and Noyori.^70^ A similar reversal in the selectivity was also observed by Myers^71^ in his synthesis of the tetracycline antibiotics. In order to account for this observation, Myers suggested that the aldehyde (in Myers case 3-benzyloxy-5-isoxazolecarboxaldehyde (**98**), shown in Scheme 19) formed a bis-chelate with the active metal complex, resulting in the exposure of the opposite enantiotopic face to reaction. We therefore speculate, but without evidence, that the presence of the pyran oxygen atom results in a similar chelation effect (for comparison, see Scheme 19).^72^

**Scheme 20:**
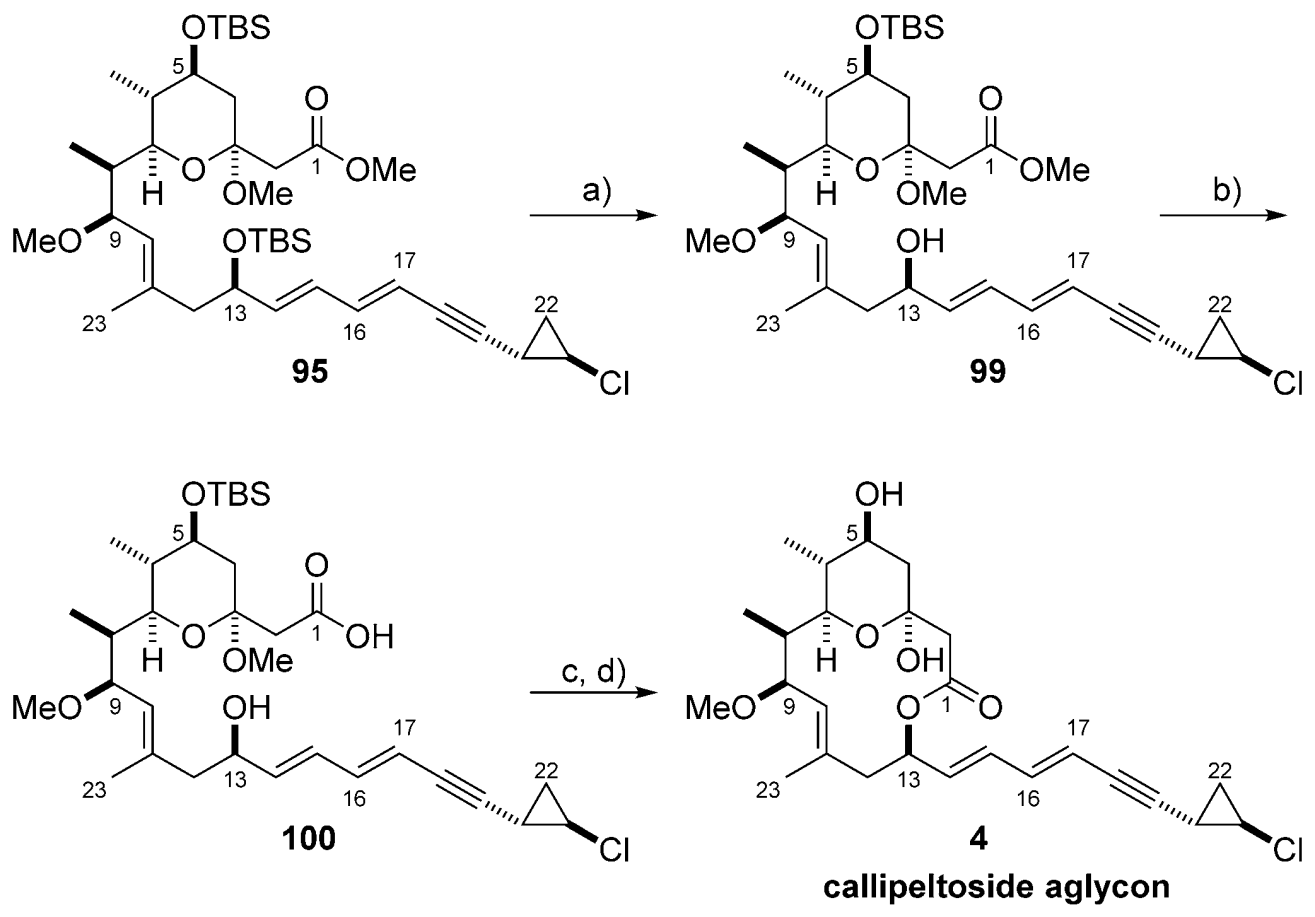
a) TBAF, THF, RT, 74 %; b) Ba(OH)_2_⋅8 H_2_O, MeOH, RT, quant.; c) 2,4,6-trichlorobenzoyl chloride, Et_3_N, PhMe, RT, then added to DMAP, PhMe, 80 °C; d) TFA, THF/H_2_O (5:1), RT, 58 % over 2 steps. TFA=trifluoroacetic acid.

With the advanced fragment **95** in hand, selective TBS deprotection was conducted using TBAF, with subsequent saponification of the ester functionality affording *seco*-acid **100**, ready for Yamaguchi macrolactonisation.^13^ Cyclisation to give the desired macrocycle was achieved, but also produced variable amounts of C3-acetal eliminated product (as observed by others), which could not be separated by flash column chromatography. However, this was inconsequential since treatment of the mixture with TFA in THF/H_2_O (5:1) reinstalled the hemi-ketal functionality and removed the C5-TBS group in a one-pot process to deliver the callipeltoside aglycon **4** in 58 % over two steps (Scheme 20).

### Synthesis of the callipeltoside sugar fragments

**Scheme 21:**
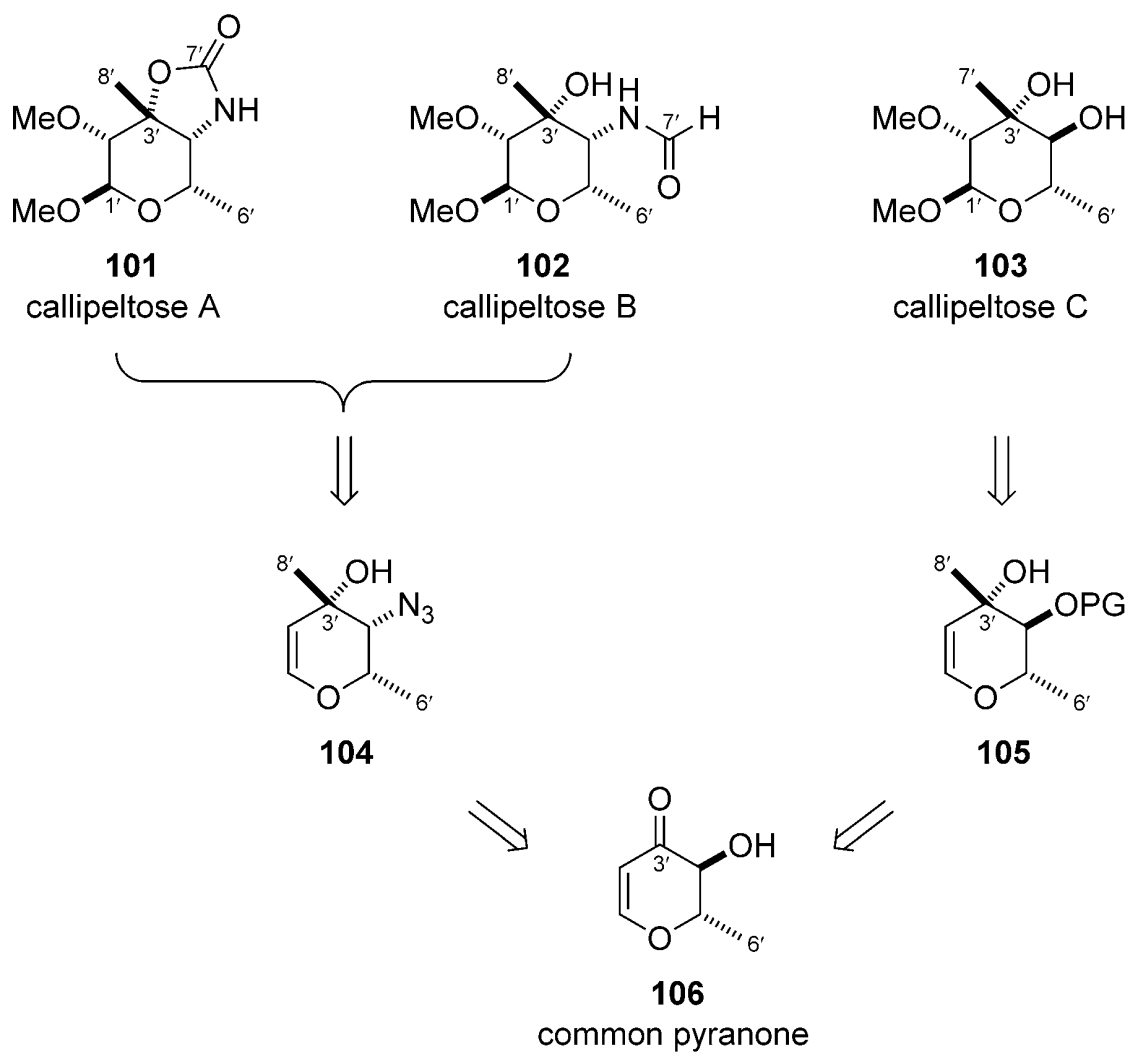
Retrosynthesis of callipeltose A, B and C.

Given the similarity of these fragments, we considered the possibility of beginning our studies from a common, readily available precursor. Since callipeltose A and B both contained nitrogen at the C4’-position, azido sugar **104** was chosen as an intermediate from which both of these sugars could be derived. In a similar manner, callipeltose C was to be accessed from C4′-epimeric compound **105**, instead containing a hydroxyl group at this position. Pyranone **106** was thereafter considered to be an appropriate common building block for the preparation of **104** and **105** (Scheme 21).

**Scheme 22:**
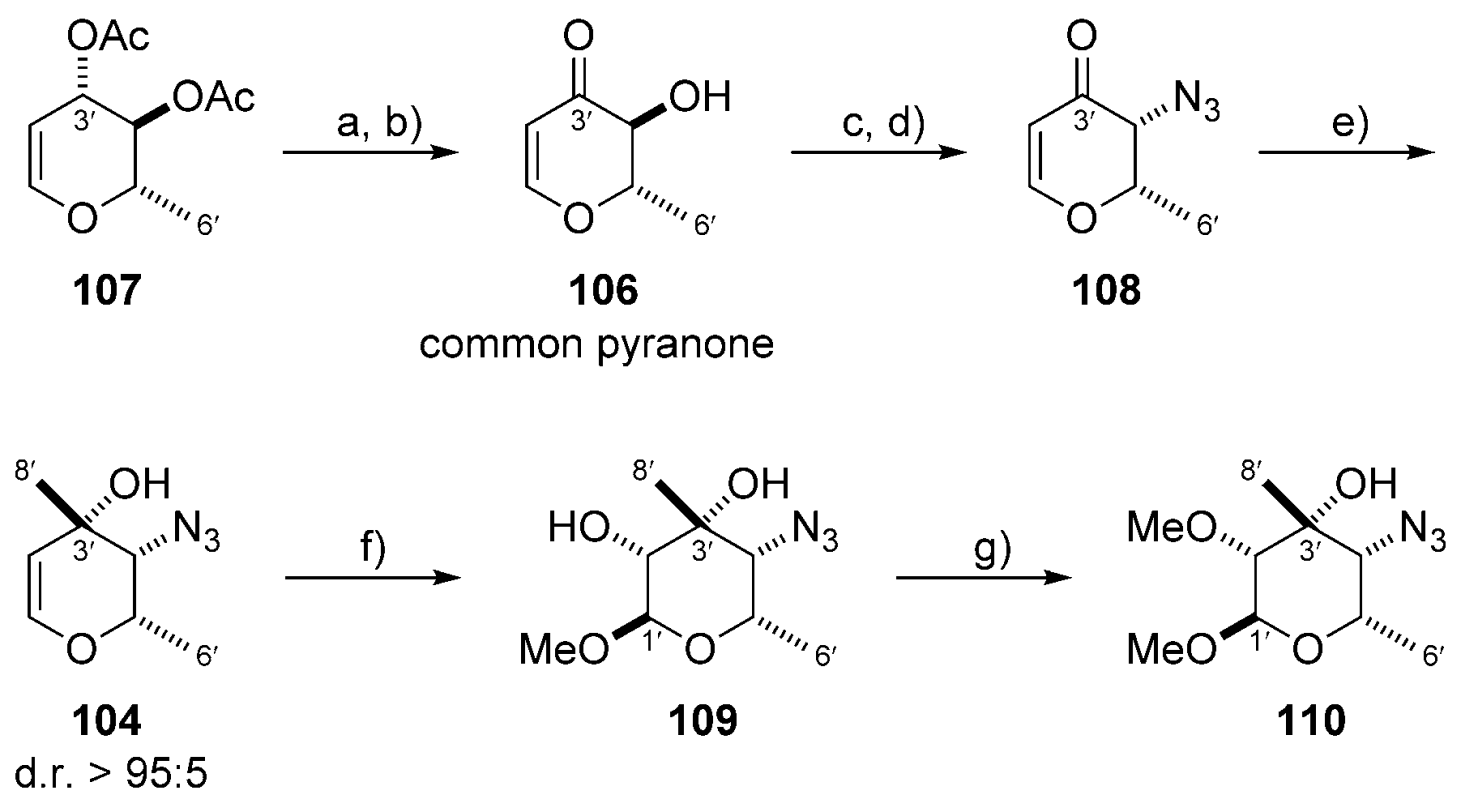
a) PS-Na_2_CO_3_, MeOH, RT; b) MnO_2_, CH_2_Cl_2_, RT, 74 % over 2 steps; c) NsCl, pyr, CH_2_Cl_2_, 0 °C→RT, 95 %; d) *n*Bu_4_NN_3_, CH_2_Cl_2_, 0 °C, 72 %; e) MeLi, THF, −100 °C, 79 %; f) *m*CPBA, NaHCO_3_, MeOH, 0 °C→RT, 52 %; g) KO*t*Bu, THF, 0 °C then MeI, 0 °C, 79 %. *m*CPBA=*meta*-chloroperoxybenzoic acid.

**a) Synthesis of common callipeltose A and B precursor 110**: Pyranone **106** was readily obtained from commercially available 3,4-di-*O*-acetyl-6-deoxy-L-glucal **107**, following deprotection using polymer supported Na_2_CO_3_, and allylic oxidation in 74 % over two steps without column chromatography required (Scheme 22). In keeping with Nicolaou’s synthesis of D-callipeltose A,^73^ we chose to activate the C4’ hydroxyl as its nosylate, and invert the stereocentre by displacement with *n*Bu_4_NN_3_. This gave pyranone **108** as a single diastereoisomer (>95:5 by ^1^H NMR spectroscopy). Following this, addition of MeLi at −100 °C occurred with complete diastereoselectivity to provide desired compound **104** in excellent yield (79 %). With the correct C3’ and C4’ stereochemistry in place, the remaining C1’ and C2’ stereocentres were installed by an epoxidation/methanolysis sequence to provide compound **109**. Finally, selective methylation of the C2′OH was carried out by careful control of KO*t*Bu stoichiometry (1.05 equiv) and addition of MeI using the conditions described by Panek^74^ to afford versatile azido sugar **110** in 79 % yield. At this point the approach could diverge to either the callipeltose A or B sugars.

**Scheme 23:**
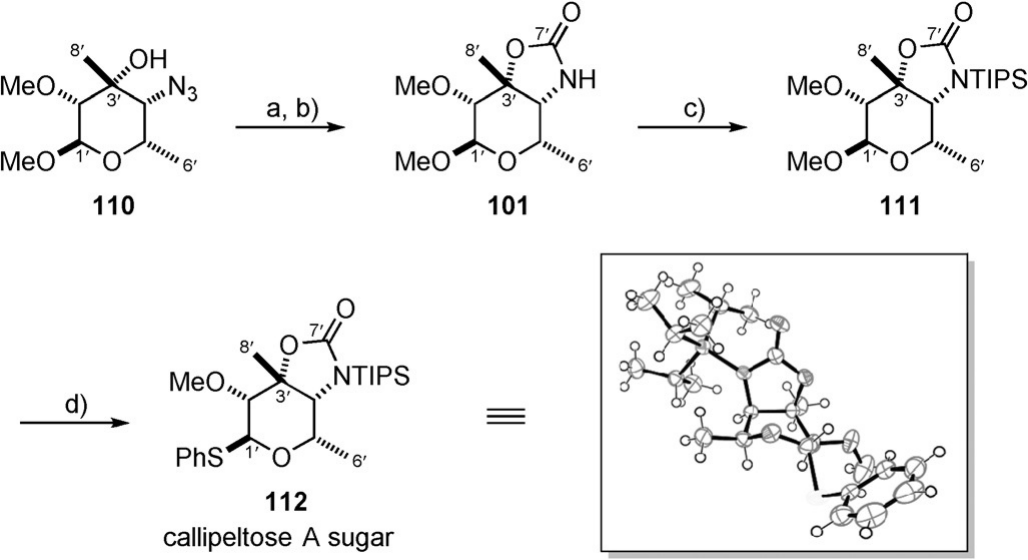
a) H_2_, Pd(OH)_2_/C (27 mol %), EtOAc, RT, 87 %; b) triphosgene, pyr, CH_2_Cl_2_, −78 °C→RT, 72 %; c) TIPSCl, 2,6-lutidine, CH_2_Cl_2_, RT, 97 %; d) PhSH, BF_3_⋅OEt_2_, CH_2_Cl_2_, 0 °C→RT, 80 %.

**b) Synthesis of the callipeltose A and B thioglycosides**: In order to synthesise the cyclic carbamate scaffold present in callipeltose A, the azide was reduced under hydrogenation conditions with Pearlman’s catalyst to form the corresponding primary amine. This was then reacted with triphosgene in pyridine to afford known bicyclic compound **101** in 72 % yield.^4^ Protection then provided callipeltose methoxyacetal **111**, ready for manipulation as an appropriate glycosyl donor (Scheme 23). Since previous reports had indicated that the corresponding trichloroacetimidate of callipeltose C was unstable to chromatography,^10^ we chose to convert each callipeltose sugar to its thioglycoside. Reaction of methoxyacetal **111** with PhSH and BF_3_**⋅**OEt_2_ resulted in thioglycoside **112** as a single anomer. The relative and absolute stereochemistry of this compound was confirmed by X-ray crystallography (Scheme 23).

**Scheme 24:**
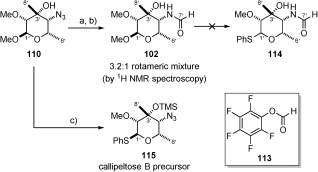
a) H_2_, Pd(OH)_2_/C (27 mol %), EtOAc, RT, 87 %; b) 113, CHCl_3_, RT, 75 % over 2 steps; c) TMSSPh, ZnI_2_, TBAI, 1,2-dichloroethane, 65 °C, 60 %.

In an identical fashion to callipeltose A, azide **110** was reduced to the primary amine and the formyl group then installed. Since we were concerned by the sensitivity of the methoxyacetal to acidic conditions, we sought a mild, acid-free procedure to install the requisite formyl group. Kisfaludy^75^ had previously shown that pentafluorophenyl formate (**113**) was a mild and selective reagent for the formylation of amines, with no reaction observed in the presence of alcohol functionality. Pleasingly, application of this reagent delivered callipeltose B methoxyacetal **102** in good yield (75 % over two steps) and as a 3.2:1 rotameric mixture (Scheme 24). This was in keeping with Minale’s original isolation paper,1b since it is mentioned that callipeltoside B exists as a mixture of ‘two inseparable conformers’. The resulting variable temperature ^1^H NMR spectroscopic studies conducted on callipeltose B methoxyacetal **102** indicated a very high barrier to interconversion, with the NMR spectroscopic signals coalescing between 393 and 413 K (see the Supporting Information).

With formylated methoxyacetal **102** in hand, we attempted to convert this compound to its corresponding thioglycoside (**114**) (Scheme 24). However, for unknown reasons, this could not be achieved (PhSH, BF_3_**⋅**OEt_2_ and TMSSPh, ZnI_2_, TBAI^76^), with re-isolation of starting material observed. As a result, we decided that azido sugar **115** would have to be converted to its thioglycoside and further manipulated to provide the formyl sugar following attachment to the callipeltoside aglycon (**4**). In contrast to formylated sugar **102**, azido precursor **115** could be easily converted into the thioglycoside by treatment with a combination of TMSSPh, ZnI_2_ and TBAI to once again provide a single anomer.^76^ In doing so, the C3’ hydroxyl was advantageously protected as its TMS-ether in readiness for its forthcoming attachment to the callipeltoside aglycon.

**Scheme 25:**
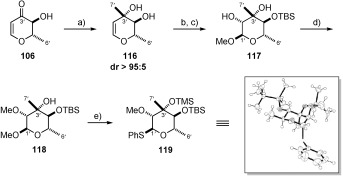
a) MeLi⋅LiBr, Et_2_O, −78 °C, 78 %; b) TBSOTf, 2,6-lutidine, DMF, 48 %; c) MMPP, NaHCO_3_, MeOH, 0 °C; d) MeI, Ag_2_O, DMF, RT, 80 % over 2 steps; e) TMSSPh, ZnI_2_, TBAI, 1,2-dichloroethane, 65 °C, 64 %. MMPP=magnesium monoperoxyphthalate.

**c) Synthesis of the callipeltose C thioglycoside**: As was the case for callipeltose A and B, work began from common pyranone **106**. Initial investigations focused on the formation of the C3’ stereocentre by means of a diastereoselective methyl addition to pyranone **106**. Early attempts at this transformation were conducted by the addition of MeLi**⋅**LiBr at −78 °C to substrates bearing a protected C4’ hydroxyl (pivolyl, not shown). This unfortunately resulted in the undesired stereochemistry, with only the C3’ (*R*)-diastereoisomer observed. After some investigation, we found that the selectivity could be completely reversed by leaving the C4’ hydroxyl group unprotected to afford diol **116** in good yield (78 %) and as a single diastereoisomer. As a result, we postulate that this excellent diastereocontrol is the result of a complex-induced proximity effect exerted by the neighbouring α-hydroxyl group (Scheme 25).^77^ Having set the C3’ stereocentre, our next challenge was to selectively protect the C4’ secondary alcohol in preference to the tertiary C3’ alcohol functionality.

Although a selective protection was ultimately not realised, the unwanted mono- or bis-silylated material could be easily separated from the required C4′-protected product and recycled to reclaim the original diol.^78^

Following protection of the C4′-hydroxyl, epoxidation with concomitant methanolysis provided product **117**. Methylation and thioglycoside formation using conditions analogous to that described for azido sugar **110** successfully delivered TBS-protected callipeltose C thioglycoside **119**. With sufficient quantities of the callipeltoside aglycon and all three callipeltoside sugars in hand efforts began to assemble all three natural products.

### Completion of callipeltoside A

**Scheme 26:**
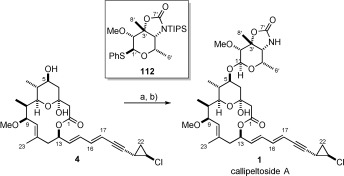
a) 4, 112, 4 Å MS, CH_2_Cl_2_, DTBMP, RT, then −15 °C, NIS, TfOH, −15 °C→RT; b) TBAF, THF, 83 % over 2 steps. DTBMP=2,6-di-*tert*-butyl-4-methylpyridine.

The callipeltoside aglycon was successfully coupled with thioglycoside donor **112** using the conditions described by Evans in his synthesis of callipeltoside A.5d Pleasingly, treatment of the TIPS-protected material with TBAF then afforded callipeltoside A in 83 % over two steps (Scheme 26). This synthetic material was found to match both the ^1^H and ^13^C NMR spectra provided for the natural isolate, whilst also exhibiting near-identical optical rotation ([*α*]
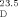
=−17.5 (*c*=0.33 in MeOH) compared to [*α*]
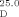
=−17.6 (*c*=0.04 in MeOH) for the natural product). Since the structure of callipeltoside A had been rigorously determined following the initial efforts of Trost,^4^ Evans^5^ and Paterson,^7^ it is assumed that the stereochemistry of the glycosyl linkage is that depicted in Scheme 26.

### Completion of callipeltoside C

**Scheme 27:**
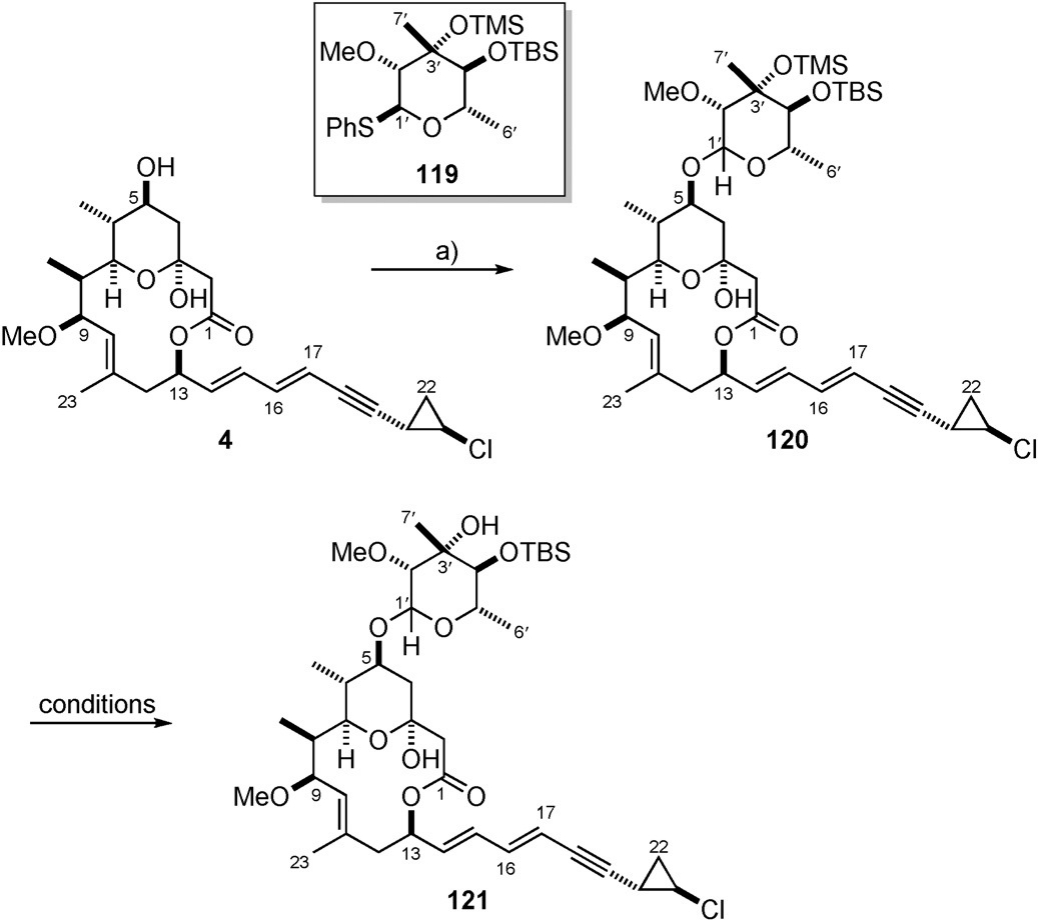
a) 4, 119, 4 Å MS, CH_2_Cl_2_, DTBMP, RT, then −15 °C, NIS, TfOH, −15 °C→RT, 80 %.

Glycosidation reaction between bis-protected callipeltose C sugar **119** and the callipeltoside aglycon (**4**) provided the desired product (**120**) in good yield (80 %; Scheme 27). However, deprotection of bis-protected callipeltoside C (**120**) proved problematic, with the TBS group being resilient to a number of different deprotection conditions (TBAF, HF**⋅**pyr, TASF,^79^ THF/HCO_2_H/H_2_O (6:3:1)]. Although the TMS protecting group could be readily cleaved under each set of conditions, in most cases the TBS-ether remained intact (**121**), resulting in either the re-isolation of TBS-protected material **120** or products whereby unidentified side reactions had occurred. While the number of deprotection conditions attempted was not extensive, the decision was made to revisit the preparation of callipeltose C, this time protecting the C4’ hydroxyl moiety as its TES-ether.

**Scheme 28:**
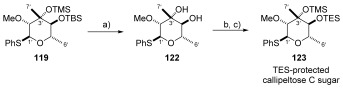
a) TBAF, THF, RT, 89 %; b) TESCl, pyr, DMAP, RT, 85 %; c) TMSOTf, 2,6-lutidine, CH_2_Cl_2_, −78 °C, 85 %.

In the absence of the callipeltoside aglycon, thioglycoside **119** could be easily deprotected using TBAF to provide diol **122** in 89 % yield. The secondary alcohol was then selectively protected as its TES-ether and the TMS-group installed in an efficient two-step process (Scheme 28).

**Scheme 29:**
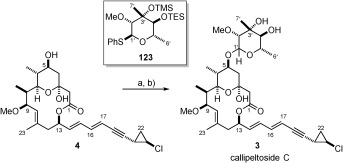
a) 4, 123, 4 Å MS, CH_2_Cl_2_, DTBMP, RT, then −15 °C, NIS, TfOH, −15 °C→RT; b) TASF, DMF, 40 °C, 57 % over 2 steps.

With TES-protected thioglycoside **123** in hand, the assembly of callipeltoside C was revisited. Once again the glycosidation reaction proceeded without incident, to afford the bis-protected substrate, ready for deprotection. On this occasion, treatment with TASF resulted in the removal of both protecting groups to provide callipeltoside C (**3**) (Scheme 29) in 57 % yield (2 steps).

This material was identical to both the ^1^H NMR spectra of the natural isolate and the synthetic material disclosed by MacMillan. However, the small amounts of natural product isolated meant that the ^13^C NMR spectra disclosed by Minale had been extrapolated from the HMQC spectrum, and so prevented further accurate ^13^C NMR spectroscopic comparison. In addition to this, no optical rotation was recorded for the natural product. Therefore, our structural assignment of callipeltoside C^80^ rests only on the comparison of the recorded ^1^H NMR spectra with the natural isolate.

### Analysis the glycosidic linkage of callipeltoside C

In order to complete the stereochemical assignment of callipeltoside C, we attempted to determine the configuration of the glycosidic linkage by analysis of the ^1^*J*_C-H_ coupling constant and NOESY data.

**a)** ^**1**^***J***_**C-H**_ **coupling constant**: Early empirical observations have shown that measurement of the ^1^*J*_C-H_ coupling constant derived from a HSQC (Heteronuclear Single Quantum Coherence) experiment without ^13^C decoupling provides an indication of whether the proton at the anomeric centre of a sugar moiety is axial or equatorial. This method has been shown to be a useful technique for the assignment of the configuration of the glycosidic linkage for a wide variety of carbohydrates. A value of ∼170 Hz typically suggests an equatorial proton at C1′H, whilst ∼160 Hz indicates an axial proton.^81^ Unfortunately, despite the good literature precedent for this technique, measurement of the ^1^*J*_C-H_ coupling constant in this system gave a value of ^1^*J*_C-H_=166.5 Hz, therefore providing an inconclusive result. Undeterred, we therefore chose to analyse the NOESY spectra of callipeltoside C in the hope that one conformation (assumed chair) of callipeltose C would be favoured over others.

**Figure 2:**
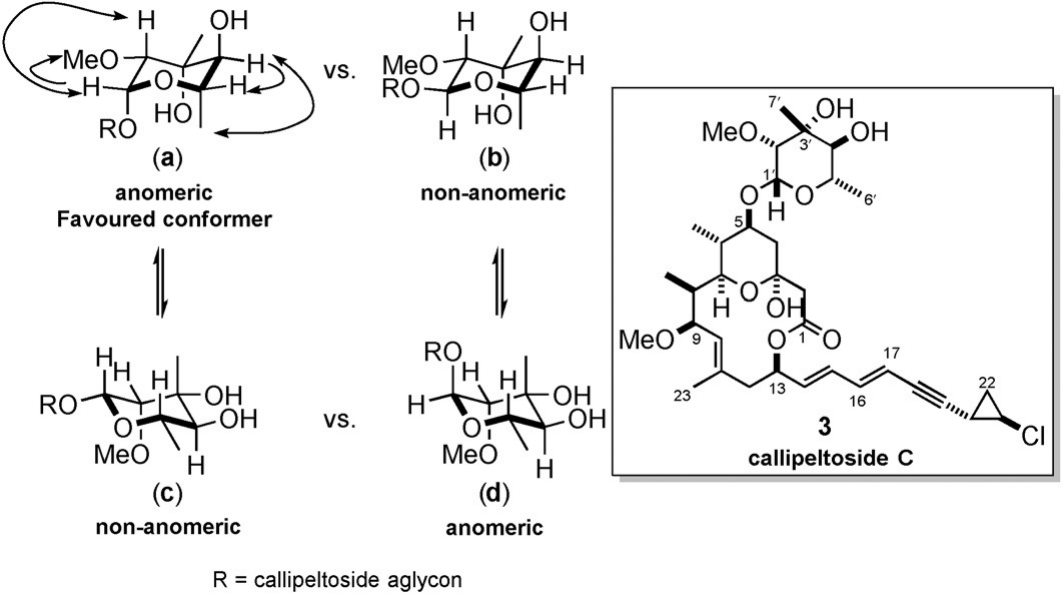
Key NOESY correlation observed for callipeltoside C.

**b)** **NOESY data**: As a result, we assessed all possible chair conformations (Figure 2a–d). Analysis of the averaged data revealed that structure (**a**) was the only conformer that accounted for all observed nOe interactions (see the Supporting Information). Therefore, based on this finding, we have tentatively assigned the glycosidic linkage of callipeltoside C to be that depicted in Figure 2. This is in full agreement with the stereochemical assignment by MacMillan.^10^

**Scheme 30:**
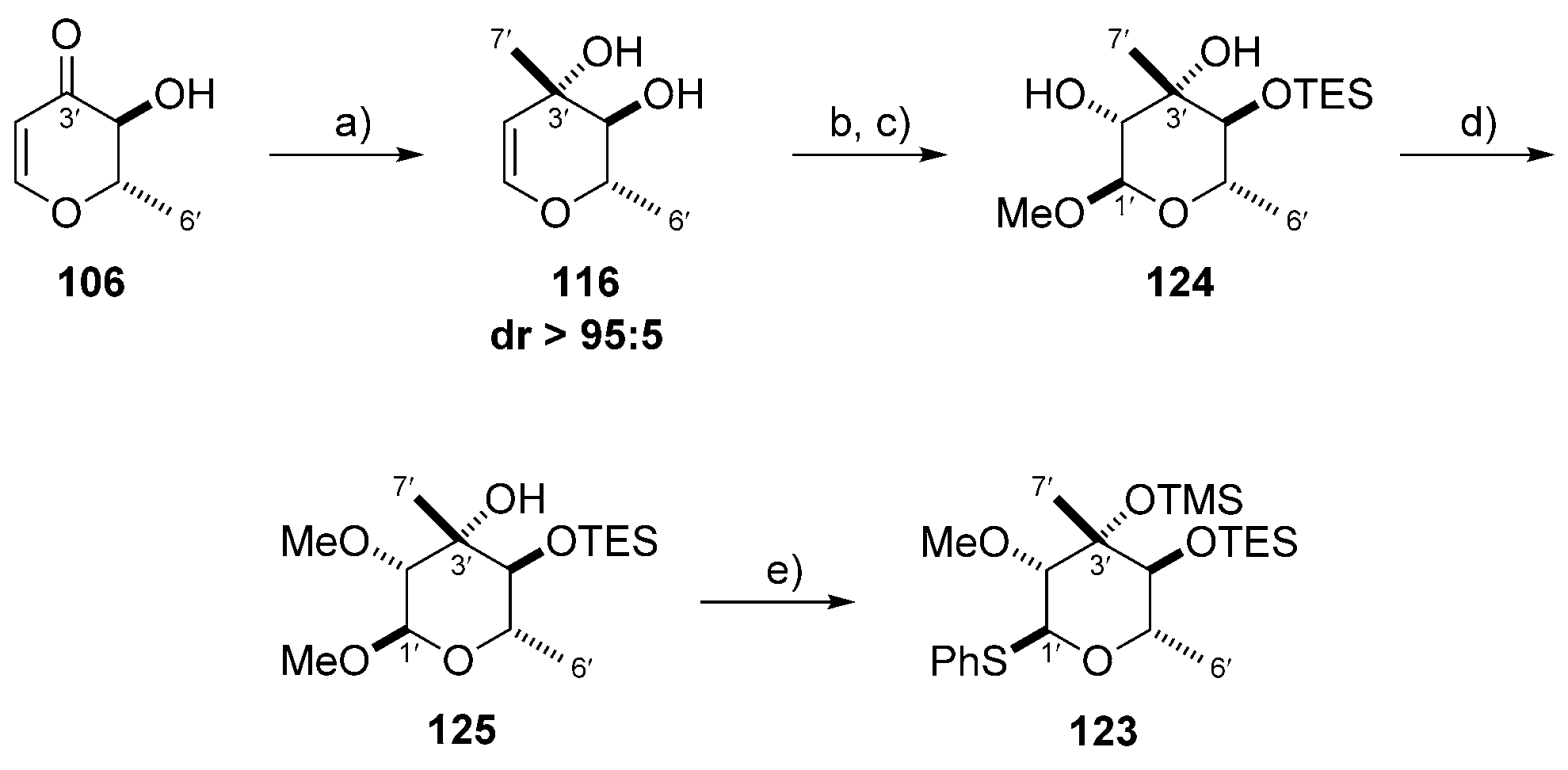
a) MeLi⋅LiBr, Et_2_O, −78 °C, 78 %; b) TESCl, pyr, DMAP, RT; c) *m*CPBA, NaHCO_3_, MeOH, 0 °C→RT, 45 % over 2 steps; d) KO*t*Bu, THF, 0 °C, then MeI, 0 °C, 81 %; e) TMSSPh, ZnI_2_, TBAI, 1,2-dichloroethane, 65 °C, 86 %.

After having completed the synthesis of callipeltoside C by means of a protecting group change (from TBS to TES), we decided it was appropriate to also complete the forward synthesis of TES-protected callipeltose C sugar **123** from common pyranone **106**. This was of course performed in an analogous manner to that described for the TBS-protected thioglycoside **119** (Scheme 25). However, to our delight, in this instance selective protection of the C4’ hydroxyl could be easily achieved using TESCl in combination with pyridine and DMAP (Scheme 30).

### Structural elucidation and completion of callipeltoside B

**Scheme 31:**
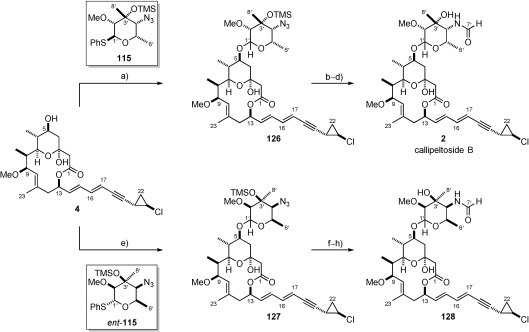
a) 4, 115, 4 Å MS, CH_2_Cl_2_, DTBMP, RT, then −15 °C, NIS, TfOH, −15 °C→RT, 56 %; b) 1,3-propanedithiol, Et_3_N, pyr/H_2_O (10:1), RT; c) 113, CHCl_3_, RT; d) TASF, DMF, 40 °C, 52 % over 3 steps, 4:1 rotameric mixture by ^1^H NMR spectroscopy; e) 4, *ent*-115, 4 Å MS, CH_2_Cl_2_, DTBMP, RT, then −15 °C, NIS, TfOH, −15 °C→RT, 41 %; f) 1,3-propanedithiol, Et_3_N, pyr/H_2_O (10:1), RT; g) 113, CHCl_3_, RT; h) TASF, DMF, 40 °C, 57 % over 3 steps, 4:1 rotameric mixture by ^1^H NMR spectroscopy.

With the syntheses of both callipeltosides A and C achieved, our attention switched to callipeltoside B in order to complete the series. As previously mentioned, there had been no prior total synthesis of callipeltoside B and therefore it was assumed that it also contained an L-configured sugar. As expected, attachment of **115** to the aglycon proceeded without incident, delivering **126** in moderate yield (56 %) (Scheme 31). At this point we faced the rather daunting prospect of having to reduce the azide moiety to the corresponding amine in the presence of multiple unsaturated functional groups. As anticipated, a hydrogenation-based approach was found to be unsuccessful, and therefore we expected that the Staudinger reaction^82^ would be an ideal method in order to achieve this transformation. However, in practice poor isolated yields and mixtures of unidentified products were obtained. Undeterred, we attempted the reduction using a combination of 1,3-propanedithiol and Et_3_N in aqueous pyridine.^83^ To our delight this afforded the requisite amine, which was immediately formylated using the aforementioned methodology developed by Kisfaludy (Scheme 24).^75^ Final treatment of this material with TASF to remove the TMS protecting group then gave callipeltoside B (**2**) as a mixture of conformers (4:1 by ^1^H NMR spectroscopy) *for the first time*. The ^1^H NMR spectrum of the synthetic material was found to be in complete agreement to that disclosed by Minale and his isolation team, and therefore provided evidence to validate our assumption that the attached sugar unit was also L-configured. However, since the structural determination of these molecules rests on only a single ^1^H NMR spectrum, we chose to synthesise the corresponding D-configured callipeltoside B sugar (*ent*-**115**, achieved in identical fashion to that shown in Scheme 22 and Scheme 24) and also attach this to the callipeltoside aglycon. Further elaboration would then allow direct comparison of ^1^H NMR spectra and provide additional evidence relating to the structure of callipeltoside B. This was straightforwardly achieved using the glycosidation, reduction, formylation and deprotection sequence described previously to deliver **128**. Comparison of the resulting ^1^H NMR spectrum revealed significant deviations from structure **2**, and therefore we confidently assign callipeltoside B to be that depicted in Scheme 31.

**Figure 3:**
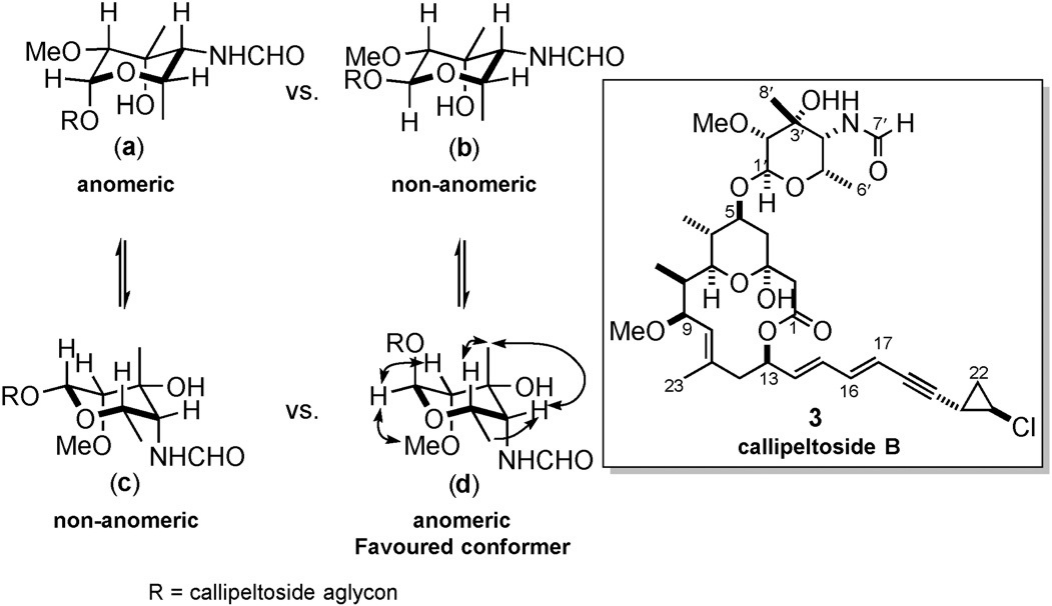
Key NOESY correlation observed for callipeltoside B.

Although it was clear that synthetic callipeltoside B had been synthesised, the configuration of the glycosidic linkage still needed to be ascertained. Our previous strategy of inferring the linkage configuration by NOESY experiments was again employed (Figure 3).

Once again, the NOESY spectra revealed that a single chair conformation (**d**) accounted for all observed correlations, suggesting that the glycosidic linkage is that depicted in Figure 3. It should be noted that whilst the configuration of the glycosidic linkage of callipeltoside B is identical to callipeltoside A, it is the opposite of callipeltoside C. Since exactly the same glycosidation conditions were used to attach each callipeltose sugar, the stereochemical course of the reaction must be influenced by the C4’ substituent present on the sugar moiety; however, additional studies to further study this effect have not been conducted.

## Conclusion

The synthesis of the entire callipeltoside family of natural products has been disclosed. At the beginning of our study we committed to the ambitious union of pyran aldehyde **5** and vinyl iodide **6** (**55** with a TBS protecting group) by a diastereoselective alkenylmetal addition with subsequent Yamaguchi macrocyclisation to complete the common callipeltoside aglycon. Attachment of callipeltoses A, B and C at a late stage then led to the synthesis of each natural product. Although we never deviated from these primary disconnections, the efficient synthesis of various key fragments provided a significant challenge and led us to re-evaluate our synthesis on the basis of selectivity, practicality and scale-up on a number of occasions. Still, perseverance, endeavour and determination have resulted in the successful completion of this research programme. We hope that this full account adequately highlights the trials and tribulations often encountered when designing, carrying out and completing a complex natural product synthesis.

## Experimental Section

A complete description of all experimental procedures and characterisation data relating to compounds described in this manuscript can be found in the Supporting Information. Additional data related to this publication is available at the University of Cambridge institutional data repository (https://www.repository.cam.ac.uk/handle/1810/248743).

## Supporting Information

As a service to our authors and readers, this journal provides supporting information supplied by the authors. Such materials are peer reviewed and may be re-organized for online delivery, but are not copy-edited or typeset. Technical support issues arising from supporting information (other than missing files) should be addressed to the authors.

miscellaneous_information
