# Supplementary material for: Callipeltosides A, B and C: Total Syntheses and Structural Confirmation
Source: Chemistry. 2015 Jul 31;21(38):13261–77. doi: 10.1002/chem.201501877 (PMC4583783; doi:10.1002/chem.201501877)
Supplement: Supplementary file 1 — miscellaneous_information [file chem0021-13261-sd1.pdf]

CHEMISTRY
A European Journal

Supporting Information

Callipeltosides A, B and C: Total Syntheses and Structural Confirmation

James R. Frost,*[a] Colin M. Pearson,[a] Thomas N. Snaddon,[a, b] Richard A. Booth,[a]
Richard M. Turner,[a] Johan Gold,[a] David M. Shaw,[a] Matthew J. Gaunt,[a] and Steven V. Ley*[a]

chem_201501877_sm_miscellaneous_information.pdf

Supporting Information

Table of Contents

Table of Contents	Page
I) General Experimental	2
II) Experimental Procedures	5
III) Selected NMR Spectra	145
IV) References	165

I) General Experimental

All experiments were performed in oven-dried glassware (anhydrous conditions) and under an argon atmosphere unless otherwise stated.

Solvents and reagents: Solvents were distilled under argon prior to use; CH_2Cl_2, MeOH, MeCN and PhMe from calcium hydride; Et_2O and THF from calcium hydride and $LiAlH_4$, with triphenyl methane indicator for THF. All solvents were dry reagent grade unless otherwise stated. All chemical reagents used were commercially available from Fisher and Sigma Aldrich in the highest available purity. Commercial *n*BuLi (in hexanes) and *t*BuLi were titrated prior to use using 2,6-di-*tert*-butyl-4-methylphenol in Et_2O with 1,10-phenanthroline as an indicator.

Chromatography: Thin layer chromatography (TLC) was performed on pre-coated glass-backed Merck Kieselgel 60 F_{254} plates with visualisation effected with ultra-violet irradiation (λ = 254 nm) and/or staining using potassium permanganate, vanillin or ninhydrin solutions. Flash column chromatography with Merck Kieselgel (230–400 mesh) silica gel performed according to the method employed by W. C. Still et al.[†] All solvents used for chromatographic purification were distilled prior to use with the exception of Et_2O and HPLC grade *n*-hexane, which were used as supplied.

Atom labeling: Labeling is in accord with the natural product numbering system and is indicated on the relevant diagram.

NMR spectroscopy: ^{1}H NMR spectra recorded on Bruker DPX-400 or Bruker Avance 500 (with dual cryoprobe) operating at 400 and 500 MHz respectively, with deuterated solvent acting as an internal deuterium lock. Data is reported in the following manner: chemical shift [in parts per million (ppm)] relative to tetramethylsilane (external standard), number of protons and assignment, chemical, multiplicity and coupling constant *J* (measured in Hz to the nearest 0.1 Hz). The multiplicity of a signal is indicated as: s-singlet, d-doublet, t-triplet, m-multiplet, br-

[†] W. C. Still, M. Kahn, M. J. Mitra, *J. Org. Chem.* **1978**, *43*, 2923.

broad, appar-apparent or combinations of these. ^{13}C NMR spectra recorded on the same DPX-400 or Bruker Avance 500 (with dual cryoprobe) operating at 100 and 126 Hz respectively with broadband proton decoupling and the deuterium solvent as an internal lock. ^{19}F NMR spectra were recorded on a Bruker Avance 400 (376 MHz) QNP Ultrashield spectrometer with broadband proton decoupling using the deuterated solvent as internal deuterium lock. Chemical shift data are given in parts per million relative to $CFCl_3$ (external standard).

Residual protic solvent also acted as an internal reference ($CDCl_3$; ^1H NMR = 7.26 ppm, ^{13}C = 77.1 ppm, $(CD_3)_2SO$; ^1H NMR 2.50 ppm, ^{13}C = 39.5 ppm, CD_3OD; ^1H NMR 4.88, 3.34 ppm, ^{13}C = 49.0 ppm; C_6D_6; ^1H NMR 7.16 ppm, ^{13}C = 128.06 ppm)

Structural assignments were made with the aid of DEPT 135, HMQC, HSQC, HMBC, COSY, NOESY and individual nOe experiments in the assignment of signals in ^1H and ^{13}C NMR spectra.

Infrared Spectroscopy: Spectra were recorded on a Perkin-Elmer Spectrum One FT-IR ATR (Attenuated Total Reflectance) Spectrometer as a thin film deposited on the ATR. Only selected characteristic peaks are recorded.

Optical rotations: Measured on Perkin Elmer 343 polarimeter and $[\alpha]_D$ values quoted in $10^{-1}degcm^2g^{-1}$ with concentration (*c*) quoted in $g(100\ mL)^{-1}$.

Mass Spectroscopy (EI, ESI): High resolution mass spectra (HRMS) recorded on Waters Micromass LCT spectrometer using time of flight with positive electrospray ionisation (ESI^+) or negative electrospray ionisation (ESI^-), an ABI/MDS Sciex Q-STAR Pulsar with ESI^+, or a Bruker BioApex II 4.7e FTICR utilising either ESI^+ or a positive electron ionisation (EI^+) source equipped with a direct insertion probe. The mass reported is that containing the most abundant isotopes (^{35}Cl and ^{79}Br). This was performed at the Department of Chemistry, University of Cambridge, Lensfield Road, Cambridge, with each value obtained within 5 ppm of the calculated mass.

Melting points: Determined using an SRS Optimelt MPA 100 automated melting point system, with range quoted to the nearest whole number.

X-ray crystallography: Recorded by Dr John E. Davies at the Department of Chemistry, University of Cambridge, Lensfield Road, Cambridge using a Nonius Kappa CCD detector. Crystal structure images presented in this thesis were produced using ORTEP-3 for windows.[†] All crystallographic data has been deposited on the Cambridge Structural Database compiled by the Cambridge Crystallographic Data Centre (CCDC). The corresponding CCDC number for each compound is listed by the relevant structure.

Elemental Analysis: Performed by Alan Dickerson at the Microanalytical Laboratories, Department of Chemistry, University of Cambridge. All reported values are within ±0.5% of the calculated value.

Naming of compounds: Carried out using the computer programme ACD/Name. This software generates the systematic name of chemical structures according to the guidelines specified by the International Union of Pure and Applied Chemistry (IUPAC). As a result the numbering system used in these names does not follow that of the natural product. In order to allow for the straightforward comparison of data, the NMR assignments given follow the natural product numbering system which is shown on the chemical structure.

Structures not shown in the manuscript that have been described in the Supporting Information are numbered with the form S#.

[†] L. J. Farrugia, *J. Appl. Cryst*. **1997**, *30*, 565.

II) Experimental Procedures

(2*S*)-Methanesulfonic acid-1-methyl-prop-2-ynyl ester 9[1]

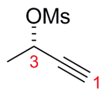

To a stirring solution of (2*S*)-3-butyn-2-ol (1.0 g, 14.3 mmol) in CH_2Cl_2 (25 mL) at –78 °C was added Et_3N (8.0 mL, 57.1 mmol) followed by methanesulfonyl chloride (3.3 mL, 42.9 mmol) over 30 mins, resulting in the formation of a yellow precipitate. The mixture was stirred for 1 h at –78 °C, quenched by the addition of sat. aq. $NaHCO_3$ (25 mL), and the layers separated. The aqueous layer was further extracted with CH_2Cl_2 (2 × 25 mL), and the combined organic layers dried ($MgSO_4$) and concentrated *in vacuo*. Purification by column chromatography [SiO_2, petroleum ether (40:60): Et_2O, 8:2] gave the title compound **9** as a colourless oil (1.97 g, 93%).

***R*$_f$** = 0.05 [petroleum ether (40:60): Et_2O, 9:1]; $[\alpha]_D^{25}$ = –106.4 (*c* = 0.42, $CHCl_3$); **IR** (film) ν_{max}/cm^{-1} 3281, 3031, 2999, 2943, 1353, 1332, 1172, 1123, 1089, 1016; **^{1}H NMR** ($CDCl_3$, 400 MHz) δ = 5.27 (1H, qd, *J* = 6.7, 2.1 Hz, C3H), 3.10 (3H, s, SO_2CH_3), 2.70 (1H, d, *J* = 2.1 Hz, C1H), 1.65 (3H, d, *J* = 6.7 Hz, C4H_3); **^{13}C NMR** ($CDCl_3$, 100 MHz) δ = 80.2 (C2), 76.3 (C1H), 67.5 (C3H), 39.2 (SO_2CH_3), 22.5 (C4H_3); **HRMS** (ESI) found 171.0095 ($[M+Na]^+$ $C_5H_8O_3SNa$ requires 171.0086). All spectroscopic data in agreement with that previously published.[1]

(2*R*,3*R*,4*R*)-1-(*tert*-Butyl-dimethyl-silanyloxy)-2,4-dimethyl-hex-5-yn-3-ol 10a

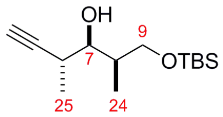

To a stirring solution of $Pd(OAc)_2$ (440 mg, 1.96 mmol) in THF (250 mL) at –78 °C was added PPh_3 (520 mg, 1.98 mmol). The resulting yellow solution was stirred for 10 mins at which point **9** (4.77 g, 30.9 mmol) and **8a** (4.46 g, 20.1 mmol)[2] were added dropwise sequentially. After

stirring for 5 mins $ZnEt_2$ (1 M in hexanes, 66.0 mL, 66.0 mmol) was added to the mixture over a period of 1 h. The reaction mixture was stirred for 30 mins at –78 °C before being slowly warmed to –20 °C and maintained at this temperature for a further 24 h. The reaction was quenched by the addition of sat. aq. NH_4Cl (80 mL), diluted with Et_2O (80 mL) and warmed to RT. The layers were separated and the aqueous layer further extracted with Et_2O (3 × 80 mL), and the combined organic layers dried ($MgSO_4$), and concentrated *in vacuo*. Purification by column chromatography [SiO_2, petroleum ether (40–60):Et_2O:Et_3N, 90:9:1→80:19:1] gave the title compound **10a** (1.41 g, 70%).as a pale yellow oil as the major diastereomer (dr = 94:6).

$\boldsymbol{R_f}$ = 0.10 [petroleum ether (40–60):Et_2O, 9:1]; $[\alpha]_D^{25.0}$ = +6.3 (*c* = 0.34, $CHCl_3$); **IR** (film) ν_{max}/cm^{-1} 3600, 3312, 2956, 2930, 2858, 1472, 1463, 1388, 1361, 1251, 1086; **^{1}H NMR** ($CDCl_3$, 400 MHz) δ = 3.67 (2H, d, *J* = 6.6 Hz, CH_AH_BOSi), 3.59–3.56 (1H, m, C7H), 2.70–2.65 (1H, m, C6H), 2.63 (1H, d, *J* = 4.5 Hz, C7HO*H*), 2.13 (1H, d, *J* = 2.3 Hz, C4H), 1.83–1.75 (1H, m, C8H), 1.20 (3H, d, *J* = 7.0 Hz, C25H_3), 0.96 (3H, d, *J* = 7.0 Hz, C24H_3), 0.90 (9H, s, C(CH_3)$_3$ of *t*Bu), 0.06 (6H, s, Si(CH_3)$_2$); **^{13}C NMR** ($CDCl_3$, 100 MHz) δ = 86.4 (C5), 76.2 (C7H), 70.2 (C4H), 67.2 (C9H_2), 37.5 (C8H), 30.5 (C6H), 25.8 (3C, C(*C*H_3)$_3$ of *t*Bu), 18.2 (*C*(CH_3)$_3$ of *t*Bu), 17.6 (C25H_3) 10.3 (C24H_3), –5.5 (2C, Si(*C*H_3)$_2$); **HRMS** (+ESI) Found $[M+H]^+$ = 257.1940; $C_{14}H_{29}O_2Si$ requires 257.1937, Δ 1.17 ppm.

***tert*-Butyl-((2*R*,3*R*,4*R*)-3-(4-methoxy-benzyloxy)-2,4-dimethyl-hex-5-ynyloxy)-dimethylsilane S1**

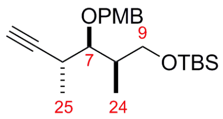

To a solution of **10a** (932 mg, 4.01 mmol) in DMF (6 mL) and THF (6 mL) at 0 °C was added NaH (60% in mineral oil, 193 mg, 4.83 mmol). The reaction mixture was stirred for 45 mins at RT. Then PMBBr (705 µL, 4.82 mmol) was added dropwise and the reaction stirred for 2 h at RT. The reaction was quenched using sat. aq. NH_4Cl (20 mL) and diluted with Et_2O (20 mL). The aqueous layer was separated and extracted with Et_2O (2 × 20 mL). The combined organics were washed with sat. aq. LiCl (50 mL), dried ($MgSO_4$) and concentrated *in vacuo* to give a pale

yellow oil. Purification by column chromatography [SiO_2, petroleum ether (40–60):Et_2O, 95:5→9:1] gave the title compound **S1** (1.16 g, 82%) as a colourless oil.

***R*f** = 0.46 [petroleum ether (40–60):Et_2O, 9:1]; $[\alpha]_D^{25.0}$ = –23.8 (*c* = 0.51, $CHCl_3$); **IR** (film) ν_{max}/cm^{-1} 3310, 2954, 2929, 2857, 1613, 1514, 1463, 1247, 1083, 1036; **^{1}H NMR** ($CDCl_3$, 400 MHz) δ = 7.30 (2H, d, *J* = 8.6 Hz, Ar), 6.86 (2H, d, *J* = 8.6 Hz, Ar), 4.76 (1H, d, *J* = 10.8 Hz, CH_AH_BAr), 4.54 (1H, d, *J* = 10.8 Hz, CH_AH_BAr), 3.80 (3H, s, OCH_3), 3.58–3.42 (3H, m, C7H and C9H_AH_B), 2.81–2.72 (1H, m, C6H), 2.07 (1H, d, *J* = 2.4 Hz, C4H), 1.98–1.92 (1H, m, C8H), 1.20 (3H, d, *J* = 7.0 Hz, C25H_3), 0.94–0.88 (12H, m, C24H_3 and $C(CH_3)_3$ of *t*Bu), 0.04 (6H, s, $Si(CH_3)_2$); **^{13}C NMR** ($CDCl_3$, 100 MHz) δ = 159.1 (Ar), 131.3 (2C, Ar), 129.4 (2C, Ar), 113.6 (Ar), 87.5 (C5), 81.5 (C7H), 74.3 (OCH_2Ar), 69.2 (C4H), 65.5 (C9H_2), 55.2 (OCH_3), 38.3 (C8H), 29.5 (C6H), 25.9 (3C, $C(CH_3)_3$ of *t*Bu), 18.2 ($C(CH_3)_3$ of *t*Bu), 17.9 (C25H_3), 11.2 (C24H_3), –5.4 (2C, $Si(CH_3)_2$); **HRMS** (+ESI) Found $[M+H]^+$ = 377.2498; $C_{22}H_{37}O_3Si$ requires 377.2512, Δ 3.71 ppm.

(4*R*,5*R*,6*R*)-7-(*tert*-Butyl-dimethyl-silanyloxy)-5-(4-methoxy-benzyloxy)-4,6-dimethylhept-2-ynoic acid ethyl ester S2

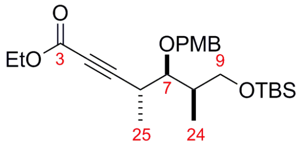

To a solution of **S1** (3.54 g, 9.40 mmol) in THF (145 mL) at –78 °C was added *n*BuLi (1.5 M in hexanes, 6.90 mL, 10.3 mmol) dropwise. The reaction was stirred for 1 h, after which ethyl chloroformate (1.35 mL, 14.1 mmol) was added and the reaction allowed to warm to RT over 18 h. The reaction was quenched by the addition of sat. aq. NH_4Cl (140 mL) and diluted with Et_2O (150 mL). The aqueous layer was separated and extracted with Et_2O (2 × 120 mL). The combined organic layers were dried ($MgSO_4$), filtered and concentrated *in vacuo* to afford a pale yellow oil. Purification by column chromatography [SiO_2, petroleum ether (40–60):Et_2O, 98:2→9:1] gave the title compound **S2** (4.02 g, 95%) as a pale yellow oil.

R_f = 0.18 [petroleum ether (40–60):Et_2O, 9:1]; $[\alpha]_D^{25.0}$ = +3.2 (*c* = 0.575, $CHCl_3$); **IR** (film) ν_{max}/cm^{-1} 2955, 2930, 2858, 2239, 1709, 1613, 1514, 1463, 1244, 1086, 1035; **^{1}H NMR** ($CDCl_3$, 400 MHz) δ = 7.32 (2H, d, *J* = 8.6 Hz, Ar), 6.87 (2H, d, *J* = 8.6 Hz, Ar), 4.75 (1H, d, *J* = 10.5 Hz, OCH_AH_BAr), 4.54 (1H, d, *J* = 10.5 Hz, OCH_AH_BAr), 4.22 (2H, q, *J* = 7.1 Hz, OCH_2CH_3), 3.80 (3H, s, OCH_3), 3.61–3.48 (3H, m, C7H and C9H_AH_B), 2.89 (1H, quint, *J* = 7.2 Hz, C6H), 1.93–1.89 (1H, m, C8H), 1.30 (3H, t, *J* = 7.1 Hz, OCH_2CH_3), 1.22 (3H, d, *J* = 7.1 Hz, C25H_3), 0.91 (9H, s, $C(CH_3)_3$ of *t*Bu), 0.87 (3H, d, *J* = 6.9 Hz, C24H_3), 0.05 (6H, s, $Si(CH_3)_2$); **^{13}C NMR** ($CDCl_3$, 100 MHz) δ = 159.2 (Ar), 154.2 (C3), 130.9 (2C, Ar), 129.6 (2C, Ar), 113.7 (Ar), 92.0 (C5), 80.9 (C7H), 74.5 (OCH_2Ar), 73.7 (C4), 65.3 (C9H_2), 61.6 (OCH_2CH_3), 55.2 (OCH_3), 38.2 (C8H), 29.9 (C6H), 25.9 (3C, $C(CH_3)_3$ of *t*Bu), 18.2 ($C(CH_3)_3$ of *t*Bu), 16.8 (C25H_3), 14.0 (OCH_2CH_3), 10.7 (C24H_3), –5.4 (2C, $Si(CH_3)_2$); **HRMS** (+ESI) Found $[M+H]^+$ = 449.2739; $C_{25}H_{41}O_5Si$ requires 449.2723, Δ 3.56 ppm.

((7*R*,9*S*,10*S*)-9-((*R*)-2-(*tert*-Butyl-dimethyl-silanyloxy)-1-methyl-ethyl)-7-hydroxy-10-methyl-8-oxa-1,4-dithia-spiro[4.5]dec-7-yl)-acetic acid methyl ester 13a

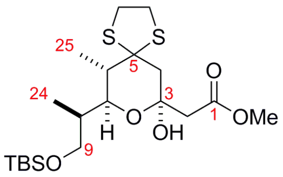

The first step of the following procedure was conducted in 6 parallel batches that were combined for purification.

To a solution of *i*Pr_2NH (690 µL, 4.90 mmol) in THF (9 mL) at –78 °C was added *n*BuLi (1.5 M in hexanes, 3.26 mL, 4.90 mmol) dropwise. The solution was allowed to warm to 0 °C, stirred for 10 min and then re-cooled to –78 °C. Methyl acetate (410 µL, 5.11 mmol) was added dropwise and the reaction stirred for 1 h. This solution of the preformed anion was then cannulated quickly to a solution of **S2** (1.00 g, 2.22 mmol) in THF (9 mL) at –78 °C. The reaction was then warmed to RT over 18 h. TLC [petroleum ether (40–60):Et_2O, 7:3] showed conversion to a major product. The reaction was quenched with sat. aq. NH_4Cl (10 mL), the aqueous layer separated and extracted with Et_2O (3 × 15 mL). The combined organic layers of 6

batches were dried ($MgSO_4$) and concentrated *in vacuo* to give **11a** (6.35 g, 6 batches, 13.32 mmol, assumed quant.) as a pale yellow oil which was used directly in the next step.

To a solution of **11a** (13.32 mmol) in MeOH (150 mL) and CH_2Cl_2 (150 mL) at –10 °C was added 1,2-ethanedithiol (1.25 mL, 14.9 mmol), followed by NaOMe (795 mg, 13.98 mmol). The reaction was allowed to warm to RT and stirred for 18 h. The reaction was quenched by the addition of sat. aq. NH_4Cl (250 mL). The aqueous layer was extracted with Et_2O (3 × 200 mL). The organic phases were dried ($MgSO_4$) and concentrated *in vacuo*.

The resulting pale yellow oil (**12a**) was dissolved in CH_2Cl_2 (800 mL) and pH 7 phosphate buffer (80 mL) at RT. DDQ (4.54 g, 20.0 mmol) was then added and the reaction mixture stirred for 3 h. The reaction was quenched by the addition of sat. aq. NH_4Cl (500 mL) and the phases separated. The aqueous layer was extracted with CH_2Cl_2 (2 × 250 mL) and Et_2O (150 mL). The combined organic layers were washed with H_2O (400 mL), dried ($MgSO_4$) and concentrated *in vacuo* to give a dark brown oil. The crude product was dissolved in CH_2Cl_2 and stirred with aminoethyl polystyrene quadra gel (6 g) overnight. Filtration, evaporation and purification by column chromatography [SiO_2, petroleum ether (40–60):Et_2O, 8:2→7:3] gave the title compound **13a** (4.12 g, 68% over 3 steps) as a white crystalline solid.

$\boldsymbol{R_f}$ = 0.32 [petroleum ether (40–60):Et_2O, 7:3]; **m.p.** = 45–46 °C; $[\alpha]_D^{25.0}$ = –15.9 (*c* = 0.50, $CHCl_3$); **IR** (film) ν_{max}/cm^{-1} 3508, 2957, 2928, 2890, 2854, 1723, 1437, 1410, 1400, 1253, 1162, 1143, 1074, 1002; **^{1}H NMR** ($CDCl_3$, 400 MHz) δ = 4.89 (1H, d, *J* = 1.4 Hz, OH), 3.86 (1H, dd, *J* = 10.1, 2.0 Hz, C7H), 3.71 (3H, s, $C1O_2CH_3$), 3.53 (1H, dd, *J* = 9.6, 6.9 Hz, C9H_AH_B), 3.37 (1H, dd, *J* = 9.5, 7.4 Hz, C9H_AH_B), 3.30–3.20 (4H, m, SCH_2CH_2S), 2.60 (1H, d, *J* = 14.0 Hz, C4H_AH_B), 2.57 (2H, s, C2H_AH_B), 2.32 (1H, dd, *J* = 14.0, 1.4 Hz, C4H_AH_B), 1.95–1.85 (2H, m, C6H and C8H), 1.09 (3H, d, *J* = 6.6 Hz, C25H_3), 0.92 (9H, s, C(CH_3)$_3$ of *t*Bu), 0.81 (3H, d, *J* = 6.9 Hz, C24H_3), 0.06 (3H, s, Si(CH_3)$_2$), 0.05 (3H, s, Si(CH_3)$_2$); **^{13}C NMR** ($CDCl_3$, 100 MHz) δ = 171.9 (*C*1O_2CH_3), 95.4 (C3), 73.6 (C7H), 70.5 (C5), 66.4 (C9H_2), 52.1 (C1O_2*C*H_3), 52.0 (C4H_2), 45.7 (C2H_2), 43.4 (C6H), 41.4 (S*C*H_2CH_2S), 39.1 (SCH_2*C*H_2S), 37.4 (C8H), 26.4 (3C, C(*C*H_3)$_3$ of *t*Bu), 18.7 (*C*(CH_3)$_3$ of *t*Bu), 12.4 (C25H_3), 9.4 (C24H_3), –5.0 (2C, Si(*C*H_3)$_2$); **HRMS** (+ESI) Found $[M+Na]^+$ = 473.1836; $C_{20}H_{38}O_5SiS_2Na$ requires 473.1828, Δ 1.69 ppm.

((7*R*,9*S*,10*S*)-9-((*R*)-2-Hydroxy-1-methyl-ethyl)-7-methoxy-10-methyl-8-oxa-1,4-dithiaspiro[4.5]dec-7-yl)-acetic acid methyl ester S3

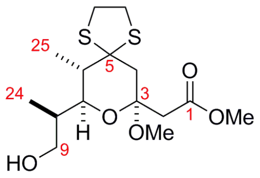

To a solution of **13a** (2.88 g, 6.40 mmol) in MeOH (60 mL) at RT was added trimethylorthoformate (10.5 mL, 96.0 mmol) and pyridinium *p*-toluenesulphonate (3.22 g, 12.8 mmol) sequentially. The reaction was stirred for 18 h at RT after which it was quenched by the addition of sat. aq. NH_4Cl (250 mL). The resulting mixture was extracted with Et_2O (3 × 200 mL) and the combined organic layers dried ($MgSO_4$) and concentrated *in vacuo* to give a white solid. Purification by column chromatography [SiO_2, petroleum ether (40–60):Et_2O, 1:1] gave the title compound **S3** (2.06 g, 92%) as white needles.

R_f = 0.21 [Et_2O:petroleum ether (40–60), 7:3]; **m.p.** = 90–91 °C; $[\alpha]_D^{25.0}$ = –45.7 (*c* = 0.67, $CHCl_3$); **IR** (film) ν_{max}/cm^{-1} 3311, 2967, 2940, 2920, 2891, 1737, 1437, 1354, 1330, 1266, 1222, 1163, 1135, 1103, 1046, 1025; **^{1}H NMR** ($CDCl_3$, 400 MHz) δ = 3.78–3.71 (2H, m, C7H and C9H_AH_B), 3.69 (3H, s, C1O_2CH_3), 3.70–3.67 (1H, m, C9H_AH_B), 3.28 (3H, s, C3OCH_3), 3.28–3.16 (4H, m, SCH_2CH_2S), 2.69–2.66 (3H, m, C2H_AH_B and C4H_AH_B), 2.56 (1H, d, *J* = 13.5 Hz, C4H_AH_B), 2.17 (1H, bs, CH_2OH), 1.98–1.90 (1H, m, C8H), 1.88–1.80 (1H, m, C6H), 1.07 (3H, d, *J* = 6.6 Hz, C25H_3), 0.93 (3H, d, *J* = 7.0 Hz, C24H_3); **^{13}C NMR** ($CDCl_3$, 100 MHz) δ = 163.9 (*C*1O_2CH_3), 98.1 (C3), 76.7 (C7H), 69.3 (C5), 67.1 (C9H_2), 51.8 (C1O_2*C*H_3), 50.5 (C2H_2), 47.8 (C3O*C*H_3), 43.4 (C6H), 41.7 (C4H_2), 41.7 (S*C*H_2CH_2S), 38.6 (SCH_2*C*H_2S), 35.9 (C8H), 11.9 (C25H_3), 8.8 (C24H_3); **HRMS** (+ESI) Found $[M+Na]^+$ = 373.1122; $C_{15}H_{26}O_5S_2Na$ requires 373.1119, Δ 0.80 ppm. **Elemental Analysis** found C, 51.41; H, 7.37. $C_{15}H_{26}O_5S_2$ requires C, 51.40; H, 7.48%.

((2*S*,5*S*,6*S*)-6-((*R*)-2-Hydroxy-1-methyl-ethyl)-2-methoxy-5-methyl-4-oxo-tetrahydropyran-2-yl)-acetic acid methyl ester 14a

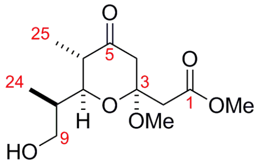

To a solution of **S3** (1.06 g, 3.04 mmol) in MeCN (50 mL) and H_2O (6.7 mL) at 0 °C was added a freshly prepared solution of BTI (0.067 M in MeCN, 90.0 mL, 6.03 mmol) dropwise. The reaction mixture was stirred at 0 °C for 2 h after which it was quenched by the addition of a mixture of sat. aq. Na_2SO_3 (50 mL) and sat. aq. $NaHCO_3$ (50 mL) (1:1). The reaction was diluted with EtOAc (150 mL) and the phases separated. The aqueous layer was extracted with EtOAc (2 × 80 mL) and the combined organic layers dried ($MgSO_4$) and concentrated *in vacuo.* Purification by column chromatography [SiO_2, Et_2O:petroleum ether (40–60), 7:3] gave the title compound **14a** (674 mg, 86%) as a pale yellow oil.

***R*f** = 0.20 [Et_2O:petroleum ether (40–60), 7:3]; $[\alpha]_D^{25.0}$ = –96.3 (*c* = 0.50, $CHCl_3$); **IR** (film) ν_{max}/cm^{-1} 3426, 2971, 2883, 1717, 1439, 1321, 1245, 1195, 1166, 1139, 1099, 1048, 1000; **^{1}H NMR** ($CDCl_3$, 400 MHz) δ = 3.81 (1H, dd, *J* = 10.6, 2.1 Hz, C7H), 3.76–3.72 (2H, m, C9H_AH_B), 3.72 (3H, s, C1O_2CH_3), 3.26 (3H, s, C3OCH_3), 2.94 (1H, d, *J* = 14.0 Hz, C4H_AH_B), 2.88 (1H, d, *J* = 13.8 Hz, C2H_AH_B), 2.64 (1H, d, *J* = 14.0 Hz, C4H_AH_B), 2.63 (1H, d, *J* = 13.8 Hz, C2H_AH_B), 2.49–2.41 (1H, m, C6H), 1.98–1.80 (1H, m, C8H), 1.71 (1H, bs, CH_2OH), 1.01 (3H, d, *J* = 7.1 Hz, C24H_3), 0.99 (3H, d, *J* = 6.7 Hz, C25H_3); **^{13}C NMR** ($CDCl_3$, 100 MHz) δ = 206.2 (C5O), 169.2 (*C*1O_2CH_3), 101.1 (C3), 76.6 (C7H), 66.3 (C9H_2), 52.0 (C1O_2*C*H_3), 49.5 (C4H_2), 48.4 (C3O*C*H_3), 45.7 (C6H), 41.1 (C2H_2), 36.2 (C8H), 8.7 (C25H_3), 8.6 (C24H_3); **HRMS** (+ESI) Found $[M+Na]^+$ = 297.1319; $C_{13}H_{22}O_6Na$ requires 297.1314, Δ 1.68 ppm.

((5*S*,6*S*)-6-((*R*)-2-(*tert*-Butyl-dimethyl-silanyloxy)-1-methyl-ethyl)-5-methyl-4-oxo-5,6-dihydro-4*H*-pyran-2-yl)-acetic acid methyl ester 17a

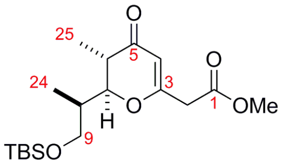

To a solution of **14a** (1.19 g, 4.34 mmol) in CH_2Cl_2 (110 mL) at –10 °C was added TfOH (460 µL, 5.21 mmol) dropwise. After approximately 1 min the reaction was quenched by the addition of sat. aq. $NaHCO_3$ (30 mL) and extracted with CH_2Cl_2 (2 × 50 mL) and Et_2O (2 × 50 mL). The combined organic extracts were dried ($MgSO_4$) and concentrated *in vacuo* to give a pale yellow oil. The crude product was then re-dissolved in CH_2Cl_2 (115 mL) and cooled to 0 °C. To this was added imidazole (740 mg, 10.85 mmol) followed by TBSCl (850 mg, 5.64 mmol) portionwise and the reaction mixture stirred at RT for 2 h. The reaction was quenched by the addition of sat. aq. NH_4Cl (100 mL) and the phases separated. The aqueous layer was extracted with Et_2O (2 × 80 mL) and the combined organic layers dried ($MgSO_4$) and concentrated *in vacuo* to give a yellow oil. Purification by column chromatography [SiO_2, petroleum ether (40–60):Et_2O, 7:3→6:4] gave the title compound **17a** (1.27 g, 83% over 2 steps) as a pale yellow oil.

***R*$_f$** = 0.29 [petroleum ether (40–60):Et_2O, 6:4]; $[\alpha]_D^{25.0}$ = –115.0 (*c* = 0.70, $CHCl_3$); **IR** (film) ν_{max}/cm^{-1} 2954, 2930, 2883, 2857, 2336, 1747, 1674, 1622, 1461, 1398, 1343, 1252, 1200, 1151, 1084, 1006; **^{1}H NMR** ($CDCl_3$, 400 MHz) δ = 5.39 (1H, s, C4H), 4.31 (1H, dd, *J* = 12.8, 2.3 Hz, C7H), 3.73 (3H, s, $C1O_2CH_3$), 3.66–3.58 (1H, m, C9H_AH_B), 3.54 (1H, dd, *J* = 9.8, 5.9 Hz, C9H_AH_B), 3.25 (2H, d, *J* = 1.8 Hz, C2H_AH_B), 2.56–2.48 (1H, m, C6H), 2.04–1.96 (1H, m, C8H), 1.08 (3H, d, *J* = 7.0 Hz, C25H_3), 0.92 (3H, d, *J* = 6.7 Hz, C24H_3), 0.88 (9H, s, C(CH_3)$_3$ of *t*Bu), 0.05 (3H, s, Si(CH_3)$_2$), 0.04 (3H, s, Si(CH_3)$_2$); **^{13}C NMR** ($CDCl_3$, 100 MHz) δ = 195.3 (C5O), 168.1 (*C*1O_2CH_3), 167.8 (C3), 105.3 (C4H), 83.2 (C7H), 64.3 (C9H_2), 52.4 (C1O_2*C*H_3), 40.4 (C2H_2), 40.2 (C6H), 36.9 (C8H), 25.8 (3C, C(*C*H_3)$_3$ of *t*Bu), 18.2 (*C*(CH_3)$_3$ of *t*Bu), 9.8 (C25H_3), 9.5 (C24H_3), –5.5 (2C, Si(*C*H_3)$_2$); **HRMS** (+ESI) Found $[M+H]^+$ = 357.2106; $C_{18}H_{33}O_5Si$ requires 357.2097, Δ 2.52 ppm.

((4*R*,5*S*,6*S*)-4-(*tert*-Butyl-dimethyl-silanyloxy)-6-((*R*)-2-(*tert*-butyl-dimethyl-silanyloxy)-1-methyl-ethyl)-5-methyl-5,6-dihydro-4*H*-pyran-2-yl)-acetic acid methyl ester 19a

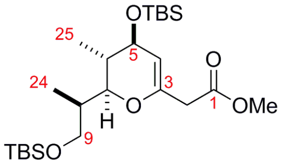

To a solution of **17a** (396 mg, 1.11 mmol) in MeOH (14 mL) at RT was added $CeCl_3$•$7H_2O$ (560 mg, 1.50 mmol) and the solution stirred for 30 min. The reaction mixture was then cooled to –78 °C and $NaBH_4$ (126 mg, 1.34 mmol) added in one portion. After 1.5 h at –78 °C, TLC analysis [Et_2O:petroleum ether (40–60), 8:2] showed no remaining starting material and so the reaction was diluted with Et_2O (25 mL) and quenched by the addition of sat. aq. $NaHCO_3$ (20 mL). The aqueous layer was separated and extracted with Et_2O (2 × 25 mL). The combined organic layers were dried ($MgSO_4$) and concentrated *in vacuo* to give a pale yellow oil (394 mg) which was used directly in the next step.

The crude product was then re-dissolved in CH_2Cl_2 (40 mL) and cooled to 0 °C. To this was added imidazole (375 mg, 5.51 mmol), TBSCl (415 mg, 2.75 mmol), and DMAP (67 mg, 0.55 mmol) and the reaction mixture stirred at RT for 18 h. The reaction was quenched by the addition of sat. aq. NH_4Cl (50 mL) and the phases separated. The aqueous layer was extracted with Et_2O (2 × 50 mL) and the combined organic layers dried ($MgSO_4$) and concentrated *in vacuo* to give a yellow residue. Purification by column chromatography [SiO_2, petroleum ether (40–60):Et_2O, 96:4] gave the title compound **19a** (470 mg, 90% over 2 steps) as a colourless oil which crystallised at low temperature.

***R*$_f$** = 0.35 [petroleum ether (40–60):Et_2O, 94:6]; $[\alpha]_D^{25.0}$ = +13.9 (*c* = 0.50, $CHCl_3$); **IR** (film) ν_{max}/cm^{-1} 2954, 2929, 2857, 1747, 1682, 1469, 1405, 1358, 1334, 1249, 1205, 1165, 1098, 1066, 1040, 1007; **^{1}H NMR** ($CDCl_3$, 400 MHz) δ = 4.59 (1H, d, *J* = 1.7 Hz, C4H), 3.96 (1H, d, *J* = 8.0 Hz, C5H), 3.81 (1H, dd, *J* = 10.1, 2.7 Hz, C7H), 3.68 (3H, s, $C1O_2CH_3$), 3.60 (1H, dd, *J* = 9.7, 7.5 Hz, C9$H_A$$H_B$), 3.48 (1H, dd, *J* = 9.7, 6.2 Hz, C9$H_A$$H_B$), 3.04 (1H, d, *J* = 15.4 Hz, C2$H_A$$H_B$), 2.99 (1H, d, *J* = 15.4 Hz, C2$H_A$$H_B$), 2.00–1.92 (1H, m, C8H), 1.80–1.72 (1H, m,

C6H), 0.94–0.89 (12H, m, C(CH_3)$_3$ of *t*Bu and C25H$_3$), 0.89 (9H, s, C(CH_3)$_3$ of *t*Bu), 0.84 (3H, d, J = 6.9 Hz, C24H$_3$), 0.09 (3H, s, Si(CH_3)$_2$), 0.08 (3H, s, Si(CH_3)$_2$), 0.03 (6H, s, Si(CH_3)$_2$); **^{13}C NMR** (CDCl$_3$, 100 MHz) δ = 170.4 (*C*1O$_2$CH$_3$), 148.1 (C3), 103.5 (C4H), 78.8 (C7H), 70.7 (C5H), 65.3 (C9H$_2$), 51.8 (C1O$_2$*C*H$_3$), 39.8 (C2H$_2$), 36.6 (C6H), 36.3 (C8H), 25.9 (6C, C(*C*H$_3$)$_3$ of *t*Bu), 18.3 (*C*(CH$_3$)$_3$ of *t*Bu), 18.1 (*C*(CH$_3$)$_3$ of *t*Bu), 13.9 (C25H$_3$), 9.4 (C24H$_3$), –4.1 (Si(*C*H$_3$)$_2$), –4.6 (Si(*C*H$_3$)$_2$), –5.4 (2C, Si(*C*H$_3$)$_2$); **HRMS** (+ESI) Found [M+Na]$^+$ = 495.2927; $C_{24}H_{48}O_5Si_2Na$ requires 495.2938, Δ 2.22 ppm.

((2*S*,4*S*,5*S*,6*S*)-4-(*tert*-Butyl-dimethyl-silanyloxy)-6-((*R*)-2-(*tert*-butyl-dimethylsilanyloxy)-1-methyl-ethyl)-2-methoxy-5-methyl-tetrahydro-pyran-2-yl)-acetic acid methyl ester 20a

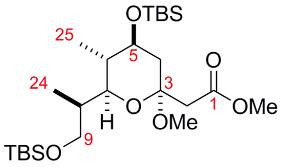

To a solution of **19a** (156 mg, 0.33 mmol) in CH_2Cl_2 (10 mL) was added MeOH (330 μL) and (±)-CSA (8 mg, 0.033 mmol) sequentially at RT. The mixture was stirred for 2 h, after which the reaction was quenched by the addition of sat. aq. $NaHCO_3$ (10 mL). The phases were separated and the aqueous layer extracted with CH_2Cl_2 (3 × 10 mL). The combined organic layers were dried ($MgSO_4$) and concentrated *in vacuo*. Purification by column chromatography [SiO_2, petroleum ether (40–60):Et_2O, 95:5→9:1] gave the title compound **20a** (113 mg, 68%) as a colourless oil.†

***R*$_f$** = 0.66 [petroleum ether (40–60):Et_2O, 85:15]; $[\alpha]_D^{25.0}$ = –48.1 (c = 0.51, CHCl$_3$); **IR** (film) ν_{max}/cm^{-1} 2954, 2929, 2858, 1746, 1472, 1437, 1382, 1360, 1314, 1251, 1220, 1148, 1128, 1067, 1034, 1005; **^{1}H NMR** (CDCl$_3$, 400 MHz) δ = 3.67 (3H, s, C1O$_2$CH$_3$), 3.66–3.54 (2H, m, C5H and C9H_AH$_B$), 3.48–3.41 (2H, m, C7H and C9H$_A$$H_B$), 3.19 (3H, s, C3OCH$_3$), 2.61 (2H, s, C2H$_AH_B$), 2.12 (1H, dd, J = 12.9, 4.7 Hz, equatorial C4H_AH$_B$), 1.88–1.80 (1H, m, C8H), 1.67

† Despite performing this reaction under identical reaction conditions, this procedure was found to be unreliable, giving yields ranging between 20–68%.

(1H, dd, J = 12.7, 11.0 Hz, axial C4H_AH_B), 1.47–1.38 (1H, m, C6H), 0.89 (9H, s, C(CH_3)$_3$ of *t*Bu), 0.88 (9H, s, C(CH_3)$_3$ of *t*Bu), 0.85 (3H, d, J = 6.5 Hz, C25H_3), 0.77 (3H, d, J = 6.9 Hz, C24H_3), 0.07 (6H, s, Si(CH_3)$_2$), 0.04 (3H, s, Si(CH_3)$_2$), 0.02 (3H, s, Si(CH_3)$_2$); **^{13}C NMR** ($CDCl_3$, 100 MHz) δ = 169.9 (*C*1O_2CH_3), 98.7 (C3), 72.5 (C7H), 70.7 (C5H), 65.6 (C9H_2), 51.5 (C1O_2*C*H_3), 47.7 (C3O*C*H_3), 43.1 (C4H_2), 41.9 (C2H_2), 39.7 (C6H), 36.6 (C8H), 25.9 (6C, C(*C*H_3)$_3$ of *t*Bu), 18.2 (*C*(CH_3)$_3$ of *t*Bu), 18.0 (*C*(CH_3)$_3$ of *t*Bu), 12.5 (C25H_3), 8.9 (C24H_3), –4.0 (Si(*C*H_3)$_2$), –4.7 (Si(*C*H_3)$_2$), –5.3 (Si(*C*H_3)$_2$), –5.4 (Si(*C*H_3)$_2$); **HRMS** (+ESI) Found $[M+Na]^+$ = 527.3198; $C_{25}H_{52}O_6Si_2Na$ requires 527.3200, Δ 0.38 ppm.

(2*R*,3*R*,4*R*)-1-(Benzyloxy)-2,4-dimethylhex-5-yn-3-ol 10b

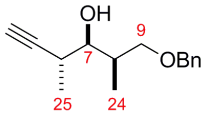

To a stirring solution of $Pd(OAc)_2$ (150 mg, 0.67 mmol) in THF (80 mL) at –78 °C was added PPh_3 (170 mg, 0.67 mmol). The resulting yellow solution was stirred for 10 mins at which point **9** (1.73 g, 11.7 mmol) and **8b** (1.54 g, 8.36 mmol)[3] were added dropwise sequentially. After stirring for 5 mins $ZnEt_2$ (1 M in hexanes, 25.0 mL, 25.0 mmol) was added to the mixture over a period of 1 h. The reaction mixture was stirred for 30 mins at –78 °C before being slowly warmed to –25 °C and maintained at this temperature for a further 24 h. The reaction was quenched by the addition of sat. aq. NH_4Cl (160 mL), diluted with Et_2O (200 mL) and warmed to RT. The layers were separated and the aqueous layer further extracted with Et_2O (3 × 150 mL), and the combined organic layers dried ($MgSO_4$), and concentrated *in vacuo*. Purification by column chromatography [SiO_2, petroleum ether (40–60):Et_2O:Et_3N, 98:1:1→95:4:1] gave the title compound **10b** (4.01 g, 71%).as a pale yellow oil as the major diastereomer (dr = 94:6).

Diastereomeric ratio ascertained by ^{1}H NMR spectroscopy of the crude mixture; δ_H 0.99 (3H, d, J = 7.0 Hz, C24H_3 major), 0.93 (3H, d, J = 6.9 Hz, C24H_3 minor).

***R*$_f$** = 0.10 [petroleum ether (40–60):Et_2O, 9:1]; $[\alpha]_D^{25.0}$ = +10.9 (c = 0.73, $CHCl_3$); **IR** (film) ν_{max}/cm^{-1} 3500, 3294, 2972, 2875, 1454, 1363, 1207, 1095, 1028; **^{1}H NMR** ($CDCl_3$, 400 MHz) δ

= 7.38–7.28 (5H, m, Ph), 4.51 (2H, s, OCH_2Ph), 3.58–3.53 (2H, m, C7H and C9H_AH$_B$), 3.48 (1H, dd, J = 9.2, 5.1 Hz, C9H$_A$$H_B$), 2.69–2.62 (1H, m, C6H), 2.35 (1H, d, J = 5.3 Hz, CHO*H*), 2.13 (1H, d, J = 2.4 Hz, C4H), 2.05–1.95 (1H, m, C8H), 1.21 (3H, d, J = 7.0 Hz, C25H_3), 0.99 (3H, d, J = 7.0 Hz, C24H_3); **^{13}C NMR** ($CDCl_3$, 100 MHz) δ = 138.3 (*ipso* Ph), 128.4 (2C, *ortho* Ph), 127.6 (2C, *meta* Ph), 127.5 (*para* Ph), 86.2 (C5), 75.7 (C7H), 73.9 (C9H_2), 73.3 (O*C*H_2Ph), 70.5 (C4H), 36.3 (C8H), 30.5 (C6H), 17.7 (C25H_3), 10.9 (C24H_3); **HRMS** (+ESI) Found $[M+Na]^+$ = 255.1353; $C_{15}H_{20}O_2Na$ requires 255.1361, Δ 3.14 ppm.

1-((1*R*,2*R*)-1-((*R*)-2-Benzyloxy-1-methyl-ethyl)-2-methyl-but-3-ynyloxymethyl)-4-methoxy-benzene S4

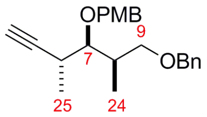

To a solution of **10b** (13.2 g, 51.5 mmol) in DMF (180 mL) and THF (180 mL) at 0 °C was added NaH (60% in mineral oil, 2.47 g, 61.8 mmol). The mixture was stirred for 45 min at RT, after which PMBBr (9.00 mL, 61.5 mmol) was added dropwise and the reaction stirred for 2 h at RT. The reaction was quenched by the addition of sat. aq. NH_4Cl (200 mL) and diluted with Et_2O (200 mL). The phases were separated and the aqueous layer extracted with Et_2O (2 × 150 mL). The combined organics were washed with sat. aq. LiCl (200 mL), dried ($MgSO_4$) and concentrated *in vacuo* to give a pale yellow oil. Purification by column chromatography [SiO_2, petroleum ether (40–60):Et_2O:Et_3N, 94:5:1→90:9:1] gave the title compound **S4** (16.8 g, 86%) as a colourless oil.

***R*$_f$** = 0.29 [petroleum ether (40–60):Et_2O, 9:1]; $[\alpha]_D^{25.0}$ = –34.7 (*c* = 0.73, $CHCl_3$); **IR** (film) ν_{max}/cm^{-1} 3293, 2971, 2932, 2906, 2859, 1612, 1514, 1454, 1362, 1301, 1246, 1172, 1073, 1036; **^{1}H NMR** ($CDCl_3$, 400 MHz) δ = 7.36–7.30 (4H, m, Ph), 7.28–7.24 (3H, m, Ph and (C*H*)$_2$COCH_3 of PMB), 6.85 (2H, d, J = 8.6 Hz, C(C*H*)$_2$ of PMB), 4.77 (1H, d, J = 10.8 Hz, OCH_AH$_B$Ph), 4.50–4.42 (3H, m, OCH$_A$$H_B$Ph and OC$H_2$Ph(OC$H_3$)), 3.80 (3H, s, OC$H_3$), 3.54 (1H, dd, J = 7.0, 4.1 Hz, C7H), 3.46 (1H, dd, J = 9.1, 7.5 Hz, C9H_AH$_B$), 3.37 (1H, dd, J = 9.1, 5.7 Hz, C9H$_A$$H_B$), 2.80–2.72 (1H, m, C6H), 2.17–2.10 (1H, m, C8H), 2.08 (1H, d, J = 2.4 Hz, C4H), 1.20 (3H, d, J

= 7.1 Hz, C25H$_3$), 0.94 (3H, d, *J* = 6.9 Hz, C24H$_3$); **^{13}C NMR** (CDCl$_3$, 100 MHz) δ = 159.1 (Ar), 138.5 (*ipso* Ph), 131.2 (2C, Ar), 129.4 (2C, Ar), 128.3 (2C, *ortho* Ph), 127.7 (2C, *meta* Ph), 127.5 (*para* Ph), 113.6 (Ar), 87.4 (C5), 81.6 (C7H), 74.3 (O*C*H$_2$Ar), 73.0 (2C, C9H$_2$ and O*C*H$_2$Ph), 69.3 (C4), 55.3 (OCH$_3$), 36.0 (C8H), 29.5 (C6H), 17.9 (C25H$_3$), 11.4 (C24H$_3$); **HRMS** (+ESI) Found [M+H]$^+$ = 353.2112; $C_{23}H_{29}O_3$ requires 353.2117, Δ 1.42 ppm.

(4*R*,5*R*,6*R*)-7-Benzyloxy-5-(4-methoxy-benzyloxy)-4,6-dimethyl-hept-2-ynoic acid ethyl ester S5

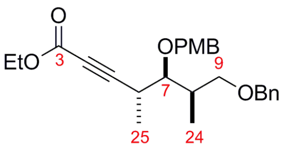

To a solution of **S4** (1.53 g, 4.34 mmol) in THF (65 mL) at –78 °C was added *n*BuLi (1.47 M in hexanes, 3.25 mL, 4.78 mmol) dropwise. The reaction was stirred for 1 h, after which ethyl chloroformate (625 μL, 6.51 mmol) was added and the reaction allowed to warm to RT over 18 h. The reaction was quenched by the addition of sat. aq. NH_4Cl (80 mL) and diluted with Et_2O (80 mL). The aqueous layer was separated and extracted with Et_2O (2 × 80 mL). The combined organic layers were dried ($MgSO_4$), filtered and concentrated *in vacuo* to afford a pale yellow oil. Purification by column chromatography [SiO_2, petroleum ether (40–60):Et_2O, 8:2] gave the title compound **S5** (1.74 g, 95%) as a pale yellow oil.

***R*$_f$** = 0.23 [petroleum ether (40–60):Et_2O, 8:2]; $[\alpha]_D^{25.0}$ = –4.1 (*c* = 0.815, CHCl$_3$); **IR** (film) ν_{max}/cm^{-1} 2973, 2938, 2921, 2240, 1706, 1613, 1514, 1454, 1365, 1301, 1243, 1173, 1033; **^{1}H NMR** (CDCl$_3$, 400 MHz) δ = 7.37–7.30 (4H, m, Ph), 7.29–7.25 (3H, m, Ph and (C*H*)$_2$COCH$_3$ of PMB), 6.85 (2H, d, *J* = 8.6 Hz, C(C*H*)$_2$ of PMB), 4.76 (1H, d, *J* = 10.5 Hz, OCH_AH$_B$Ph), 4.50–4.42 (3H, m, OCH$_A$$H_B$Ph and OC*H*$_2$Ph(OCH$_3$)), 4.21 (2H, q, *J* = 7.1 Hz, OC*H*$_2$CH$_3$), 3.80 (3H, s, OCH$_3$), 3.63 (1H, dd, *J* = 7.6, 3.6 Hz, C7H), 3.47 (1H, dd, *J* = 9.0, 8.0 Hz, C9H_AH$_B$), 3.37 (1H, dd, *J* = 9.1, 5.4 Hz, C9H$_A$$H_B$), 2.88 (1H, quint, *J* = 7.2 Hz, C6H), 2.12–2.05 (1H, m, C8H), 1.29 (3H, t, *J* = 7.1 Hz, OCH$_2$C*H*$_3$), 1.22 (3H, d, *J* = 7.1 Hz, C25H$_3$), 0.91 (3H, d, *J* = 6.9 Hz, C24H$_3$); **^{13}C NMR** (CDCl$_3$, 100 MHz) δ = 159.2 (Ar), 153.8 (C3), 138.4 (*ipso* Ph), 130.8 (2C, Ar), 129.6 (2C, Ar), 128.4 (2C, *ortho* Ph), 127.7 (2C, *meta* Ph), 127.6 (*para* Ph), 113.7 (Ar), 91.9 (C5), 81.0

(C7H), 74.6 (OCH_2Ar), 74.1 (C4), 73.1 (OCH_2Ph), 72.7 (C9H_2), 61.7 (OCH_2CH_3), 55.3 (OCH_3), 35.8 (C8H), 29.9 (C6H), 16.8 (C25H_3), 14.0 (OCH_2CH_3), 10.9 (C24H_3); **HRMS** (+ESI) Found $[M+Na]^+$ = 447.2151; $C_{26}H_{32}O_5Na$ requires 447.2147, Δ 0.89 ppm.

(7*R*,9*S*,10*S*)-9-((*R*)-2-Benzyloxy-1-methyl-ethyl)-7-hydroxy-10-methyl-8-oxa-1,4-dithiaspiro[4.5]dec-7-yl)-acetic acid methyl ester 13b

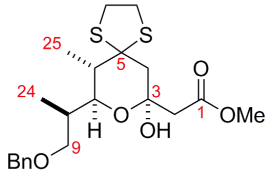

To a solution of *i*Pr_2NH (440 μL, 3.12 mmol) in THF (5 mL) at –78 °C was added *n*BuLi (1.5 N in hexanes, 2.08 mL, 3.12 mmol) dropwise. The solution was allowed to warm to 0 °C, stirred for 10 min and then re-cooled to –78 °C. Methyl acetate (260 μL, 3.25 mmol) was added dropwise and the reaction stirred for 1 h. This solution of the preformed anion was then cannulated quickly to a solution of **S5** (575 mg, 1.35 mmol) in THF (6 mL) at –78 °C. The reaction was then warmed to RT over 18 h. The reaction was quenched with sat. aq. NH_4Cl (10 mL), the aqueous layer separated and extracted with Et_2O (3 × 15 mL). The combined organic layers were dried ($MgSO_4$) and concentrated *in vacuo* to give **11b** (3.12 mmol, assumed quant.) as a pale yellow oil which was used directly in the next step.

To a solution of **11b** (3.12 mmol, assumed quant.) in MeOH (18 mL) and CH_2Cl_2 (18 mL) at –10 °C was added 1,2-ethanedithiol (125 μL, 1.49 mmol), followed by NaOMe (80 mg, 1.49 mmol). The reaction was allowed to warm to RT and stirred for 18 h. The reaction was quenched by the addition of sat. aq. NH_4Cl (25 mL). The aqueous layer was extracted with Et_2O (3 × 20 mL). The organic phases were dried ($MgSO_4$) and concentrated *in vacuo*. The resulting pale yellow oil (**12b**) was dissolved in CH_2Cl_2 (80 mL) and pH 7 phosphate buffer (8 mL) at RT. DDQ (340 mg, 1.49 mmol) was then added and the reaction mixture stirred for 3 h. The reaction was quenched by the addition of sat. aq. NH_4Cl (50 mL) and the phases separated. The aqueous layer was extracted with CH_2Cl_2 (2 × 25 mL) and Et_2O (15 mL). The combined organic layers

were washed with H_2O (60 mL), dried ($MgSO_4$) and concentrated *in vacuo* to give a dark brown oil. The crude product was dissolved in CH_2Cl_2 and stirred with aminoethyl polystyrene quadra gel (1.0 g) overnight. Filtration, evaporation and purification by column chromatography [SiO_2, petroleum ether (40–60):Et_2O, 7:3] gave the title compound **13b** (345 mg, 60% over 3 steps) as a colourless oil.

***R*f** = 0.31 [petroleum ether (40–60):Et_2O, 6:4]; $[\alpha]_D^{25.0}$ = –9.9 (*c* = 0.91, $CHCl_3$); **IR** (film) ν_{max}/cm^{-1} 3456, 2969, 2924, 2875, 1717, 1453, 1437, 1347, 1170, 1092, 1002; **^{1}H NMR** ($CDCl_3$, 400 MHz) δ = 7.37–7.29 (4H, m, Ph), 7.28–7.23 (1H, m, Ph), 4.86 (1H, d, *J* = 1.5 Hz, O*H*), 4.55 (1H, d, *J* = 12.0 Hz, OCH_AH_BPh with H_A italic), 4.46 (1H, d, *J* = 12.0 Hz, $OCH_A\textit{H}_B Ph$), 3.93 (1H, dd, *J* = 10.1, 2.1 Hz, C7H), 3.65 (3H, s, $C1O_2CH_3$), 3.40 (1H, dd, *J* = 9.0, 6.9 Hz, $C9\textit{H}_AH_B$), 3.31–3.23 (5H, m, $C9H_A\textit{H}_B$ and SCH_2CH_2S), 2.60 (1H, d, *J* = 13.8 Hz, $C2\textit{H}_AH_B$), 2.56 (2H, s, $C4H_AH_B$), 2.31 (1H, dd, *J* = 13.8, 1.5 Hz, $C2H_A\textit{H}_B$), 2.13–2.07 (1H, m, C8H), 1.91–1.87 (1H, m, C6H), 1.09 (3H, d, *J* = 6.6 Hz, $C25H_3$), 0.84 (3H, d, *J* = 6.9 Hz, $C24H_3$); **^{13}C NMR** ($CDCl_3$, 100 MHz) δ = 171.6 ($\textit{C}1O_2CH_3$), 138.8 (*ipso* Ph), 128.3 (2C, *ortho* Ph), 127.6 (2C, *meta* Ph), 127.4 (*para* Ph), 95.0 (C3), 73.6 ($C9H_2$), 73.3 (C7H), 73.2 ($O\textit{C}H_2Ph$), 70.0 (C5), 51.7 ($C1O_2\textit{C}H_3$), 51.6 ($C2H_2$), 45.3 ($C4H_2$), 43.3 (C6H), 41.2 ($S\textit{C}H_2CH_2S$), 38.8 ($S\textit{C}H_2\textit{C}H_2S$), 34.7 (C8H), 12.0 ($C25H_3$), 9.6 ($C24H_3$); **HRMS** (+ESI) Found $[M+Na]^+$ = 449.1439; $C_{21}H_{30}O_5S_2Na$ requires 449.1432, Δ 1.56 ppm.

((7*R*,9*S*,10*S*)-9-((*R*)-2-Benzyloxy-1-methyl-ethyl)-7-methoxy-10-methyl-8-oxa-1,4-dithiaspiro[4.5]dec-7-yl)-acetic acid methyl ester S6

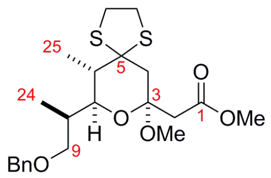

To a solution of **13b** (275 mg, 0.64 mmol) in MeOH (6 mL) at RT was added trimethylorthoformate (1.05 mL, 9.67 mmol) and pyridinium *p*-toluenesulphonate (325 mg, 1.29 mmol) sequentially. The reaction was stirred for 18 h at RT after which it was quenched by the addition of sat. aq. NH_4Cl (25 mL). The resulting mixture was extracted with Et_2O (3 ×

20 mL) and the combined organic layers dried ($MgSO_4$) and concentrated *in vacuo* to give a white solid. Purification by column chromatography [SiO_2, petroleum ether (40–60):Et_2O, 7:3] gave the title compound **S6** (230 mg, 81%) as a colourless oil.

***R*f** = 0.36 [petroleum ether (40–60):Et_2O, 7:3]; $[\alpha]_D^{25.0}$ = –28.9 (*c* = 0.425, $CHCl_3$); **IR** (film) ν_{max}/cm^{-1} 2969, 2926, 2881, 1737, 1454, 1436, 1319, 1230, 1191, 1096, 1025; **^{1}H NMR** ($CDCl_3$, 400 MHz) δ = 7.37–7.29 (4H, m, Ph), 7.28–7.26 (1H, m, Ph), 4.51 (1H, d, *J* = 11.9 Hz, OCH_AH_BPh), 4.46 (1H, d, *J* = 11.9 Hz, OCH_AH_BPh), 3.73 (1H, dd, *J* = 10.1, 1.9 Hz, C7H), 3.66 (3H, s, $C1O_2CH_3$), 3.57 (1H, t, *J* = 8.6 Hz, $C9H_AH_B$), 3.33 (1H, dd, *J* = 8.6, 6.5 Hz, $C9H_AH_B$), 3.25 (3H, s, $C3OCH_3$), 3.25–3.16 (4H, m, SCH_2CH_2S), 2.66 (2H, s, $C2H_AH_B$), 2.60 (2H, s, $C4H_2$), 2.18–2.10 (1H, m, C8H), 1.87–1.82 (1H, m, C6H), 1.07 (3H, d, *J* = 6.6 Hz, $C25H_3$), 0.84 (3H, d, *J* = 6.9 Hz, $C24H_3$); **^{13}C NMR** ($CDCl_3$, 100 MHz) δ = 169.5 ($C1O_2CH_3$), 138.5 (*ipso* Ph), 128.3 (2C, *ortho* Ph), 127.6 (2C, *meta* Ph), 127.5 (*para* Ph), 97.8 (C3), 73.3 (C7H), 73.2 ($C9H_2$), 73.0 (OCH_2Ph), 69.7 (C5), 51.7 ($C1O_2CH_3$), 50.7 ($C2H_2$), 47.5 ($C3OCH_3$), 43.3 (C6H), 41.9 ($C4H_2$), 41.7 (SCH_2CH_2S), 38.6 (SCH_2CH_2S), 34.5 (C8H), 11.9 ($C25H_3$), 9.4 ($C24H_3$); **HRMS** (+ESI) Found $[M+Na]^+$ = 463.1567; $C_{22}H_{32}O_5S_2Na$ requires 463.1589, Δ 4.75 ppm.

((2*S*,5*S*,6*S*)-6-((*R*)-2-Benzyloxy-1-methyl-ethyl)-2-methoxy-5-methyl-4-oxo-tetrahydropyran-2-yl)-acetic acid methyl ester 14b

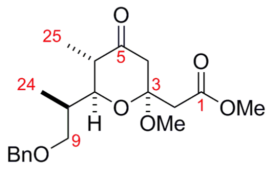

To a solution of **S6** (513 mg, 1.16 mmol) in MeCN (20 mL) and H_2O (2.6 mL) at 0 °C was added a freshly prepared solution of BTI (0.033 M in MeCN, 70.0 mL, 2.33 mmol) dropwise. The reaction mixture was stirred at 0 °C for 2 h after which it was quenched by the addition of a mixture of sat. aq. Na_2SO_3 (50 mL) and sat. aq. $NaHCO_3$ (50 mL) (1:1). The reaction was diluted with EtOAc (70 mL) and the phases separated. The aqueous layer was extracted with EtOAc (2 × 70 mL) and the combined organic layers dried ($MgSO_4$) and concentrated *in vacuo*. Purification

by column chromatography [SiO_2, Et_2O:petroleum ether (40–60), 8:2] gave the title compound **14b** (368 mg, 87%) as a pale yellow oil.

***R*f** = 0.25 [petroleum ether (40–60):Et_2O, 7:3]; $[\alpha]_D^{25.0}$ = –68.0 (*c* = 0.57, $CHCl_3$); **IR** (film) ν_{max}/cm^{-1} 2973, 2937, 2880, 1720, 1454, 1437, 1315, 1244, 1195, 1166, 1139, 1096, 1047, 1016; **^{1}H NMR** ($CDCl_3$, 400 MHz) δ = 7.40–7.26 (5H, m, Ph), 4.49 (2H, s, OCH_AH_BPh), 3.81 (1H, dd, *J* = 10.7, 1.7 Hz, C7H), 3.69 (3H, s, $C1O_2CH_3$), 3.61 (1H, t, *J* = 8.8 Hz, $C9H_AH_B$), 3.41 (1H, dd, *J* = 8.9, 5.9 Hz, $C9H_AH_B$), 3.17 (3H, s, $C3OCH_3$), 2.93 (1H, d, *J* = 14.0 Hz, $C4H_AH_B$), 2.83 (1H, d, *J* = 13.9 Hz, $C2H_AH_B$), 2.65 (1H, d, *J* = 13.9 Hz, $C2H_AH_B$), 2.63 (1H, d, *J* = 14.0 Hz, $C4H_AH_B$), 2.48–2.42 (1H, m, C6H), 2.15–2.08 (1H, m, C8H), 0.98 (3H, d, *J* = 6.6 Hz, $C25H_3$), 0.93 (3H, d, *J* = 7.0 Hz, $C24H_3$); **^{13}C NMR** ($CDCl_3$, 100 MHz) δ = 206.9 (C5O), 169.3 (*C*$1O_2CH_3$), 138.3 (*ipso* Ph), 128.4 (2C, *ortho* Ph), 127.6 (2C, *meta* Ph), 127.5 (*para* Ph), 100.8 (C3), 74.0 (C7H), 73.0 (O*C*H_2Ph), 72.6 ($C9H_2$), 51.8 ($C1O_2CH_3$), 49.7 ($C4H_2$), 48.2 ($C3OCH_3$), 45.6 (C6H), 41.0 ($C2H_2$), 34.5 (C8H), 9.0 ($C24H_3$), 8.7 ($C25H_3$); **HRMS** (+ESI) Found $[M+Na]^+$ = 387.1777; $C_{20}H_{28}O_6Na$ requires 387.1784, Δ 1.81 ppm.

((5*S*,6*S*)-6-((*R*)-2-Benzyloxy-1-methyl-ethyl)-5-methyl-4-oxo-5,6-dihydro-4*H*-pyran-2-yl)-acetic acid methyl ester 17b

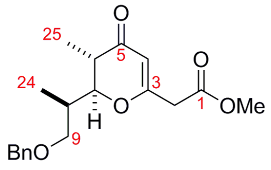

To a solution of **14b** (285 mg, 0.78 mmol) in CH_2Cl_2 (20 mL) at –10 °C was added TfOH (85 μL, 0.94 mmol) dropwise. After approximately 2 mins the reaction was quenched by the addition of sat. aq. $NaHCO_3$ (5 mL) and extracted with CH_2Cl_2 (2 × 20 mL) and Et_2O (2 × 20 mL). The combined organic extracts were dried ($MgSO_4$) and concentrated *in vacuo* to give a colourless oil. Purification by column chromatography [SiO_2, Et_2O:petroleum ether (40–60), 1:1] gave the title compound **17b** (228 mg, 88%) as a colourless oil.

***R*f** = 0.14 [petroleum ether (40–60):Et_2O, 1:1]; $[\alpha]_D^{25.0}$ = –96.1 (*c* = 0.71, $CHCl_3$); **IR** (film) ν_{max}/cm^{-1} 2971, 2938, 2904, 2882, 1743, 1671, 1619, 1455, 1397, 1343, 1256, 1200, 1152, 1086, 1012; **^{1}H NMR** ($CDCl_3$, 400 MHz) δ = 7.36–7.25 (5H, m, Ph), 5.39 (1H, s, C4H), 4.51 (2H, s, OCH_AH_BPh), 4.34 (1H, dd, *J* = 13.0, 2.3 Hz, C7H), 3.69 (3H, s, $C1O_2CH_3$), 3.54 (1H, t, *J* = 8.9, 8.6 Hz, C9H_AH_B), 3.43 (1H, dd, *J* = 9.1, 5.9 Hz, C9H_AH_B), 3.25 (1H, d, *J* = 15.9 Hz, C2H_AH_B), 3.19 (1H, d, *J* = 15.9 Hz, C2H_AH_B), 2.55–2.48 (1H, m, C6H), 2.22–2.15 (1H, m, C8H), 1.09 (3H, d, *J* = 6.9 Hz, $C25H_3$), 0.96 (3H, d, *J* = 7.0 Hz, $C24H_3$); **^{13}C NMR** ($CDCl_3$, 100 MHz) δ = 195.2 (C5O), 168.2 (*C*1O_2CH_3), 167.9 (C3), 138.3 (*ipso* Ph), 128.4 (2C, *ortho* Ph), 127.6 (2C, *meta* Ph), 127.5 (*para* Ph), 105.4 (C4H), 83.6 (C7H), 73.2 (O*C*H_2Ph), 71.7 ($C9H_2$),, 52.3 ($C1O_2CH_3$), 40.3 ($C2H_2$), 40.3 (C6H), 34.9 (C8H), 9.7 ($C24H_3$), 9.6 ($C25H_3$); **HRMS** (+ESI) Found $[M+H]^+$ = 333.1695; $C_{19}H_{25}O_5$ requires 333.1702, Δ 2.10 ppm.

(4*R*,5*S*,6*S*)-6-((*R*)-2-Benzyloxy-1-methyl-ethyl)-4-(*tert*-butyl-dimethyl-silanyloxy)-5-methyl-5,6-dihydro-4*H*-pyran-2-yl]-acetic acid methyl ester 19b

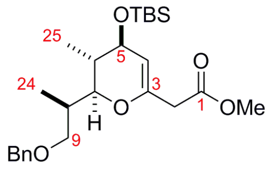

To a solution of **17b** (223 mg, 0.67 mmol) in MeOH (8 mL) at RT was added $CeCl_3$•$7H_2O$ (338 mg, 0.91 mmol) and the solution stirred for 30 min. The reaction mixture was then cooled to –78 °C and $NaBH_4$ (76 mg, 2.01 mmol) added in one portion. After 1.5 h at –78 °C, TLC analysis [Et_2O:petroleum ether (40–60), 7:3] showed no remaining starting material and so the reaction was diluted with Et_2O (10 mL) and quenched by the addition of sat. aq. $NaHCO_3$ (10 mL). The aqueous layer was separated and extracted with Et_2O (2 × 10 mL). The combined organic layers were dried ($MgSO_4$) and concentrated *in vacuo* to give a pale yellow oil (195 mg) which was used directly in the next step.

The crude product was then re-dissolved in CH_2Cl_2 (15 mL) and cooled to 0 °C. To this was added imidazole (250 mg, 3.36 mmol), TBSCl (253 mg, 1.68 mmol), and DMAP (40 mg, 0.33 mmol) and the reaction mixture stirred at RT for 18 h. The reaction was quenched by the

addition of sat. aq. NH_4Cl (25 mL) and the phases separated. The aqueous layer was extracted with Et_2O (2 × 25 mL) and the combined organic layers dried ($MgSO_4$) and concentrated *in vacuo* to give a yellow residue. Purification by column chromatography [SiO_2, petroleum ether (40–60):Et_2O, 9:1→8:2] gave the title compound **19b** (292 mg, 97% over 2 steps) as a colourless oil.

***R*$_f$** = 0.54 [petroleum ether (40–60):Et_2O, 7:3]; $[\alpha]_D^{25.0}$ = +16.4 (*c* = 0.50, $CHCl_3$); **IR** (film) ν_{max}/cm^{-1} 2956, 2930, 2884, 2856, 1746, 1681, 1454, 1358, 1251, 1189, 1151, 1072, 1048, 1007; **^{1}H NMR** ($CDCl_3$, 400 MHz) δ = 7.37–7.29 (4H, m, Ph), 7.28–7.26 (1H, m, Ph), 4.59 (1H, d, *J* = 1.6 Hz, C4H), 4.52 (1H, d, *J* = 12.0 Hz, OCH_AH_BPh), 4.47 (1H, d, *J* = 12.0 Hz, OCH_AH_BPh), 3.99 (1H, d, *J* = 8.2 Hz, C5H), 3.87 (1H, dd, *J* = 10.3, 2.5 Hz, C7H), 3.63 (3H, s, $C1O_2CH_3$), 3.50 (1H, t, *J* = 8.9, 7.8 Hz, $C9H_AH_B$), 3.37 (1H, dd, *J* = 9.0, 6.3 Hz, $C9H_AH_B$), 3.05 (1H, d, *J* = 15.4 Hz, $C2H_AH_B$), 2.98 (1H, d, *J* = 15.4 Hz, $C2H_AH_B$), 2.15–2.08 (1H, m, C8H), 1.80–1.70 (1H, m, C6H), 0.95–0.84 (15H, m, $C(CH_3)_3$ of *t*Bu, $C24H_3$ and $C25H_3$), 0.06 (6H, s, $Si(CH_3)_2$); **^{13}C NMR** ($CDCl_3$, 100 MHz) δ = 170.4 ($C1O_2CH_3$), 148.1 (C3), 138.7 (*ipso* Ph), 128.3 (2C, *ortho* Ph), 127.5 (2C, *meta* Ph), 127.4 (*para* Ph), 103.8 (C4H), 79.1 (C7H), 73.2 (OCH_2Ph), 72.9 ($C9H_2$), 70.8 (C5H), 51.8 ($C1O_2CH_3$), 39.8 ($C2H_2$), 36.7 (C8H), 31.9 (C6H), 25.9 (3C, $C(CH_3)_3$ of *t*Bu), 18.3 ($C(CH_3)_3$ of *t*Bu), 13.6 ($C25H_3$), 9.4 ($C24H_3$), –4.5 ($Si(CH_3)_2$), –4.1 ($Si(CH_3)_2$); **HRMS** (+ESI) Found $[M+Na]^+$ = 471.2545; $C_{25}H_{40}O_5SiNa$ requires 471.2543, Δ 0.42 ppm.

(2*R*,3*R*,4*R*)-1-(enzyloxy)-2,4-dimethylhex-5-en-3-ol 22[4]

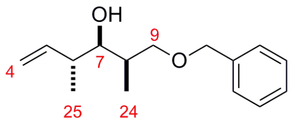

Procedure 1

To a 3-necked flask containing THF (150 mL) at –78 °C was condensed *trans*-but-2-ene (8.50 mL, 94.8 mmol). The resulting solution was stirred for 5 min, after which KO*t*Bu (1 M in THF, 36.4 mL, 36.4 mmol) and *n*BuLi (2.5 M in hexanes, 14.6 mL, 36.4 mmol) were added dropwise sequentially *via* cannula ensuring that the internal temperature did not exceed –70 °C.

The resulting bright yellow solution was warmed to –50 °C and maintained at this temperature for a further 30 min after which it was re-cooled to –78 °C. A solution of (+)-Ipc_2BOMe (13.9 g, 44.0 mmol) in Et_2O (54 mL) was then added dropwise, again ensuring that the temperature did not rise above –70 °C. The colourless mixture was stirred for 45 min at –78 °C and BF_3•OEt_2 (6.30 mL, 51.0 mmol) added slowly. After stirring for a further 5 min, **8b** (5.22 g, 29.3 mmol) in Et_2O (100 mL) was added dropwise. The solution was stirred at –78 °C for 4 h and then treated with 3 M NaOH (21 mL) followed by H_2O_2 (30%, 9 mL) and warmed to RT slowly over 12 h. The reaction mixture was diluted with H_2O (75 mL) and Et_2O (75 mL) and the layers separated. The aqueous layer was further extracted with Et_2O (3 × 100 mL), and the combined organic layers washed with brine (150 mL), dried ($MgSO_4$), and concentrated *in vacuo*. Purification firstly by sublimation of (+)-isopinocampheol (3 days), followed by column chromatography [SiO_2, petroleum ether (40–60):Et_2O, 9:1] gave the title compound **22** (2.54 g, 37%, dr = 86:14) as a yellow oil.

Diastereomeric ratio ascertained by ^{1}H NMR spectroscopy of the crude mixture; δ_H 5.90 (1H, ddd, J = 16.5, 11.2, 8.5 Hz, C5H minor), 5.80 (1H, ddd, J = 16.2, 10.0, 8.4 Hz, C5H major).

Procedure 2

To a solution of *(S,S)-Diisopropyl tartrate (E)-crotylboronate* **21**[†] (17.0 g, 57.0 mmol) in PhMe (160 mL) at RT was added 4 Å MS (6.0 g). The mixture was cooled to –78 °C, at which point a solution of **8b** (8.5 g, 47.5 mmol) in PhMe (15 mL) was added *via* cannula over 30 min. The reaction was stirred at –78 °C for 16 h, warmed to –10 °C and 2 *N* NaOH (130 mL) added dropwise over 15 mins. The mixture was stirred for a further 1 h 30 mins, and then filtered through a pad of Celite® under suction using PhMe (5 × 50 mL). The layers were separated and the aqueous layer further extracted with Et_2O (2 × 200 mL). The combined organic layers were washed with sat. aq. $NaHCO_3$ (200 mL), brine (200 mL), dried ($MgSO_4$) and concentrated *in*

[†]*(S,S)-Diisopropyl tartrate (E)-crotylboronate* **21** prepared according to the procedure detailed by Roush.[5] This reagent was obtained as a 0.4:0.6 mixture of unbound *(S,S)-diisopropyltartrate*: *(S,S)-Diisopropyl tartrate (E)-crotylboronate*, and as such was scaled in order to compensate for this ratio.

vacuo. Purification by column chromatography [SiO_2, petroleum ether (40–60):Et_2O, 7:3] gave the title compound **22** (11.4 g, 70%, dr = 88:12) as a yellow oil.

Diastereomeric ratio ascertained by ^{1}H NMR spectroscopy of the crude mixture; δ_H 5.90 (1H, ddd, J = 16.5, 11.2, 8.5 Hz, C5H minor), 5.80 (1H, ddd, J = 16.2, 10.0, 8.4 Hz, C5H major).

***R*f** = 0.21 [petroleum ether (40–60):Et_2O, 8:2]; $[\alpha]_D^{25.0}$ = –6.4 (c = 0.713, $CHCl_3$); **IR** (film) ν_{max}/cm^{-1} 3468, 3067, 3031, 2971, 2933, 2869, 1639, 1494, 1454, 1364, 1306, 1209, 1094, 1029; **^{1}H NMR** ($CDCl_3$, 400 MHz) δ = 7.36–7.26 (5H, m, Ph), 5.80 (1H, ddd, J = 16.2, 10.0, 8.4 Hz, C5H), 5.15–5.08 (2 × 1H, 2 × dd, broad unresolved, C4H_AH_B), 4.52 (2H, s, OCH_2Ph), 3.57 (1H, dd, J = 9.0, 6.0 Hz, C9H_AH_B), 3.50 (1H, dd, J = 9.2, 5.4 Hz, C9H_AH_B), 3.49–3.45 (1H, m, C7H), 2.28–2.23 (1H, m, C6H), 2.15–2.02 (1H, br s, OH), 1.97–1.93 (1H, m, C8H), 0.98 (3H, d, J = 6.7 Hz, C25H_3), 0.97 (3H, d, J = 6.9 Hz, C24H_3); **^{13}C NMR** ($CDCl_3$, 100 MHz) δ = 141.9 (C5H), 138.4 (*ipso* Ph), 128.5 (2C, *ortho* Ph), 127.8 (*para* Ph), 127.6 (2C, *meta* Ph), 115.7 (C4H_2), 75.4 (C7H), 74.8 (C9H_2), 73.6 (OCH_2Ph), 41.1 (C6H), 35.1 (C8H), 17.7 (C25H_3), 14.0 (C24H_3); **HRMS** (+ESI) Found $[M+Na]^+$ = 257.1513; $C_{15}H_{22}O_2Na$ requires 257.1512, Δ 0.39 ppm.

(2*R*,3*R*,4*R*)-1-(Benzyloxy)-2,4-dimethylhex-5-en-3-yl (2*S*)-3,3,3-trifluoro-2-methoxy-2-phenylpropanoate S7

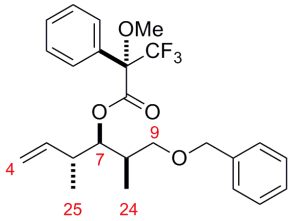

To a solution of **22** (16.6 mg, 70.8 μmol) in CH_2Cl_2 (1.1 mL) at RT was added (*S*)-(–)-α-methyl-α-(trifluoromethyl)phenylacetic acid (51.5 mg, 0.220 mmol) followed by DCC (45.4 mg, 0.220 mmol) and DMAP (26.9 mg, 0.220 mmol). After 20 h the resulting white precipitate was

removed by filtration and the solvent removed *in vacuo*. Purification by column chromatography (SiO_2, hexane:Et_2O, 98:2→95:5) gave the title compound **S7** (22.3 mg, 70%) as a colourless oil.[†]

***R*$_f$** = 0.53 (hexane:Et_2O, 7:3); $[\alpha]_D^{27.4}$ = –25.6 (*c* = 1.32, $CHCl_3$); **IR** (film) ν_{max}/cm^{-1} 3063, 2974, 2937, 2857, 1743, 1497, 1453, 1380, 1364, 1255, 1166, 1105, 1081, 1015, 994; **^{1}H NMR** ($CDCl_3$, 400 MHz) δ = 7.58 (2H, d, *J* = 6.9 Hz, *ortho* Ph), 7.40–7.27 (8H, m, Ph), 5.73 (1H, ddd, *J* = 16.8, 10.6, 8.8 Hz, C5H), 5.30–5.26 (1H, m, C7H), 5.01–4.95 (2 × 1H, 2 × dd, broad unresolved, C4H_AH_B), 4.46 (1H, d, *J* = 12.0 Hz, OC$H_A$$H_B$Ph), 4.37 (1H, d, *J* = 12.0 Hz, OC$H_A$$H_B$Ph), 3.53 (3H, s, OCH_3), 3.19 (2H, d, *J* = 6.6 Hz, C9H_AH_B), 2.58–2.48 (1H, m, C6H), 2.17–2.10 (1H, m, C8H), 1.02 (3H, d, *J* = 6.9 Hz, C25H_3), 0.87 (3H, d, *J* = 6.9 Hz, C24H_3); **^{13}C NMR** ($CDCl_3$, 100 MHz) δ = 166.1 (C=O), 140.1 (C5H), 138.3 (*ipso* benzyl Ph), 132.2 (Ph), 129.5 (Ph), 128.4 (2C, *ortho* benzyl Ph), 128.2 (2C, Ph), 127.8 (*para* benzyl Ph), 127.7 (2C, Ph), 127.6 (2C, *meta* benzyl Ph), 123.5 (q, *J* = 288.5 Hz, *C*F_3), 116.0 (C4H_2), 84.5 (q, *J* = 27.6 Hz, *C*CF_3), 79.9 (C7H), 73.2 (O*C*H_2Ph), 72.3 (C9H_2), 55.5 (OCH_3), 40.8 (C6H), 35.2 (C8H), 17.6 (C25H_3), 11.2 (C24H_3); **^{19}F** ($CDCl_3$, 400 MHz) δ = –71.21; **HRMS** (+ESI) Found $[M+H]^+$ = 451.2085; $C_{25}H_{30}O_4F_3$ requires 451.2091, Δ 1.33 ppm.

(2*R*,3*S*,4*R*)-1-(Benzyloxy)-2,4-dimethylhex-5-en-3-yl (2*R*)-3,3,3-trifluoro-2-methoxy-2-phenylpropanoate S8

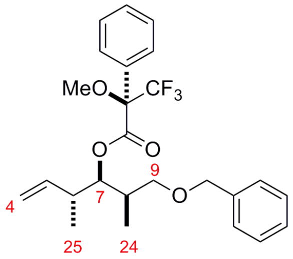

To a solution of **22** (18.8 mg, 80 μmol) in CH_2Cl_2 (1.25 mL) at RT was added (*R*)-(+)-α-methyl-α-(trifluoromethyl)phenylacetic acid (93.7 mg, 0.40 mmol) followed by DCC (82.5 mg, 0.40 mmol) and DMAP (48.9 mg, 0.40 mmol). After 20 h the resulting white precipitate was

[†] The minor diastereomer obtained from **22** could not be removed from the reaction at this stage.

removed by filtration and the solvent removed *in vacuo*. Purification by column chromatography (SiO_2, hexane:Et_2O, 98:2→95:5) gave the title compound **S8** (26.6 mg, 74%) as a colourless oil.†

***R*$_f$** = 0.63 (hexane:Et_2O, 7:3); $[\alpha]_D^{27.5}$ = –7.8 (*c* = 1.39, $CHCl_3$); **IR** (film) ν_{max}/cm^{-1} 3071, 2975, 2944, 2853, 1742, 1497, 1453, 1380, 1364, 1254, 1166, 1105, 1082, 1014, 994, 920; **^{1}H NMR** ($CDCl_3$, 400 MHz) δ = 7.57 (2H, d, *J* = 6.9 Hz, *ortho* Ph), 7.40–7.28 (8H, m, Ph), 5.67 (1H, ddd, *J* = 17.2, 10.0, 8.8 Hz, C5H), 5.28–5.24 (1H, m, C7H), 4.97–4.89 (2 × 1H, 2 × dd, broad unresolved, $C4H_AH_B$), 4.48 (1H, d, *J* = 11.9 Hz, $OC\mathit{H_A}H_BPh$), 4.39 (1H, d, *J* = 11.9 Hz, $OCH_A\mathit{H_B}Ph$), 3.48 (3H, s, OCH_3), 3.26 (2H, d, *J* = 6.1 Hz, $C9H_AH_B$), 2.55–2.45 (1H, m, C6H), 2.19–2.11 (1H, m, C8H), 0.99 (3H, d, *J* = 6.9 Hz, $C25H_3$), 0.93 (3H, d, *J* = 6.9 Hz, $C24H_3$); **^{13}C NMR** δ = ($CDCl_3$, 100 MHz) 166.1 (C=O), 139.7 (C5H), 138.3 (*ipso* benzyl Ph), 132.0 (Ph), 129.5 (2C, Ph), 128.4 (2C, *ortho* benzyl Ph), 128.3 (2C, Ph), 127.9 (*para* benzyl Ph), 127.8 (Ph), 127.6 (2C, *meta* benzyl Ph), 123.5 (q, *J* = 288.4 Hz, $\mathit{C}F_3$) 116.0 ($C4H_2$), 84.7 (q, *J* = 27.8 Hz, $\mathit{C}CF_3$), 79.9 (C7H), 73.3 ($O\mathit{C}H_2Ph$), 72.4 ($C9H_2$), 55.3 (OCH_3), 40.7 (C6H), 35.2 (C8H), 17.3 ($C25H_3$), 11.5 ($C24H_3$); **^{19}F** ($CDCl_3$, 400 MHz) δ = –71.24; **HRMS** (+ESI) Found $[M+H]^+$ = 451.2091; $C_{25}H_{30}O_4F_3$ requires 451.2091, Δ 0.00 ppm.

† The minor diastereomer obtained from **22** could not be removed from the reaction at this stage.

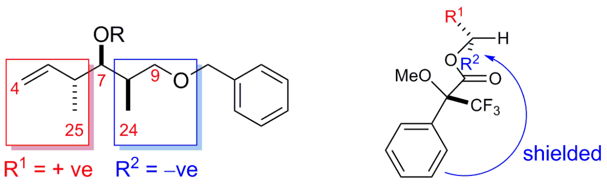

R = MTPA

H	δ (*S*)-ester S7 (ppm)[a]	δ (*R*)-ester S8 (ppm)[a]	Δδ (δ_S-δ_R)-ester × 400 (Hz)
C5H	5.73	5.67	+24
C4H	4.98	4.93	+20
C6H	2.53	2.50	+12
C25H_3	1.02	0.99	+12
C7H	5.28	5.26	+8
C8H	2.135	2.15	–6
C24H_3	0.87	0.93	–24
C9H_2	3.19	3.26	–28

[a]For chemical shifts quoted as a multiplet in the above procedures, the midpoint of the range has been taken.

Based on the model proposed by Mosher,[6] the alcohol was assigned the **(*R*)-configuration**.

5-*O*-Benzyl-2,4-dideoxy-2,4-dimethyl-D-lyxose S9

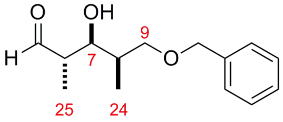

To a solution of **22** (3.40 g, 14.5 mmol) in acetone (242 mL) and H_2O (121 mL) (2:1) at RT was added NMO (2.30 g, 19.6 mmol) and OsO_4 [2.5% (w/w) in *t*BuOH (9.20 mL, 0.725 mmol]. The reaction mixture was stirred for 16 h after which it was quenched with sodium sulfite (5.0 g) and stirred for a further 2 h. The mixture was diluted with H_2O (150 mL) and the layers separated. The aqueous layer was further extracted with CH_2Cl_2 (2 × 150 mL) and the combined organic layers dried ($MgSO_4$) and concentrated *in vacuo*. The isolated triol (3.89 g, 14.5 mmol) was used immediately in the next step of the reaction without further purification.

To a solution of the crude triol (3.89 g, 14.5 mmol) in THF (153 mL) and H_2O (15.3 mL) (10:1) at 0 °C was added $NaIO_4$ (9.30 g, 43.5 mmol). The reaction mixture was warmed to RT over 45 min and then diluted with Et_2O (100 mL) and H_2O (100 mL). The layers were separated and the aqueous layer further extracted with Et_2O (2 × 100 mL). The combined organic layers were washed with H_2O (300 mL) and brine (300 mL) before being dried ($MgSO_4$) and concentrated *in vacuo* to give the title compound **S9**. The relatively clean (> 90% by ^{1}H NMR) aldehyde (3.43 g, quant.) was carried through to the next step in the synthesis without further purification.

***R*$_f$** = 0.19 [petroleum ether (40–60):Et_2O, 7:3]; $[\alpha]_D^{25.0}$ = +1.4 (*c* = 0.68, $CHCl_3$); **IR** (film) ν_{max}/cm^{-1} 3452, 2972, 2940, 2877, 1721, 1455, 1381, 1366, 1195, 1148, 1097, 1031; **^{1}H NMR** ($CDCl_3$, 400 MHz) δ = 9.79 (1H, d, *J* = 2.2 Hz, C5HO), 7.38–7.30 (5H, m, Ph), 4.52 (2H, d, *J* = 1.8 Hz, OC*H*$_2$Ph), 4.01 (1H, dd, *J* = 8.9, 2.3 Hz, C7H), 3.59–3.55 (2 × 1H, 2 × dd, broad unresolved, C9H_AH_B), 2.53–2.49 (1H, m, C6H), 1.93–1.89 (1H, m, C8H), 1.04 (3H, d, *J* = 7.2 Hz, C25H_3), 0.99 (3H, d, *J* = 7.1 Hz, C24H_3); **^{13}C NMR** ($CDCl_3$, 100 MHz) δ = 205.7 (C5HO), 137.9 (*ipso* Ph), 128.6 (2C, *ortho* Ph), 127.8 (*para* Ph), 127.7 (2C, *meta* Ph), 74.7 (C9H_2), 74.3 (C7H), 73.5 (O*C*H_2Ph), 49.4 (C6H), 35.1 (C8H), 10.7 (C25H_3), 9.8 (C24H_3); **HRMS** (+ESI) Found $[M+Na]^+$ = 259.1306; $C_{14}H_{20}O_3Na$ requires 259.1305, Δ 0.39 ppm.

(2*R*,3*S*,4*R*,5*S*)-1-(Benzyloxy)-2,4-dimethyloct-7-yne-3,5-diol 23

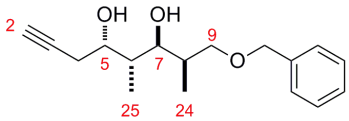

To a suspension of freshly activated Zn† (5.49 g, 84.0 mmol) in THF (43 mL) at 0 °C was added propargyl bromide (80% in PhMe, 12.67 mL, 117.54 mmol) dropwise. The mixture was cooled to –100 °C and freshly prepared **S9** (3.96 g, 16.8 mmol) was added dropwise *via* cannula as a solution in THF (43 mL), ensuring that the internal temperature did not rise above –95 °C. The reaction was maintained at –100 °C for 2 h and then diluted with 0.1 *N* HCl (800 mL) and

† Zn activated by stirring in 1 *N* HCl for 1 h, washing with H_2O (2 × 100 mL) and dried under vacuum at 150 °C. The resulting powder was then ground using a mortar and pestle.

allowed to warm to RT. The layers were separated and the aqueous layer extracted with EtOAc (2 × 1 L), dried ($MgSO_4$) and concentrated *in vacuo*. Purification by column chromatography (SiO_2, hexane:EtOAc, 7:3) gave the title compound **23** (2.90 g, 72% over 3 steps, dr = 85:15) as an inseparable mixture of diastereomers.[7]

Diastereomeric ratio ascertained by ^{1}H NMR spectroscopy of the crude mixture; δ_H 0.84 (3H, d, J = 7.1 Hz, C25H_3 major), 0.80 (3H, d, J = 6.9 Hz, C25H_3, minor).

$\boldsymbol{R_f}$ = 0.20 [petroleum ether (40–60):Et_2O, 1:1]; $[\alpha]_D^{25.0}$ = –7.7 (c = 0.73, $CHCl_3$); **IR** (film) ν_{max}/cm^{-1} 3397, 3296, 2964, 2924, 2873, 1455, 1383, 1366, 1255, 1207, 1102; **^{1}H NMR** ($CDCl_3$, 500 MHz) δ = 7.36–7.28 (5H, m, Ph), 4.52 (1H, d, J = 12.0 Hz, OCH_AH_BPh), 4.48 (1H, d, J = 12.0 Hz, OCH_AH_BPh), 4.07 (1H, ddd, J = 8.0, 5.5, 2.4 Hz, C5H), 3.82 (1H, dd, J = 8.8, 2.6 Hz, C7H), 3.60 (1H, dd, J = 9.0, 3.9 Hz, C9H_AH_B), 3.54 (1H, dd, J = 9.0, 4.6 Hz, C9H_AH_B), 2.49 (1H, ddd, J = 17.0, 8.5, 2.7 Hz, C4H_AH_B), 2.35 (1H, ddd, J = 16.5, 5.3, 2.7 Hz, C4H_AH_B), 2.00 (1H, t, J = 2.7 Hz, C2H), 1.96–1.90 (1H, m, C6H), 1.89–1.83 (1H, m, C8H), 1.02 (3H, d, J = 7.1 Hz, C24H_3), 0.84 (3H, d, J = 7.1 Hz, C25H_3); **^{13}C NMR** ($CDCl_3$, 126 MHz) δ = 137.8 (*ipso* Ph), 128.5 (2C, *ortho* Ph), 127.8 (*para* Ph), 127.6 (2C, *meta* Ph), 82.0 (C3) 76.6 (C7H), 75.6 (C9H_2), 73.5 (O*C*H_2Ph), 72.2 (C5H), 69.9 (C2H), 38.9 (C6H), 35.3 (C8H), 23.5 (C4H_2), 11.7 (C25H_3), 10.0 (C24H_3); **HRMS** (+ESI) Found $[M+Na]^+$ = 299.1610; $C_{17}H_{24}O_3Na$ requires 299.1618, Δ 2.67 ppm.

(5*S*)-1-*O*-Benzyl-2,4-dideoxy-2,4-dimethyl-3,5-*O*-(1-methylethylidene)-5-*C*-prop-2-yn-1-yl-D-arabinitol S10

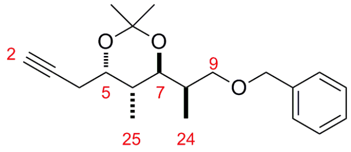

To a solution of **23** (1.89 g, 6.85 mmol) in acetone (33 mL) at RT was added 2,2-dimethoxypropane (6.74 mL, 54.8 mmol) and (±)-CSA (0.16 g, 0.685 mmol). The reaction mixture was stirred for 1 h and then diluted with sat. aq. $NaHCO_3$ (20 mL). The layers were

separated and the aqueous layer further extracted with Et_2O (4 × 30 mL), dried ($MgSO_4$) and concentrated *in vacuo* to give a yellow oil. The crude title compound **S10** (assumed quant.) was used directly.

A small amount of the title compound **S10** was purified by column chromatography [SiO_2, petroleum ether (40–60):Et_2O, 9:1] for full characterisation as a colourless oil.

***R*f** = 0.29 [petroleum ether (40:60):Et_2O, 9:1]; $[\alpha]_D^{25.0}$ = –2.0 (*c* = 0.30, $CHCl_3$); **IR** (film) ν_{max}/cm^{-1} 3302, 2980, 2918, 2873, 1455, 1382, 1364, 1227, 1201, 1173, 1140, 1097, 1060, 1022; **^{1}H NMR** ($CDCl_3$, 500 MHz) δ = 7.33–7.26 (5H, m, Ph), 4.51 (1H, d, *J* = 11.7 Hz, OCH_AH$_B$Ph), 4.48 (1H, d, *J* = 12.0 Hz, OCH$_A$$H_B$Ph), 3.96 (1H, td, *J* = 7.2, 4.8 Hz, C5H), 3.48–3.46 (1H, m, C7H), 3.45–3.41 (1H, dd, *J* = 7.7, 3.9 Hz, C9H_AH$_B$), 3.33 (1H, dd, *J* = 9.1, 5.9 Hz, C9H$_A$$H_B$), 2.35 (1H, dd, *J* = 7.1, 2.6 Hz, C4H_AH$_B$), 2.26 (1H, dd, *J* = 8.1, 2.7 Hz, C4H$_A$$H_B$), 2.06–1.98 (1H, m, C6H), 1.95 (1H, t, *J* = 2.6 Hz, C2H), 1.92–1.88 (1H, m, C8H), 1.30 (3H, s, acetonide CH_3), 1.28 (3H, s, acetonide CH_3), 0.93 (3H, d, *J* = 6.9 Hz, C24H_3), 0.86 (3H, d, *J* = 6.8 Hz, C25H_3); **^{13}C NMR** ($CDCl_3$, 126 MHz) δ = 138.6 (*ipso* Ph), 128.3 (2C, *ortho* Ph), 127.6 (*para* Ph), 127.4 (2C, *meta* Ph), 100.6 (*C*(CH_3)$_2$, *trans* acetonide), 81.0 (C3), 73.8 (C7H), 73.1 (O*C*H$_2$Ph), 72.8 (C9H$_2$), 69.3 (C2H), 68.2 (C5H), 36.3 (C8H), 35.4 (C6H), 25.1 (one of C(*C*H$_3$)$_2$ *trans* acetonide), 23.5 (one of C(*C*H$_3$)$_2$ *trans* acetonide), 21.0 (C4H$_2$), 11.4 (C25H$_3$), 11.1 (C24H$_3$); **HRMS** (+ESI) Found $[M+Na]^+$ = 339.1937; $C_{20}H_{28}O_3Na$ requires 339.1931, Δ 1.77 ppm.

(5*S*)-1-*O*-Benzyl-2,4-dideoxy-5-*C*-(4-methoxy-4-oxobut-2-yn-1-yl)-2,4-dimethyl-3,5-*O*-(1-methylethylidene)-D-arabinitol 24

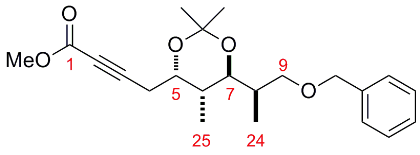

To a solution of **S10** (assumed quant., 6.85 mmol) in THF (68 mL) at –40 °C was added *n*BuLi (2.08 M in hexanes, 3.95 mL, 8.22 mmol) dropwise. The resulting yellow solution was cooled to

–78 °C and stirred for 15 min after which methyl chloroformate (1.59 mL, 20.6 mmol) was added dropwise. The now colourless solution was stirred at –78 °C for 30 min and then warmed to RT slowly over 90 mins. The reaction mixture was quenched with sat. aq. NH_4Cl (50 mL) and diluted with Et_2O (50 mL). The layers were separated and the aqueous layer further extracted with Et_2O (3 × 75 mL). The combined organic layers were dried ($MgSO_4$) and concentrated *in vacuo*. Purification by column chromatography [SiO_2, petroleum ether (40–60):Et_2O:Et_3N, 9:0.9:0.1] gave the title compound **24** (1.87 g, 73% over 2 steps) as a white solid and as a single diastereomer.

***R*f** = 0.62 [petroleum ether (40–60):Et_2O, 7:3]; **m.p.** = 60–63 °C; $[\alpha]_D^{25.0}$ = –3.8 (*c* = 0.79, $CHCl_3$); **IR** (film) ν_{max}/cm^{-1} 2984, 2936, 2873, 2241, 1715, 1455, 1435, 1382, 1362, 1252, 1226, 1171, 1141, 1088, 1072, 1057, 1019; **^{1}H NMR** ($CDCl_3$, 500 MHz) δ = 7.35–7.25 (5H, m, Ph), 4.49 (1H, d, *J* = 12.1 Hz, OCH_AH_BPh), 4.47 (1H, d, *J* = 12.0 Hz, OCH_AH_BPh), 4.01 (1H, td, *J* = 7.5, 4.9 Hz, C5H), 3.75 (3H, s, $C1O_2CH_3$), 3.46 (1H, dd, *J* = 8.1, 2.9 Hz, C7H), 3.42 (1H, dd, *J* = 9.0, 7.8 Hz, $C9H_AH_B$), 3.32 (1H, dd, *J* = 9.0, 5.8 Hz, $C9H_AH_B$), 2.49 (1H, dd, *J* = 17.0, 7.3 Hz, $C4H_AH_B$), 2.39 (1H, dd, *J* = 17.0, 7.7 Hz, $C4H_AH_B$), 1.99–1.95 (1H, m, C6H), 1.90–1.86 (1H, m, C8H), 1.30 (3H, s, acetonide CH_3), 1.27 (3H, s, acetonide CH_3), 0.92 (3H, d, *J* = 6.9 Hz, $C24H_3$), 0.85 (3H, d, *J* = 6.8 Hz, $C25H_3$); **^{13}C NMR** ($CDCl_3$, 126 MHz) δ = 154.0 ($C1O_2CH_3$), 138.6 (*ipso* Ph), 128.3 (2C, *ortho* Ph), 127.6 (*para* Ph), 127.5 (2C, *meta* Ph), 100.9 ($C(CH_3)_2$, *trans* acetonide), 86.2 (C3), 73.7 (C7H), 73.6 (C2), 73.1 (OCH_2Ph), 72.7 ($C9H_2$), 67.6 (C5H), 52.6 ($C1O_2CH_3$), 36.2 (C6H), 35.6 (C8H), 25.0 (one of $C(CH_3)_2$ *trans* acetonide), 23.3 (one of $C(CH_3)_2$ *trans* acetonide), 21.3 ($C4H_2$), 11.5 ($C25H_3$), 11.0 ($C24H_3$); **HRMS** (+ESI) Found $[M+H]^+$ = 375.2176; $C_{22}H_{31}O_5$ requires 375.2171, Δ 1.33 ppm; **Elemental Analysis** found C, 70.60; H, 8.17. $C_{22}H_{30}O_5$ requires C, 70.56; H, 8.07%.

The structure and relative stereochemistry was confirmed by X-ray crystallographic analysis after crystallisation from analytical grade EtOH.

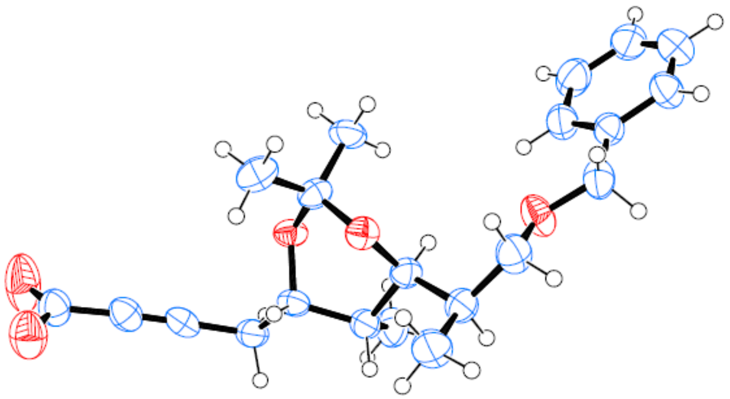

CCDC 882400 contains the supplementary crystallographic data for this thesis. This data can be obtained free of charge from the Cambridge Crystallographic Data Centre *via* www.ccdc.cam.ac.uk/data_request/cif.

Methyl (5*S*,6*R*,7*S*,8*R*)-9-(benzyloxy)-5,7-dihydroxy-6,8-dimethylnon-2-ynoate 25

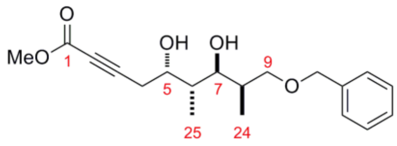

To a solution of **24** (733 mg, 1.96 mmol) in MeOH (24 mL) at RT was added QP-SA resin (1.12 g, 3.5 mmol/g, 3.92 mmol). The reaction was stirred at RT until TLC analysis indicated that the starting material had been consumed. The QP-SA resin was removed by filtration and the reaction mixture concentrated *in vacuo*. Purification by column chromatography [SiO_2, petroleum ether (40–60):Et_2O, 1:1] gave the title compound **25** (619 mg, 95%) as a colourless oil.

$\boldsymbol{R_f}$ = 0.26 [petroleum ether (40–60):Et_2O, 1:1]; $[\alpha]_D^{25.0}$ = –9.8 (*c* = 0.56, $CHCl_3$); **IR** (film) ν_{max}/cm^{-1} 3409, 2918, 2964, 2861, 2240, 1714, 1453, 1436, 1363, 1258, 1102, 1074, 1029; **^{1}H NMR** ($CDCl_3$, 500 MHz) δ = 7.37–7.27 (5H, m, Ph), 4.53 (1H, d, *J* = 11.9 Hz, OCH_AH_BPh), 4.48 (1H, d, *J* = 11.9 Hz, OCH_AH_BPh), 4.08 (1H, ddd, *J* = 7.8, 5.4, 2.2 Hz, C5H), 3.83 (1H, dd, *J* = 9.0, 2.3 Hz, C7H), 3.73 (3H, s, $C1O_2CH_3$), 3.62 (1H, dd, *J* = 9.0, 3.5 Hz, $C9H_AH_B$), 3.54 (1H, dd, *J* = 9.0, 4.4 Hz, $C9H_AH_B$), 3.07–2.93 (2H, br s, 2 × OH), 2.63 (1H, dd, *J* = 17.1, 8.1 Hz, $C4H_AH_B$), 2.51 (1H, dd, *J* = 17.1, 5.4 Hz, $C4H_AH_B$), 1.98–1.94 (1H, m, C6H), 1.87–1.83 (1H, m, C8H), 1.02 (3H, d, *J* = 7.1 Hz, $C24H_3$), 0.84 (3H, d, *J* = 7.1 Hz, $C25H_3$); **^{13}C NMR** ($CDCl_3$, 126 MHz) δ = 154.1 ($C1O_2CH_3$), 137.7 (*ipso* Ph), 128.5 (2C, *ortho* Ph), 127.9 (*para* Ph), 127.6 (2C, *meta* Ph), 87.6 (C3), 77.2 (C7H), 75.8 ($C9H_2$), 74.0 (C2), 73.6 (OCH_2Ph), 72.2 (C5H), 52.6 ($C1O_2CH_3$), 39.2 (C6H), 35.2 (C8H), 23.6 ($C4H_2$), 12.2 ($C25H_3$), 9.9 ($C24H_3$); **HRMS** (+ESI) Found $[M+H]^+$ = 335.1873; $C_{19}H_{27}O_5$ requires 335.1858, Δ 4.48 ppm.

Dimethyl 9-*O*-benzyl-2,4,6,8-tetradeoxy-6,8-dimethyl-β-D-*galacto*-non-3-ulopyranosidonate 26

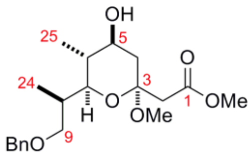

To substrate **25** (1.42 g, 4.25 mmol) was added $AuCl_3$ (25.8 mg, 85 μmol) in MeOH (0.01 M, 8.5 mL) at RT. After 45 min the reaction mixture was diluted with EtOAc and petroleum ether (30–40) (1:1, 100 mL), and quenched with sat. aq. $NaHCO_3$ (100 mL). The layers were separated and the organic layer washed with H_2O (2 × 50 mL) and brine (2 × 50 mL) before being dried ($MgSO_4$) and concentrated *in vacuo*. The residue was filtered through a pad of Celite® under suction using EtOAc (3 × 30 mL), and concentrated *in vacuo* to give the title compound **26** (1.49 g, 96%) as a colourless oil.

***R*f** = 0.33 (hexane:EtOAc, 1:1); $[\alpha]_D^{25.0}$ = –48.1 (*c* = 0.59, $CHCl_3$); **IR** (film) ν_{max}/cm^{-1} 3466, 2968, 2932, 2885, 1738, 1454, 1438, 1378, 1362, 1313, 1221, 1093, 1072, 1054, 1017; **^{1}H NMR** ($CDCl_3$, 500 MHz) δ = 7.34–7.22 (5H, m, Ph), 4.48 (1H, d, *J* = 11.9 Hz, OCH_AH_BPh), 4.43 (1H, d, *J* = 11.9 Hz, OCH_AH_BPh), 3.65 (3H, s, $C1O_2CH_3$), 3.66–3.62 (1H, m, C5H), 3.54 (1H, dd, *J* = 8.7, 8.6 Hz, $C9H_AH_B$), 3.48 (1H, dd, *J* = 10.9, 1.3 Hz, C7H), 3.32 (1H, dd, *J* = 8.7, 6.2 Hz, $C9H_AH_B$), 3.14 (3H, s, $C3OCH_3$), 2.66 (1H, d, *J* = 13.7 Hz, $C2H_AH_B$), 2.58 (1H, d, *J* = 13.6 Hz, $C2H_AH_B$), 2.23 (1H, dd, *J* = 12.7, 4.7 Hz, equatorial $C4H_AH_B$), 2.11–2.07 (1H, m, C8H), 1.63 (1H, dd, *J* = 12.3, 11.5 Hz, axial $C4H_AH_B$), 1.43–1.39 (1H, m, C6H), 1.39 (1H, d, *J* = 5.6 Hz, C5HO*H*), 0.92 (3H, d, *J* = 6.5 Hz, $C25H_3$), 0.81 (3H, d, *J* = 6.9 Hz, $C24H_3$); **^{13}C NMR** ($CDCl_3$, 126 MHz) δ = 169.7 ($C1O_2CH_3$), 138.4 (*ipso* Ph), 128.3 (2C, *ortho* Ph), 127.6 (*para* Ph), 127.5 (2C, *meta* Ph), 98.7 (C3), 73.1 ($C9H_2$), 73.0 (OCH_2Ph), 72.7 (C7H), 70.0 ($C1O_2CH_3$), 51.7 (C5H), 47.8 ($C3OCH_3$), 42.7 ($C4H_2$), 41.7 ($C2H_2$), 39.6 (C6H), 33.7 (C8H), 12.0 ($C25H_3$), 9.3 ($C24H_3$); **HRMS** (+ESI) Found $[M+Na]^+$ = 389.1948; $C_{20}H_{30}O_6Na$ requires 389.1940, Δ 2.06 ppm; **Elemental Analysis** found C, 66.05; H, 8.29. $C_{20}H_{30}O_6$ requires C, 65.55; H, 8.25%.

Dimethyl 9-*O*-benzyl-5-*O*-(*tert*-butyl(dimethyl)silyl)-2,4,6,8-tetradeoxy-6,8-dimethyl-β-D-*galacto*-non-3-ulopyranosidonate 20a

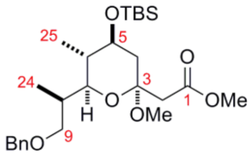

Procedure 1

To a solution of **26** (1.49 g, 4.07 mmol) in CH_2Cl_2 (41 mL) at –78 °C was added 2,6-lutidine (1.04 mL, 8.95 mmol) and TBSOTf (1.03 mL, 4.48 mmol) sequentially. The reaction mixture was stirred for 2 h at –78 °C and then allowed to warm to 0 °C after which CH_2Cl_2 (120 mL) and H_2O (120 mL) were added. The layers were separated and the aqueous layer further extracted with CH_2Cl_2 (4 × 100 mL). The combined organic layers were washed sequentially with a cooled solution (0 °C) of 1 *N* HCl (150 mL), sat. aq. $NaHCO_3$ (150 mL), H_2O (150 mL) and brine (150 mL) before being dried ($MgSO_4$) and concentrated *in vacuo*. Purification by column chromatography [SiO_2, hexane→hexane:Et_2O, 1:1] gave the title compound **20a** (1.78 g, 91%) as a colourless oil.

Procedure 2

To a solution of **19b** (226 mg, 0.50 mmol) in CH_2Cl_2 (11 mL) was added MeOH (500 μL) and (±)-CSA (12 mg, 0.05 mmol) sequentially at RT. The mixture was stirred for 2 h, after which the reaction was quenched by the addition of sat. aq. $NaHCO_3$ (10 mL). The phases were separated and the aqueous layer extracted with CH_2Cl_2 (3 × 10 mL). The combined organic layers were dried ($MgSO_4$) and concentrated *in vacuo*. Purification by column chromatography [SiO_2, petroleum ether (40–60):Et_2O, 95:5→9:1] gave the title compound **20a** (178 mg, 74%) as a colourless oil.[†]

[†] Despite performing this reaction under identical reaction conditions, this procedure was found to be unreliable, giving yields ranging between 20–74%.

R_f = 0.42 [petroleum ether (40–60):Et_2O, 8:2]; $[\alpha]_D^{25.0}$ = –31.5 (c = 0.83, $CHCl_3$); **IR** (film) ν_{max}/cm^{-1} 2964, 2930, 2856, 1743, 1455, 1437, 1378, 1361, 1315, 1250, 1220, 1069, 1029, 1005; **^{1}H NMR** ($CDCl_3$, 500 MHz) δ = 7.37–7.29 (4H, m, Ph), 7.28–7.26 (1H, m, Ph), 4.50 (1H, d, J = 11.9 Hz, OCH_AH_BPh), 4.46 (1H, d, J = 11.9 Hz, OCH_AH_BPh), 3.67 (3H, s, $C1O_2CH_3$), 3.64 (1H, m, C5H), 3.56 (1H, dd, J = 8.6, 8.5 Hz, $C9H_AH_B$), 3.48 (1H, dd, J = 10.6, 1.6 Hz, C7H), 3.34 (1H, dd, J = 8.8, 6.2 Hz, $C9H_AH_B$), 3.17 (3H, s, $C3OCH_3$), 2.62 (1H, d, J = 13.6 Hz, $C2H_AH_B$), 2.58 (1H, d, J = 13.5 Hz, $C2H_AH_B$), 2.14–2.07 (2H, m, equatorial C4H and C8H), 1.67 (1H, dd, J = 12.8, 10.9 Hz, axial C4H), 1.48–1.39 (1H, m, C6H), 0.89 (9H, s, $C(CH_3)_3$ of *t*Bu), 0.87 (3H, d, J = 6.6 Hz, $C25H_3$), 0.84 (3H, d, J = 6.9 Hz, $C24H_3$), 0.06 (6H, s, $Si(CH_3)_2$); **^{13}C NMR** ($CDCl_3$, 126 MHz) δ = 169.8 ($C1O_2CH_3$), 138.5 (*ipso* Ph), 128.3 (2C, *ortho* Ph), 127.6 (*para* Ph), 127.5 (2C, *meta* Ph), 98.8 (C3), 73.2 ($C9H_2$), 73.0 (OCH_2Ph), 72.8 (C7H), 70.6 ($C1O_2CH_3$), 51.6 (C5H), 47.6 ($C3OCH_3$), 43.1 ($C4H_2$), 41.8 ($C2H_2$), 39.8 (C6H), 33.9 (C8H), 25.8 (3C, $C(CH_3)_3$ of *t*Bu), 18.0 ($C(CH_3)_3$ of *t*Bu), 12.4 ($C25H_3$), 9.3 ($C24H_3$), –4.1 ($Si(CH_3)_2$), –4.7 ($Si(CH_3)_2$); **HRMS** (+ESI) Found $[M+Na]^+$ = 503.2805; $C_{26}H_{44}O_6SiNa$ requires 503.2806, Δ 0.20 ppm.

Dimethyl 2,4,6,8-tetradeoxy-6,8-dimethyl-β-D-*galacto*-non-3-ulopyranosidonate S11

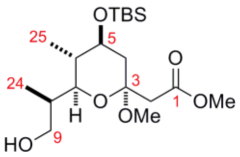

Procedure 1

To a solution of **20b** (642 mg, 1.34 mmol) in EtOAc (40 mL) at RT was added 10% Pd/C (164 mg). The resulting suspension was evacuated and back filled with hydrogen (× 3) and left to stir under a hydrogen atmosphere for 48 h. The mixture was then filtered through a pad of Celite® under suction using PhMe (3 × 50 mL) and concentrated *in vacuo* to give the title compound **S11** (502 mg, 96%) as a colourless oil.[9]

Procedure 2

To a solution of **20a** (113 mg, 0.224 mmol) in THF (5 mL) at RT was added TBAF (1.0 M in THF, 225 µL, 0.225 mmol) dropwise. After 1 h, TLC showed partial conversion to a new product. A further equivalent of TBAF (225 µL, 0.225 mmol) was added and the reaction mixture stirred for an additional 1 h. The reaction was quenched by the addition of sat. aq. $NaHCO_3$ (10 mL) and the phases separated. The aqueous layer was extracted with Et_2O (3 × 10 mL) and the combined organic layers dried ($MgSO_4$) and concentrated *in vacuo*. Purification by column chromatography [SiO_2, petroleum ether (40–60):Et_2O, 9:1→6:4] gave the title compound **S11** (58 mg, 67%) as a colourless oil as well as starting unreacted **20a** (17 mg, 11%).

R$_{\mathbf{f}}$ = 0.10 (hexane:Et_2O, 3:2); $[\alpha]_D^{25.0}$ = –39.4 (*c* = 1.12, $CHCl_3$), [lit.[7] $[\alpha]_D^{25}$ –38 (*c* = 1.0, $CHCl_3$)]; **IR** (film) ν_{max}/cm^{-1} 3479, 2956, 2931, 2858, 1743, 1463, 1438, 1378, 1362, 1315, 1250, 1223, 1073, 1033, 1003; **^{1}H NMR** ($CDCl_3$, 500 MHz) δ = 3.72 (1H, d, *J* = 10.7, 3.6 Hz, C9H_AH_B), 3.66 (3H, s, C1O_2CH_3), 3.67–3.58 (2H, m, C5H and C9H_AH_B), 3.48 (1H, dd, *J* = 10.6, 2.3 Hz, C7H), 3.20 (3H, s, C3OCH_3), 2.67 (1H, d, *J* = 13.4 Hz, C2H_AH_B), 2.54 (1H, d, *J* = 13.4 Hz, C2H_AH_B), 2.33 (1H, br s, C9H_AH_BO*H*), 2.07 (1H, dd, *J* = 13.0, 4.7 Hz, equatorial C4H_AH_B), 1.88–1.84 (1H, m, C8H), 1.68 (1H, dd, *J* = 12.8, 13.0 Hz, axial C4H_AH_B), 1.46–1.39 (1H, m, C6H), 0.91 (3H, d, *J* = 7.1 Hz, C25H_3), 0.86 (9H, s, C(CH_3)$_3$ of *t*Bu), 0.84 (3H, d, *J* = 6.6 Hz, C24H_3), 0.04 (6H, s, Si(CH_3)$_2$); **^{13}C NMR** ($CDCl_3$, 126 MHz) δ = 169.6 (*C*1O_2CH_3), 99.2 (C3), 77.2 (C7H), 70.2 (C5H), 67.4 (C9H_2), 51.7 (C1O_2*C*H_3), 47.8 (C3O*C*H_3), 42.9 (C4H_2), 41.8 (C2H_2), 39.9 (C6H), 35.2 (C8H), 25.8 (3C, C(*C*H_3)$_3$ of *t*Bu), 18.0 (*C*(CH_3)$_3$ of *t*Bu), 12.4 (C24H_3), 8.8 (C25H_3), –4.1 (Si(*C*H_3)$_2$), –4.8 (Si(*C*H_3)$_2$); **HRMS** (+ESI) Found $[M+Na]^+$ = 413.2335; $C_{19}H_{38}O_6SiNa$ requires 413.2329, Δ 1.45 ppm.

Dimethyl (7*S*)-2,4,6,8-tetradeoxy-2,4-dimethyl-L-*galacto*-non-7-ulopyranosiduronate 5[7]

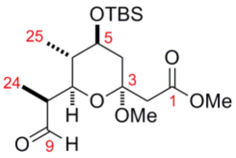

To a solution of **S11** (652 mg, 1.67 mmol) in CH_2Cl_2 (84 mL) at RT was added K_2CO_3 (3.23 g, 23.4 mmol) and freshly prepared Dess–Martin periodinane[8] (2.12 g, 5.00 mmol) sequentially. The cloudy reaction mixture was stirred for 90 min after which hexane (100 mL) was added, resulting in precipitation. The solids were removed by filtration and the liquor partially concentrated *in vacuo* before being re-filtered and concentrated to dryness. Purification by column chromatography (SiO_2, hexane:Et_2O, 4:1) gave the title compound **5** (564 mg, 87%) as a colourless oil.

$\boldsymbol{R_f}$ = 0.16 (hexane:Et_2O, 4:1); $[\alpha]_D^{27.6}$ = –21.9 (c = 0.95, $CHCl_3$); **IR** (film) ν_{max}/cm^{-1} 2952, 2932, 2858, 1736, 1463, 1438, 1377, 1360, 1318, 1251, 1221, 1148, 1073, 1031, 1004; **^{1}H NMR** ($CDCl_3$, 400 MHz) δ = 9.71 (1H, s, C9HO), 3.89 (1H, dd, J = 10.6, 2.3 Hz, C7H), 3.74–3.67 (1H, m, C5H), 3.66 (3H, s, C1O_2CH_3), 3.16 (3H, s, C3OCH_3), 2.62 (1H, d, J = 13.9 Hz, C2H_AH_B), 2.54 (1H, d, J = 13.9 Hz, C2H_AH_B), 2.50 (1H, qd, J = 6.9, 2.3 Hz, C8H), 2.16 (1H, dd, J = 13.0, 4.7 Hz, equatorial C4H_AH_B), 1.67 (1H, dd, J = 13.0, 10.9 Hz, axial C4H_AH_B), 1.53–1.46 (1H, m, C6H), 1.12 (3H, d, J = 7.0 Hz, C24H_3), 0.91 (3H, d, J = 6.7 Hz, C25H_3), 0.89 (9H, s, C($CH_3)_3$ of *t*Bu), 0.07 (6H, s, Si($CH_3)_2$); **^{13}C NMR** ($CDCl_3$, 100 MHz) δ = 204.2 (C9HO), 169.5 (C1O_2CH_3), 99.3 (C3), 73.2 (C7H), 70.1 (C5H), 51.7 (C1O_2CH_3), 48.1 (C3OCH_3), 47.2 (C8H), 43.0 (C4H_2), 41.5 (C2H_2), 39.6 (C6H), 25.7 (3C, C($CH_3)_3$ of *t*Bu), 18.0 ($C(CH_3)_3$ of *t*Bu), 12.6 (C24H_3), 6.2 (C25H_3), –4.0 (Si($CH_3)_2$), –4.8 (Si($CH_3)_2$); **HRMS** (+ESI) Found $[M+Na]^+$ = 411.2194; $C_{19}H_{36}O_6SiNa$ requires 411.2179, Δ 3.65 ppm; **Elemental Analysis** found C, 58.81; H, 9.19. $C_{19}H_{36}O_6Si$ requires C, 58.73; H, 9.34%.

5-Iodo-4-methyl-pent-4-en-l-ol S12[9]

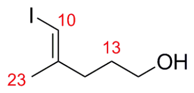

To a slurry of Cp_2ZrCl_2 (3.48 g, 11.9 mmol) in CH_2Cl_2 (100 mL) at RT was added Me_3Al (17.2 mL, 179.0 mmol) dropwise. The reaction was stirred for 30 mins at RT and then cooled to –10 °C, at which point a solution of 4-pentyn-1-ol (5.0 g, 59.5 mmol) in CH_2Cl_2 (25 mL) was added dropwise *via* syringe over 10 mins. The mixture was warmed to RT and stirred for 18 h, with the reaction progress monitored by removal of an aliquot and integration of the resulting C10CH_2 methylene group by ^{1}H NMR. When complete, the reaction mixture was cooled to –30 °C and a solution of I_2 (19.6 g, 77.4 mmol) in THF (50 mL) was added dropwise over 1 h. The mixture was allowed to warm to RT slowly over a period of 5 h, after which it was re-cooled to –30 °C and quenched by slowly pouring onto a mixture of hexane (500 mL) and Rochelle's salt (100 mL). The mixture was diluted with Et_2O (500 mL) and the layers separated. The aqueous layer was further extracted with Et_2O (2 × 500 mL) and the combined organics dried ($MgSO_4$) and concentrated *in vacuo*. Purification by column chromatography [SiO_2, petroleum ether (30:40):Et_2O, 9:1] gave the title compound **S12** (10.7 g, 84%) as a pale yellow oil.

R

$\boldsymbol{R_f}$ = 0.25 [petroleum ether (30:40):Et_2O, 9:1]; **IR** (film) ν_{max}/cm^{-1} 3324, 2940, 2874, 1617, 1438, 1376, 1267, 1142, 1059; **^{1}H NMR** ($CDCl_3$, 400 MHz) δ = 5.93 (1H, s, C10H), 3.66 (2H, t, *J* = 6.2 Hz, C14H_2), 2.30 (2H, dd, *J* = 7.7, 7.5 Hz, C12H_2), 1.85 (3H, s, C23H_3), 1.77–1.69 (2H, m, C13H_2); **^{13}C NMR** ($CDCl_3$, 150 MHz) δ = 147.4 (C11), 74.9 (C10H), 62.0 (C14H_2), 35.7 (C12H_2), 30.5 (C13H_2), 23.8 (C23H_3); **HRMS** (+EI) Found $[M]^+$ = 225.9854; $C_6H_{11}IO$ requires 225.9855, Δ 0.44 ppm. All spectroscopic data in agreement with that previously published.[9]

5-Iodo-4-methyl-pent-4-enal 31

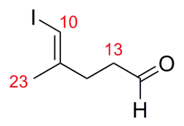

To a stirring solution of oxalyl chloride (3.63 mL, 33 mmol) in CH_2Cl_2 (30 mL) at –78 °C was added DMSO (4.68 mL, 66.0 mmol) dropwise. The reaction mixture was stirred for 10 min at –78 °C and a solution of **S12** (5.00 g, 22.0 mmol) in CH_2Cl_2 (30 mL) was added slowly resulting in the formation of a white precipitate. The mixture was stirred for 20 min at –78 °C at which point Et_3N (15.3 mL, 110.0 mmol) was added dropwise. The suspension was allowed to warm to RT over 30 min after which sat. aq. NH_4Cl (50 mL) was added and the layers separated. The organic layer was washed with sat. aq. NH_4Cl (3 × 20 mL), brine (100 mL), dried ($MgSO_4$), and concentrated *in vacuo* to give the title compound **31** (3.96 g, 80%) as a pale yellow oil.

$\boldsymbol{R_f}$ = 0.13 [petroleum ether (40–60):Et_2O, 9:1]; **IR** (film) ν_{max}/cm^{-1} 2913, 2823, 2724, 1721, 1442, 1376, 1269, 1128, 1069; **^{1}H NMR** ($CDCl_3$, 600 MHz) δ = 9.78 (1H, s, C14HO), 5.98 (1H, s, C10H), 2.60–2.58 (2H, m, C13H_2), 2.55–2.52 (2H, m, C12H_2), 1.86 (3H, s, C23H_3); **^{13}C NMR** ($CDCl_3$, 150 MHz) δ = 200.7 (C14HO), 145.8 (C11), 75.9 (C10H), 41.8 (C13H_2), 31.5 (C12H_2), 24.0 (C23H_3); **HRMS** (+EI) Found $[M]^+$ = 223.9695; C_6H_9IO requires 223.9698, Δ 1.34 ppm.

Ethyl (*R*,2*E*,6*E*)-7-iodo-6-methyl-4-((phenylamino)oxy)hepta-2,6-dienoate 35

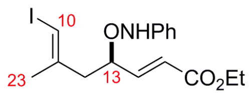

To a solution of **31** (87.0 mg, 0.380 mmol) in DMSO (2 mL) at RT was added L-Proline (7.3 mg, 63.0 μmol) and nitrosobenzene (33.9 mg, 0.316 mmol) sequentially to give a green solution. The initial green solution turned yellow after 20 minutes, and after an additional 40 mins, THF (2 ml) was added, followed by phosphorane **34** (165 mg, 0.474 mmol) in one portion. The reaction was stirred for 3 h at RT, after which sat. aq. NH_4Cl (10 mL) was added and the mixture extracted with Et_2O (2 × 10 mL). The combined organic layers were dried ($MgSO_4$) and concentrated *in*

vacuo. Purification by column chromatography [SiO_2, petroleum ether (40–60):Et_2O, 9:1→8:2] gave the title compound **35** [84.0 mg, 66% (based on PhNO)] as a clear oil.

***R*f** = 0.21 (hexane:EtOAc, 9:1); $[\alpha]_D^{28.7}$ = +22.4 (*c* = 1.0, $CHCl_3$); **IR** (film) ν_{max}/cm^{-1} 3291, 3053, 2980, 2923, 2853, 1713, 1657, 1600, 1493, 1444, 1369, 1304, 1274, 1206, 1165, 1095, 1028, 980; **^{1}H NMR** ($CDCl_3$, 600 MHz) δ = 6.28–6.24 (2H, obscured dd, *meta*-Ph-H), 7.00 (1H, br s, NH), 6.97 (2H, t, *J* = 7.2 Hz, *para*-Ph-H), 6.93–6.87 (3H, m, *ortho*-Ph-H and C14H), 6.12 (1H, s, C10H), 6.06 (1H, d, *J* = 15.8 Hz, C15H), 4.52 (1H, q, *J* = 6.6 Hz, C13H), 4.22 (2H, q, *J* = 7.1 Hz, C$H_2$$CH_3$), 2.27 (1H, dd, *J* = 14.0, 8.0 Hz, C12$H_A$$H_B$), 2.49 (1H, dd, *J* = 14.1, 5.3 Hz, C12$H_A$$H_B$), 1.93 (3H, s, C23$H_3$), 1.31 (3H, t, *J* = 7.1 Hz, CH_2CH_3); **^{13}C NMR** ($CDCl_3$, 150 MHz) δ = 166.3 (*C*$O_2CH_2CH_3$), 148.5 (*ipso* Ph), 146.2 (C14H), 143.4 (C11), 129.5 (2C, *meta* Ph), 123.6 (*para* Ph), 122.8 (C15H), 114.9 (2C, *ortho* Ph), 81.4 (C13H), 79.2 (C10H), 61.1 (*C*H_2CH_3), 43.6 (C12H_2), 24.9 (C23H_3), 14.6 (CH_2*C*H_3); **HRMS** (+ESI) Found $[M+H]^+$ = 402.0578; $C_{16}H_{20}INO_3$ requires 402.0566, Δ 2.90 ppm.

(*R*,2*E*,6*E*)-Ethyl 4-hydroxy-7-iodo-6-methylhepta-2,6-dienoate S13

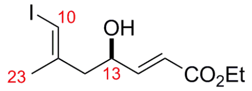

To a solution of **35** (300 mg, 0.747 mmol) in *i*PrOH (2 mL) and THF (0.2 mL) at RT was added $CuSO_4$ (239 mg, 149.0 mmol) in one portion. The reaction was heated to 40°C for 4 days, after which it was quenched by the addition of sat. aq. NH_4Cl (2 mL) and the mixture extracted with Et_2O (3 × 5 mL). The combined organic layers were dried ($MgSO_4$) and concentrated *in vacuo*. Purification by column chromatography [SiO_2, petroleum ether (40–60):Et_2O, 65:35] gave the title compound **S13** (121 mg, 52%) as a colourless oil.

***R*f** = 0.32 (hexane:Et_2O, 1:1); $[\alpha]_D^{25.0}$ = +17.1 (*c* = 0.70, $CHCl_3$); **IR** (film) ν_{max}/cm^{-1} 3442, 2981, 1699, 1657; **^{1}H NMR** ($CDCl_3$, 600 MHz) δ = 6.91 (1H, dd, *J* = 15.6, 4.6 Hz, C14H), 6.10–6.07 (2H, m overlapped, C10H and C15H), 4.46–4.43 (1H, m, C13H), 4.21 (2H, q, *J* = 7.1 Hz, OC$H_2$$CH_3$), 2.51 (1H, dd, *J* = 13.9, 4.1 Hz, C12$H_A$$H_B$), 2.43 (1H, dd, *J* = 13.9, 8.9 Hz,

C12H$_A$*H$_B$*), 1.90 (3H, s, C23H$_3$), 1.29 (3H, t, *J* = 7.1 Hz, OCH$_2$C*H$_3$*); **^{13}C NMR** (CDCl$_3$, 150 MHz) δ = 166.3 (C=O), 148.3 (C14H), 143.4 (C11), 120.9 (C15H), 78.7 (C10H), 68.4 (C13H), 60.5 (O*C*H$_2$CH$_3$), 46.6 (C12H$_2$), 24.0 (*C*23H$_3$), 14.2 (OCH$_2$*C*H$_3$); **HRMS** (+ESI) Found [M+Na]$^+$ = 332.9972; $C_{10}H_{15}INO_3$ requires 332.9958, Δ 4.20 ppm.

(*R*,2*E*,6*E*)-Ethyl 4-(*tert*-butyldimethylsilyloxy)-7-iodo-6-methylhepta-2,6-dienoate 36

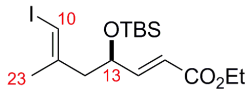

To a solution of **S13** (797 mg, 2.57 mmol) in CH_2Cl_2 (25 mL) at −78 °C was added 2,6-lutidine (1.2 mL, 10.3 mmol) followed by TBSOTf (1.18 mL, 5.14 mmol). The reaction was stirred at –78 °C for 3.5 h after which the mixture quenched with sat. aq. $NaHCO_3$ (15 mL), diluted with CH_2Cl_2 (15 mL) and allowed to warm to RT. The organic layers were separated and the aqueous phase further extracted with CH_2Cl_2 (10 mL). The combined organic layers were washed with 1 M HCl (10 mL), brine (10 mL), dried ($MgSO_4$) and concentrated *in vacuo*. Purification by column chromatography [SiO_2, petroleum ether (40–60):Et_2O, 99:1→9:1] gave the title compound **36** (1.01 g, 93%) as a colourless oil.

***R*$_f$** = 0.31 (hexane:Et_2O, 95:5); $[\alpha]_D^{25.0}$ = +29.0 (*c* = 0.40, $CHCl_3$); **IR** (film) ν_{max}/cm^{-1} 2955, 2930, 2857, 1718, 1660; **^{1}H NMR** (CDCl$_3$, 600 MHz) δ = 6.89 (1H, dd, *J* = 15.5, 4.7 Hz, C14H), 5.99–5.97 (2H, m overlapped, C10H and C15H), 4.39–4.35 (1H, m, C13H), 4.22–4.18 (2H, m, OC*H$_2$*CH$_3$), 2.44–2.37 (2H, m, C12H$_A$H$_B$), 1.86 (3H, s, C23H$_3$), 1.30 (3H, t, *J* = 7.1 Hz, OCH$_2$C*H$_3$*), 0.90 (9H, s, C(C*H$_3$*)$_3$ of *t*Bu), 0.03 (3H, s, Si(C*H$_3$*)$_2$), 0.01 (3H, s, Si(C*H$_3$*)$_2$); **^{13}C NMR** (CDCl$_3$, 150 MHz) δ = 166.5 (C=O), 149.8 (C14H), 143.3 (C11), 120.3 (C15H), 78.7 (C10H), 69.8 (C13H), 60.4 (O*C*H$_2$CH$_3$), 47.3 (C12H$_2$), 25.8 (3C, C(*C*H$_3$)$_3$ of *t*Bu), 24.5 (C23H$_3$), 18.1 (*C*(CH$_3$)$_3$ of *t*Bu), 14.2 (OCH$_2$*C*H$_3$), −4.7 (Si(*C*H$_3$)$_2$), −4.9 (Si(*C*H$_3$)$_2$); **HRMS** (+ESI) Found [M+Na]$^+$ = 447.0828; $C_{16}H_{29}IO_3SiNa$ requires 447.0828, Δ 0.00 ppm.

(*R*,2*E*,6*E*)-4-(*tert*-Butyldimethylsilyloxy)-7-iodo-6-methylhepta-2,6-dien-1-ol 37

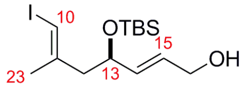

To a stirred solution of **36** (1.01 g, 2.39 mmol) in THF (25 mL) at −78 °C was added DIBAL (1 M in hexanes; 5.98 mL, 5.98 mmol), and the reaction was stirred for 3.5 h whilst allowing to warm to 0 °C. The reaction was quenched with Rochelle's salt (15 mL), diluted with Et_2O (40 mL) and stirred overnight. The layers were separated and the aqueous phase extracted with Et_2O (2 × 10 mL). The combined organic layers were dried ($MgSO_4$) and concentrated *in vacuo* to yield **37** (913 mg, quant.) as a colourless oil:

$\boldsymbol{R_f}$ = 0.17 (hexane:Et_2O, 8:2); $[\alpha]_D^{25.0}$ = –12.5 (*c* = 0.58, $CHCl_3$); **IR** (film) ν_{max}/cm^{-1} 3348, 2927, 2857; **^{1}H NMR** ($CDCl_3$, 600 MHz) δ = 5.92 (1H, s, C10H), 5.78 (1H, dt, *J* = 15.4, 5.4 Hz, C15H), 5.66 (1H, dd, *J* = 15.4, 6.0 Hz, C14H), 4.25–4.22 (1H, m, C13H), 4.14 (2H, d, *J* = 5.4 Hz, $C16H_2$), 2.41 (1H, dd, *J* = 13.4, 7.8 Hz, $C12H_AH_B$), 2.33 (1H, dd, *J* = 13.4, 4.8 Hz, $C12H_AH_B$), 1.85 (3H, s, $C23H_3$), 1.49 (1H, br s, OH), 0.88 (9H, s, $C(CH_3)_3$ of *t*Bu), 0.02 (3H, s, $Si(CH_3)_2$), 0.01 (3H, s, $Si(CH_3)_2$); **^{13}C NMR** ($CDCl_3$, 150 MHz) δ = 144.2 (C11), 134.2 (C14H), 128.9 (C15H), 77.9 (C10H), 70.8 (C13H), 63.0 ($C16H_2$), 48.2 ($C12H_2$), 25.8 (3C, $C(CH_3)_3$ of *t*Bu), 24.5 ($C23H_3$), 18.1 ($C(CH_3)_3$ of *t*Bu), −4.4 ($Si(CH_3)_2$), −4.8 ($Si(CH_3)_2$); **HRMS** (+ESI) Found $[M+Na]^+$ = 405.0708; $C_{14}H_{27}IO_2SiNa$ requires 405.0717, Δ 2.22 ppm.

((*R*,1*E*,5*E*)-7-Bromo-1-iodo-2-methylhepta-1,5-dien-4-yloxy)(*tert*butyl) dimethylsilane 38

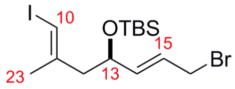

To a solution of **37** (90.2 mg, 0.236 mmol) in CH_2Cl_2 (0.5 mL) at −40 °C was added PPh_3 (68.0 mg, 0.260 mmol) and CBr_4 (86.0 mg, 0.260 mmol) in CH_2Cl_2 (2 mL) and the mixture was stirred for 3 h. Further PPh_3 (34.0 mg, 0.130 mmol) and CBr_4 (43 mg, 0.130 mmol) in CH_2Cl_2 (0.4 mL) were added and the mixture was stirred for a further 1.5 h. The reaction was quenched

with sat. aq. $NaHCO_3$ (2 mL), removed from the cold bath and diluted with CH_2Cl_2 (2 mL). After warming to RT, the layers were separated, and the aqueous phase extracted with CH_2Cl_2 (2 × 2 mL). The combined organic layers were washed with brine (4 mL), dried ($MgSO_4$) and concentrated *in vacuo*. Purification by column chromatography (SiO_2, hexane→hexane:Et_2O, 96:4) gave the title compound **38** (40.0 mg, 0.090 mmol) with a minor impurity (15%). The corresponding signals for **38** are reported for characterisation:

***R*$_f$** = 0.45 (hexane:Et_2O, 95:5); **IR** (film) ν_{max}/cm^{-1} 2954, 2929, 2856, 1617; **^{1}H NMR** ($CDCl_3$, 600 MHz) δ = 5.93 (1H, s, C10H), 5.87–5.82 (1H, m, C15H), 5.71 (1H, dd, *J* = 15.2, 5.8 Hz, C14H), 4.24–4.21 (1H, m, C13H), 3.94 (2H, d, *J* = 7.5 Hz, C16H$_2$), 2.40 (1H, dd, *J* = 13.4, 7.7 Hz, C12H_AH$_B$), 2.32 (1H, dd, *J* = 13.4, 4.8 Hz, C12H$_A$$H_B$), 1.85 (3H, s, C23CH$_3$), 0.88 (9H, s, C(C$H_3$)$_3$ of *t*Bu), 0.02 (3H, s, Si(CH_3)$_2$), 0.02 (3H, s, Si(CH_3)$_2$); **^{13}C NMR** ($CDCl_3$, 150 MHz) δ = 143.8 (C11), 137.7 (C14H), 126.1 (C15H), 78.2 (C10H), 70.4 (C13H), 47.9 (C12H$_2$), 32.1 (C16H$_2$), 25.8 (3C, C(CH$_3$)$_3$ of *t*Bu), 24.6 (C23H$_3$), 18.1 (C(CH$_3$)$_3$ of *t*Bu), −4.4 (Si(CH$_3$)$_2$), −4.9 (Si(CH$_3$)$_2$); **HRMS** (+ESI) Found [M+Na]$^+$ = 466.9864; $C_{14}H_{26}$BrIOSiNa requires 466.9873, Δ 1.93 ppm.

Diethyl (*R*,2*E*,6*E*)-4-(*tert*-butyldimethylsilyloxy)-7-iodo-6-methylhepta-2,6-dienylphosphonate 39

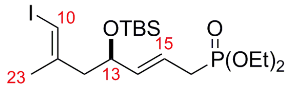

To **38** (40.0 mg, 0.090 mmol) was added freshly distilled P(OEt)$_3$ (100 μL). The mixture was heated at 100 °C for 4 h, after which the remaining P(OEt)$_3$ was removed under vacuum (100 °C). Purification by column chromatography (SiO_2, EtOAc:hexane, 8:2→9:1) gave the title compound **39** (40.0 mg, 80% over 2 steps) as a colourless oil.

***R*$_f$** = 0.33 (EtOAc:hexane, 8:2); $[\alpha]_D^{25.0}$ = +15.0 (*c* = 0.90, $CHCl_3$); **IR** (film) ν_{max}/cm^{-1} 2929, 2857, 1618, 1250, 1024; **^{1}H NMR** ($CDCl_3$, 600 MHz) δ = 5.92 (1H, s, C10H), 5.59–5.57 (2H, m, C14H and C15H), 4.21–4.19 (1H, m, C13H), 4.12–4.07 (4H, m, P(OCH_2CH$_3$)$_2$), 2.58–2.54 (2H,

m, C16H_2), 2.39 (1H, dd, *J* = 13.4, 7.7 Hz, C12H_AH_B), 2.31 (1H, dd, *J* = 13.4, 4.8 Hz, C12H_AH_B), 1.55 (3H, s, C23H_3), 1.32 (6H, t, *J* = 7.0 Hz, $P(OCH_2CH_3)_2$), 0.88 (9H, s, $C(CH_3)_3$ of *t*Bu), 0.02 (3H, s, $Si(CH_3)_2$), 0.01 (3H, s, $Si(CH_3)_2$); **^{13}C NMR** ($CDCl_3$, 150 MHz) δ = 144.2 (C11), 137.8 (d, *J* = 13.5 Hz, C15H), 119.3 (d, *J* = 10.5 Hz, C14H), 77.9 (C10H), 71.1 (C13H), 61.8 (d, *J* = 7.5 Hz, $POCH_2$), 61.7 (d, *J* = 6.0 Hz, $POCH_2$), 48.2 (C12H_2), 30.1 (d, *J* = 138 Hz, C16H_2), 25.8 (3C, $C(CH_3)_3$ of *t*Bu), 24.6 (C23H_3), 18.1 ($C(CH_3)_3$ of *t*Bu), 16.5–16.4 (m, $P(OCH_2CH_3)_2$), −4.5 ($Si(CH_3)_2$), −4.9 ($Si(CH_3)_2$); **HRMS** (+ESI) Found $[M+Na]^+$ = 525.1061; $C_{18}H_{36}IO_4PSiNa$ requires 525.1063, Δ 0.38 ppm.

1-*tert*-Butyl-5-((*R*,2*E*,6*E*)-4-(*tert*-butyldimethylsilyloxy)-7-iodo-6-methylhepta-2,6-dienylthio)-1*H*-tetrazole 40a

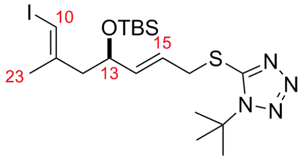

To a solution of **37** (74.0 mg, 0.194 mmol), 1-*tert*-butyl-1*H*-tetrazole-5-thiol (46.0 mg, 0.290 mmol) and PPh_3 (76.0 mg, 0.290 mmol) in THF (3 mL) at RT was added DIAD (69 µL, 0.350 mmol) dropwise, resulting in a yellow-orange solution. After 10 min, the solution was concentrated *in vacuo*. Purification by column chromatography (SiO_2, hexane:Et_2O, 9:1→7:3) gave the title compound **40a** (86.0 mg, 85%) as a colourless oil.

***R*$_f$** = 0.33 (hexane:Et_2O, 7:3); $[\alpha]_D^{25.0}$ = +7.3 (*c* = 1.10, $CHCl_3$); **IR** (film) ν_{max}/cm^{-1} 2928, 2856; **^{1}H NMR** ($CDCl_3$, 600 MHz) δ = 5.89 (1H, s, C10H), 5.79–5.78 (2H, m overlapped, C14H and C15H), 4.19–4.17 (1H, m, C13H), 4.00–3.98 (2H, m, C16H_2), 2.36 (1H, dd, *J* = 13.4, 7.6 Hz, C12H_AH_B), 2.29 (1H, dd, *J* = 13.4, 4.9 Hz, C12H_AH_B), 1.81 (3H, s, C23H_3), 1.71 (9H, s, $NC(CH_3)_3$), 0.84 (9H, s, $C(CH_3)_3$ of *t*Bu), −0.02 (3H, s, $Si(CH_3)_2$), −0.07 (3H, s, $Si(CH_3)_2$); **^{13}C NMR** ($CDCl_3$, 150 MHz) δ = 152.0 (S(*C*N)N), 143.9 (C11), 138.4 (C14H or C15H), 123.3 (C14H or C15H), 78.0 (C10H), 70.6 (C13H), 61.0 ($NC(CH_3)_3$), 48.0 (C12H_2), 35.3 (C16H_2), 28.8 (3C, $NC(CH_3)_3$), 25.8 (3C, $C(CH_3)_3$ of *t*Bu), 24.5 (C23H_3), 18.1 ($C(CH_3)_3$ of *t*Bu), −4.5 ($Si(CH_3)_2$), −4.9 ($Si(CH_3)_2$); **HRMS** (+ESI) Found $[M+Na]^+$ = 545.1255; $C_{19}H_{35}IN_4OSSiNa$ requires 545.1243, Δ 2.20 ppm.

1-*tert*-Butyl-5-((2*E*,6*E*)-4-(*tert*-butyldimethylsilyloxy)-7-iodo-6-methylhepta-2,6-dienylsulfonyl)-1*H*-tetrazole 41a

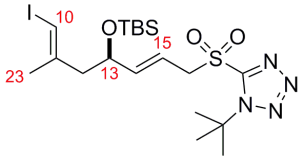

To a stirred solution of **40a** (63.0 mg, 0.121 mmol) in EtOH (2 mL) at 0 °C was added ammonium molybdate tetrahydrate (149 mg, 0.121 mmol) in H_2O_2 (100 volumes, 500 μL) dropwise. After 1 h, the ice-bath was removed then the reaction stirred for a further 2 h after which it was quenched with sat. aq. NH_4Cl (3 mL) and diluted with Et_2O (10 mL). The layers were separated then the organic layer was washed with brine (5 mL), dried ($MgSO_4$) and concentrated *in vacuo*. Purification by column chromatography (SiO_2, hexane:Et_2O, 9:1→8:2) gave the title compound **41a** (51.0 mg, 76%) as a colourless oil.

$\boldsymbol{R_f}$ = 0.27 (hexane:Et_2O, 8:2); $[\alpha]_D^{25.0}$ = +28.3 (*c* = 0.40, $CHCl_3$); **IR** (film) ν_{max}/cm^{-1}2929, 2857, 1340, 1162; **^{1}H NMR** ($CDCl_3$, 600 MHz) δ = 6.04 (1H, dd, *J* = 15.4, 5.2 Hz, C14H), 5.93 (1H, s,C10H), 5.81–5.76 (1H, m, C15H), 4.53 (1H, dd, *J* = 14.2, 7.1 Hz, C16H_AH$_B$), 4.48 (1H, dd, *J* = 14.2, 7.8 Hz, C16H$_A$$H_B$), 4.26–4.23 (1H, m,C13H), 2.38–2.30 (2H, m, C12H_2), 1.84–1.83 (12H, m overlapped, C23CH_3 and NC(CH_3)$_3$), 0.84 (9H, s, C(CH_3)$_3$ of *t*Bu), −0.03 (3H, s, Si(CH_3)$_2$), −0.11 (3H, s, Si(CH_3)$_2$)); **^{13}C NMR** ($CDCl_3$, 150 MHz) δ = 153.7 (S(*C*N)N), 145.7 (C14H), 143.5 (C11), 113.7 (C15H), 78.5 (C10H), 70.3 (C13H), 65.4 (N*C*(CH_3)$_3$), 59.7 (C16H_2), 47.7 (C12H_2), 29.7 (NC(*C*H_3)$_3$), 25.7 (3C, C(*C*H_3)$_3$ of *t*Bu), 24.5 (C23H_3), 18.0 (*C*(CH_3)$_3$ of *t*Bu), −4.8 (Si(*C*H_3)$_2$), −5.0 (Si(*C*H_3)$_2$); **HRMS** (+ESI) Found $[M+Na]^+$ = 577.1146; $C_{19}H_{35}IN_4O_3SSiNa$ requires 577.1142, Δ 0.69 ppm.

5-((*R*,2*E*,6*E*)-4-(*tert*-Butyldimethylsilyloxy)-7-iodo-6-methylhepta-2,6-dienylthio)-1-phenyl-1*H*-tetrazole 40b

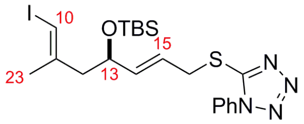

To a solution of **37** (189 mg, 0.495 mmol), 1-phenyl-1*H*-tetrazole-5-thiol (132 mg, 0.743 mmol) and PPh_3 (195 mg, 0.743 mmol) in THF (6 mL) at RT was added DIAD (175 µL, 0.891 mmol) dropwise, resulting in a yellow-orange solution. After 10 min, the solution was concentrated *in vacuo*. Purification by column chromatography (SiO_2, hexane:Et_2O, 95:5→7:3) gave the title compound **40b** (258 mg, 96%) as a colourless oil.

$\boldsymbol{R_f}$ = 0.21 (hexane:Et_2O, 8:2); $[\alpha]_D^{25.0}$ = +11.1 (*c* = 0.95, $CHCl_3$); **IR** (film) ν_{max}/cm^{-1} 2928, 2855, 1597; **^{1}H NMR** ($CDCl_3$, 600 MHz) δ = 7.58–7.53 (5H, m, Ar), 5.89 (1H, s, C10H), 5.81–5.79 (2H, m, C14H and C15H), 4.20–4.17 (1H, m, C13H), 4.01 (2H, d, *J* = 5.8 Hz, C16H_2), 2.36 (1H, dd, *J* = 13.4, 6.8 Hz, C12H_AH_B), 2.29 (1H, dd, *J* = 13.4, 5.0 Hz, C12H_AH_B), 1.82 (3H, s, C23H_3), 0.84 (9H, s, C(CH_3)$_3$ of *t*Bu), −0.02 (3H, s, Si(CH_3)$_2$), −0.07 (3H, s, Si(*C*H_3)$_2$); **^{13}C NMR** ($CDCl_3$, 150 MHz) δ = 153.7 (S(*C*N)N), 143.8 (C11), 138.7 (C14H or C15H), 133.7 (*ipso*-Ar-C), 130.1 (*para*-Ar-C), 129.8 (2C, *ortho*-Ar-C or *meta*-Ar-C), 123.8 (2C, *ortho*-Ar-C or *meta*-Ar-C), 123.0 (C14H or C15H), 78.1 (C10H), 70.5 (C13H), 48.0 (C12H_2), 34.7 (C16H_2), 25.8 (3C, C(*C*H_3)$_3$ of *t*Bu), 24.6 (C23H_3), 18.1 (*C*(CH_3)$_3$ of *t*Bu), −4.5 (Si(*C*H_3)$_2$), −4.9 (Si(*C*H_3)$_2$); **HRMS** (+ESI) Found $[M+Na]^+$ = 565.0950; $C_{21}H_{31}IN_4OSSiNa$ requires 565.0930, Δ 3.54 ppm.

5-((*R*,2*E*,6*E*)-4-(*tert*-Butyldimethylsilyloxy)-7-iodo-6-methylhepta-2,6-dienylsulfonyl)-1-phenyl-1*H*-tetrazole 41b

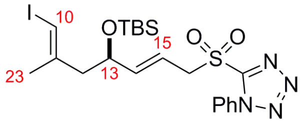

To a stirred solution of **40b** (85.0 mg, 0.157 mmol) in EtOH (3 mL) at 0 °C was added ammonium moylbdate tetrahydrate (387 mg, 0.313 mmol) in H_2O_2 (100 volumes, 1 mL)

dropwise. The reaction mixture became yellow solution and a white precipitate formed. The ice-bath was removed, the reaction stirred for a further 2 h at RT after which it was quenched by the addition of sat. aq. NH_4Cl (4 mL) and diluted with Et_2O (15 mL). The layers were separated and the organic layer washed with brine (2 × 5 mL), dried (Na_2SO_4) and concentrated *in vacuo*. Purification by column chromatography (SiO_2, hexane:Et_2O, 9:1→8:2) gave the title compound **41b** (74.0 mg, 82%) as a colourless oil.

R$_f$ = 0.21 (hexane:Et_2O, 8:2); $[\alpha]_D^{25.0}$ = +13.4 (*c* = 1.05, $CHCl_3$); **IR** (film) ν_{max}/cm^{-1} 2952, 2929, 2857, 1596, 1345, 1148; **^{1}H NMR** ($CDCl_3$, 600 MHz) δ = 7.67–7.59 (5H, m, Ar), 6.02 (1H, dd, *J* = 15.3, 5.0 Hz, C14H), 5.91 (1H, s, C10H), 5.78–5.74 (1H, m, C15H), 4.46 (1H, dd, *J* = 14.4, 7.1 Hz, C16H_AH_B), 4.40 (1H, dd, *J* = 14.4, 7.7 Hz, C16H_AH_B) 4.25–4.22 (1H, m, C13H), 2.36–2.28 (2H, m, C12H_AH_B), 1.82 (3H, s, C23CH_3), 0.84 (9H, s, C(CH_3)$_3$ of *t*Bu), −0.03 (3H, s, Si(CH_3)$_2$), −0.11 (3H, s, Si(CH_3)$_2$); **^{13}C NMR** ($CDCl_3$, 150 MHz) δ = 153.1 (S(*C*N)N), 146.1 (C14H) 143.4 (C11), 133.0 (*ipso*-Ar-C), 131.5 (*para*-Ar-C), 129.7 (2C, *ortho*-Ar-C or *meta*-Ar-C), 125.1 (2C, *ortho*-Ar-C or *meta*-Ar-C), 112.9 (C15H), 78.6 (C10H), 70.2 (C13H), 59.1 (C16H_2), 47.6 (C12H_2), 25.7 (3C, C(CH_3)$_3$ of *t*Bu), 24.5 (C23H_3), 18.0 (*C*(CH_3)$_3$ of *t*Bu), −4.8 (Si(CH_3)$_2$), −5.0 (Si(CH_3)$_2$); **HRMS** (+ESI) Found $[M+Na]^+$ = 597.0829; $C_{21}H_{31}IN_4O_3SSiNa$ requires 597.0829, Δ 0.00 ppm.

2-((*R*,2*E*,6*E*)-4-(*tert*-Butyldimethylsilyloxy)-7-iodo-6-methylhepta-2,6-dienylthio)benzo[*d*]thiazole 40c

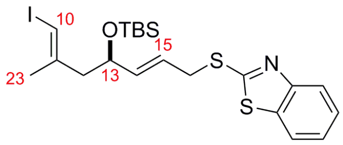

To a solution of **37** (90.2 mg, 0.236 mmol), mercaptobenzothiazole (59.0 mg, 0.354 mmol) and PPh_3 (93.0 mg, 0.354 mmol) in THF (4 mL) at RT was added DIAD (86 μL, 0.425 mmol) dropwise, resulting in a yellow-orange solution. After 10 min, the solution was concentrated *in vacuo*. Purification by column chromatography (SiO_2, hexane→hexane:Et_2O, 95:5) gave the title compound **40c** (131.0 mg, quant.) as a colourless oil.

***R*$_f$** = 0.18 (hexane:Et_2O, 95:5); $[\alpha]_D^{25.0}$ = +12.4 (*c* = 0.75, $CHCl_3$); **IR** (film) ν_{max}/cm^{-1} 2954, 2927, 2855, 1618; **^{1}H NMR** ($CDCl_3$, 600 MHz) δ = 7.87 (1H, d, *J* = 7.9 Hz, Ar), 7.76 (1H, d, *J* = 7.9 Hz, Ar), 7.42 (1H, t, *J* = 7.9 Hz, Ar), 7.30 (1H, t, *J* = 7.9 Hz, Ar), 5.87 (1H, s, C10H), 5.81–5.78 (2H, m, C14H and C15H), 4.20–4.18 (1H, m, C13H), 3.97 (2H, d, *J* = 5.6 Hz, C16H_2), 2.37 (1H, dd, *J* = 13.5, 7.7 Hz, C12H_AH_B), 2.28 (1H, dd, *J* = 13.5, 4.6 Hz, C12H_AH_B), 1.82 (3H, s, C23CH_3), 0.83 (9H, s, C(CH_3)$_3$ of *t*Bu), −0.02 (3H, s, Si(CH_3)$_2$), −0.06 (3H, s, Si(CH_3)$_2$); **^{13}C NMR** ($CDCl_3$, 150 MHz) δ = 165.1 (Ar), 153.2 (Ar), 144.0 (C11), 137.6 (C14H or C15H), 135.3 (Ar), 126.0 (Ar), 124.3 (Ar), 124.0 (C14H or C15H), 121.6 (Ar), 121.0 (Ar), 78.0 (C10H), 70.8 (C13H), 48.1 (C12H_2), 35.0 (C16H), 25.8 (3C, C(CH_3)$_3$ of *t*Bu), 24.6 (C23H_3), 18.1 (*C*(CH_3)$_3$ of *t*Bu), −4.5 (Si(CH_3)$_2$), −4.9 (Si(CH_3)$_2$); **HRMS** (+ESI) Found $[M+H]^+$ = 532.0684; $C_{21}H_{31}INOS_2Si$ requires 532.0661, Δ 4.32 ppm.

2-((*R*,2*E*,6*E*)-4-(*tert*-Butyldimethylsilyloxy)-7-iodo-6-methylhepta-2,6-dienylthio)benzo[*d*]thiazole 41c

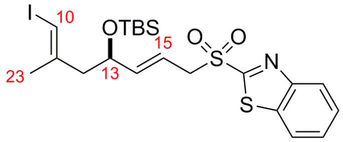

To a stirred solution of **40c** (48.0 mg, 0.090 mmol) in EtOH (3 mL) at 0 °C was added ammonium molybdate tetrahydrate (167 mg, 0.135 mmol) in H_2O_2 (100 volumes, 500 µL). The reaction mixture became a yellow solution and a white precipitate formed. The ice-bath was removed, the reaction stirred at RT for 2 h then quenched with sat. aq. NH_4Cl (4 mL) and diluted with Et_2O (15 mL). The layers were separated and the organic layer washed with brine (3 × 5 mL), dried (Na_2SO_4) and concentrated *in vacuo*. Purification by column chromatography (SiO_2, hexane:Et_2O, 9:1→8:2) gave the title compound **41c** (43.0 mg, 85%) as a colourless oil.

***R*$_f$** = 0.49 (hexane:Et_2O, 1:1); $[\alpha]_D^{25.0}$ = +10.3 (*c* = 1.05, $CHCl_3$); **IR** (film) ν_{max}/cm^{-1}2953, 2928, 2856, 1555, 1332, 1142; **^{1}H NMR** ($CDCl_3$, 600 MHz) δ = 8.22 (1H, d, *J* = 8.1 Hz, Ar), 8.01 (1H, d, *J* = 8.1 Hz, Ar), 7.65 (1H, dd, *J* = 8.1, 7.5 Hz, Ar), 7.60 (1H, dd, *J* = 8.1, 7.5 Hz, Ar), 5.78 (1H, s, C10H), 5.75–5.67 (2H, m, C14H and C15H), 4.27–4.19 (2H, m, C16H_2), 4.17–4.14 (1H, m, C13H), 2.22 (1H, dd, *J* = 13.4, 7.7 Hz, C12H_AH_B), 2.14 (1H, dd, *J* = 13.4, 4.8 Hz, C12H_AH_B),

1.76 (3H, s, C23CH$_3$), 0.79 (9H, s, C(C*H*$_3$)$_3$ of *t*Bu), −0.05 (3H, s, Si(*C*H$_3$)$_2$), −0.12 (3H, s, Si(*C*H$_3$)$_2$); **^{13}C NMR** (CDCl$_3$, 150 MHz) δ = 165.4 (Ar), 152.6 (Ar), 144.4 (C14H or C15H), 143.5 (C11), 136.7 (Ar), 128.1 (Ar), 127.7 (Ar), 125.5 (Ar), 122.3 (Ar), 114.3 (C14H or C15H), 78.3 (C10H), 70.2 (C13H), 57.7 (C16H$_2$), 47.7 (C12H$_2$), 25.7 (3C, C(*C*H$_3$)$_3$ of *t*Bu), 24.5 (C23H$_3$), 18.0 (*C*(CH$_3$)$_3$ of *t*Bu), −4.8 (Si(*C*H$_3$)$_2$), −5.0 (Si(*C*H$_3$)$_2$); **HRMS** (+ESI) Found [M+Na]$^+$ = 586.0385; $C_{21}H_{30}INO_3S_2SiNa$ requires 586.0379, Δ 1.02 ppm.

(1*S*,2*R*)-2-Benzoyl-cyclopropanecarboxylic acid diethylamide 45

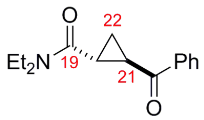

To a vigorously stirred slurry of Cs_2CO_3 (19.0 g, 58.3 mmol) and **44** (3.27 g, 9.64 mmol) in MeCN (60 mL) at 80 °C was added a solution of 1-phenyl-propenone **43** (7.70 g, 58.3 mmol) and 2-bromo-*N*,*N*-diethyl-acetamide **42** (9.38 g, 48.6 mmol) in MeCN (250 mL) dropwise over 24 h. Following the addition, the reaction was stirred for an additional 12 h at 80 °C after which it was cooled to RT and diluted with Et_2O (200 mL). The mixture was washed with 1 *N* HCl (150 mL) and the layers separated. The aqueous layer was further extracted with Et_2O (200 mL) and the combined organics dried ($MgSO_4$) and concentrated *in vacuo*. Purification by column chromatography [SiO_2, petroleum ether (30–40):Et_2O, 1:1] gave the title compound **45** (10.4 g, 82%) as a pale yellow oil.

***R*$_f$** = 0.38 [petroleum ether (30–40):Et_2O, 1:1]; $[\alpha]_D^{25.0}$ = –121.2 (*c* = 0.90, CHCl$_3$); **IR** (film) ν_{max}/cm^{-1} 2976, 2935, 1671, 1620, 1448, 1385, 1331, 1259, 1220, 1142; **^{1}H NMR** (CDCl$_3$, 600 MHz) δ = 8.04 (2H, d, *J* = 7.3 Hz, *ortho*-Ar-H), 7.58 (1H, t, *J* = 7.3 Hz, *para*-Ar-H), 7.48 (2H, t, *J* = 7.3 Hz, *meta*-Ar-H), 3.48–3.43 (2H, m, NC*H*$_2$CH$_3$), 3.41 (2H, q, *J* = 7.1 Hz, NC*H*$_2$'CH$_3$'), 3.25 (1H, ddd, *J* = 8.4, 5.4, 3.9 Hz, C21H), 2.53 (1H, ddd, *J* = 8.6, 5.9, 3.9 Hz, C20H), 1.63 (1H, ddd, *J* = 8.6, 5.9, 2.9 Hz, C22*H$_A$*H$_B$), 1.55 (1H, ddd, *J* = 8.4, 5.5, 2.9 Hz, C22H$_A$*H$_B$*), 1.21 (3H, t, *J* = 7.2 Hz, NCH$_2$C*H*$_3$), 1.21 (3H, t, *J* = 7.1 Hz, NCH$_2$'C*H*$_3$'); **^{13}C NMR** (CDCl$_3$, 150 MHz) δ = 198.5 (C=O), 169.7 (C19), 137.2 (*ipso*-Ar-C), 133.3 (*para*-Ar-C), 128.6 (2C, *meta*-Ar-C), 128.4 (2C, *ortho*-Ar-C), 42.3 (N*C*H$_2$CH$_3$), 41.1 (N*C*H$_2$'CH$_3$'), 25.9 (C21H),

23.7 (C20H), 18.2 ($C22H_2$), 14.8 (NCH_2CH_3), 13.2 ($NCH_2'CH_3'$); **HRMS** (EI) Found $[M]^+$ = 245.1417; $C_{15}H_{19}NO_2$ requires 245.1416, Δ 0.41 ppm.
HPLC: Daicel Chiralpak AD-H. Hexane:*i*PrOH, 98:2, 1 mL/min, 254 nm: t_R (major) = 26 min, t_R (minor) = 20 min. er = 98.5:1.5. All spectroscopic data in agreement with that previously published.[10]

(l*R*,2*S*)-2-Diethylcarbamoyl-cyclopropanecarboxylic acid 46

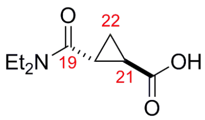

To a solution of **45** (2.00 g, 8.15 mmol) and Urea•H_2O_2 (7.67 g, 81.6 mmol) in HFIP (20 mL) at 0 °C was added TFAA (2.88 mL, 20.4 mmol) dropwise (*caution: exothermic*) and the reaction mixture slowly warmed to RT. After 12 h stirring at RT additional TFAA (2.88 mL, 20.4 mmol) was added and the stirring continued for 12 h. At this point further TFAA (2.88 mL, 20.4 mmol) was added and the reaction stirred for a final supplementary period of 12 h. The reaction was diluted with Et_2O (200 mL) and carefully poured onto a mixture of $Na_2S_2O_4$ (100 mL) and sat. aq. $NaHCO_3$ (100 mL). The layers were separated and the aqueous layer further extracted with Et_2O (2 × 100 mL). The combined organic layers were dried ($MgSO_4$) and concentrated *in vacuo*. Purification by column chromatography [SiO_2, petroleum ether (30:40):Et_2O, 1:1] gave a 2.4:1 (by ^1H NMR) mixture (2.12 g) of the desired ester [$\boldsymbol{R_f}$ = 0.35 (petroleum ether:Et_2O, 1:1)].and starting material **S14**. The mixture was used directly in the next step of the reaction sequence.

To a solution of **S14** (2.12 g) in MeCN (5 mL) at RT was added a solution of NaOH (1.63 g, 40.8 mmol) in H_2O (20 mL) dropwise. The reaction mixture was heated to 50 °C and maintained at this temperature for 4 h after which it was cooled to RT and diluted with Et_2O (50 mL). The aqueous phase was neutralised by the dropwise addition of 3 *N* HCl and extracted with Et_2O (2 × 100 mL). The aqueous layer was then further acidified to pH 1 by the addition of aqueous 3 *N* HCl, and re-extracted with EtOAc (5 × 100 mL). The combined organics were dried ($MgSO_4$)

and concentrated *in vacuo* to give the title compound (**46)** contaminated with phenol. The phenol was removed from the crude reaction mixture by evaporation under vacuum (<10 mmHg) and high temperature (55 °C) to provide the title compound **46** (731 mg, 48% over 2 steps) as a yellow oil, which crystallised as needles on storage.

m.p. = 68–70 °C; $[\alpha]_D^{25.0}$ = +179.8 (*c* = 0.40, $CHCl_3$); **IR** (film) ν_{max}/cm^{-1} 2982, 2938, 1725, 1585, 1493, 1430, 1404, 1364, 1316, 1265, 1179, 1147, 1081; **^{1}H NMR** ($CDCl_3$, 600 MHz) δ = 3.48 (2H, q, *J* = 7.2 Hz, NCH_2CH_3), 3.45–3.38 (2H, m, $NCH_2'CH_3'$), 2.38–2.32 (1H, m, C21H), 2.26–2.21 (1H, m, C20H), 1.59–1.53 (1H, m, $C22H_AH_B$), 1.45–1.39 (1H, m, $C22H_AH_B$), 1.25 (3H, t, *J* = 7.2 Hz, NCH_2CH_3), 1.11 (3H, t, *J* = 7.1 Hz, $NCH_2'CH_3'$); **^{13}C NMR** ($CDCl_3$, 150 MHz) δ = 177.8 (C=O), 169.1 (C19), 42.4 (NCH_2CH_3), 41.2 ($NCH_2'CH_3'$), 21.7 (C21H), 21.5 (C20H), 15.8 ($C22H_2$), 14.8 (NCH_2CH_3), 13.1 ($NCH_2'CH_3'$); An accurate mass could not be obtained for this compound.

(1*R*,2*S*)-2-Diethylcarbamoyl-cyclopropanecarbonyl chloride S15

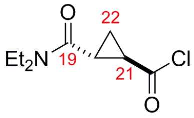

To **S14** (100 mg, 0.541 mmol) was added thionyl chloride (5 mL) and the reaction mixture stirred at RT for 12 h. The mixture was then concentrated *in vacuo* to give the title compound **S15** (108 mg, 98%) as a yellow oil which was used directly in the next step of the reaction sequence.

(1*S*,2*R*)-2-Chloro-cyclopropanecarboxylic acid diethylamide 47

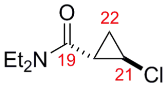

To a solution of **S15** (108 mg, 0.512 mmol) in CCl_4 (25.6 mL) was added 2-mercaptopyridine *N*-oxide sodium salt (92 mg, 0.615 mmol), DMAP (13 mg, 0.102 mmol) and TBAI (38 mg,

0.102 mmol). The reaction was stirred in the absence of light for 1 h after which TLC analysis indicated the formation of a major product [$\boldsymbol{R_f}$ = 0.16 (petroleum ether (30-40):Et_2O, 4:1)]. At this point AIBN (4 mg, 0.256 mmol) was added and the reaction heated to 80 °C for 5 h in the absence of light. The reaction mixture was cooled to RT and the volatiles removed *in vacuo* to leave a dark brown residue. Purification by column chromatography [SiO_2, petroleum ether (30–40):Et_2O, 15:1→4:1] gave the title compound **47** (51 mg, 57%) as a colourless oil.

$\boldsymbol{R_f}$ = 0.34 [petroleum ether (30–40):Et_2O, 1:1]; $[\alpha]_D^{25.0}$ = –80.0 (*c* = 0.75, $CHCl_3$); **IR** (film) ν_{max}/cm^{-1} 2974, 1630, 1449, 1362, 1271, 1244, 1222, 1142, 1076; **^{1}H NMR** ($CDCl_3$, 600 MHz) δ = 3.51–3.46 (2H, m, NC$H_2$$CH_3$), 3.41–3.36 (3H, m, NC$H_2$'$CH_3$' and C21H), 2.11–2.05 (1H, m, C20H), 1.64–1.58 (1H, m, C22$H_A$$H_B$), 1.29–1.24 (4H, m, NCH_2CH_3 and C22$H_A$$H_B$), 1.11 (3H, t, *J* = 7.2 Hz, NCH_2'CH_3'); **^{13}C NMR** ($CDCl_3$, 150 MHz) δ = 169.1 (C19), 42.3 (N*C*H_2CH_3), 41.0 (N*C*H_2'CH_3'), 33.7 (C21H), 22.6 (C20H), 17.6 (C22H_2), 14.9 (NCH_2*C*H_3), 13.2 (NCH_2'*C*H_3'); **HRMS** (ESI) Found $[M+H]^+$ = 176.0837; $C_8H_{15}ClNO$ requires 176.0842, Δ 2.84 ppm.

(4*S*,5*S*)-2-Butyl-*N*,*N*,*N′*,*N′*-tetramethyl-1,3,2-dioxaborolane-4,5-dicarboxamide 51[11]

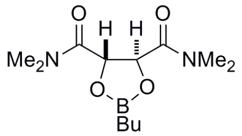

To a solution of (–)-*N*,*N*,*N′*,*N′*-tetramethyl-D-tartaric acid (20 g, 97.9 mmol) in PhMe (65 mL) was added 1-butaneboronic acid (11.98 g, 117.5 mmol) in one portion. The mixture was heated at reflux under Dean–Stark conditions until *ca*. 3.5 mL of H_2O was collected. The reaction was cooled to RT and the PhMe removed *in vacuo*. The mixture was suspended in CH_2Cl_2 (35 mL), filtered through a pad of Celite® under suction using CH_2Cl_2 (3 × 100 mL). The mixture was once again concentrated *in vacuo* to give the title compound **51** (24.6 g, 93%) as a colourless oil.

$[\alpha]_D^{25.0}$ = +99.0 (*c* = 1.0, $CHCl_3$), [lit.[14] $[\alpha]_D^{20}$ = +106 (*c* = 1.00, $CHCl_3$)]; **IR** (film) ν_{max}/cm^{-1} 3433, 2928, 2872, 1644, 1501, 1466, 1401, 1380, 1254, 1218, 1153, 1059, 1025, 971; **^{1}H NMR** ($CDCl_3$, 400 MHz) δ = 5.52 (2H, s, 2 × C*H*CON$(CH_3)_2$), 3.20 (6H, s, N$(CH_3)_2$), 2.98 (6H, s,

N(CH_3)$_2$), 1.44–1.34 (2H, m, CH_2 of Bu), 1.34–1.26 (2H, m, CH_2 of Bu), 0.89–0.83 (5H, m, BCH_2 and CH_3 of Bu); **^{13}C NMR** ($CDCl_3$, 100 MHz) δ = 168.5 (2 × CH*C*ON(CH_3)$_2$), 75.8 (2 × *C*HCON(CH_3)$_2$), 37.2 (2C, N(CH_3)$_2$), 36.0 (2C, N(CH_3)$_2$), 25.9 (CH_3*C*H_2), 25.2 (BCH_2*C*H_2), 13.8 (*C*H_3CH_2)†; **HRMS** (+ESI) Found $[M+Na]^+$ = 293.1631; $C_{12}H_{23}O_4N_2BNa$ requires 293.1643, Δ 4.09 ppm. All spectroscopic data in agreement with that previously published.

((1*S*,2*R*)-2-Chlorocyclopropyl)methanol 52[12]

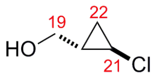

To a solution of $ZnEt_2$ (7.31 mL, 71.3 mmol) in CH_2Cl_2 (71 mL) cooled to 0 °C (internal) was added CH_2I_2 (11.5 mL, 142.6 mmol) dropwise over 90 min.† Once the addition was complete a pre-mixed solution of (*E*)-3-chloroprop-2-en-1-ol (**50**)[13] (1 g, 10.8 mmol), (4*S*,5*S*)-2-butyl-*N*,*N*,*N*′,*N*′-tetramethyl-1,3,2-dioxaborolane-4,5-dicarboxamide **51** (3.21 g, 11.9 mmol) in CH_2Cl_2 (79 mL) was added rapidly *via* cannula over 5 min ensuring that the internal temperature did not exceed 10 °C. The reaction mixture was allowed to warm to RT slowly over 16 h. The reaction was quenched with sat. aq. NH_4Cl (10 mL) and 3 *N* HCl (36 mL) added and stirred for 10 min. After this time, 5 M KOH (140 mL) was added to the entire mixture at RT and stirred for 4 h. The layers were separated and the aqueous layer further extracted with Et_2O (3 × 100 mL). The combined organic layers were dried ($MgSO_4$) and concentrated *in vacuo*. Purification by column chromatography [SiO_2, petroleum ether (30–40):Et_2O, 3:2] gave the title compound **52** (848 mg, 74%) as a colourless oil.

***R*$_f$** = 0.37 [petroleum ether (30–40):Et_2O, 1:1]; $[\alpha]_D^{26.9}$ = –60.5 (*c* = 0.97, $CHCl_3$), [lit.[15] $[\alpha]_D^{20}$ = –54.5 (c = 0.56, $CHCl_3$)]; **IR** (film) ν_{max}/cm^{-1} 3323, 2925, 2875, 1442, 1399, 1368, 1318, 1271, 1086, 1025; **^{1}H NMR** ($CDCl_3$, 400 MHz) δ = 3.63–3.56 (1H, m, C19H_AH_B), 3.54–3.47 (1H, m, C19H_AH_B), 2.94–2.89 (1H, m, C21H), 1.54–1.48 (2H, m, C20H and OH), 1.05–0.99 (1H, m, C22H_AH_B), 0.91 (1H, m, C22H_AH_B); **^{13}C NMR** ($CDCl_3$, 100 MHz) δ = 63.9 (C19H_2), 30.8 (C21H), 24.5 (C20H), 13.6 (C22H_2); **HRMS** (+ESI) Found $[M+Na]^+$ = 129.0082; C_4H_7OClNa

† Due to the long relaxation time the reported broad chemical shift of 9.9 ppm for BCH_2 could not be accounted for.
†Note - ′violent explosions′ have been reported during this addition in excess of an 8 mmol scale.[11]

requires 129.0077, Δ 3.88 ppm. All spectroscopic data in agreement with that previously published.[12]

GC analysis of **52** was performed using a 6890N Network GC system (Agilent Technologies Inc., Palo Alto, CA, USA), equipped with a Varian CP7502, CHIRASIL DEX CB (25.0 m × 250 µm × 0.25 µ nominal) capillary column. The GC analyses were carried out in split mode (ratio 50:1) using helium as a carrier gas at a flow rate of 134 mL/min 25.00 psig). The injection port temperature was 250 °C the oven was maintained at an initial temperature of 100 °C, programmed at 0.5 °C/min to reach a temperature of 120 °C where it was held, post-run, for 1 min. The FID detector was at 250 °C, using H_2 flow at 40.00 mL/min, air at 450 mL/min and helium makeup flow at 45.0 mL/min. The two enantiomers eluted at 6.0 and 6.3 min respectively. Cyclopropane **52** was shown to have er = 97.5:2.5.

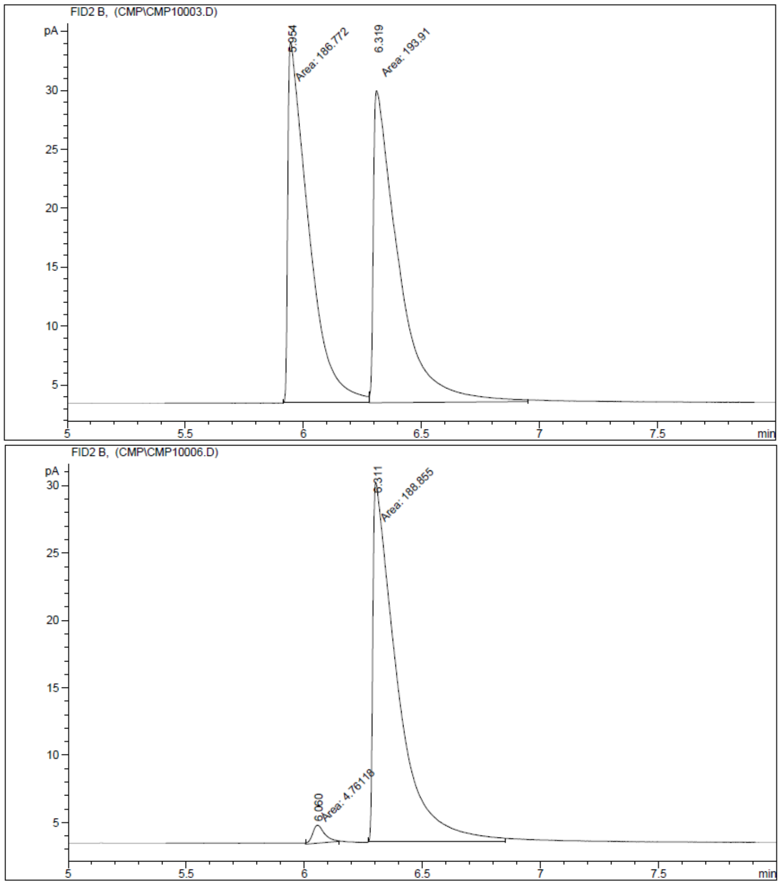

(1*R*,2*S*)-1-Chloro-2-(2,2-dibromoethenyl)cyclopropane 48[12]

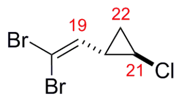

Procedure from compound 52

To a solution of **52** (848 mg, 7.96 mmol) in CH_2Cl_2 (57 mL) at RT was added Celite® (3.43 g) followed by PCC (3.43 g, 15.92 mmol) in one portion and the brown mixture stirred for 12 h. The reaction was diluted with Et_2O (50 mL) and filtered through a pad of silica. The solvent was carefully removed by distillation at 65 °C using a vigreux column and the crude aldehyde (**53**) (assumed quant.) used directly in the next step.

A solution of PPh_3 (8.35 g, 31.8 mmol) in CH_2Cl_2 (34 mL) was cooled to 0 °C and CBr_4 (5.28 g, 15.9 mmol) added in one portion, resulting in a bright yellow solution. This solution was stirred for 10 min, after which a solution of crude aldehyde **53** (assumed quant., 7.96 mmol) in CH_2Cl_2 (10 mL) was added dropwise *via* cannula. The resulting brown mixture was allowed to warm to RT slowly over 22 h. The reaction mixture was transferred to a conical flask and pentane (100 mL) added resulting in precipitation. The mixture was stirred vigorously for 1 min, and the liquor filtered through a sinter funnel. The remaining brown residue was re-suspended in CH_2Cl_2 (15 mL) and pentane (50 mL) added. Once again, the mixture was stirred vigorously and the liquor decanted off. This process was repeated once more, and the combined organics concentrated *in vacuo* (400 mbar, RT). Purification by column chromatography (SiO_2, pentane) gave the title compound **48** (1.44 g, 70% over 2 steps) as a colourless oil.

$\boldsymbol{R_f}$ = 0.58 (pentane): $[\alpha]_D^{26.1}$ = –75.7 (*c* = 1.03, $CHCl_3$), [lit.[12] $[\alpha]_D^{20}$ = –70.9 (*c* = 0.65, $CHCl_3$)]; **IR** (film) ν_{max}/cm^{-1} 3022, 1609, 1432, 1360, 1279, 1241, 1197, 1162, 1086, 1066, 1042; **^{1}H NMR** ($CDCl_3$, 400 MHz) δ = 5.84 (1H, d, *J* = 8.9 Hz, C19H), 3.08–3.03 (1H, m, C21H), 2.07–1.99 (1H, m, C20H), 1.40–1.34 (1H, m, C22H_AH_B), 1.18–1.12 (1H, m, C22H_AH_B); **^{13}C NMR** ($CDCl_3$, 100 MHz) δ = 137.2 (C19H), 89.8 (C18Br_2), 33.0 (C21H), 26.0 (C20H), 17.5 (C22H_2); **HRMS** (+EI) Found $[M]^+$ = 257.8445; $C_5H_5ClBr_2$ requires 257.8441, Δ 1.55 ppm. All spectroscopic data in agreement with that previously published.[12]

3-((1*S*,2*R*)-2-Chlorocyclopropyl)prop-2-yn-1-ol 57

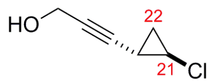

To a solution of **48** (177 mg, 0.680 mmol) in THF (14 mL) at −78 °C was added *n*BuLi (1.6 M in hexane; 850 µL, 1.36 mmol) dropwise, and the resulting solution stirred for 30 min. To this solution was added paraformaldehyde (38.0 mg, 1.36 mmol) in one portion and the reaction stirred for 10 min. The cold bath was removed and the reaction stirred for a further 30 min before quenching with sat. aq. NH_4Cl (5 mL). The resulting layers were separated, the aqueous phase was extracted with Et_2O (5 mL), the combined organic layers dried ($MgSO_4$) and concentrated *in vacuo*. Purification by column chromatography (SiO_2, hexane:Et_2O, 75:25→65:35) gave the title compound **57** (63.0 mg, 71%) as a colourless oil.

$\boldsymbol{R_f}$ = 0.14 (hexane:Et_2O, 7:3); $[\alpha]_D^{25.0}$ = –166.5 (*c* = 0.95, $CHCl_3$); **IR** (film) ν_{max}/cm^{-1} 3326, 2932, 2869; **^{1}H NMR** ($CDCl_3$, 600 MHz) δ = 4.22 (2H, dd, *J* = 6.1, 1.8 Hz, C17H_2), 3.17–3.14 (1H, m, C21H), 1.71–1.68 (1H, m, C20H), 1.46 (1H, t, *J* = 6.1 Hz, OH), 1.27–1.24 (2H, m, C22H_2); **^{13}C NMR** ($CDCl_3$, 150 MHz) δ = 85.1 (C19), 76.5 (C18), 51.2 (C17H_2), 33.8 (C21H), 18.8 (C22H_2), 11.2 (C20H); **HRMS** (+ESI) Found $[M+Na]^+$ = 153.0077; C_6H_7ClONa requires 153.0083, Δ 3.92 ppm.

5-((3-((1*S*,2*R*)-2-Chlorocyclopropyl)prop-2-yn-1-yl)thio)-1-phenyl-1*H*-tetrazole 59

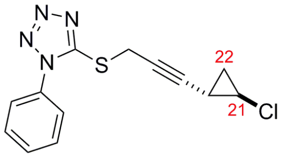

To a solution of **57** (25.0 mg, 0.192 mmol) in THF (1.5 mL) at RT was added PPh_3 (75.0 mg, 0.288 mmol), 1-phenyl-1*H*-tetrazole-5-thiol (68.0 mg, 0.384 mmol) and DIAD (68.0 µl, 0.345 mmol) sequentially. After stirring for 10 mins, the solvent was removed *in vacuo.* Purification by column chromatography (SiO_2, hexane:Et_2O, 4:1) gave the title compound **59** (50.0 mg, 89%) as a colourless oil.

R_f = 0.25 (hexane:Et_2O, 4:1), $[\alpha]_D^{25.0}$ = –75.0 (*c* = 1.00, $CHCl_3$),.IR (film) ν_{max} /cm^{-1} 1598, 1498, 1462, 1235, 1090, 1074, 1049, 1014, 994, 919; **^{1}H NMR** ($CDCl_3$, 600 MHz) δ = 7.57 (5H, br s, Ph), 4.13 (2H, d, *J* = 1.8 Hz, $C17H_2$), 3.13–3.10 (1H, m, C21H), 1.67–1.62 (1H, m, C20H), 1.24–1.20 (2H, m, $C22H_2$); **^{13}C NMR** ($CDCl_3$, 125 MHz) 153.0 (S(*C*N)N), 133.5 (*ipso*-Ar-C), 130.3, *para*-Ar-C), 129.9 (2C, *ortho*-Ar-C or *meta*-Ar-C), 123.8 (2C, *ortho*-Ar-C or *meta*-Ar-C), 84.3 (C19), 71.5 (C18), 33.8 (C21H), 22.6 ($C17H_2$), 18.8 ($C22H_2$), 11.2 (C20H); **HRMS** (+ESI) Found $[M+H]^+$ = 291.0484; $C_{13}H_{12}N_4SCl$ requires 291.0471, Δ 4.47 ppm

***tert*-Butyl(((1*E*,4*R*,5*E*,7*E*)-10-((1*S*,2*R*)-2-chlorocyclopropyl)-1-iodo-2-methyldeca-1,5,7,-trien-9-yn-4-yl)oxy)dimethylsilane 55**

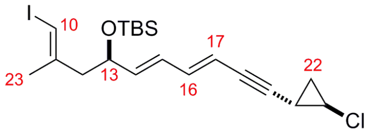

Procedure 1

To a stirred solution of **57** (63 mg, 0.482 mmol) in CH_2Cl_2 (5 mL) at RT was added MnO_2 (419 mg, 4.82 mmol) portionwise over 2 h until TLC analysis (hexane:Et_2O, 3:2) indictated the reaction was complete (compound **27**). The reaction mixture was filtered through oven dried SiO_2 and washed with CH_2Cl_2 (10 mL). The filtrate was partially concentrated *in vacuo* (400 mbar, 30 °C)[‡] to a volume of *ca*. 3 mL then DMF (1 mL) was added and the solution was further concentrated *in vacuo* (400 mbar, 30 °C) to remove the remaining CH_2Cl_2. The resulting aldehyde **28** solution in DMF was then dried over 4 Å molecular sieves.

In a separate flask **41b** (185 mg, 0.321 mmol) was dissolved in DMPU (2 mL) at RT and oven dried Cs_2CO_3 (209 mg, 0.642 mmol) added. The aldehyde **28** solution was immediately cannulated into the reaction and rinsed through the cannula with additional DMPU (0.5 mL). After stirring for 15 h, the reaction was quenched with sat. aq. NH_4Cl (5 mL) and diluted with Et_2O (10 mL). The layers were separated and the aqueous phase extracted with Et_2O (10 mL). The combined organic layers were washed with sat. aq. LiCl (4 × 5 mL), brine (5 mL), dried (Na_2SO_4) and concentrated *in vacuo*. Purification by column chromatography (SiO_2,

hexane:Et_2O, 99:1→95:5) gave the title compound **41b** (79.6 mg, 52%) as a colourless oil [1:1 mixture of *E*:*Z* isomers at C16/C17].

Procedure 2

To a stirring solution of oxalyl chloride (0.26 mL, 3.0 mmol) in CH_2Cl_2 (10 mL) at –78 °C was added DMSO (0.43 mL, 6.0 mmol) dropwise. The reaction mixture was stirred for 10 min at –78 °C and a solution of **63** (0.71 g, 2.0 mmol) in CH_2Cl_2 (5 mL +1 mL rinse) was added dropwise. The mixture was stirred for 20 min at –78 °C at which point Et_3N (1.4 mL, 10.0 mmol) was added dropwise. The suspension was allowed to warm to RT over 30 min after which sat. aq. NH_4Cl (20 mL) was added and the layers separated. The organic layer was washed with sat. aq. NH_4Cl (3 × 20 mL), brine (30 mL), dried ($MgSO_4$), and concentrated *in vacuo* to give crude aldehyde **64** (0.71 g, assumed quant.) as a colourless oil.

To a stirring solution of phosphonate **66** (0.55 g, 2.0 mmol) in THF (8 mL) at –78 °C was added LiHMDS (1 M in THF, 2.1 mL, 2.1 mmol) dropwise. The solution was stirred for 1 h at –78 °C before a solution of aldehyde **64** (0.71 g) was added as solution in THF (3 mL + 1 mL rinse). After stirring for 3 h, the reaction was warmed to RT, diluted with EtOAc (35 mL) and quenched by the addition of NH_4Cl (20 mL). The aqueous layer was separated and extracted with EtOAc (2 × 20 mL). The combined organic layers were dried ($MgSO_4$), filtered and concentrated *in vacuo* to afford a pale yellow oil. Purification by column chromatography (SiO_2, hexane) gave the title compound **55** (0.76 g, 80%) as a colourless oil [4:1 mixture of *E*:*Z* isomers at C14/C15].

Procedure 3

To a solution of vinyl silane **67** (347 mg, 0.82 mmol) in MeCN (51 mL) at RT was added NIS (277 mg, 1.23 mmol) in one portion. The reaction mixture was stirred in the absence of light for 90 min, after which it was quenched by the addition of sat. aq. $Na_2S_2O_3$ (50 mL) and diluted with pentane (100 mL) to give a triphasic system. The top layer was collected and the remaining other layers further extracted with pentane (3 × 120 mL). The combined top layers were dried ($MgSO_4$) and concentrated *in vacuo*. Purification by column chromatography (SiO_2, hexane) gave the title compound **55** (330 mg, 84%) as a colourless oil.

***R*$_f$** = 0.23 (pentane:Et_2O, 99:1); $[\alpha]_D^{25.9}$ = –88.9 (*c* = 1.08, $CHCl_3$); **IR** (film) νmax/cm-1 2955, 2928, 2856, 1472, 1463, 1429, 1361, 1255, 1143, 1107, 1070, 983, 925, 834; **^{1}H NMR** ($CDCl_3$, 500 MHz) δ = 6.47 (1H, dd, *J* = 15.5, 10.9 Hz, C16H), 6.15 (1H, dd, *J* = 15.2, 10.9 Hz, C15H), 5.90 (1H, s, C10H), 5.68 (1H, dd, *J* = 15.2, 6.2 Hz, C14H), 5.50 (1H, dd, *J* = 15.6, 1.8 Hz, C17H), 4.25–4.20 (1H, m, C13H), 3.18–3.14 (1H, m, C21H), 2.39 (1H, dd, *J* = 13.4, 7.7 Hz, C12H_AH_B), 2.30 (1H, dd, *J* = 13.4, 4.8 Hz, C12H_AH_B), 1.82 (3H, s, C23H_3), 1.81–1.76 (1H, m, C20H), 1.29–1.25 (2H, m, C22H_AH_B), 0.86 (9H, s, C(CH_3)$_3$ of *t*Bu), 0.00 (3H, s, Si(CH_3)$_2$), –0.03 (3H, s, Si(CH_3)$_2$); **^{13}C NMR** ($CDCl_3$, 126 MHz) δ = 143.9 (C11), 140.9 (C16H), 138.4 (C14H), 128.7 (C15H), 110.8 (C17H), 91.5 (C19), 78.0 (C10H), 77.7 (C18), 71.0 (C13H), 48.1 (C12H_2), 34.3 (C21H), 25.8 (3C, C(*C*H_3)$_3$ of *t*Bu), 24.5 (C23H_3), 19.3 (C22H_2), 18.1 (*C*(CH_3)$_3$ of *t*Bu), 12.1 (C20H), –4.5 (Si(*C*H_3)$_2$), –4.9 (Si(*C*H_3)$_2$); An accurate mass could not be obtained for this compound.

Key nOe observation:

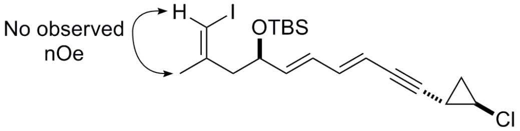

No observed nOe suggests that TMS-iodine exchange has occurred with retention of configuration.

(*R*,*E*)-2-((*tert*-Butyldimethylsilyl)oxy)-5-iodo-4-methylpent-4-en-1-ol 63

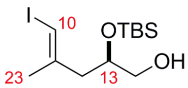

To a solution of **62**[14] (1.21 g, 5.0 mmol) in pyridine (10 mL) at 0 °C was added pivaloyl chloride (0.62 mL, 5.3 mmol) dropwise. The reaction was stirred for 30 min, diluted with Et_2O (30 mL), and quenched with 2 M HCl (30 mL). The aqueous layer was separated and extracted with Et_2O (2 × 20 mL). The combined organic layers were dried ($MgSO_4$), filtered and concentrated *in vacuo* to afford a colourless oil. The crude alcohol was then dissolved in CH_2Cl_2 (10 mL), 2,6-lutidine added (1.16 mL, 10.0 mmol) and cooled to –78 °C. TBSOTf (1.22 mL, 5.30 mmol) was

then added dropwise and the reaction was stirred for 1 h. The reaction was quenched with 1 M HCl (20 mL), diluted with CH_2Cl_2 (15 mL) and warmed to RT. The aqueous layer was separated and extracted with CH_2Cl_2 (2 × 15 mL). The combined organic layers were dried ($MgSO_4$), filtered and concentrated *in vacuo* to afford a colourless oil. The crude was then dissolved in CH_2Cl_2 (30 mL), cooled to –78 °C and diisobutylaluminium hydride (1 M in CH_2Cl_2, 10.0 mL, 10.0 mmol) added dropwise. After 1 hr the reaction was quenched by the addition of MeOH (5 mL) and warmed to RT. The reaction was diluted with CH_2Cl_2 (20 mL) and sat. aq. Rochelles salt added (50 mL). After stirring for 2 h, the aqueous layer was separated and extracted with CH_2Cl_2 (2 × 20 mL). The combined organic layers were dried ($MgSO_4$), filtered and concentrated *in vacuo* to afford a colourless oil. Purification by column chromatography [SiO_2, petroleum ether (40–60):EtOAc, 9:1] gave the title compound **63** (1.43 g, 80% over 3 steps) as a colourless oil.

$\boldsymbol{R_f}$ = 0.23 [petroleum ether (40–60):EtOAc, 9:1]; $[\alpha]_D^{25.0}$ = –3.0 (*c* = 1.20, $CHCl_3$); **IR** (film) ν_{max}/cm^{-1} 3403, 2928, 2857, 2342, 1471, 1361, 1252, 1104, 1042, 960, 834; **^{1}H NMR** ($CDCl_3$, 400 MHz) δ = 5.97–5.96 (1H, m, C10H), 3.87–3.82 (1H, m, C13H), 3.53 (1H, dd, *J* = 11.0 Hz, 4.0 Hz, C14H_AH_B), 3.42 (1H, dd, *J* = 11.0 Hz, 4.0 Hz, C14H_AH_B), 2.41 (2H, d, *J* = 8.0 Hz, C12H_2), 1.85 (3H, m, C23H_3), 0.91 (9H, s, C(CH_3)$_3$ of *t*Bu), 0.07 (3H, s, Si(CH_3)), 0.06 (3H, s, Si(CH_3)); **^{13}C NMR** ($CDCl_3$, 100 MHz) δ = 144.0 (C11), 78.0 (C10H), 70.6 (C13H), 65.9 (C14H_2), 44.0 (C12H_2), 25.9 (3C, C(*C*H_3)$_3$ of *t*Bu), 24.4 (C23H_3) 18.0 (*C*(CH_3)$_3$ of *t*Bu), –4.6 (2C, Si(*C*H_3)$_2$); **HRMS** (+ESI) Found $[M–H]^+$ = 355.0603; $C_{12}H_{24}O_2ISi$ requires 355.596, Δ 2.10 ppm.

Diethyl ((*E*)-5-((1*R*,2*S*)-2-chlorocyclopropyl)pent-2-en-4-yn-1-yl)phosphonate 66

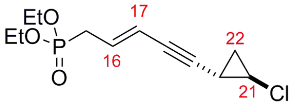

To a 50 mL round-bottomed flask was added **48** (1.17 g, 4.5 mmol), DMF (5 mL) and TBAF (1 M in THF, 12.5 mL, 12.5 mmol). The reaction vessel was sealed, and the mixture heated at 65 °C for 90 min. The reaction mixture was cooled to RT after which brine (7 mL) was added and the mixture extracted with pentane (5 × 20 mL). The combined organic layers were dried ($MgSO_4$) and concentrated *in vacuo* (400 mbar, RT) to give the crude alkynyl bromide (**86**) (assumed quant.). The crude was dissolved in THF (12 mL) and stannane **65**[15] (2.10 g, 4.5 mmol), $AsPh_3$ (98.0 mg, 0.32 mmol) and Pd_2dba_3 (73.3 mg, 0.08 mmol) were added. After stirring for 16 hr at RT, the reaction was concentrated *in vacuo*. Purification by column chromatography [SiO_2, EtOAc:petroleum ether (40–60), 7:3] gave the title compound **66** (0.70 g, 56% over 2 steps) as a clear oil.

R$_f$ = 0.15 [petroleum ether (40–60):EtOAc, 3:7]; $[\alpha]_D^{25.0}$ = –83.0 (*c* = 1.70, $CHCl_3$); **IR** (film) ν_{max}/cm^{-1} 2932, 2246, 1393, 1252, 1026, 957, 904; **^{1}H NMR** ($CDCl_3$, 400 MHz) δ = 5.99 (1H, dt, *J* = 15.7 Hz, 7.7 Hz, C16H), 5.56 (1H, dd, *J* = 15.7 Hz, 5.5 Hz, C17H), 4.15–4.07 (4H, m, 2 × OCH_2CH_3), 3.18–3.15 (1H, m, C21H), 2.66 (1H, dd, *J* = 7.9Hz, 1.3 Hz, C15H_AH_B), 2.60 (1H, dd, *J* = 7.9Hz, 1.3 Hz, C15H_AH_B), 1.79–1.74 (1H, m, C20H), 1.34–1.25 (8H, m, 2 × OCH_2CH_3 and C22H_AH_B), **^{13}C NMR** ($CDCl_3$, 100 MHz) δ = 132.2 (d, *J* = 12.7 Hz, C16H), 114.2 (d, *J* = 16.0 Hz, C17H), 89.0 (C19), 76.4 (d, *J* = 5.5Hz, C18), 62.1 (d, *J* = 6.6 Hz, OCH_2CH_3), 34.1 (C15H_2), 31.8 (C21H), 19.1 (C22H_2), 16.4 (d, *J* = 6.0 Hz, OCH_2CH_3), 11.8 (C20H); **HRMS** (+ESI) Found $[M+H]^+$ = 277.0774; $C_{12}H_{18}ClO_3P$ requires 277.0760, Δ 4.60 ppm.

((1*E*)-2-Iodoprop-1-en-1-yl)(trimethyl)silane 73[16]

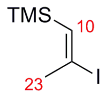

To a solution of catalyst **71**[17] (4.18 g, 11.8 mmol) in THF (233mL) at RT was added 1-(trimethylsilyl)-1-propyne (35.9 mL, 242.6 mmol) followed by the dropwise addition of Bu_3SnH (63.3 mL, 235.5 mmol). The dark reaction mixture was stirred for 40 min after which the mixture was concentrated *in vacuo*. Purification by column chromatography (SiO_2, hexane) gave the desired hydrostannylated product as well as Bu_3SnH as an inseparable mixture (83.3 g). The crude mixture was separated into 2 batches (40 g and 43.3 g) and used directly in the next step in the reaction sequence.

To a solution of the crude mixture (40 g) in CH_2Cl_2 (300 mL) at 0 °C was added a solution of I_2 in CH_2Cl_2 (0.0262 M, 119.1 mmol) *via* cannula dropwise until a dark purple colour persisted. The mixture was immediately quenched with 20% aq. $Na_2S_2O_3$ (60 mL) and warmed to RT. The layers were separated and the aqueous layer further extracted with CH_2Cl_2 (250 mL). The combined organic layers were dried ($MgSO_4$) and concentrated *in vacuo*. Purification by column chromatography (SiO_2, hexane) gave the title compound **73** (17.5 g, 62% over 2 steps) as a colourless oil.‡

$\boldsymbol{R_f}$ = 0.56 (hexane); **IR** (film) ν_{max}/cm^{-1} 2921, 2955, 2921, 2901, 2853, 1592, 1430, 1409, 1373, 1248, 1050, 955, 835; **^{1}H NMR** ($CDCl_3$, 400 MHz) δ = 6.20 (1H, s, C10H), 2.54 (3H, s, C23H_3), 0.15 (9H, s, C10HSi(C*H*$_3$)$_3$); **^{13}C NMR** ($CDCl_3$, 100 MHz) δ = 144.3 (C10H), 110.0 (C11), 34.7 (C23H_3), –0.2 (3C, C10HSi(*C*H_3)$_3$); An accurate mass could not be obtained for this compound. All spectroscopic data in agreement with that previously published.

‡ Purification often had to be repeated due to the similar $\boldsymbol{R_f}$ of vinyl iodide **73** and the tin byproducts.

(2*R*,4*E*)-4-Methyl-5(trimethylsilyl)pent-4-ene-1,2-diol 75

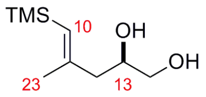

To a solution of thiophene (0.33 mL, 4.16 mmol) in THF (4.9 mL) at –78 °C was added *n*BuLi (2.33 M in hexanes, 1.79 mL, 4.16 mmol) dropwise and the pale yellow solution stirred for 40 min. The resulting thienyllithium solution was added to a suspension of CuCN (0.37 g, 4.16 mmol) in THF (4.9 mL) at –40 °C *via* cannula providing a bright yellow solution of Li(2-Th)CuCN. In the meantime, *t*BuLi (1.6 M in pentane, 4.68 mL, 7.49 mmol) was added to a solution of vinyl iodide **73**[†] (1.0 g, 4.16 mmol) in Et_2O (18 mL) at –90 °C and the pale yellow solution stirred for 40 min. After this time the Li(2-Th)CuCN solution (–40 °C) was added to the vinyllithium solution (–90 °C) dropwise *via* cannula and then warmed to –40 °C. After 1 h at –40 °C a solution of (*S*)-glycidol (**61**) (55 µL, 0.832 mmol) in Et_2O (8.3 mL) was added, followed immediately by BF_3•OEt_2 (0.15 mL, 1.25 mmol). The mixture was stirred for 90 min ensuring that the temperature did not rise above –25 °C, and then quenched by the addition of NH_4Cl–NH_3 (2:1, 25 mL) and allowed to warm to RT. The layers were separated and the aqueous layer further extracted with EtOAc (4 × 50 mL). The combined organic layers were dried ($MgSO_4$) and concentrated *in vacuo*. Purification by column chromatography (SiO_2, EtOAc:hexane, 7:3) gave the title compound **75** (113 mg, 72%) as a colourless oil.

$\boldsymbol{R_f}$ = 0.47 (EtOAc); $[\alpha]_D^{27.6}$ = +4.2 (*c* = 0.78, $CHCl_3$); **IR** (film) ν_{max}/cm^{-1} 3361, 2953, 2901, 1617, 1437, 1409, 1378, 1247, 1147, 1091, 1037; **^{1}H NMR** ($CDCl_3$, 400 MHz) δ = 5.34 (1H, s, C10H), 3.91–3.84 (1H, m, C13H), 3.71–3.64 (1H, m, C14H_AH_B), 3.52–3.45 (1H, m, C14H_A*H_B*), 2.27–2.22 (2H, m, C12H_AH_B), 2.00 (1H, d, *J* = 3.0 Hz, C13HO*H*), 1.90 (1H, t, *J* = 5.9 Hz, C14H_2O*H*), 1.83 (3H, s, C23H_3), 0.2 (9H, s, C10HSi(CH_3)$_3$); **^{13}C NMR** ($CDCl_3$, 100 MHz) δ = 150.9 (*C*11), 128.1 (*C*10H), 69.5 (C13HOH), 66.6 (C14H_2), 46.7 (C12H_2), 21.8 (C23H_3), –0.1 (3C, C10HSi(CH_3)$_3$); **HRMS** (+ESI) Found $[M+Na]^+$ = 211.1129; $C_9H_{20}O_2SiNa$ requires 211.1125, Δ 1.89 ppm.

[†] The volatile vinyl iodide was dried by stirring with 4 Å MS for 1 h in Et_2O, and then separated by filtration under inert atmosphere directly before use.

(2*R*)-4,4-Dimethylpentane-1,2-diol 77

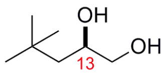

Variable amounts of by-product **77** (colourless oil) were isolated from the previous procedure (for **75**) when less than 5.0 equiv of vinyl iodide **73** were used.

***R*f** = 0.74 (Et_2O:hexane, 1:1), $[\alpha]_D^{25.9}$ = +20.0 (*c* = 0.20, $CHCl_3$); **IR** (film) ν_{max}/cm^{-1} 3347, 2952, 2869, 1467, 1415, 1395, 1365, 1248, 1199, 1171, 1087, 1039, 1012; **^{1}H NMR** ($CDCl_3$, 400 MHz) δ = 3.88–3.81 (1H, m, C13H), 3.60–3.54 (1H, m, C14H_AH_B), 3.42–3.35 (1H, m, C14H_AH_B), 2.02–1.98 (2H, m, 2 × OH), 1.36 (1H, dd, *J* = 14.6, 7.7 Hz, C12H_AH_B), 1.27 (1H, dd, *J* = 14.6, 2.9 Hz, C12H_AH_B), 0.98 (9H, s, $C(CH_3)_3$ of *t*Bu); **^{13}C NMR** ($CDCl_3$, 100 MHz) δ = 69.9 (C13H), 68.1 (C14H_2), 46.9 (C12H_2), 30.1 ($C(CH_3)_3$ of *t*Bu), 30.0 (3C, $C(CH_3)_3$ of *t*Bu); **HRMS** (+ESI) Found $[M+Na]^+$ = 155.1049; $C_7H_{16}O_2Na$ requires 155.1043, Δ 3.87 ppm.

(2*R*)-4,4-Dimethylpentane-1,2-diyl bis(4-bromobenzoate) S16

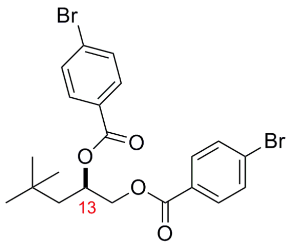

The structure by by-product **77** was confirmed by conversion to the above compound (**S16**).

To a solution of **77** (36.1 mg, 0.27 mmol) in CH_2Cl_2 (3 mL) at RT was added Et_3N (0.11 mL, 0.79 mmol) followed by DMAP (2 crystals) and 4-bromobenzoyl chloride (70.0 mg, 0.320 mmol). The reaction mixture was stirred for 3 h, before more Et_3N (0.11 mL, 0.79 mmol), DMAP (2 small crystals) and 4-bromobenzoyl chloride (70 mg, 0.320 mmol) were added. After a further 16 h and quenched by the addition of sat. aq. NH_4Cl (5 mL). The layers were separated and the aqueous layer further extracted with EtOAc (3 × 10 mL) and the combined organic layers

washed with brine (20 mL), dried ($MgSO_4$), and concentrated *in vacuo*. Purification by column chromatography (SiO_2, hexane:EtOAc, 9:1) gave the title compound **S16** (104 mg, 77%) as a white solid.

***R*f** = 0.85 (EtOAc:hexane, 7:3); **m.p. =** 101–104 °C; $[\alpha]_D^{28.5}$ = –31.1 (*c* = 0.75, $CHCl_3$); **IR** (film) ν_{max}/cm^{-1} 2952, 2869, 1713, 1590, 1483, 1398, 1367, 1301, 1266, 1206, 1172, 1137, 1116, 1101, 1013, 995; **^{1}H NMR** ($CDCl_3$, 400 MHz) δ = 7.88 (2H, d, *J* = 8.5 Hz, Ar), 7.82 (2H, d, *J* = 8.5 Hz, Ar), 7.57 (2H, d, *J* = 8.5 Hz, Ar), 7.55 (2H, d, *J* = 8.5 Hz, Ar), 5.68–5.61 (1H, m, C13H), 4.49 (1H, dd, *J* = 11.7, 3.6 Hz, C14H_AH_B), 4.34 (1H, dd, *J* = 11.7, 7.2 Hz, C14H_AH_B), 1.83 (1H, dd, *J* = 14.9, 8.6 Hz, C12H_AH_B), 1.59 (1H, dd, *J* = 14.8, 2.7 Hz, C12H_AH_B), 0.98 (9H, C(CH_3)$_3$ of *t*Bu); **^{13}C NMR** ($CDCl_3$, 100 MHz) δ = 165.5 (C=O), 165.3 (C=O), 131.8 (2C, *C*H of Ar), 131.8 (2C, *C*H of Ar), 131.1 (2C, *C*H of Ar), 131.1 (2C, *C*H of Ar), 129.1 (quaternary Ar), 128.7 (quaternary Ar), 128.3 (quaternary Ar), 128.3 (quaternary Ar), 70.1 (C13H), 67.1 (C14H_2), 44.2 (C12H_2), 30.1 (*C*(CH_3)$_3$ of *t*Bu), 29.8 (3C, C(*C*H_3)$_3$ of *t*Bu); **HRMS** (+ESI) Found $[M+Na]^+$ = 518.9784; $C_{21}H_{22}O_4Br_2Na$ requires 518.9777, Δ 1.35 ppm.

The structure and absolute stereochemistry was confirmed by X-ray crystallographic analysis after crystallisation from analytical grade Et_2O.

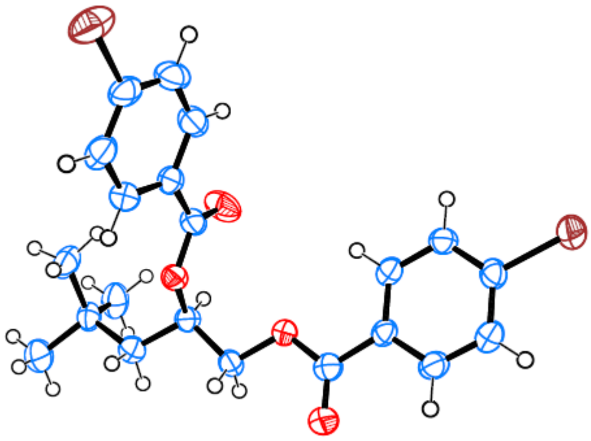

CCDC 882398 contains the supplementary crystallographic data for this thesis. This data can be obtained free of charge from the Cambridge Crystallographic Data Centre *via* www.ccdc.cam.ac.uk/data_request/cif.

(2*R*)-2-(((4-Methoxybenzyl)oxy)methyl)oxirane 74[18]

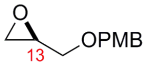

To a suspension of NaH (60% in mineral oil, 8.92 g, 223 mmol) in DMF (260 mL) cooled to 0 °C was added *p*-methoxybenzyl chloride (30.2 mL, 223 mmol) dropwise over 25 min. The mixture was stirred for 25 min at 0 °C after which (*S*)-glycidol (15.0 g, 202.5 mmol) was added dropwise over 25 min *via* syringe. The mixture was then allowed to warm to RT slowly over 16 h after which it was poured into a separating funnel containing NH_4Cl (400 mL) and EtOAc (700 mL). The layers were separated and the organic layer washed with 10% aq. $NaHCO_3$ (400 mL) and H_2O (400 mL). The combined aqueous layers were then further extracted with EtOAc (500 mL) and the combined organic layers dried ($MgSO_4$) and concentrated *in vacuo*. Purification by column chromatography (SiO_2, hexane:EtOAc, 9:1→3:2) gave the title compound **74** (38.7 g, 98%) as a colourless oil.

$\boldsymbol{R_f}$ = 0.18 (hexane:EtOAc, 4:1); $[\alpha]_D^{24.1}$ = +3.5 (*c* = 0.86, $CHCl_3$), [lit.[22] $[\alpha]_D^{23}$ +3.5 (*c* = 1.00, $CHCl_3$)]; **IR** (film) ν_{max}/cm^{-1} 3000, 1905, 2837, 1612, 1586, 1512, 1464, 1443, 1384, 1335, 1302, 1244, 1174, 1086, 1032; **^{1}H NMR** ($CDCl_3$, 400 MHz) δ = 7.27 (2H, d, *J* = 10.1 Hz, $(CH)_2COCH_3$ of PMB), 6.88 (2H, d, *J* = 8.6 Hz, $C(CH)_2$ of PMB), 4.55 (1H, d, *J* = 11.5 Hz, $OCH_AH_BPh(OCH_3)$), 4.49 (1H, d, *J* = 11.5 Hz, $OCH_AH_BPh(OCH_3)$), 3.81 (3H, s, OCH_3), 3.73 (1H, dd, *J* = 11.4, 3.1 Hz, $C14H_AH_B$), 3.42 (1H, dd, *J* = 11.4, 5.8 Hz, $C14H_AH_B$), 3.20–3.15 (1H, m, C13H), 2.79 (1H, t, *J* = 4.7 Hz, $C12H_AH_B$), 2.61 (1H, dd, *J* = 5.0, 2.7 Hz, $C12H_AH_B$); **^{13}C NMR** ($CDCl_3$, 100 MHz) δ = 159.3 ($C(OCH_3)$ of PMB), 130.0 ($CCH_2CH_2C(OCH_3)$ of PMB), 129.5 (2C, $CCH_2CH_2C(OCH_3)$ of PMB), 113.9 (2C, $CCH_2CH_2C(OCH_3)$ of PMB), 73.0 (OCH_2), 70.6 ($C14H_2$), 55.3 (OCH_3 of PMB), 50.9 (C13H), 44.4 ($C12H_2$); **HRMS** (+ESI) Found $[M+Na]^+$ = 217.0843; $C_{11}H_{14}O_3Na$ requires 217.0835, Δ 3.69 ppm.

***tert*-Butyl(((2*R*,4*E*)-1-((4-methoxybenzyl)oxy)-4-methyl-5-(trimethylsilyl)pent-4-en-2-yl)dimethylsilane S17**

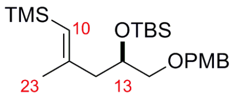

To a solution of vinyl iodide **73**[†] (12.7 g, 53.0 mmol) in PhMe (530 mL) at –78 °C was added *t*BuLi (1.28 M in pentane, 82.8 mL, 105.9 mmol) dropwise and the pale yellow solution stirred for 45 min. After cooling to –85 °C (internal), a solution of epoxide **74** (25.7 g, 132.4 mmol) in PhMe (150 mL) was added dropwise to the vinyl lithium solution *via* cannula, followed immediately by BF_3•OEt_2 (16.3 mL, 132.4 mmol) ensuring that the temperature did not exceed –70 °C. The reaction was maintained at –78 °C for 2 h and then quenched with sat. aq. $NaHCO_3$ (450 mL), diluted with EtOAc (450 mL) and slowly warmed to RT. The layers were separated and the aqueous layer further extracted with EtOAc (3 × 400 mL). The combined organic layers were dried ($MgSO_4$) and concentrated *in vacuo*. Purification by column chromatography (SiO_2, dry load, hexane:EtOAc, 8:2) gave the desired alcohol (**76**) (9.71 g, 31.5 mmol) which was used directly in the next step of the reaction sequence.

To a solution of alcohol **76** (9.71 g, 31.5 mmol) in CH_2Cl_2 (315 mL) at –78 °C was added 2,6-lutidine (8.1 mL, 69.4 mmol) and TBSOTf (8.0 mL, 34.7 mmol) sequentially. The reaction mixture was stirred for 90 min at –78 °C and H_2O (200 mL) added. The layers were separated and the aqueous layer further extracted with CH_2Cl_2 (4 × 150 mL). The combined organic layers were dried ($MgSO_4$) and concentrated *in vacuo*. Purification by column chromatography (SiO_2, hexane→hexane:EtOAc, 95:5) gave the title compound **S17** (11.8 g, 53% over 2 steps) as a colourless oil.

$\boldsymbol{R_f}$ = 0.48 (hexane:EtOAc, 95:5); $[\alpha]_D^{26.8}$ = +5.8 (*c* = 1.19, $CHCl_3$); **IR** (film) ν_{max}/cm^{-1} 2953, 2929, 2856, 1615, 1513, 1464, 1362, 1302, 1246, 1172, 1087, 1038, 1005, 971; **^{1}H NMR** ($CDCl_3$, 400 MHz) δ = 7.25 (2H, d, *J* = 10.1 Hz, $(CH)_2COCH_3$ of PMB), 6.87 (2 H, d, *J* =

[†] The vinyl iodide was used directly without any drying techniques due to its volatility.

8.6 Hz, C(C*H*)$_2$ of PMB), 5.25 (1H, s, C10H), 4.45 (2H, s, OCH_2Ar(OCH_3)), 3.99–3.93 (1H, m, C13H), 3.81 (3H, s, OCH_3), 3.36 (1H, dd, *J* = 9.2, 5.2 Hz, C14H_AH$_B$), 3.32 (1H, dd, *J* = 9.5, 5.3 Hz, C14H$_A$$H_B$), 2.33 (1H, dd. *J* = 13.2, 4.8 Hz, C12H_AH$_B$), 2.17 (1H, dd, *J* = 13.4, 7.5 Hz, C12H$_A$$H_B$), 1.79 (3H, s, C23H$_3$), 0.87 (9H, s, C(C$H_3$)$_3$ of *t*Bu), 0.08 (9H, s, C10HSi(CH_3)$_3$), 0.04 (3H, s, Si(CH_3)$_2$), 0.03 (3H, s, Si(CH_3)$_2$); **^{13}C NMR** (CDCl$_3$, 100 MHz) δ = 159.1 (*C*(OCH$_3$) of PMB), 151.9 (C11), 130.6 (*C*CH$_2$CH$_2$C(OCH$_3$) of PMB), 129.2 (C*C*H$_2$CH$_2$C(OCH$_3$) of PMB), 126.8 (C10H), 113.7 (C*C*H$_2$CH$_2$C(OCH$_3$) of PMB), 74.5 (C14H$_2$), 73.0 (O*C*H$_2$Ph(OCH$_3$)), 70.6 (C13H), 55.3 (OCH$_3$ of PMB), 48.3 (C12H$_2$), 25.9 (3C, C(*C*H$_3$)$_3$ of *t*Bu), 22.3 (C23H$_3$), 18.2 (*C*(CH$_3$)$_3$ of *t*Bu), 0.1 (3C, C10HSi(*C*H$_3$)$_3$), –4.3 (Si(*C*H$_3$)$_2$), –4.7 (Si(*C*H$_3$)$_2$); **HRMS** (+ESI) Found $[M+H]^+$ = 423.2759; $C_{23}H_{43}O_3Si_2$ requires 423.2751, Δ 1.89 ppm and $[M+Na]^+$ = 445.2581; $C_{23}H_{42}O_3Si_2Na$ requires 445.2570, Δ 2.47 ppm.

(2*R*,4*E*)-2-((*tert*-Butyl(dimethyl)silyl)oxy)-4-methyl-5-(trimethylsilyl)pent-4-en-1-ol 79

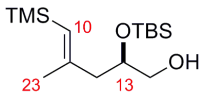

To a solution of **S17** (11.8 g, 28.0 mmol) in CH_2Cl_2 (340 mL) at 0 °C was added pH 7 phosphate buffer (35 mL) followed by DDQ (8.89 g, 39.2 mmol). The reaction mixture was stirred for 40 min, after which another portion of DDQ (3.18 g, 14.0 mmol) was added. After an additional 40 min at 0 °C the mixture allowed to warm to RT, diluted with CH_2Cl_2 (150 mL) and sat. aq. $NaHCO_3$ (400 mL) added. The layers were separated and the organic layer further washed with H_2O (400 mL), dried ($MgSO_4$) and filtered. To the resulting filtrate was added BZA resin (30.5 g, 5.5 mmol/g, 167.9 mmol) and the mixture stirred for 90 min. The resin was removed by filtration and the reaction mixture concentrated *in vacuo*. Purification by column chromatography (SiO_2, hexane→hexane:Et_2O, 9:1) gave the title compound **79** (7.50 g, 89%) as a colourless oil.

$\boldsymbol{R_f}$ = 0.28 (hexane:Et_2O, 9:1); $[\alpha]_D^{28.6}$ = –6.8 (*c* = 0.83, $CHCl_3$); **IR** (film) ν_{max}/cm^{-1} 3381, 2954, 2930, 2858, 1618, 1473, 1463, 1439, 1387, 1378, 1362, 1248, 1105, 1072, 1043, 1005; **^{1}H NMR** ($CDCl_3$, 400 MHz) δ = 5.27 (1H, s, C10H), 3.93–3.87 (1H, m, C13H), 3.60–3.53 (1H, m, C14H_AH_B), 3.47–3.40 (1H, m, C14H_AH_B), 2.33–2.21 (2H, m, C12H_AH_B), 1.86 (1H, t, *J* = 6.4 Hz, C14H_2O*H*), 1.80 (3H, s, C23H_3), 0.90 (9H, s, C(CH_3)$_3$ of *t*Bu), 0.10–0.09 (15H, br s, Si(CH_3)$_2$ and CHSi(CH_3)$_3$); **^{13}C NMR** ($CDCl_3$, 100 MHz) δ = 151.1 (C11), 127.4 (C10H), 71.6 (C13H), 66.3 (C14H_2), 47.7 (C12H_2), 25.9 (3C, C(*C*H_3)$_3$ of *t*Bu), 22.3 (C23H_3), 18.1 (*C*(CH_3)$_3$ of *t*Bu), 0.0 (3C, C10HSi(*C*H_3)$_3$), –4.4 (Si(*C*H_3)$_2$), –4.5 (Si(*C*H_3)$_2$); **HRMS** (+ESI) Found $[M+Na]^+$ = 325.2005; $C_{15}H_{34}O_2Si_2Na$ requires 325.1995, Δ 3.08 ppm; **Elemental Analysis** found C, 59.59; H, 11.32. $C_{15}H_{34}O_2Si_2$ requires C, 59.54; H, 11.33%.

***tert*-Butyl(((3*R*,5*E*)-1,1-dibromo-5-methyl-6-(trimethylsilyl)hexa-1,5-dien-3-yl)oxy)dimethylsilane 81**

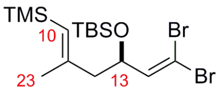

To a solution of **79** (3.77 g, 12.5 mmol) and Et_3N (17.4 mL, 124.7 mmol) in CH_2Cl_2 (80 mL) at 0 °C was added a solution of SO_3•pyr (9.9 g, 62.4 mmol) in DMSO (67 mL) dropwise *via* syringe over 15 min. The reaction mixture was stirred at 0 °C for 30 min, after which it was warmed to RT slowly over 1 h and diluted with EtOAc:hexane (1:1, 566 mL). The mixture was washed with 1 *N* HCl (2 × 75 mL), sat. aq. $NaHCO_3$ (150 mL), brine (150 mL), dried ($MgSO_4$) and concentrated *in vacuo* to give aldehyde **80** (3.74 g, 99%). The aldehyde was carried through to the next step in the synthesis without further purification.

A solution of PPh_3 (13.08 g, 49.9 mmol) in CH_2Cl_2 (290 mL) was cooled to 0 °C and CBr_4 (8.27 g, 24.9 mmol) added in one portion. The resulting bright yellow solution was stirred for 15 min, during which time a separate solution of crude aldehyde **80** (3.74 g, 12.5 mmol) in CH_2Cl_2 (290 mL) was also cooled to 0 °C and 2,6-lutidine (2.92 mL, 25.1 mmol) added. The aldehyde solution was then transferred to the ylide dropwise over 20 min *via* cannula, and the mixture maintained at 0 °C for 1 h. The solution was quenched with sat. aq. NH_4Cl (290 mL), further diluted with CH_2Cl_2 (290 mL) and then warmed to RT. The layers were separated, and the aqueous layer further extracted with CH_2Cl_2 (300 mL). The combined organic layers were dried ($MgSO_4$) and concentrated *in vacuo*. Purification by column chromatography (SiO_2, dry load, hexane) gave the title compound **81** (5.03 g, 89% over 2 steps) as a colourless oil.

$\boldsymbol{R_f}$ = 0.50 (pentane); $[\alpha]_D^{26.2}$ = –11.8 (*c* = 1.00, $CHCl_3$); **IR** (film) ν_{max}/cm^{-1} 2955, 2928, 2856, 1618 (C=CBr_2), 1475, 1463, 1362, 1248, 1078, 1022; **^{1}H NMR** ($CDCl_3$, 400 MHz) δ = 6.36 (1H, d, *J* = 8.1 Hz, C14H), 5.26 (1H, s, C10H), 4.45–4.39 (1H, m, C13H), 2.27 (1H, dd, *J* = 13.0, 7.8 Hz, C12H_AH_B), 2.20 (1H, dd, *J* = 13.0, 5.3 Hz, C12H_AH_B), 1.82 (3H, s, C23H_3), 0.87 (9H, s, C(CH_3)$_3$ of *t*Bu), 0.10 (9H, s, C10HSi(CH_3)$_3$), 0.06 (3H, s, Si(CH_3)$_2$), 0.05 (3H, s, Si(CH_3)$_2$); **^{13}C NMR** ($CDCl_3$, 100 MHz) δ = 150.0 (C11), 142.0 (C14H), 128.1 (C10H), 88.2 (C15Br_2), 72.8

(C13H), 49.9 ($C12H_2$), 25.8 (3C, C(CH_3)$_3$ of *t*Bu), 22.4 ($C23H_3$), 18.1 (*C*(CH_3)$_3$ of *t*Bu), 0.0 (3C, C10HSi(CH_3)$_3$), –4.5 (Si(CH_3)$_2$), –4.9 (Si(CH_3)$_2$); **HRMS** (+ESI) Found $[M+Na]^+$ = 477.0260; $C_{16}H_{32}OSiBr_2Na$ requires 477.0251, Δ 1.89 ppm.

***tert*-Butyl(dimethyl)(((3*R*,5*E*)-5-methyl-6-(trimethylsilyl)hex-5-en-1-yn-3-yl)oxy)silane 82**

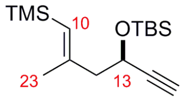

To a solution of **81** (8.10 g, 17.8 mmol) in THF (310 mL) at –78 °C was added *n*BuLi (2.21 M in hexanes, 16.9 mL, 37.4 mmol) dropwise resulting in the formation of a red-brown solution. After 40 min at –78 °C, the dry-ice/acetone bath was removed and the solution allowed to warm to RT over 1 h, during which time the solution once again became bright yellow. The reaction mixture was quenched with H_2O (300 mL) and diluted with pentane (400 mL). The layers were separated and the aqueous layer further extracted with pentane (2 × 300 mL). The combined organic layers were dried ($MgSO_4$) and concentrated *in vacuo*. Purification by column chromatography (SiO_2, hexane) gave the title compound **82** (4.48 g, 85%) as a colourless oil.

$\boldsymbol{R_f}$ = 0.38 (pentane); $[\alpha]_D^{28.5}$ = +34.2 (*c* = 0.75, $CHCl_3$); **IR** (film) ν_{max}/cm^{-1} 3313, 2955, 2930, 1858, 1619, 1475, 1464, 1377, 1362, 1342, 1249, 1088, 1005; ^{1}**H NMR** ($CDCl_3$, 400 MHz) δ = 5.31 (1H, s, C10H), 4.47 (1H, ddd, *J* = 7.7, 5.5, 2.0 Hz, C13H), 2.49–2.40 (2H, m, $C12H_AH_B$), 2.38 (1H, d, *J* = 2.0 Hz, C15H), 1.81 (3H, s, $C23H_3$), 0.89 (9H, s, C(CH_3)$_3$ of *t*Bu), 0.13 (3H, s, Si(CH_3)$_2$), 0.09 (12H, br s, Si(CH_3)$_2$ and C10HSi(CH_3)$_3$); ^{13}**C NMR** ($CDCl_3$, 100 MHz) δ = 150.3 (C11), 127.5 (C10H), 85.6 (C14), 72.1 (C15H), 62.0 (C13H), 51.5 ($C12H_2$), 25.8 (3C, C(CH_3)$_3$ of *t*Bu), 22.2 ($C23H_3$), 18.2 (*C*(CH_3)$_3$ of *t*Bu), 0.0 (3C, C10HSi(CH_3)$_3$), –4.6 (Si(CH_3)$_2$), –5.1 (Si(CH_3)$_2$); **HRMS** (+ESI) Found $[M+Na]^+$ = 319.1877; $C_{16}H_{32}OSi_2Na$ requires 319.1884, Δ 2.19 ppm.

***tert*-Butyl(dimethyl)(((1*E*,3*R*,5*E*)-5-methyl-1-(tributylstannanyl)-6-(trimethylsilyl)hex-1,5-dien-3-yl)oxy)silane 83**

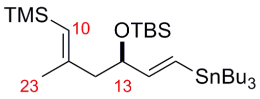

To a solution of **82** (52 mg, 0.176 mmol) in THF (5.9 mL) at 0 °C was added $Pd(PPh_3)_2Cl_2$ (3.7 mg, 5.28 μmol) in one portion, followed by the dropwise addition of Bu_3SnH (56 μL, 0.21 mmol) *via* syringe. The dark yellow solution was stirred at 0 °C for 2 h, after which it was allowed to warm to RT and the solvent removed *in vacuo*. Purification by column chromatography (SiO_2, hexane) gave the title compound **83** (57.8 mg, 56%) as a colourless oil.

$\boldsymbol{R_f}$ = 0.28 (hexane); $[\alpha]_D^{25.2}$ = +10.3 (*c* = 0.94, $CHCl_3$); **IR** (film) ν_{max}/cm^{-1} 2956, 2927, 2856, 1617, 1463, 1376, 1361, 1248, 1090, 1068; **^{1}H NMR** ($CDCl_3$, 400 MHz) δ = 6.04 (1H, d, *J* = 19.0 Hz, C15H), 5.93 (1H, dd, *J* = 19.0, 5.4 Hz, C14H), 5.24 (1H, s, C10H), 4.21–4.15 (1H, m, C13H), 2.27 (1H, dd, *J* = 13.0, 7.6 Hz, C12H_AH_B), 2.18 (1H, dd, *J* = 13.0, 5.5 Hz, C12H_AH_B), 1.80 (3H, s, C23H_3), 1.52–1.44 (6H, m, CH_2 of $SnBu_3$), 1.35–1.25 (6H, m, CH_2 of $SnBu_3$), 0.91–0.86 (24H, m, CH_2 of $SnBu_3$, CH_3 of $SnBu_3$ and $C(CH_3)_3$ of *t*Bu), 0.09 (9H, s, C10HSi$(CH_3)_3$), 0.03 (3H, s, $Si(CH_3)_2$), 0.02 (3H, s, $Si(CH_3)_2$); **^{13}C NMR** ($CDCl_3$, 100 MHz) δ = 151.9 (C11), 151.5 (C15H), 126.7 (C10H), 126.3 (C14H), 75.8 (C13H), 51.8 (C12H_2), 29.1 (3C, Bu), 27.3 (3C, Bu), 26.0 (3C, $C(CH_3)_3$ of *t*Bu), 22.5 (C23H_3), 18.4 ($C(CH_3)_3$ of *t*Bu), 13.7 (3C, Bu), 9.5 (3C, Bu), 0.1 (3C, C10HSi$(CH_3)_3$), –4.3 ($Si(CH_3)_2$), –4.8 ($Si(CH_3)_2$); An accurate mass could not be obtained for this compound, but a mass corresponding to the loss of butane was obtained **HRMS** (+EI) Found $[M–C_4H_9]^+$ = 531.2516; $C_{24}H_{51}OSi_2Sn$ requires 531.2495, Δ 3.95 ppm.

tert-Butyl(((1_E_,3_R_,5_E_)-1-iodo-5-methyl-6-(trimethylsilyl)hexa-1,5-dien-3-yl)oxy)dimethylsilane 84

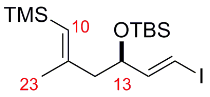

To a solution of **82** (4.48 g, 15.1 mmol) in THF (500 mL) at 0 °C was added $(PPh_3)_2PdCl_2$ (318 mg, 0.45 mmol) in one portion, followed by the dropwise addition of Bu_3SnH (10.2 mL, 37.8 mmol) *via* syringe. The dark yellow solution was stirred at 0 °C for 2 h, after which it was allowed to warm to RT and the solvent removed *in vacuo* to leave a dark residue.

The residue was re-dissolved in CH_2Cl_2 (250 mL) and cooled to –78 °C. A solution of I_2 in CH_2Cl_2 (0.079 M, 37.8 mmol) was added *via* cannula dropwise until a dark purple colour persisted. The mixture was immediately quenched with 20% aq. $Na_2S_2O_3$ (500 mL) and warmed to RT. The layers were separated and the aqueous layer further extracted with CH_2Cl_2 (3 × 500 mL). The combined organic layers were washed with 20% aq. $Na_2S_2O_3$ (500 mL), brine (500 mL), dried ($MgSO_4$) and concentrated *in vacuo* to give an orange oil. Purification by column chromatography (SiO_2, hexane) gave the title compound **84** (3.16 g, 49% over 2 steps) as a colourless oil.

$\boldsymbol{R_f}$ = 0.38 (hexane); $[\alpha]_D^{25.3}$ = +14.0 (*c* = 1.06, $CHCl_3$); **IR** (film) ν_{max}/cm^{-1} 2954, 2929, 2857, 1616, 1472, 1463, 1361, 1248, 1206, 1160, 1094, 1074, 1006, 940; **^{1}H NMR** ($CDCl_3$, 400 MHz) δ = 6.51 (1H, dd, *J* = 14.4, 6.0 Hz, C14H), 6.19 (1H, dd, *J* = 14.4, 0.8 Hz, C15H), 5.24 (1H, s, C10H), 4.24–4.18 (1H, m, C13H), 2.26 (1H, dd, *J* = 13.1, 7.3 Hz, C12H_AH_B), 2.18 (1H, dd, *J* = 13.1, 5.6 Hz, C12H_AH_B), 1.78 (3H, s, C23H_3), 0.88 (9H, s, C(CH_3)$_3$ of *t*Bu), 0.10 (9H, s, C10HSi(CH_3)$_3$), 0.04 (3H, s, Si(CH_3)$_2$), 0.03 (3H, s, Si(CH_3)$_2$); **^{13}C NMR** ($CDCl_3$, 100 MHz) δ = 150.5 (C11), 149.0 (C14H), 127.9 (C10H), 75.5 (C15H), 74.5 (C13H), 51.0 (C12H_2), 25.9 (3C, C(*C*H_3)$_3$ of *t*Bu), 22.5 (C23H_3), 18.2 (*C*(CH_3)$_3$ of *t*Bu), 0.0 (3C, C10HSi(*C*H_3)$_3$), –4.5 (Si(*C*H_3)$_2$), –4.9 (Si(*C*H_3)$_2$); **HRMS** (+ESI) Found $[M+Na]^+$ = 447.1028; $C_{16}H_{33}IOSi_2Na$ requires 447.1007, Δ 4.70 ppm; **Elemental Analysis** found C, 45.31; H, 7.75. $C_{16}H_{33}IOSi_2$ requires C, 45.27; H, 7.84%.

((*E*)-2-Iodoethenyl)(trimethyl)silane 91

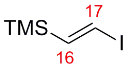

To stannane **87**[19] (308 mg, 0.79 mmol) in CH_2Cl_2 (2.4 mL) at 0 °C was added a solution of I_2 in CH_2Cl_2 (0.0787 M, 0.947 mmol) *via* cannula dropwise until a dark purple colour persisted. The mixture was immediately quenched with sat. aq. $Na_2S_2O_3$ (5 mL) and warmed to RT. The resulting dark mixture was extracted with CH_2Cl_2 (2 × 20 mL) and the combined organic layers dried ($MgSO_4$) and carefully concentrated *in vacuo* (400 mbar, RT). Purification by column chromatography (SiO_2, pentane) gave the title compound **91** (70.4 mg, 40%) as a colourless oil.

***R*f** = 0.58 (pentane); **IR** (film) ν_{max}/cm^{-1} 2956, 2898, 1542, 1412, 1247, 1146, 953, 835, 767; **^{1}H NMR** ($CDCl_3$, 400 MHz) δ = 7.08 (1H, d, *J* = 16.2 Hz, C16H), 6.68 (1H, d, *J* = 16.1 Hz, C17H), 0.09 (9H, s, Si(C*H*$_3$)$_3$); **^{13}C NMR** ($CDCl_3$, 100 MHz) δ = 150.5 (C16H), 89.5 (C17H), –1.5 (3C, Si(*C*H$_3$)$_3$); **HRMS** (+EI) Found [M]$^+$ = 225.9660; $C_5H_{11}ISi$ requires 225.9669, Δ 3.98 ppm.

((1*E*)-4-((1*S*,2*R*)-2-Chlorocyclopropyl)but-1-en-3-yn-1-yl)(trimethyl)silane 88

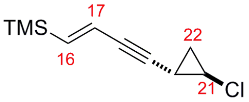

To a solution of dibromide **48** (417 mg, 1.60 mmol) in Et_2O at –78 °C was added *n*BuLi (2.16 M in hexanes, 1.76 mmol) dropwise, resulting in the formation of a yellow solution. The mixture was stirred for 15 min at this temperature after which it was warmed to 0 °C, stirred for a further 10 min and then re-cooled to –78 °C. Additional *n*BuLi (2.16 M in hexanes, 1.76 mmol) was added dropwise to the reaction mixture at –78 °C, stirred for 15 min and then once again warmed to 0 °C and stirred for 30 min. The now dark yellow solution was again re-cooled to –78 °C whereupon H_2O (20 mL) was added. The reaction was warmed to RT, extracted with Et_2O (3 × 50 mL) and the combined extracts washed with brine (50 mL) and dried ($MgSO_4$). The solvent was carefully removed by distillation at 65 °C using a vigreux column and the crude alkyne (**90**) (assumed quant.) used directly in the next step without further purification.

To a solution of crude alkyne **90** (assumed quant., 1.60 mmol), vinyl iodide **91** (145 mg, 0.64 mmol) in MeCN (12.8 mL) at RT was added $(PPh_3)_2PdCl_2$ (31.4 mg, 45 μmol) and CuI (25.6 mg, 0.134 mmol) sequentially. The mixture was cooled to 0 °C and Et_3N (0.39 mL, 2.82 mmol) added dropwise. The reaction was maintained at 0 °C for 1 h in the absence of light and then warmed to RT, diluted with pentane (10 mL) and quenched with pH 7 phosphate buffer (10 mL). The mixture was extracted with pentane (3 × 20 mL) and the combined organics washed with H_2O (30 mL), brine (30 mL), dried ($MgSO_4$) and carefully concentrated *in vacuo* (400 mbar, RT). Purification by column chromatography (SiO_2, pentane) gave the title compound **88** (104 mg, 82% from vinyl iodide **91**) as a colourless oil.

$\boldsymbol{R_f}$ = 0.48 [petroleum ether (30–40)]; $[\alpha]_D^{26.6}$ = –162.4 (*c* = 1.33, $CHCl_3$); **IR** (film) ν_{max}/cm^{-1} 2956, 2929, 1572, 1465, 1432, 1306, 1248, 1209, 1096, 1060, 1044, 999, 975; **^{1}H NMR** ($CDCl_3$, 500 MHz) δ = 6.35 (1H, d, *J* = 19.2 Hz, C16H), 5.86 (1H, dd, *J* = 19.2, 1.8 Hz, C17H), 3.17–3.14 (1H, m, C21H), 1.80–1.74 (1H, m, C20H), 1.28–1.24 (2H, m, $C22H_AH_B$), 0.06 (9H, s, $Si(CH_3)_3$); **^{13}C NMR** ($CDCl_3$, 126 MHz) δ = 145.4 (C16H), 123.0 (C17H), 89.3 (C19), 78.9 (C18), 34.2 (C21H), 19.2 ($C22H_2$), 11.9 (C20H), –1.7 (3C, $Si(CH_3)_3$); **HRMS** (+EI) Found $[M]^+$ = 198.0634; $C_{10}H_{15}ClSi$ requires 198.0626, Δ 4.04 ppm.

(1*R*,2*S*)-1-Chloro-2-((3*Z*)-4-iodobut-3-en-1-yn-1-yl)cyclopropane S18

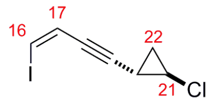

To a solution of **88** (9.70 mg, 48.8 μmol) in MeCN (1 mL) at RT was added NIS (16.5 mg, 73.2 μmol) in one portion and the reaction stirred in the absence of light for 16 h. The mixture was quenched by the addition of sat. aq. $Na_2S_2O_3$ (1 mL) and diluted with pentane (2 mL). The layers were separated and the aqueous layer extracted with pentane (3 × 2 mL), dried ($MgSO_4$) and carefully concentrated *in vacuo* (400 mbar, RT) to give the title compound **S18** as well as *E*-isomer **89** in a 3:1 mixture. Purification by column chromatography (SiO_2, pentane) provided a sample of the title compound **S18** (2.5 mg, 20%) for full characterisation as a colourless oil.

***R*$_f$** = 0.53 (pentane); $[\alpha]_D^{27.6}$ = –90.0 (*c* = 0.07, $CHCl_3$) **IR** (film) ν_{max}/cm^{-1} 2956, 2924, 2213, 1574, 1457, 1429, 1297, 1257, 1058, 1041, 989; **^{1}H NMR** ($CDCl_3$, 500 MHz) δ = 6.69 (1H, d, *J* = 8.2 Hz, C16H), 6.53 (1H, dd, *J* = 8.3, 2.0 Hz, C17H), 3.28–3.24 (1H, m, C21H), 1.85–1.80 (1H, m, C20H), 1.41–1.32 (2H, m, $C22H_AH_B$); **^{13}C NMR** ($CDCl_3$, 126 MHz) δ = 122.4 (C17H), 96.9 (C19), 91.9 (C16H), 78.1 (C18), 34.3 (C21H), 19.5 ($C22H_2$), 12.2 (C20H); **HRMS** (+EI) Found $[M]^+$ = 251.9201; C_7H_6ClI requires 251.9197, Δ 1.59 ppm.

(1*R*,2*S*)-1-Chloro-2-((3*E*)-4-iodobut-3-en-1-yn-1-yl)cyclopropane 89

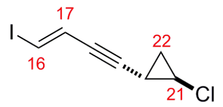

To a solution of IPy_2BF_4 (48.7 mg, 0.131 mmol) in CH_2Cl_2 (0.4 mL) at RT was added HBF_4•OEt_2 (36 µL, 0.262 mmol) dropwise resulting in a dark red solution. The mixture was cooled to 0 °C after which **88** (13 mg, 65.4 µmol) in CH_2Cl_2 (0.4 mL) was added dropwise. The reaction was maintained at 0 °C for 30 min† and then diluted with CH_2Cl_2 (2 mL) followed by the addition of sat. aq. $NaHCO_3$ (1 mL) and L-ascorbic acid (20% w/w, 2 mL). The resulting yellow solution was allowed to warm to RT over 30 min. The layers were separated and the aqueous layer further extracted with CH_2Cl_2 (3 × 5 mL), dried ($MgSO_4$) and concentrated *in vacuo*. Purification by column chromatography (Florisil, pentane) gave the title compound **89** (15.9 mg, 96%) as a colourless oil.

***R*$_f$** = 0.48 [petroleum ether (30–40)]; $[\alpha]_D^{27.8}$ = –152.7 (*c* = 1.18, $CHCl_3$); **IR** (film) ν_{max}/cm^{-1} 3058, 2924, 2218, 1686, 1564, 1430, 1358, 1256, 1177, 1096, 1060, 1045, 997, 923; **^{1}H NMR** ($CDCl_3$, 400 MHz) δ = 6.73 (1H, d, *J* = 15.0 Hz, C16H), 6.44 (1H, dd, *J* = 15.0, 1.9 Hz, C17H), 3.20–3.15 (1H, m, C21H), 1.75–1.69 (1H, m, C20H), 1.32–1.27 (2H, m, $C22H_AH_B$); **^{13}C NMR** ($CDCl_3$, 100 MHz) δ = 124.7 (C17H), 90.8 (C19), 89.7 (C16H), 77.7 (C18), 34.0 (C21H), 19.2 ($C22H_2$), 11.9 (C20H); **HRMS** (+EI) Found $[M]^+$ = 251.9197; C_7H_6ClI requires 251.9197, Δ 0.00 ppm.

† The starting material **88** and product **89** were co-polar. On several occasions the reaction was not complete after 30 min, but re-submission of the crude material to the above conditions gave comparable yields.

Tributyl((1*E*)-4-((1*S*,2*R*)-2-chlorocyclopropyl)but-1-en-3-yn-1-yl)stannane 93

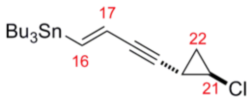

To a 50 mL round-bottomed flask was added **48** (2.56 g, 9.85 mmol), DMF (9.9 mL) and TBAF (1 M in THF, 24.6 mL, 24.6 mmol). The reaction vessel was sealed, and the mixture heated at 65 °C for 90 min, after which TLC analysis showed formation of a single product. The reaction mixture was cooled to RT after which brine (12 mL) was added and the mixture extracted with pentane (5 × 40 mL). The combined organic layers were dried ($MgSO_4$) and concentrated *in vacuo* (400 mbar, RT) to give the crude alkynyl bromide **86** (assumed quant.) which was used directly in the next step [$\boldsymbol{R_f}$ = 0.83 (hexane)].

To a solution of crude alkynyl bromide **86** (assumed quant., 9.85 mmol) in THF (25 mL) at RT was added Pd_2dba_3 (901 mg, 0.985 mmol) and $AsPh_3$ (1.21 g, 3.94 mmol) sequentially and stirred for 10 min. This was followed by the addition of Ag_2CO_3 (2.72 g, 9.85 mmol) in one portion and stirred for a further 10 min. The reaction mixture was then cooled to –10 °C and a solution of (*E*)-ethene-1,2-diylbis(tributylstannane) **92**[20] (10.5 mL, 19.7 mmol) in THF (25 mL) added in one portion *via* syringe. The reaction was maintained at –10 °C for 40 h, after which it was warmed to RT and the solvent removed *in vacuo*. Purification by column chromatography (SiO_2, pentane) gave the title compound **93** (1.83 g, 45% over 2 steps) as a colourless oil.

$\boldsymbol{R_f}$ = 0.73 (hexane); $[\alpha]_D^{25.0}$ = –73.7 (*c* = 1.14, $CHCl_3$); **IR** (film) ν_{max}/cm^{-1} 2956, 2923, 2872, 2853, 1556, 1464, 1417, 1377, 1356, 1340, 1292, 1255, 1184, 1152, 1072, 1043; **^{1}H NMR** ($CDCl_3$, 400 MHz) δ = 6.78 (1H, d, *J* = 19.7 Hz, C16H), 5.89 (1H, dd, *J* = 19.7, 1.7 Hz, C17H), 3.20–3.15 (1H, m, C21H), 1.82–1.75 (1H, m, C20H), 1.54–1.44 (6H, m, CH_2 of $SnBu_3$), 1.33–1.25 (8H, m, $C22H_AH_B$ and CH_2 of $SnBu_3$), 0.93–0.86 (15H, m, CH_2 of $SnBu_3$ and CH_3 of $SnBu_3$); **^{13}C NMR** ($CDCl_3$, 100 MHz) δ = 146.7 (C16H), 125.4 (C17H), 87.3 (C19), 79.1 (C18), 34.3 (C21H), 28.9 (3C, Bu), 27.5 (3C, Bu), 19.2 ($C22H_2$), 13.7 (3C, Bu), 11.4 (C20H), 9.6 (3C, Bu); An accurate mass could not be obtained for this compound, but a mass corresponding to the loss of butane was obtained **HRMS** (+EI) Found $[M–C_4H_9]^+$ = 359.0589; $C_{15}H_{24}ClSn$ requires

359.0583, Δ 1.67 ppm; **Elemental Analysis** found C, 55.37; H, 8.01; Cl, 8.28. $C_{19}H_{33}ClSn$ requires C, 54.91; H, 8.00; Cl, 8.53%.

***tert*-Butyl(((1*E*,4*R*,5*E*,7*E*)-10-((1*S*,2*R*)-2-chlorocyclopropyl)-2-methyl-1-trimethylsilyl)deca-1,5,7-trien-9-yn-4-yl)oxy)dimethylsilane 67**

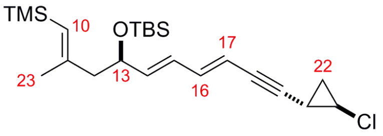

To a solution of vinyl iodide **84** (555 mg, 1.31 mmol) and stannane **93** (707 mg, 1.70 mmol) in DMF (28 mL) at RT was added freshly prepared $Pd(PFur_3)_2Cl_2$[21] (126 mg, 0.197 mmol) in one portion resulting in an orange solution. The reaction was stirred in the absence of light for 22 h, after which brine (28 mL) was added. The mixture was extracted with pentane (5 × 40 mL) and the combined organic layers dried ($MgSO_4$) and concentrated *in vacuo*. Purification by column chromatography (SiO_2, hexane) gave the title compound **67** (347 mg, 63%) as a pale yellow oil.

***R*$_f$** = 0.16 (pentane); $[\alpha]_D^{27.0}$ = –97.4 (*c* = 0.59, $CHCl_3$); **IR** (film) ν_{max}/cm^{-1} 2955, 2929, 2857, 1617, 1472, 1465, 1431, 1362, 1249, 1108, 1098, 1069, 983, 939, 926, 836; **^{1}H NMR** ($CDCl_3$, 400 MHz) δ = 6.49 (1H, dd, *J* = 15.5, 10.9 Hz, C16H), 6.14 (1H, dd, *J* = 15.2, 10.9 Hz, C15H), 5.73 (1H, dd, *J* = 15.2, 6.1 Hz, C14H), 5.49 (1H, d, *J* = 15.5 Hz, C17H), 5.22 (1H, s, C10H), 4.31–4.25 (1H, m, C13H), 3.19–3.14 (1H, m, C21H), 2.27 (1H, dd, *J* = 13.0, 7.5 Hz, C12H_AH_B), 2.17 (1H, dd, *J* = 13.0, 5.4 Hz, C12H_AH_B), 1.83–1.78 (1H, m, C20H), 1.78 (3H, s, C23H_3), 1.30–1.25 (2H, m, C22H_AH_B), 0.88 (9H, s, C(CH_3)$_3$ of *t*Bu), 0.08 (9H, s, C10HSi(CH_3)$_3$), 0.02 (3H, s, Si(CH_3)$_2$), 0.00 (3H, s, Si(CH_3)$_2$); **^{13}C NMR** ($CDCl_3$, 100 MHz) δ = 151.1 (C11), 141.3 (C16H), 139.5 (C14H), 128.1 (C15H), 127.3 (C10H), 110.2 (C17H), 91.1 (C19), 77.9 (C18), 72.1 (C13H), 51.6 (C12H_2), 34.3 (C21H), 25.9 (3C, C(*C*H_3)$_3$ of *t*Bu), 22.5 (C23H_3), 19.3 (*C*(CH_3)$_3$ of *t*Bu), 18.3 (C22H_2), 12.1 (C20H), 0.0 (3C, C10HSi(*C*H_3)$_3$), –4.6 (Si(*C*H_3)$_2$), –4.8 (Si(*C*H_3)$_2$); An accurate mass could not be obtained for this compound.

(1*S*,2*R*,1′*R*,2′*R*)-1,1′-(3*E*,5*E*)-Octa-3,5-diene-1,7-diyne-1,8-diylbis(2-chlorocyclopropane) S19

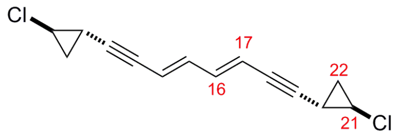

A small amount of homocoupled material **S19** (26.5 mg) was isolated from the previous procedure (for compound **67**) for full characterisation as an off-white solid.

R R_f = 0.18 (hexane); **m.p.** = 54–56 °C; $[\alpha]_D^{25.8}$ = –399.8 (*c* = 1.12, $CHCl_3$); **IR** (film) ν_{max}/cm^{-1} 2925, 2854, 2209, 1465, 1430, 1358, 1254, 1097, 1067, 1047, 975; **^{1}H NMR** ($CDCl_3$, 400 MHz) δ = 6.53–6.45 (2 × 1H, m, C17H), 5.63–5.53 (2 × 1H, m, C16H), 3.20–3.15 (2 × 1H, C21H), 1.84–1.78 (2 × 1H, m, C20H), 1.32–1.25 (2 × 2H, m, C22H_AH_B); **^{13}C NMR** ($CDCl_3$, 100 MHz) δ = 140.4 (2C, C16H), 113.0 (2C, C17H), 93.4 (2C, C19), 77.8 (2C, C18), 34.3 (2C, C21H), 19.4 (2C, C22H_2), 12.2 (2C, C20H); **HRMS** (+EI) Found $[M]^+$ = 250.0305; $C_{14}H_{12}Cl_2$ requires 250.0311, Δ 2.40 ppm.

Dimethyl (7*S*)-5-*O*-(*tert*-butyl(dimethyl)silyl)-7-((2*R*,3*R*,4*E*,7*R*,8*E*,10*E*)-7-((*tert*-butyl(dimethyl)silyl)oxy)-13-((1*S*,2*R*)-2-chlorocyclopropyl)-3-hydroxy-5-methyltrideca-4,8,10-trien-12-yn-2-yl)-2,4,6-trideoxy-6-methyl-α-L-*threo*-hept-3-ulopyranosidonate S20

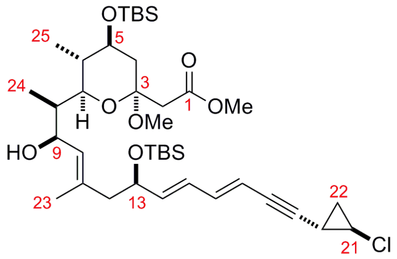

To a solution of vinyl iodide **55**† (202.5 mg, 0.425 mmol) in Et_2O (2 mL) at –78 °C was added added *t*BuLi (1.61 M, 0.57 mL, 0.916 mmol) dropwise resulting in the formation of a dark red solution. The mixture was stirred for 10 min after which a freshly prepared ethereal $ZnBr_2$ solution‡ (0.9 M, 0.49 mL, 0.441 mmol) was added dropwise. The resulting bright yellow solution was immediately warmed to 0 °C and maintained at this temperature for 1 h. In the meantime, a stock solution of (1*S*,2*R*)-(+)-*N*-methylephedrine (129 mg, 0.720 mmol) in PhMe (3.6 mL) was made, and dried by stirring with CaH_2 for 45 min. The stock solution was then cooled to 0 °C and *n*BuLi (2.25 M, 0.32 mL, 0.720 mmol) added dropwise and stirred for a further 15 min. Following this 1.96 mL (0.360 mmol) of this stock solution was then added to the organozinc solution and the reaction maintained at 0 °C for a further 1 h. After this time, aldehyde **5**† (127.2 mg, 0.327 mmol) was added as a solution in PhMe (0.70 mL) with further rinsing with PhMe (2 × 1 mL). The reaction was stirred for a further 1 h at 0 °C after which it was quenched by the addition of sat. aq. NH_4Cl (1.5 mL) and H_2O (0.5 mL) and warmed to RT. The layers were separated and the aqueous layer further extracted with Et_2O (3 × 10 mL), dried ($MgSO_4$) and concentrated *in vacuo*. Purification by column chromatography (SiO_2, hexane→hexane:Et_2O, 4:1→2:1→1:1) gave the title compound **S20** (116 mg, 48%) as a pale yellow oil.

---

† Azeotroped with PhMe (× 3) prior to use.

‡ Prepared by heating $ZnBr_2$ at 250 °C under vacuum for 3 days, then dissolved in freshly distilled Et_2O.

***R*$_f$** = 0.38 (hexane:Et_2O, 1:1); $[\alpha]_D^{26.2}$ = –70.3 (*c* = 1.00, $CHCl_3$); **IR** (film) ν_{max}/cm^{-1} 2954, 2930, 2857, 1742, 1463, 1438, 1378, 1361, 1317, 1253, 1223, 1146, 1074, 1039, 985; **^{1}H NMR** ($CDCl_3$, 400 MHz) δ = 6.47 (1H, dd, *J* = 15.5, 10.8 Hz, C16H), 6.13 (1H, dd, *J* = 15.1, 11.1 Hz, C15H), 5.72 (1H, dd, *J* = 15.1, 6.2 Hz, C14H), 5.48 (1H, d, *J* = 15.6 Hz, C17H), 5.24 (1H, d, *J* = 8.6 Hz, C10H), 4.36–4.27 (2H, m, C9H and C13H), 3.77 (1H, d, *J* = 10.6 Hz, C7H), 3.67 (3H, s, C1O_2CH_3), 3.70–3.63 (1H, m, C5H), 3.28 (3H, s, C3OCH_3), 3.18–3.14 (1H, m, C21H), 2.69 (1H, d, *J* = 13.4 Hz, C2H_AH_B), 2.61 (1H, d, *J* = 13.5 Hz, C2H_AH_B), 2.29 (1H, dd, *J* = 13.4, 6.1 Hz, C12H_AH_B), 2.17 (1H, dd, *J* = 13.2, 6.6 Hz, C12H_AH_B), 2.12 (1H, dd, *J* = 13.2, 4.8 Hz, equatorial C4H_AH_B), 1.91 (1H, d, *J* = 5.2 Hz, C9H(O*H*)), 1.82–1.76 (1H, m, C20H), 1.74–1.65 (2H, m, axial C4H_AH_B and C8H), 1.70 (3H, s, C23H_3), 1.50–1.40 (1H, m, C6H), 1.30–1.25 (2H, m, C22H_AH_B), 0.90 (9H, s, C(CH_3)$_3$ of *t*Bu), 0.88 (9H, s, C(CH_3)$_3$ of *t*Bu), 0.84 (3H, d, *J* = 6.5 Hz, C25H_3), 0.81 (3H, d, *J* = 7.0 Hz, C24H_3), 0.07 (6H, s, Si(CH_3)$_2$), 0.04 (3H, s, Si(CH_3)$_2$), 0.01 (3H, s, Si(CH_3)$_2$); **^{13}C NMR** ($CDCl_3$, 100 MHz) δ = 169.8 (*C*1O_2CH_3), 141.1 (C16H), 139.0 (C14H), 135.1 (C11), 131.0 (C10H), 128.5 (C15H), 110.6 (C17H), 99.2 (C3), 91.3 (C19), 77.8 (C18), 72.9 (C7H), 72.1 (one of C9H or C13H), 70.4 (one of C9H or C13H), 70.2 (C5H), 51.7 (C1O_2CH_3), 48.5 (C12H_2), 48.4 (C3O*C*H_3), 43.1 (C4H_2), 42.0 (C2H_2), 40.0 (C6H), 39.3 (C8H), 34.3 (C21H), 25.9 (6C, C(*C*H_3)$_3$ of *t*Bu), 19.3 (C22H_2), 18.2 (*C*(CH_3)$_3$ of *t*Bu), 18.1 (*C*(CH_3)$_3$ of *t*Bu), 17.8 (C23H_3), 12.5 (C20H), 12.1 (C25H_3), 9.7 (C24H_3), –4.0 (Si(*C*H_3)$_2$), –4.4 (Si(*C*H_3)$_2$), –4.6 (Si(*C*H_3)$_2$), –4.7 (Si(*C*H_3)$_2$); **HRMS** (+ESI) Found $[M+Na]^+$ = 761.3987; $C_{39}H_{67}O_7ClSi_2Na$ requires 761.4006, Δ 2.50 ppm.†

† Note: Pyran **5** is completely consumed in this reaction.

***tert*-Butyl(((4*R*,5*E*,7*E*)-10-((1*S*,2*R*)-2-chlorocyclopropyl)-2-methyldeca-1,5,7,-trien-9-yn-4-yl)oxy)dimethylsilane S21**

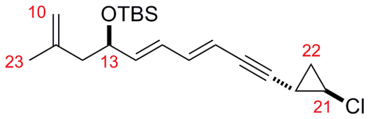

Deiodinated by-product **S21** (92.8 mg) was isolated from the previous procedure (for compound **S20**) for full characterisation as a colourless oil.

R R_f = 0.86 (hexane:Et_2O, 1:1); $[\alpha]_D^{25.7}$ = –110.2 (*c* = 1.02, $CHCl_3$); **IR** (film) ν_{max}/cm^{-1} 2954, 2930, 2857, 1648, 1472, 1463, 1431, 1391, 1362, 1255, 1107, 1096, 1068, 983; **^{1}H NMR** ($CDCl_3$, 400 MHz) δ = 6.50 (1H, dd, *J* = 15.5, 10.8 Hz, C16H), 6.16 (1H, dd, *J* = 15.1, 10.9 Hz, C15H), 5.75 (1H, dd, *J* = 15.2, 6.1 Hz, C14H), 5.49 (1H, d, *J* = 15.6, C17H), 4.78 (1H, s, C10$H_A$$H_B$), 4.69 (1H, s, C10$H_A$$H_B$), 4.31–4.25 (1H, m, C13H), 3.19–3.14 (1H, m, C21H), 2.26 (1H, dd, *J* = 13.3, 6.4 Hz, C12$H_A$$H_B$), 2.15 (1H, dd, *J* = 13.4, 6.2 Hz, C12$H_A$$H_B$), 1.83–1.77 (1H, m, C20H), 1.73 (3H, s, C23H_3), 1.30–1.25 (2H, m, C22$H_A$$H_B$), 0.88 (9H, s, C(C$H_3$)$_3$ of *t*Bu), 0.04 (3H, s, Si(CH_3)$_2$), 0.01 (3H, s, Si(CH_3)$_2$); **^{13}C NMR** ($CDCl_3$, 100 MHz) δ = 141.9 (C11), 141.3 (C16H), 139.3 (C14H), 128.3 (C10H_2), 113.4 (C15H), 110.3 (C17H), 91.2 (C19), 77.9 (C18), 71.7 (C13H), 46.9 (C12H_2), 34.3 (C21H), 25.9 (3C, C(*C*H_3)$_3$ of *t*Bu), 23.1 (C23H_3), 19.3 (C22H_2), 18.3 (*C*(CH_3)$_3$ of *t*Bu), 12.1 (C20H), –4.4 (Si(*C*H_3)$_2$), –4.8 (Si(*C*H_3)$_2$); An accurate mass could not be obtained for this compound.

Dimethyl (7*S*)-5-*O*-(*tert*-butyl(dimethyl)silyl)-7-((2*R*,3*R*,4*E*,7*R*,8*E*,10*E*)-7-((*tert*-butyl(dimethyl)silyl)oxy)-13-((1*S*,2*R*)-2-chlorocyclopropyl)-3-methoxy-5-methyltrideca-4,8,10-trien-12-yn-2-yl)-2,4,6-trideoxy-6-methyl-α-L-*threo*-hept-3-ulopyranosidonate 95[7]

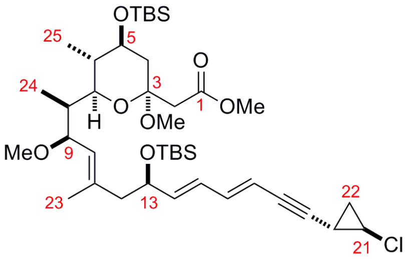

To a solution of **S20** (102.0 mg, 0.138 mmol) in CH_2Cl_2 (6.9 mL) at RT was added 2,6-di-*tert*-butylpyridine (0.62 mL, 2.76 mmol) and MeOTf (0.15 mL, 1.38 mmol) sequentially. The reaction was stirred at RT for 21 h after which it was quenched by the addition of MeOH (2.5 mL) and poured onto sat. aq. $NaHCO_3$ (30 mL). The reaction mixture was extracted with CH_2Cl_2 (3 × 30 mL) and the combined organic layers dried ($MgSO_4$) and concentrated *in vacuo*. Purification by column chromatography (SiO_2, hexane→hexane:Et_2O, 20:1→10:1) gave the title compound **95** (75.7 mg, 73%) as a colourless oil.

$\boldsymbol{R_f}$ = 0.58 (hexane:Et_2O, 7:3); $[\alpha]_D^{26.0}$ = –84.8 (*c* = 1.07, $CHCl_3$), [lit.[7] $[\alpha]_D^{23}$ = –76.58 (*c* = 1.45, $CHCl_3$)]; **IR** (film) ν_{max}/cm^{-1} 2952, 2929, 2857, 1743, 1471, 1463, 1437, 1376, 1361, 1315, 1255, 1223, 1188, 1146, 1079, 1041, 984; **^{1}H NMR** ($CDCl_3$, 400 MHz) δ = 6.46 (1H, dd, *J* = 15.5, 10.9 Hz, C16H), 6.12 (1H, dd, *J* = 15.2, 10.9 Hz, C15H), 5.72 (1H, dd, *J* = 15.1, 6.5 Hz, C14H), 5.46 (1H, d, *J* = 15.5 Hz, C17H), 4.93 (1H, d, *J* = 9.6 Hz, C10H), 4.36–4.30 (1H, m, C13H), 3.86 (1H, t, *J* = 9.7 Hz, C9H), 3.72 (1H, m, C5H), 3.69–3.66 (1H, m, C7H), 3.66 (3H, s, $C1O_2CH_3$), 3.23 (3H, s, $C3OCH_3$), 3.20–3.14 (1H, m, C21H), 3.11 (3H, s, C9H(OCH_3)), 2.66 (1H, d, *J* = 13.5 Hz, C2H_AH$_B$), 2.62 (1H, d, *J* = 13.5 Hz, C2H$_A$$H_B$), 2.38 (1H, dd, *J* = 13.2, 5.3 Hz, C12H_AH$_B$), 2.23 (1H, dd, *J* = 13.3, 7.5 Hz, C12H$_A$$H_B$), 2.12 (1H, dd, *J* = 12.8, 4.6 Hz, equatorial C4H_AH$_B$), 1.83–1.76 (1H, m, C20H), 1.69 (3H, s, C23H$_3$), 1.72–1.65 (2H, m, axial C4H$_A$$H_B$ and C8H), 1.44–1.36 (1H, m, C6H), 1.30–1.25 (2H, m, C22H$_A$H$_B$), 0.89 (9H, s, C(CH_3)$_3$ of *t*Bu), 0.88 (9H, s, C(CH_3)$_3$ of *t*Bu), 0.84 (3H, d, *J* = 6.5 Hz, C25H$_3$), 0.68 (3H, d, *J* = 7.0 Hz, C24H$_3$), 0.07 (6H, s, Si(CH_3)$_2$), 0.05 (3H, s, Si(CH_3)$_2$), 0.03 (3H, s, Si(CH_3)$_2$); **^{13}C NMR** ($CDCl_3$, 100 MHz) δ = 170.0 (*C*1O$_2$CH$_3$), 141.1 (C16H), 138.9 (C14H), 136.9 (C11), 129.0 (C10H), 128.7

(C15H), 110.5 (C17H), 98.9 (C3), 91.3 (C19), 77.8 (C9H), 77.3 (C18), 72.3 (C13H), 71.8 (C5H), 70.7 (C7H), 55.3 (C9H(O*C*H$_3$)), 51.6 (C1O$_2$*C*H$_3$), 48.8 (C12H$_2$), 47.7 (C3O*C*H$_3$), 43.4 (C4H$_2$), 42.1 (C2H$_2$), 39.9 (C6H), 38.6 (C8H), 34.3 (C21H), 25.9 (6C, C(*C*H$_3$)$_3$ of *t*Bu), 19.3 (C22H$_2$), 18.1 (2C, *C*(CH$_3$)$_3$ of *t*Bu), 18.0 (C23H$_3$), 12.4 (C20H), 12.1 (C25H$_3$), 8.7 (C24H$_3$), –4.0 (Si(*C*H$_3$)$_2$), –4.3 (Si(*C*H$_3$)$_2$), –4.6 (Si(*C*H$_3$)$_2$), –4.7 (Si(*C*H$_3$)$_2$); **HRMS** (+ESI) Found $[M+Na]^+$ = 775.4148; $C_{40}H_{69}O_7ClSi_2Na$ requires 775.4163, Δ 1.93 ppm; **Elemental Analysis** found C, 63.80; H, 9.24; Cl, 4.68. $C_{40}H_{69}O_7ClSi_2$ requires C, 63.75; H, 9.23; Cl, 4.70%.

Dimethyl (7*S*)-5-*O*-(*tert*-butyl(dimethyl)silyl)-7-((2*R*,3*R*,4*E*,7*R*,8*E*,10*E*)-13-((1*S*,2*R*)-2-chlorocyclopropyl)-7-hydroxy-3-methoxy-5-methyltrideca-4,8,10-trien-12-yn-2-yl)-2,4,6-trideoxy-6-methyl-α-L-*threo*-hept-3-ulopyranosidonate 99[7]

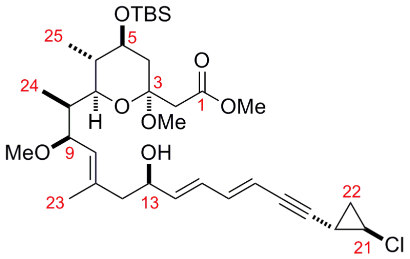

To a solution of **95** (75.7 mg, 0.10 mmol) in THF (3 mL) at RT was added TBAF (1 M in THF, 0.60 mL, 0.60 mmol) dropwise. The reaction mixture was stirred at RT for 30 min at which point TLC analysis indicated complete consumption of starting material. The reaction was quenched by the addition of sat. aq. NH_4Cl (1.2 mL) and EtOAc (10 mL) added. The layers were separated and the aqueous layer further extracted with EtOAc (3 × 10 mL). The combined organic layers were dried ($MgSO_4$) and concentrated *in vacuo*. Purification by column chromatography (SiO_2, hexane:Et_2O, 4:1→2:1→1:1) gave the title compound **99** (47.1 mg, 74%) as an off-white foam/gum.

R_f = 0.20 (hexane:Et_2O, 1:1); $[\alpha]_D^{26.0}$ = –71.6 (*c* = 0.86, $CHCl_3$), [lit.[7] $[\alpha]_D^{23}$ = –68.33 (*c* = 1.10, $CHCl_3$)]; **IR** (film) ν_{max}/cm^{-1} 3434, 2956, 2930, 2857, 1741, 1465, 1437, 1381, 1316, 1255, 1223, 1189, 1146, 1123, 1078, 1034, 984; **^{1}H NMR** ($CDCl_3$, 400 MHz) δ = 6.49 (1H, dd, *J* = 15.4, 10.9 Hz, C16H), 6.26 (1H, dd, *J* = 15.1, 10.9 Hz, C15H), 5.77 (1H, dd, *J* = 15.2, 6.2 Hz, C14H), 5.52 (1H, d, *J* = 15.5 Hz, C17H), 5.03 (1H, d, *J* = 9.5 Hz, C10H), 4.36–4.29 (1H, m, C13H), 3.90 (1H, t, *J* = 9.7 Hz, C9H), 3.73 (1H, m, C5H), 3.71–3.63 (1H, m, C7H), 3.66 (3H, s, $C1O_2CH_3$), 3.23 (3H, s, $C3OCH_3$), 3.20–3.15 (1H, m, C21H), 3.13 (3H, s, C9H(OCH_3)), 2.66 (1H, d, *J* = 13.5 Hz, C2$H_A$$H_B$), 2.62 (1H, d, *J* = 13.2 Hz, C2$H_A$$H_B$), 2.31 (2H, appar. d, *J* = 6.8 Hz, C12H_AH_B), 2.12 (1H, dd, *J* = 12.8, 4.6 Hz, equatorial C4$H_A$$H_B$), 1.82–1.76 (1H, m, C20H), 1.73 (3H, s, C23H_3), 1.71–1.65 (2H, m, axial C4$H_A$$H_B$ and C8H), 1.46–1.35 (1H, m, C6H), 1.30–1.26 (2H, m, C22H_AH_B), 0.89 (9H, s, C(CH_3)$_3$ of *t*Bu), 0.84 (3H, d, *J* = 6.5 Hz, C25H_3), 0.70 (3H, d, *J* = 7.0 Hz, C24H_3), 0.06 (6H, s, Si(CH_3)$_2$); **^{13}C NMR** ($CDCl_3$, 100 MHz) δ = 169.9 (*C*1O_2CH_3), 140.7 (C16H), 137.7 (C14H), 136.7 (C11), 129.7 (C10H), 129.5 (C15H), 111.4 (C17H), 98.9

(C3), 91.7 (C19), 77.7 (C9H), 77.3 (C18), 71.8 (C5H), 70.7 (C7H), 70.0 (C13H), 55.4 (C9H(O*C*H_3)), 51.6 (C1O_2*C*H_3), 48.0 (C12H_2), 47.8 (C3O*C*H_3), 43.4 (C4H_2), 42.1 (C2H_2), 39.9 (C6H), 38.5 (C8H), 34.3 (C21H), 25.9 (3C, C(*C*H_3) of *t*Bu), 19.3 (C22H_2), 18.1 (*C*(CH_3)$_3$ of *t*Bu), 17.2 (C23H_3), 12.4 (C20H), 12.1 (C25H_3), 8.9 (C24H_3), –4.0 (Si(*C*H_3)$_2$), –4.6 (Si(*C*H_3)$_2$); **HRMS** (+ESI) Found $[M+Na]^+$ = 661.3299; $C_{34}H_{55}O_7ClSiNa$ requires 661.3298, Δ 0.15 ppm. All spectroscopic data in agreement with that previously published.[7]

Methyl (7*S*)-5-*O*-(*tert*-butyl(dimethyl)silyl)-7-((2*R*,3*R*,4*E*,7*R*,8*E*,10*E*)-13-((1*S*,2*R*)-2-chlorocyclopropyl)-7-hydroxy-3-methoxy-5-methyltrideca-4,8,10-trien-12-yn-2-yl)-2,4,6-trideoxy-6-methyl-α-L-*threo*-hept-3-ulopyranosidonic acid 100[7]

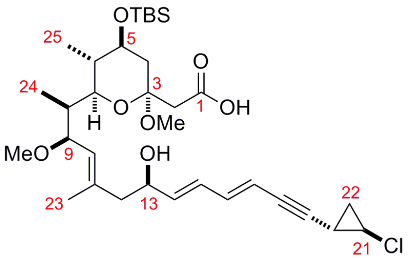

To a solution of **99** (47.1 mg, 73.7 μmol) in MeOH (2.5 mL) at RT was added $Ba(OH)_2•8H_2O$ (349 mg, 1.105 mmol) in one portion and stirred for 18 h. The reaction was poured onto EtOAc (20 mL) and H_2O (10 mL) (2:1) and stirred vigorously, with 0.05 M HCl added slowly dropwise until the pH of the aqueous layer reached 1 (approximately 100 mL of 0.05 M HCl). The layers were separated and the aqueous layer further extracted with EtOAc (50 mL). The combined organic layers were dried ($MgSO_4$) and concentrated *in vacuo* to give the title compound **100** (50.0 mg, quant.) as an off-white foam/gum.

$\boldsymbol{R_f}$ = 0.13 (hexane:Et_2O, 1:1); $[\alpha]_D^{25.9}$ = –66.3 (*c* = 1.25, $CHCl_3$), [lit.[7] $[\alpha]_D^{23}$ = –98.41 (*c* = 0.57, $CHCl_3$)]; **IR** (film) ν_{max}/cm^{-1} 2927, 2853, 1712, 1463, 1437, 1383, 1315, 1256, 1221, 1189, 1147, 1124, 1078, 1031, 984; **^{1}H NMR** (C_6D_6, 400 MHz) δ = 6.66 (1H, dd, *J* = 15.4, 10.9 Hz, C16H), 6.23 (1H, dd, *J* = 15.0, 11.1 Hz, C15H), 5.69–5.62 (2H, m, C14H and C17H), 5.13 (1H, d, *J* = 9.4 Hz, C10H), 4.22–4.16 (2H, m, C7H and C13H), 4.13 (1H, t, *J* = 9.7 Hz, C9H), 4.01 (1H, td, *J* = 10.2, 4.4 Hz, C5H), 3.35 (3H, s, C3OCH_3), 3.20 (3H, s, C9H(OC*H*$_3$)), 3.02–2.97 (1H, m, C21H), 2.72 (1H, d, *J* = 13.9 Hz, C2H_AH_B), 2.68 (1H, d, *J* = 13.8 Hz, C2H_AH_B), 2.56 (1H, dd, *J*

= 12.9, 12.7, 4.5, 4.3 Hz, equatorial C4$H_A$$H_B$), 2.30–2.16 (2H, m, 2 × C12H_AH_B), 2.07–1.92 (2H, m, axial C4$H_A$$H_B$ and C8H), 1.73 (3H, s, C23H_3), 1.75–1.68 (2H, m, C6H and C20H), 1.09 (9H, s, C(CH_3)$_3$ of *t*Bu), 1.04 (3H, d, J = 6.4 Hz, C25H_3), 0.95 (3H, d, J = 6.9 Hz, C24H_3), 0.93–0.90 (2H, m, C22H_AH_B), 0.23 (3H, s, Si(CH_3)$_2$), 0.17 (3H, s, Si(CH_3)$_2$); ^{13}**C NMR** (C_6D_6, 100 MHz) δ = 174.2 (C1O_2H), 141.1 (C16H), 138.5 (C14H), 136.9 (C11), 129.5 (C10H), 129.2 (C15H), 111.5 (C17H), 99.1 (C3), 91.9 (C19), 78.2 (C18), 77.5 (C9H), 72.4 (one of C7H or C13H), 70.9 (C5H), 70.0 (one of C7H or C13H), 55.0 (C9H(O*C*H_3)), 47.8 (C12H_2), 47.8 (C3O*C*H_3), 43.8 (C4H_2), 42.1 (C2H_2), 40.2 (C6H), 38.9 (C8H), 34.2 (C21H), 25.9 (3C, C(*C*H_3)$_3$ of *t*Bu), 18.9 (C22H_2), 18.1 (*C*(CH_3)$_3$ of *t*Bu), 17.1 (C23H_3), 12.5 (C20H), 12.2 (C25H_3), 8.9 (C24H_3), –4.1 (Si(*C*H_3)$_2$), –4.8 (Si(*C*H_3)$_2$); **HRMS** (+ESI) Found $[M+Na]^+$ = 647.3146; $C_{33}H_{53}O_7ClSiNa$ requires 647.3141, Δ 0.77 ppm.

(1*R*,6*R*,8*E*,10*R*,11*R*,12*R*,13*R*,14*S*)-6-((1*E*,3*E*)-6-((1*S*,2*R*)-2-Chlorocyclopropyl)hexa-1,3-dien-5-yn-1-yl)-1,14-dihydroxy-10-methoxy-8,11,13-trimethyl-5,16-dioxabicyclo[10.3.1]hexadec-8-en-4-one 4[22]

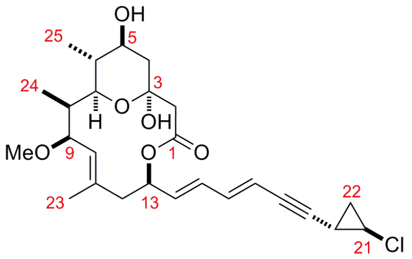

To a solution of **100** (62.9 mg, 0.10 mmol) in PhMe (7.4 mL) at RT was added Et_3N (83.6 μL, 0.60 mmol) and freshly distilled 2,4,6-trichlorobenzoyl chloride (78.1 μL, 0.50 mmol) sequentially dropwise. The reaction mixture was stirred for 30 min at RT after which it was further diluted with PhMe (29 mL). This solution was then added *via* syringe pump to a solution of DMAP (61.1 mg, 0.50 mmol) in PhMe (74 mL) at 80 °C over 8.5 h. Following addition, the reaction mixture was stirred for a further 30 min at 80 °C before being cooled to RT and quenched by the addition of MeOH (10 mL) and sat. aq. $NaHCO_3$ (34 mL). The layers were separated and the aqueous layer further extracted with EtOAc (3 × 60 mL) and the combined organic layers washed with brine (120 mL), dried ($MgSO_4$), and concentrated *in vacuo*.

Purification by column chromatography (SiO_2, hexane:EtOAc, 9:1) gave a mixture (assumed quant.) of TBS-protected aglycon and C3-eliminated material.

To the semi-pure mixture (assumed quant., 0.10 mmol) dissolved in THF (15 mL) at RT was added H_2O (3 mL) (5:1) followed by TFA (0.82 mL, 10.7 mmol) dropwise. The reaction was stirred at RT for 12 h after which it was quenched by the addition of sat. aq. $NaHCO_3$ (27 mL). The mixture was extracted with EtOAc (3 × 30 mL) and the combined organic extracts dried ($MgSO_4$), and concentrated *in vacuo*. Purification by column chromatography (SiO_2, hexane:EtOAc, 7:3→1:1) gave the title compound **4** (27.6 mg, 58% over 2 steps) as an off-white foam/gum.

$\boldsymbol{R_f}$ = 0.17 (hexane:EtOAc, 7:3); $[\alpha]_D^{25.6}$ = –44.6 (*c* = 0.75, $CHCl_3$), [lit.[22] $[\alpha]_D^{20}$ = –33.4 (*c* = 0.53, $CHCl_3$)]; **IR** (film) ν_{max}/cm^{-1} 3442, 2964, 2925, 2873, 1702, 1431, 1415, 1348, 1323, 1256, 1225, 1178, 1151, 1080, 1023, 999, 978; **^{1}H NMR** ($CDCl_3$, 400 MHz) δ = 6.48 (1H, dd, *J* = 15.5, 10.9 Hz, C16H), 6.27 (1H, dd, *J* = 14.9, 10.9 Hz, C15H), 5.86–5.80 (1H, m, C13H), 5.76 (1H, dd, *J* = 14.9, 6.4 Hz, C14H), 5.57 (1H, dd, *J* = 15.5, 1.5 Hz, C17H), 5.31 (1H, d, *J* = 9.4 Hz, C10H), 5.02 (1H, d, *J* = 2.2 Hz, C3OH), 3.80 (1H, dd, *J* = 9.7, 2.0 Hz, C9H), 3.79–3.75 (1H, m, C5H), 3.61 (1H, dd, *J* = 10.2, 2.4 Hz, C7H), 3.23 (3H, s, C9H(OCH_3)), 3.20–3.16 (1H, m, C21H), 2.55 (1H, d, *J* = 12.9 Hz, C2$H_A$$H_B$), 2.44 (1H, d, *J* = 12.9 Hz, C2$H_A$$H_B$), 2.31–2.28 (2H, m, 2 × C12$H_A$$H_B$), 2.25–2.18 (1H, m, C8H), 2.10 (1H, dd, *J* = 11.9, 4.6 Hz, equatorial C4$H_A$$H_B$), 1.84–1.76 (1H, m, C20H), 1.73 (3H, s, C23H_3), 1.59–1.52 (1H, br s, C5OH), 1.43–1.35 (1H, m, C6H), 1.31–1.25 (3H, m, C22$H_A$$H_B$ and axial C4$H_A$$H_B$), 0.98 (6H, d, *J* = 6.7 Hz, C24H_3 and C25H_3); **^{13}C NMR** ($CDCl_3$, 100 MHz) δ = 171.7 (C1O_2), 140.2 (C16H) 132.7 (C14H), 132.3 (C11), 131.0 (C15H), 127.9 (C10H), 112.6 (C17H), 95.4 (C3), 92.3 (C19), 79.8 (C9H), 77.3 (C18), 75.0 (C7H), 71.5 (C13H), 69.6 (C5H), 55.3 (C9H(OCH_3)), 47.0 (C12H_2), 44.8 (C2H_2), 43.8 (C4H_2), 40.1 (C6H), 36.9 (C8H), 34.3 (C21H), 19.4 (C22H_2), 16.1 (C23H_3), 12.1 (C20H), 12.1 (C25H_3), 6.6 (C24H_3); **HRMS** (+ESI) Found $[M+Na]^+$ = 501.2009; $C_{26}H_{35}O_6ClNa$ requires 501.2014, Δ 1.00 ppm. All spectroscopic data in agreement with that previously published.[22]

1,5-Anhydro-2,6-dideoxy-L-*arabino*-hex-1-enitol S22

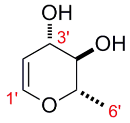

To a solution of 3,4-di-*O*-acetyl-6-deoxy-L-glucal† **107** (5.0 g, 23.3 mmol) in MeOH (110 mL) at RT was added PS-Na_2CO_3 (3.21 mmol/g, 7.56 g, 24.3 mmol) and the mixture shaken for 4 h. The reaction mixture was filtered and the beads washed with CH_2Cl_2 (2 × 30 mL), and MeOH (2 × 30 mL). The combined washings were concentrated *in vacuo* to give the title compound **S22** as a white solid (3.31 g, quant.) which was used without further purification.

$\boldsymbol{R_f}$ = 0.10 (hexane:Et_2O, 6:4); **m.p.** = 67–70 °C; $[\alpha]_D^{25.0}$ = +12.6 (*c* = 0.44, $CHCl_3$); **IR** (film) ν_{max}/cm^{-1} 3262, 3002, 2932, 2889, 1643, 1449, 1412, 1389, 1358, 1263, 1226, 1149, 1112, 1044, 1025; ^{1}**H NMR** ($CDCl_3$, 400 MHz) δ = 6.32 (1H, d, *J* = 6.1 Hz, C1′H), 4.71 (1H, dd, *J* = 6.0, 1.9 Hz, C2′H), 4.21 (1H, tt, *J* = 6.9, 1.7 Hz, C3′H), 3.90–3.83 (1H, m, C5′H), 3.43–3.39 (1H, m, C4′H), 2.29–2.27 (1H, br s, C3′HO*H*), 1.77–1.75 (1H, br s, C4′HO*H*), 1.39 (3H, d, *J* = 6.3 Hz, C6′H_3); ^{13}**C NMR** ($CDCl_3$, 100 MHz) δ = 144.9 (C1′H), 102.7 (C2′H), 75.7 (C4′H), 74.4 (C5′H), 70.4 (C3′H), 17.1 (C6′H_3); **HRMS** (+EI) Found $[M]^+$ = 130.0624; $C_6H_{10}O_3$ requires 130.0624, Δ 0.00 ppm; **Elemental Analysis** found C, 55.29; H, 7.86. $C_6H_{10}O_3$ requires C, 55.37; H, 7.74%.

1,5-Anhydro-2,6-dideoxy-L-*erythro*-hex-1-en-3-ulose 106

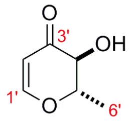

To a vigorously stirring solution of **S22** (3.31 g, 25.5 mmol) in CH_2Cl_2 (255 mL) at RT was added MnO_2 (13.3 g, 152.8 mmol). The mixture was stirred at RT until TLC analysis indicated that the starting material had been consumed. The MnO_2 was removed by filtration through a pad of silica under suction. The silica pad was washed with Et_2O (2 × 30 mL) and CH_2Cl_2 (2 × 30 mL) and the combined washings concentrated *in vacuo* to give the title compound **106** (2.2 g, 74% over 2 steps) as a white solid.

† er = 98.5:1.5

***R*f** = 0.19 (hexane:Et_2O, 6:4); **m.p.** = 93–94 °C; $[\alpha]_D^{25.0}$ = –154.0 (*c* = 1.04, $CHCl_3$); **IR** (film) ν_{max}/cm^{-1} 3399, 3106, 2986, 2915, 1661, 1592, 1466, 1406, 1379, 1325, 1253, 1203, 1126, 1051, 1030; **^{1}H NMR** ($CDCl_3$, 400 MHz) δ = 7.39 (1H, d, *J* = 5.8 Hz, C1′H), 5.46 (1H, d, *J* = 5.8 Hz, C2′H), 4.24–4.16 (1H, m, C5′H), 3.97 (1H, dd, *J* = 13.1, 1.7 Hz, C4′H), 3.51 (1H, d, *J* = 1.7 Hz, C4′HO*H*), 1.59 (3H, d, *J* = 6.2 Hz, C6′H_3); **^{13}C NMR** ($CDCl_3$, 100 MHz) δ = 194.1 (C3′), 164.7 (C1′H), 103.5 (C2′H), 80.0 (C5′H), 72.8 (C4′H), 18.0 (C6′H_3); An accurate mass could not be obtained for this compound, but a low resolution mass could be obtained; **HRMS** (+ESI): 129.17 $[M+H]^+$, 126.98 $[M–H]^+$.

1,5-Anhydro-2,6-dideoxy-4-*O*-((4-nitrophenyl)sulfonyl)-L-*erythro*-hex-1-en-3-ulose S23[23]

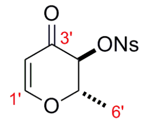

To a solution of **S22** (820 mg, 6.41 mmol) in CH_2Cl_2 (30 mL) at 0 °C was added pyridine (0.78 mL, 9.61 mmol) followed by nosyl chloride (1.70 g, 7.69 mmol) and the mixture slowly warmed to RT. After 48 h stirring at RT additional pyridine (2.85 mL, 35.26 mmol) and nosyl chloride (3.98 g, 17.95 mmol) were added and stirring continued for a further 24 h. The mixture was cooled to 0 °C, H_2O (10 mL) added and stirred at this temperature for 30 min, after which more H_2O (50 mL) was added. The layers were separated and the aqueous layer further extracted with CH_2Cl_2 (3 × 50 mL) and the combined organic layers washed with brine, dried ($MgSO_4$) and concentrated *in vacuo*. Purification by column chromatography (SiO_2, hexane:EtOAc, 85:15) gave the title compound **S23** (1.89 g, 95%) as a yellow solid.

***R*f** = 0.53 [petroleum ether (40–60):EtOAc, 1:1]; **m.p.** = 92–93 °C (lit[23] = 93 °C); $[\alpha]_D^{25.0}$ = –128.3 (*c* = 0.71, $CHCl_3$); **IR** (film) ν_{max}/cm^{-1} 3107, 2988, 1693, 1595, 1530, 1450, 1405, 1378, 1349, 1314, 1254, 1184, 1094, 1043, 1023; **^{1}H NMR** ($CDCl_3$, 500 MHz) δ = 8.38 (2H, d, *J* = 9.1 Hz, Ar, *ortho* to NO_2), 8.17 (2H, d, *J* = 9.1 Hz, Ar, *ortho* to SO_2), 7.33 (1H, d, *J* = 5.8 Hz, C1′H), 5.35 (1H, d, *J* = 5.8 Hz, C2′H), 4.99 (1H, d, *J* = 11.6 Hz, C4′H), 4.54–4.50 (1H, m, C5′H), 1.60 (3H, d, *J* = 6.4 Hz, C6′H_3); **^{13}C NMR** ($CDCl_3$, 126 MHz) δ = 186.1 (C3′), 163.6 (C1′H), 150.8 (Ar, *ipso* to NO_2), 142.0 (Ar, *ipso* to SO_2), 129.7 (2C, Ar, *ortho* to NO_2), 124.1 (2C, Ar, *ortho* to SO_2), 105.0 (C2′H), 79.6 (C4′H), 77.4 (C5′H), 17.4 (C6′H); **HRMS** (+ESI)

Found $[M+H]^+$ = 314.0349; $C_{12}H_{12}NO_7S$ requires 314.0334, Δ 4.78 ppm; **Elemental Analysis** found C, 46.14; H, 3.53; N, 4.31. $C_{12}H_{11}NO_7S$ requires C, 46.01; H, 3.54; N, 4.47%. All physical data in agreement with enantiomeric compound published by Nicolaou.[23]

1,5-Anhydro-4-azido2,4,6-trideoxy-L-*threo*-hex-1-en-3-ulose 108[23]

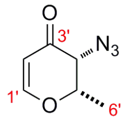

To a solution of **S23** (1.67 g, 5.33 mmol) in CH_2Cl_2 (33 mL) at 0 °C was added a solution of *n*Bu_4NN_3 (4.28 g, 15.0 mmol) in CH_2Cl_2 (30 mL, cooled to 0 °C) *via* cannula. The reaction mixture was stirred at 0 °C for 1 h and then warmed to RT slowly over the course of 2 h. The reaction was quenched by the addition of H_2O (50 mL) and the layers separated. The aqueous layer was further extracted using CH_2Cl_2 (2 × 100 mL) and the combined organic layers washed with brine (150 mL), dried ($MgSO_4$) and concentrated *in vacuo* to give an orange oil. Purification by column chromatography (SiO_2, hexane:Et_2O, 2:1) gave the title compound **108** (586 mg, 72%) as a yellow oil.

$\boldsymbol{R_f}$ = 0.21 (hexane:EtOAc, 4:1); $[\alpha]_D^{28.4}$ = +48.1 (*c* = 0.73, $CHCl_3$); **IR** (film) ν_{max}/cm^{-1} 2991, 2101, 1668, 1589, 1453, 1410, 1384, 1273, 1224, 1192, 1153, 1049; **^{1}H NMR** ($CDCl_3$, 500 MHz) δ = 7.37 (1H, d, *J* = 7.5 Hz, C1′H), 5.48 (1H, dd, *J* = 7.5, 1.0 Hz, C2′H), 4.55–4.51 (1H, m, C5′H), 3.80 (1H, dd, *J* = 4.0, 1.5 Hz, C4′H), 1.48 (3H, d, *J* = 8.0 Hz, C6′H_3); **^{13}C NMR** ($CDCl_3$, 126 MHz) δ = 187.3 (C3′), 163.6 (C1′H), 104.9 (C2′H), 77.3 (C5′H), 63.9 (C4′H), 15.3 (C6′H_3); **HRMS** (+ESI) Found $[M+H]^+$ = 176.0430; $C_6H_7N_3O_2Na$ requires 176.0429, Δ 0.57 ppm. All physical data in agreement with enantiomeric compound published by Nicolaou.[23]

nOe experiments performed to reveal the stereochemical relationship gave inconclusive results.

2,6-Anhydro-3-azido-1,3,5-trideoxy-4-*C*-methyl-L-*arabino*-hex-5-enitol 104[23]

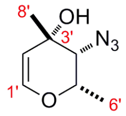

To a solution of **108** (2.6 g, 17.0 mmol) in THF (154 mL) cooled to –100 °C was added MeLi (1.6 M in Et_2O, 21.2 mL, 34.0 mmol) dropwise. After 2 h at –100 °C, the reaction was quenched with sat. aq. NH_4Cl (120 mL), diluted with EtOAc (120 mL) and allowed to warm to RT. The layers were separated and the aqueous layer further extracted with EtOAc (3 × 120 mL). The combined organic layers were washed with brine, dried ($MgSO_4$) and concentrated *in vacuo* to give a yellow oil. Purification by column chromatography (SiO_2, hexane:Et_2O, 8:2) gave the title compound **104** (2.27 g, 79%) as a yellow oil and single diastereomer.

$\boldsymbol{R_f}$ = 0.62 [petroleum ether (40–60):EtOAc, 1:1]; $[\alpha]_D^{25.0}$ = –51.1 (*c* = 1.31, $CHCl_3$); **IR** (film) ν_{max}/cm^{-1} 3423, 2986, 2937, 2913, 2107, 1645, 1449, 1385, 1342, 1312, 1278, 1233, 1157, 1136, 1076, 1054; **^{1}H NMR** ($CDCl_3$, 400 MHz) δ = 6.27 (1H, d, *J* = 6.3 Hz, C1′H), 4.69 (1H, dd, *J* = 6.3, 1.8 Hz, C2′H), 4.19 (1H, q, *J* = 6.5 Hz, C5′H), 3.34–3.32 (1H, br s, C4′H), 1.45 (3H, d, *J* = 6.5 Hz, C6′H_3), 1.43 (3H, s, C8′H_3); **^{13}C NMR** ($CDCl_3$, 100 MHz) δ = 143.6 (C1′H), 106.8 (C2′H), 72.2 (C5′H), 68.9 (C4′H), 68.4 (C3′), 29.6 (C8′H_3), 18.0 (C6′H_3); An accurate mass could not be obtained for this compound. All physical data in agreement with enantiomeric compound published by Nicolaou.[23]

Methyl 4-azido-4,6-dideoxy-3-*C*-methyl-α-L-talopyranoside 109[23]

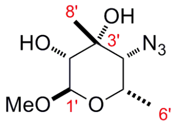

To a suspension of **104** (327 mg, 1.93 mmol) and $NaHCO_3$ (0.49 g, 5.79 mmol) in MeOH (7.4 mL) at 0 °C was added *m*-CPBA (70%, 0.57 g, 2.32 mmol). The mixture was stirred at 0 °C for 5 min and then warmed to RT. After 1 h the reaction was quenched with H_2O (25 mL), the layers separated and the aqueous layer further extracted with EtOAc (3 × 25 mL). The combined organic layers were partially concentrated *in vacuo* and the resulting bilayer re-dissolved in EtOAc (10 mL). The layers were again separated and the aqueous layer further extracted with EtOAc (3 × 25 mL). The combined organics were dried ($MgSO_4$) and concentrated *in vacuo*. Purification by column chromatography (Florisil, hexane:Et_2O, 7:3) gave the title compound **109** (218 mg, 52%) as a colourless oil.

$\boldsymbol{R_f}$ = 0.34 (hexane:EtOAc, 1:1); $[\alpha]_D^{28.4}$ = –104.2 (*c* = 0.48, $CHCl_3$); **IR** (film) ν_{max}/cm^{-1} 3448, 2983, 2938, 2105, 1449, 1385, 1341, 1259, 1128, 1066, 1050, 999, 976, 943, 909; **^{1}H NMR** ($CDCl_3$, 400 MHz) δ = 4.77 (1H, s, C1′H), 4.03 (1H, q, *J* = 6.4 Hz, C5′H), 3.58 (1H, s, C3′OH), 3.37 (3H, s, C1′HOCH_3), 3.31 (1H, d, *J* = 12.0 Hz, C2′H), 3.25 (1H, s, C4′H), 2.67 (1H, d, *J* = 12.0 Hz, C2′HO*H*), 1.39 (3H, s, C8′H_3), 1.35 (3H, d, *J* = 6.4 Hz, C6′H_3); **^{13}C NMR** ($CDCl_3$, 100 MHz) δ = 102.1 (C1′H), 73.3 (C2′H), 71.2 (C4′H), 70.0 (C3′), 64.3 (C5′H), 55.5 (C1′HOCH_3), 23.6 (C8′H_3), 18.1 (C6′H_3); **HRMS** (+ESI) Found $[M+Na]^+$ = 240.0955; $C_8H_{15}N_3O_4Na$ requires 240.0962, Δ 2.92 ppm. All physical data in agreement with enantiomeric compound published by Nicolaou.[23]

Key selected observed nOe's:

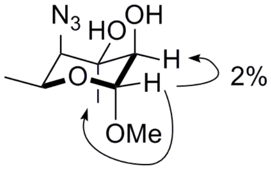

No nOe was observed between C1′H and C5′H, suggesting a *trans*-arrangement.

Methyl 4-azido-4,6-dideoxy-3-*C*-methyl-2-*O*-methyl-α-L-talopyranoside 110[23]

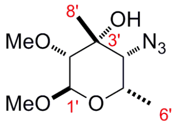

To a mixture of freshly sublimed KO*t*Bu (299 mg, 2.66 mmol) in THF (6.8 mL) at 0 °C was added a solution of **109** (551 mg, 2.54 mmol) in THF (8.3 mL) dropwise. The reaction mixture was stirred for 40 min at 0 °C after which MeI (0.19 mL, 3.05 mmol) was added dropwise to the bright yellow solution and stirred for 4 h. After this time, further KO*t*Bu (71.2 mg, 0.636 mmol) and MeI (31.6 μL, 0.508 mmol) were added and the reaction maintained at 0 °C until TLC analysis indicated full conversion of the starting material. The reaction was quenched with H_2O (18 mL), diluted with CH_2Cl_2 (30 mL) and warmed to RT. The layers were separated, the aqueous layer further extracted with CH_2Cl_2 (3 × 30 mL), and the combined organics dried ($MgSO_4$) and concentrated *in vacuo*. Purification by column chromatography (Florisil, hexane:Et_2O, 7:3→Et_2O) gave the title compound **110** (446 mg, 79%) as a colourless oil.[23]

$\boldsymbol{R_f}$ = 0.28 (hexane:Et_2O, 7:3); $[\alpha]_D^{27.0}$ = –49.2 (*c* = 1.03, $CHCl_3$); **IR** (film) ν_{max}/cm^{-1} 3516, 2984, 2944, 2913, 2833, 2104, 1463, 1445, 1383, 1348, 1280, 1263,1179, 1134, 1104, 1060, 1023; **^{1}H NMR** ($CDCl_3$, 500 MHz) δ = 4.77 (1H, s C1′H), 3.98 (1H, dq, *J* = 6.5, 1.4 Hz, C5′H), 3.68 (1H, s, C3′OH), 3.47 (3H, s, OCH_3), 3.34 (3H, s, C1′HOCH_3), 3.09 (1H, br s, C4′H), 2.88 (1H, s, C2′H), 1.38 (3H, s, C8′H_3), 1.34 (3H, d, *J* = 6.5 Hz, C6′H_3); **^{13}C NMR** ($CDCl_3$, 126 MHz) δ = 98.4 (C1′H), 81.9 (C2′H), 70.1 (C4′H), 69.6 (C3′), 64.2 (C5′H), 59.8 (OCH_3), 55.2 (C1′HOCH_3), 24.5 (C8′H_3), 17.9 (C6′H_3); **HRMS** (+ESI) Found $[M+Na]^+$ = 254.1122; $C_9H_{17}N_3O_4Na$ requires 254.1111, Δ 4.33 ppm. All physical data in agreement with enantiomeric compound published by Nicolaou.[23]

The corresponding D-configured enantiomer was synthesised and found to have the following optical rotation: $[\alpha]_D^{26.0}$ = +54.6 (*c* = 1.17, $CHCl_3$).

(3a*R*,4*S*,6*R*,7*R*,7a*R*)-6,7-Dimethoxy-4,7a-dimethyltetrahydro-4*H*-pyrano[3,4-*d*]oxazol-2(3*H*)-one 101[23]

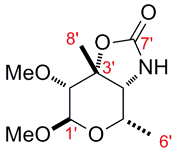

To a solution of **110** (156 mg, 0.675 mmol) in EtOAc (68 mL) at RT was added $Pd(OH)_2/C$ (26 mg). The flask was then evacuated and backfilled with hydrogen (× 3) and left to stir at RT for 4 h 30 min. The mixture was filtered through a pad of Celite® under suction using EtOAc (3 × 80 mL) and concentrated *in vacuo*. The isolated amine (**S24**) (120 mg, 87%) was used immediately in the next step of the reaction without further purification.

To a solution of amine (**S24**) (120 mg, 0.585 mmol) in CH_2Cl_2 (9.8 mL) at –78 °C was added pyridine (0.85 mL, 10.5 mmol) followed by a solution of triphosgene (189.5 mg, 0.702 mmol) in CH_2Cl_2 (5.1 mL) *via* syringe. The resulting bright orange solution was stirred at –78 °C for 20 min after which it was warmed to RT over 1 h. The solution was quenched with 1 *N* HCl (5 mL) and the layers separated. The aqueous layer was further extracted with CH_2Cl_2 (3 × 20 mL) and the combined organic layers washed with sat. aq. $NaHCO_3$ (20 mL), brine (20 mL), dried ($MgSO_4$) and concentrated *in vacuo*. Purification by column chromatography (SiO_2, EtOAc:hexane, 4:1) gave the title compound **101** (97 mg, 72%) as a white solid.

$\boldsymbol{R_f}$ = 0.26 [EtOAc:petroleum ether (40–60), 4:1]; **m.p.** = 145–147 °C (lit[23] = 147–148 °C); $[\alpha]_D^{27.0}$ = –87.3 (*c* = 1.01, $CHCl_3$); **IR** (film) ν_{max}/cm^{-1} 3298, 2913, 1744, 1719, 1425, 1374, 1320, 1273, 1202, 1105, 1061, 1027, 999; **^{1}H NMR** ($CDCl_3$, 400 MHz) δ = 5.17 (1H, br s, NH), 4.63 (1H, d, *J* = 5.5 Hz, C1′H), 3.91 (1H, qd, *J* = 6.4, 1.4 Hz, C5′H), 3.55 (3H, s, OCH_3), 3.43 (3H, s, C1′HOCH_3), 3.35 (1H, br s, C4′H), 3.19 (1H, d, *J* = 5.5 Hz, C2′H), 1.53 (3H, s, C8′H_3), 1.17 (3H, d, *J* = 6.5 Hz, C6′H_3); **^{13}C NMR** ($CDCl_3$, 100 MHz) δ = 158.0 (NH*C*7′O), 101.6 (C1′H), 81.8 (C3′), 81.1 (C2′H), 63.3 (C5′H), 61.1 (C4′H), 60.7 (OCH_3), 55.0 (C1′HO*C*H_3), 23.3 (C8′H_3), 15.7 (C6′H_3); **HRMS** (+ESI) Found $[M+Na]^+$ = 232.1189; $C_{10}H_{18}NO_5$ requires 232.1185, Δ 1.72 ppm; **Elemental Analysis** found C, 51.85; H, 7.30; N, 5.83. $C_{10}H_{18}NO_5$ requires C, 51.94; H, 7.41; N, 6.06%.

(3a*R*,4*S*,6*R*,7*R*,7a*R*)-6,7-Dimethoxy-4,7a-dimethyl-3-(tripropan-2-ylsilyl)tetrahydro-4*H*-pyrano[3,4-*d*][1,3]oxazol-2(3*H*)-one 111

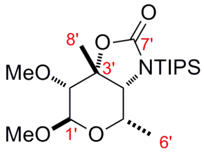

To a solution of **101** (130 mg, 0.563 mmol) in CH_2Cl_2 (13 mL) at RT was added 2,6-lutidine (0.26 mL, 2.25 mmol) followed by TIPSOTf (0.30 mL, 1.13 mmol) and the reaction stirred for 14 h. The reaction was diluted with CH_2Cl_2 (20 mL) and quenched with sat. aq. NH_4Cl (20 mL). The layers were separated and the aqueous layer further extracted with CH_2Cl_2 (3 × 50 mL). The combined organic layers were dried ($MgSO_4$) and concentrated *in vacuo*. Purification by column chromatography (Florisil, hexane→hexane:Et_2O, 1:1→EtOAc) gave the title compound **111** (211 mg, 97%) as a white solid.

R$_{\mathbf{f}}$ = 0.22 (Et_2O:hexane, 1:1); **m.p.** = 154–156 °C; $[\alpha]_D^{27.1}$ = –79.6 (*c* = 0.96, $CHCl_3$); **IR** (film) ν_{max}/cm^{-1} 2973, 2948, 2870, 1731, 1470, 1447, 1389, 1363, 1318, 1284, 1248, 1207, 1195, 1138, 1103, 1055, 1026; **^{1}H NMR** ($CDCl_3$, 400 MHz) δ = 4.60 (1H, d, *J* = 5.8 Hz, C1′H), (1H, qd, *J* = 6.3, 2.2 Hz, C5′H), 3.53 (3H, s, OCH_3), 3.43 (1H, br s, C4′H), 3.42 (3H, s, C1′HOCH_3), 3.23 (1H, d, *J* = 5.8 Hz, C2′H), 1.53 (3H, s, C8′H_3), 1.41 (3H, septet, *J* = 7.5 Hz, Si(C*H*(CH_3)$_2$)$_3$), 1.24–1.16 (21H, m, Si(CH(CH_3)$_2$)$_3$ and C6′H_3); **^{13}C NMR** ($CDCl_3$, 100 MHz) δ = 161.2 (*C*7′O_2), 102.1 (C1′H), 82.1 (C3′), 81.6 (C2′H), 65.6 (C5′H), 65.2 (C4′H), 60.3 (OCH_3), 54.9 (C1′HO*C*H_3), 22.5 (C8′H_3), 19.1 (3C, Si(CH(*C*H_3)$_2$)$_3$), 19.1 (3C, Si(CH(*C*H_3)$_2$)$_3$), 17.0 (C6′H_3), 13.2 (3C, Si(*C*H(CH_3)$_2$)$_3$); **HRMS** (+ESI) Found $[M+H]^+$ = 388.2519; $C_{19}H_{38}NO_5Si$ requires 388.2524, Δ 1.29 ppm and $[M+Na]^+$ = 410.2339; $C_{19}H_{37}NO_5SiNa$ requires 410.2347, Δ 1.95 ppm; **Elemental Analysis** found C, 58.94; H, 9.59; N, 3.55. $C_{19}H_{37}NO_5Si$ requires C, 58.88; H, 9.62; N, 3.61%.

(3a*R*,4*S*,6*S*,7*R*,7a*R*)-7-Methoxy-4,7a-dimethyl-6-(phenylthio)-3-tripropan-2-ylsilyl)tetrahydro-4*H*-pyrano[3,4-*d*][1,3]oxazol-2(3*H*)-one 112

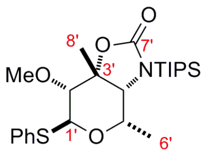

To a solution of **111** (121 mg, 0.313 mmol) in CH_2Cl_2 (3.2 mL) at 0 °C was added thiophenol (0.32 mL, 3.13 mmol) followed by BF_3•OEt_2 (0.58 mL, 4.70 mmol) and the mixture allowed to warm to RT slowly over 16 h. The reaction was quenched by the addition of sat. aq. $NaHCO_3$ (3 mL). The layers were separated and the aqueous layer further extracted with CH_2Cl_2 (3 × 10 mL). The combined organic layers were dried ($MgSO_4$) and concentrated *in vacuo*. Purification by column chromatography (Florisil, dry load, hexane→hexane:Et_2O, 9:1→7:3→EtOAc) gave the title compound **112** (116 mg, 80%) as a white solid.

***R*_f** = 0.28 (hexane:Et_2O, 7:3); **m.p.** = 127–130 °C; $[\alpha]_D^{28.3}$ = –75.0 (*c* = 0.82, $CHCl_3$); **IR** (film) ν_{max}/cm^{-1} 3690, 3677, 2969, 2905, 1722, 1469, 1455, 1443, 1395, 1385, 1365, 1318, 1249, 1211, 1126, 1104, 1083, 1057, 1025; **^{1}H NMR** ($CDCl_3$, 400 MHz) δ = 7.52 (2H, d, *J* = 6.9 Hz, *ortho* Ph), 7.32–7.24 (3H, m, *meta* and *para* Ph), 5.31 (1H, d, *J* = 7.9 Hz, C1′H), 4.06 (1H, qd, *J* = 6.5, 2.4 Hz, C5′H), 3.66 (3H, s, OCH_3), 3.49 (1H, d, *J* = 2.5 Hz, C4′H), 3.30 (1H, d, *J* = 8.2 Hz, C2′H), 1.59 (3H, s, C8′H_3), 1.44–1.34 (3H, m, Si(C*H*(CH_3)$_2$)$_3$), 1.22–1.16 (21H, m, Si(CH(C*H*$_3$)$_2$)$_3$ and C6′H_3); **^{13}C NMR** ($CDCl_3$, 100 MHz) δ = 161.1 (*C*7′O_2), 134.1 (*ipso* Ph), 131.7 (2C, *ortho* Ph), 128.9 (2C, *meta* Ph), 127.4 (*para* Ph), 86.7 (C1′H), 82.6 (C2′H), 81.5 (C3′), 66.4 (C5′H), 64.6 (C4′H), 61.8 (OCH_3), 22.8 (C8′H_3), 19.0 (3C, Si(CH(*C*H$_3$)$_2$)$_3$), 19.0 (3C, Si(CH(*C*H$_3$)$_2$)$_3$), 17.0 (C6′H_3), 13.1 (3C, Si(*C*H(CH_3)$_2$)$_3$); **HRMS** (+ESI) Found $[M+H]^+$ = 466.2462; $C_{24}H_{40}NO_4SiS$ requires 466.2447, Δ 3.22 ppm; **Elemental Analysis** found C, 62.01; H, 8.37; N, 2.87. $C_{24}H_{39}NO_4SiS$ requires C, 61.90; H, 8.44; N, 3.01%.

The structure and absolute stereochemistry was confirmed by X-ray crystallographic analysis after crystallisation from analytical grade Et_2O.

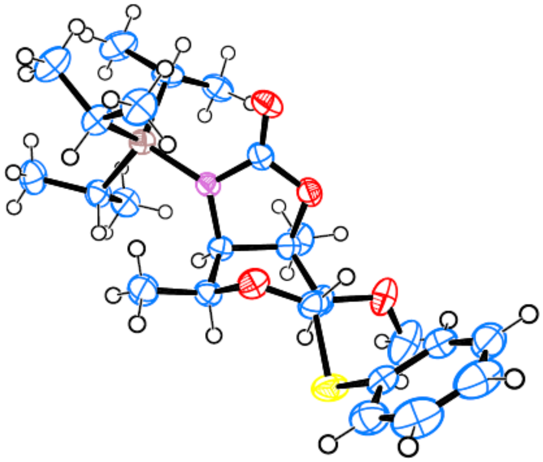

CCDC 882399 contains the supplementary crystallographic data for this thesis. This data can be obtained free of charge from the Cambridge Crystallographic Data Centre *via* www.ccdc.cam.ac.uk/data_request/cif.

Methyl 4,6-dideoxy-4-(formylamino)-3-*C*-methyl-2-*O*-methyl-α-L-talopyranoside 102

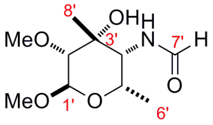

To a solution of **110** (212 mg, 0.918 mmol) in EtOAc (92 mL) at RT was added $Pd(OH)_2/C$ (35 mg). The flask was then evacuated and backfilled with hydrogen (× 3) and left to stir at RT for 4 h 30 min. The mixture was filtered through a pad of Celite® under suction using EtOAc (3 × 100 mL) and concentrated *in vacuo*. The isolated amine (**S24**) (assumed quant.) was used immediately in the next step of the reaction without further purification.

To a solution of the crude amine (**S24**) (assumed quant., 0.91 mmol) in $CHCl_3$ (9.1 mL) was added freshly prepared **113**[24] (0.23 mL, 1.82 mmol). The reaction was stirred at RT for 1 h, and then concentrated *in vacuo*. Purification by column chromatography (SiO_2, CH_2Cl_2:MeOH, 9:1) gave the title compound **102** (160 mg, 75% over 2 steps) as an off-white gum consisting of 2 rotamers (3.2:1 by ^{1}H NMR).

Rotameric ratio ascertained by ^{1}H NMR spectroscopy of the purified mixture; δ_H 8.16 (1H, d, *J* = 1.7 Hz, NHC7′*H*O major), 7.81 (1H, d, *J* = 11.9 Hz, NHC7′*H*O minor).

***R*f** = 0.28 (minor), 0.23 (major) (CH_2Cl_2:MeOH, 9:1); $[\alpha]_D^{27.6}$ = –72.7 (*c* = 0.94, $CHCl_3$); **IR** (film) ν_{max}/cm^{-1} 3397, 2984, 2940, 2911, 2832, 1681, 1643, 1513, 1464, 1449, 1385, 1356, 1344, 1311, 1185, 1131, 1105, 1058, 1024; **^{1}H NMR** (d^6-DMSO, 500 MHz) δ = 8.16 (1H, d, *J* = 1.7 Hz, NHC7′*H*O major rotamer), 7.81 (1H, d, *J* = 11.9 Hz, NHC7′*H*O minor rotamer), 7.08 (1H, d, *J* = 9.9 Hz, N*H*C7′HO major rotamer), 6.19 (1H, t, *J* = 11.3 Hz, N*H*C7′HO minor rotamer), 4.72 (1H, d, *J* = 1.5 Hz, C1′H minor rotamer), 4.71 (1H, d, *J* = 2.2 Hz, C1′H major rotamer), 4.64 (1H, s, C3′O*H* minor rotamer), 4.43 (1H, s, C3′OH major rotamer), 3.94–3.90 (2 × 1H, qd, *J* = 6.2, 2.3 Hz, C5′H major and minor rotamers), 3.70 (1H, dd, *J* = 9.8, 2.1 Hz, C4′H major rotamer), 3.40 (3H, s, OCH_3 major rotamer), 3.39 (3H, s, OCH_3 minor rotamer), 3.29 (6H, s, C1′HOCH_3 major and minor rotamers), 3.13 (1H, dd, *J* = 12.4, 1.5 Hz, C4′H minor rotamer), 2.87 (1H, s, C2′H minor rotamer), 2.85 (1H, s, C2′H major rotamer), 1.28 (3H, s, C8′H_3 minor

rotamer), 1.26 (3H, s, C8′H_3 major rotamer), 1.05 (3H, d, J = 6.4 Hz, C6′H_3 minor rotamer), 1.01 (3H, d, J = 6.5 Hz, C6′H_3 major rotamer); **^{13}C NMR** (d^6-DMSO, 126 MHz) δ = 164.7 (NH*C*7′HO minor rotamer), 162.0 (NH*C*7′HO major rotamer), 98.7 (C1′H major and minor rotamers†), 82.7 (C2′H major rotamer), 82.4 (C2′H minor rotamer), 68.5 (C3′ major rotamer), 68.0 (C3′ minor rotamer), 64.9 (C5′H major rotamer), 64.3 (C5′H minor rotamer), 59.5 (OCH_3 major and minor rotamers†), 54.8 (C1′HO*C*H_3 major and minor rotamers†), 54.5 (C4′H major and minor rotamers†), 24.8 (C8′H_3 major and minor rotamers†), 17.4 (C6′H_3 minor rotamer), 17.0 (C6′H_3 major rotamer); **HRMS** (+ESI) Found $[M+H]^+$ = 234.1341; $C_{10}H_{20}NO_5$ requires 234.1342, Δ 0.43 ppm and $[M+Na]^+$ = 256.1161; $C_{10}H_{19}NO_5Na$ requires 256.1160, Δ 0.39 ppm.

The structure and relative stereochemistry was confirmed by X-ray crystallographic analysis after crystallisation from analytical grade Et_2O.

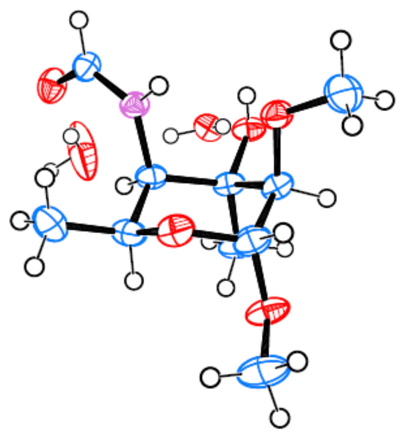

CCDC 882397 contains the supplementary crystallographic data for this thesis. This data can be obtained free of charge from the Cambridge Crystallographic Data Centre *via* www.ccdc.cam.ac.uk/data_request/cif.

† minor rotamer cannot be distinguished.

Variable Temperature ^{1}H NMR of 102 (formyl region shown for clarity)

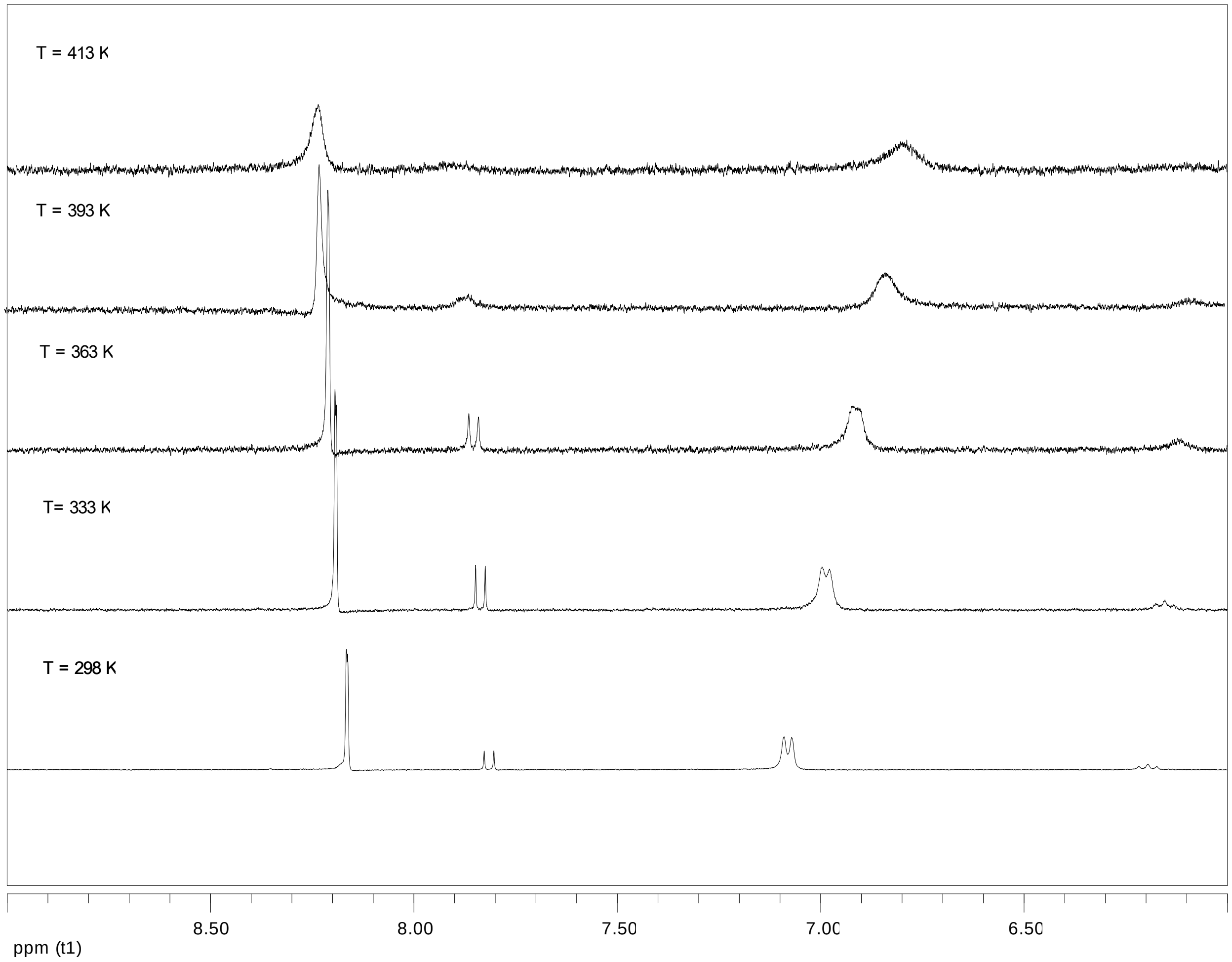

Phenyl 4-azido-4,6-dideoxy-3-*C*-methyl-2-*O*-methyl-1-thio-3-*O*-(trimethylsilyl)-α-L-talopyranoside 115

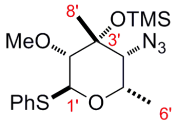

To a 20 mL Biotage® microwave vial was added **110** (103.4 mg, 0.448 mmol), 1,2-dichloroethane (5.8 mL), ZnI_2 (429 mg, 1.34 mmol), Bu_4NI (253 mg, 0.685 mmol), and TMSSPh (0.42 mL, 2.24 mmol). The reaction was heated to 65 °C and maintained at this temperature for 1 h, after which it was cooled to RT. The reaction was quenched with sat. aq. $Ba(OH)_2$•$8H_2O$ (8 mL). The mixture was extracted with CH_2Cl_2 (3 × 15 mL) and the combined organic layers washed with brine (20 mL), dried ($MgSO_4$) and concentrated *in vacuo*. Purification by column chromatography (SiO_2, dry load, hexane→hexane:Et_2O, 99:1→98:2) gave the title compound **115** (102.5 mg, 60%) as a colourless oil.

***R*f** = 0.56 (hexane:Et_2O, 4:1); $[\alpha]_D^{28.9}$ = –40.2 (*c* = 1.24, $CHCl_3$); **IR** (film) ν_{max}/cm^{-1} 2938, 2976, 2901, 2102, 1584, 1478, 1440, 1382, 1364, 1337, 1249, 1155, 1112, 1096, 1070, 1010, 984; **^{1}H NMR** ($CDCl_3$, 400 MHz) δ = 7.50 (2H, d, *J* = 7.2 Hz, *ortho* Ph), 7.33–7.23 (3H, m, *meta* and *para* Ph), 5.39 (1H, d, *J* = 5.5 Hz, C1′H), 4.33–4.26 (1H, m, C5′H), 3.54 (3H, s, OCH_3), 3.17 (1H, d, *J* = 4.0 Hz, C4′H), 3.05 (1H, d, *J* = 5.5 Hz, C2′H), 1.53 (3H, s, C8′H_3), 1.38 (3H, d, *J* = 6.7 Hz, C6′H_3), 0.19 (9H, s, $Si(CH_3)_3$); **^{13}C NMR** ($CDCl_3$, 100 MHz) δ = 135.0 (*ipso* Ph), 130.8 (2C, *ortho* Ph), 129.0 (2C, *meta* Ph), 127.2 (*para* Ph), 84.5 (C2′H), 82.6 (C1′H), 77.1 (C3′), 69.0 (C5′H), 66.7 (C4′H), 60.4 (OCH_3), 25.5 (C8′H_3), 16.1 (C6′H_3), 2.7 (3C, $Si(CH_3)_3$); **HRMS** (+ESI) Found $[M+Na]^+$ = 404.1435; $C_{17}H_{27}N_3O_3SiSNa$ requires 404.1435, Δ 0.00 ppm.

Key selected observed nOe’s:

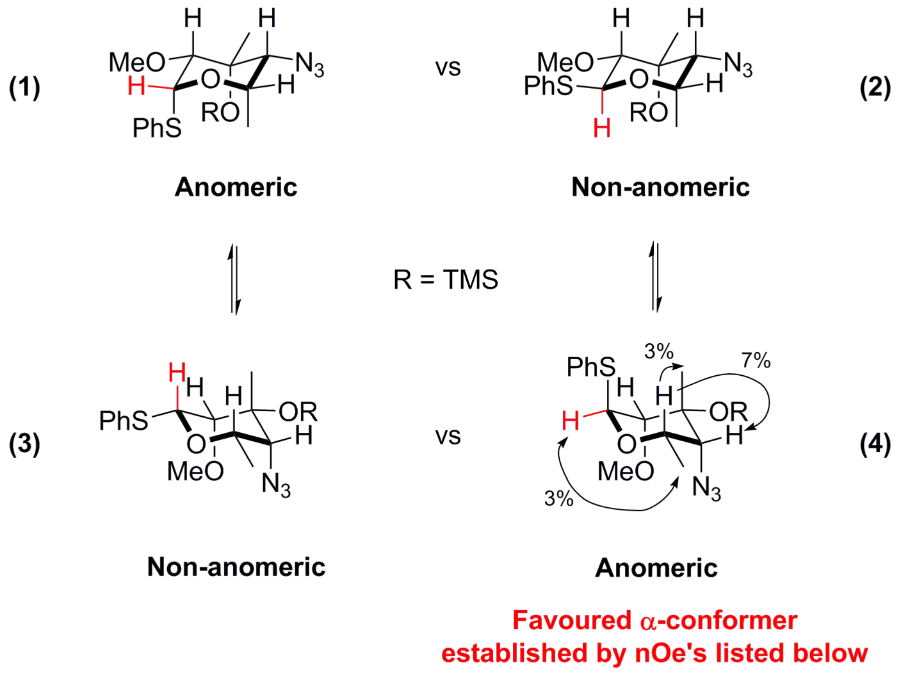

- An nOe was observed between C1′H and C6′H_3 suggesting that **conformers 1** and **3** are **disfavoured**.
- An nOe was observed between C5′H and C8′H_3 suggesting that **conformer 2** is **disfavoured**.
- **α-conformer 4** accounts for all observed nOe interactions indicating that **C1′H** is **equatorial**.

The corresponding D-configured enantiomer was synthesised and found to have the following optical rotation: $[\alpha]_D^{25.1}$ = +41.1 (c = 1.10, $CHCl_3$).

(2*S*,3*S*,4*S*)-2,4-Dimethyl-3,4-dihydro-2*H*-pyran-3,4-diol 116

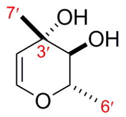

To a stirred solution of **106** (100 mg, 0.78 mmol) in Et_2O (8 mL) at −78 °C was added MeLi•LiBr (1.5 M in Et_2O; 1.56 mL, 2.34 mmol) dropwise. After stirring for 1 h, the reaction was removed from the cold bath then immediately quenched with sat. aq. NH_4Cl (3 mL) and warmed to RT. The resulting layers were separated and the aqueous phase extracted with EtOAc (3 × 5 mL). The combined organic layers were dried (Na_2SO_4) and concentrated *in vacuo* to give the title compound **116** (113 mg, 78%, dr > 95:5) as a white crystalline solid:

***R*$_f$** = 0.17 (hexane:EtOAc, 1:1); **m.p.** = 98–99 °C (from EtOAc) (lit.[25] 101 °C); $[\alpha]_D^{25.0}$ = −78.0 (*c* = 0.20, $CHCl_3$); **IR** (film) ν_{max}/cm^{-1} 3374 (br, OH), 2997, 2934, 2907, 1645 (C=C); **^{1}H NMR** ($CDCl_3$, 600 MHz) δ = 6.20 (1H, d, *J* = 6.0 Hz, C1'H), 4.71 (1H, d, *J* = 6.0 Hz, C2'H), 3.87–3.92 (1H, m, C5'H), 3.63 (1H, dd, *J* = 9.9, 4.1 Hz, C4'H), 2.09 (1H, d, *J* = 4.1 Hz, C4'OH), 1.39 (3H, d, *J* = 6.3 Hz, C6'H_3), 1.33 (s, 3H, C7'CH_3); **^{13}C NMR** ($CDCl_3$, 150 MHz) δ = 143.0 (C1'H), 107.6 (C2'H), 78.1 (C5'H), 73.6 (C4'H), 70.9 (C3'), 23.8 (C7'H_3), 17.7 (C6'H_3); **HRMS** (+ESI) Found $[M+Na]^+$ = 167.0679; $C_7H_{12}O_3Na$ requires 167.0679, Δ 0.0 ppm; **Elemental Analysis** found C, 58.30; H, 8.40. $C_7H_{12}O_3$ requires C, 58.30; H, 8.40%.

(2*S*,3*S*,4*S*)-3-(*tert*-Butyldimethylsilyloxy)-2,4-dimethyl-3,4-dihydro-2*H*pyran-4-ol S25

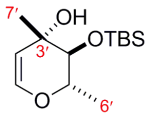

To a stirred solution of **116** (50.0 mg, 0.35 mmol) and 2,6-lutidine (163 µL, 1.40 mmol) in DMF (4 mL) at RT was added TBSOTf (122 µL, 0.53 mmol). After 1 h, additional 2,6-lutidine (163 µL, 1.40 mmol) and TBSOTf (122 µL, 0.53 mmol) were added, the reaction stirred for a further 1.5 h then quenched with sat. aq. NH_4Cl (10 mL) and diluted with EtOAc (10 mL). The

layers were separated and the aqueous phase extracted with EtOAc (10 mL). The combined organic layers were washed with sat. aq. LiCl (2 × 5 mL), dried (Na_2SO_4) and concentrated *in vacuo*. Purification by column chromatography (SiO_2, hexane→hexane:Et_2O, 8:2) gave the title compound **S25** (43.0 mg, 48%) as a colourless oil:

***R*f** = 0.14 (hexane:Et_2O, 8:2); $[\alpha]_D^{25.0}$ −65.4 (*c* = 1.30, $CHCl_3$); **IR** (film) ν_{max}/cm^{-1} 3479 (br, OH), 2956, 2931, 2891, 2858, 1649 (C=C); **^{1}H NMR** ($CDCl_3$, 600 MHz) δ = 6.19 (1H, d, *J* = 6.0 Hz, C1'H), 4.67 (1H, d, *J* = 6.0 Hz, C2'H), 3.83–3.78 (1H, m, C5'H), 3.61 (1H, d, *J* = 10.2 Hz, C4'H), 1.36 (1H, s, C3'OH), 1.31 (3H, d, *J* = 6.0 Hz, C6'H_3), 1.29 (3H, s, C7'H_3), 0.93 (9H, s, C(CH_3)$_3$ of *t*Bu), 0.17 (3H, s, Si(CH_3)$_3$), 0.12 (3H, s, Si(CH_3)$_3$); **^{13}C NMR** ($CDCl_3$, 150 MHz) δ = 142.7 (C1'H), 108.4 (C2'H), 79.1 (C4'H), 74.6 (C5'H), 71.6 (C3'), 26.0 (3C, C(*C*H_3)$_3$ of *t*Bu), 24.4 (C7'H_3), 18.4 (C6'H_3 or *C*(CH_3)$_3$ of *t*Bu), 18.3 (C6'H_3 or *C*(CH_3)$_3$ of *t*Bu), –3.7 (Si(*C*H_3)$_2$), –4.7 (Si(*C*H_3)$_2$); **HRMS** (+ESI) Found $[M+Na]^+$ = 281.1533; $C_{13}H_{26}O_3SiNa$ requires 281.1543, Δ 3.56 ppm.

(2*S*,3*S*,4*S*)-3,4-Di(*tert*-butyldimethylsilyloxy)-2,4-dimethyl-3,4-dihydro-2*H*pyran-4-ol S26

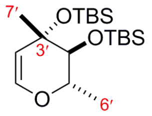

Isolated from the previous reaction as a colourless oil (61.0 mg, 47%).

***R*f** = 0.78 (hexane:Et_2O, 8:2); $[\alpha]_D^{25.0}$ +12.8 (*c* = 1.00, $CHCl_3$); **IR** (film) ν_{max}/cm^{-1} 2956, 2930, 2858, 2889, 1652 (C=C); **^{1}H NMR** ($CDCl_3$, 600 MHz) δ = 6.17 (1H, d, *J* = 6.1 Hz, C1'H), 4.73 (1H, d, *J* = 6.1 Hz, C2'H), 3.76–3.73 (1H, m, C5'H), 3.63 (1H, d, *J* = 13.4 Hz, C4'H), 1.30 (3H, d, *J* = 6.4 Hz, C6'H_3), 1.29 (3H, s, C7'H_3), 0.91 (9H, s, C(CH_3)$_3$ of *t*Bu), 0.88 (9H, s, C(CH_3)$_3$ of *t*Bu), 0.16 (3H, s, Si(CH_3)$_3$), 0.15 (3H, s, Si(CH_3)$_3$), 0.10 (3H, s, Si(CH_3)$_3$), 0.10 (3H, s, Si(CH_3)$_3$); **^{13}C NMR** ($CDCl_3$, 150 MHz) δ = 142.0 (C1'H), 109.0 (C2'H), 79.6 (C4'H), 75.0 (C5'H), 74.2 (C3'), 26.3 (3C, C(*C*H_3)$_3$ of *t*Bu), 26.1 (3C, C(*C*H_3)$_3$ of *t*Bu), 26.0 (C7'H_3), 18.6 (C6'H_3), 18.4 (*C*(CH_3)$_3$ of *t*Bu), 18.4 (*C*(CH_3)$_3$ of *t*Bu), –1.6 (Si(*C*H_3)$_2$), –1.7 (Si(*C*H_3)$_2$), –3.4

(Si(CH_3)$_2$), –4.7 (Si(CH_3)$_2$); **HRMS** (+ESI) Found $[M+Na]^+$ = 395.2413; $C_{19}H_{40}O_3Si_2Na$ requires 395.2408, Δ 1.27 ppm.

(2*R*,3*R*,4*S*,5*S*,6*S*)-5-(*tert*-Butyldimethylsilyloxy)-2-methoxy-4,6-dimethyltetrahydro-2*H*-pyran-3,4-diol 117

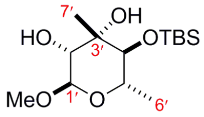

To a stirred solution of **S25** (355 mg, 1.37 mmol) in MeOH (14 mL) at RT was added $NaHCO_3$ (575 mg, 6.85 mmol). The resulting suspension was cooled to 0 °C and magnesium monoperoxyphthalate hexahydrate (80%; 1.02 g, 1.65 mmol) was added. After 2 h, the reaction was quenched with sat. aq. $NaHCO_3$ (5 mL) and diluted with EtOAc (10 mL). The layers were separated, the aqueous phase extracted with EtOAc (3 × 5 mL) and the combined organic layers partially concentrated *in vacuo* to remove MeOH. The resulting bilayer oil was redissolved in EtOAc (10 mL), the layers separated, the organic layer dried (Na_2SO_4) and concentrated *in vacuo* to give **117** as a white solid, which was used without further purification.

An analytical quantity was purified for characterisation by column chromatography (SiO_2, hexane:Et_2O, 1:1→2:3).

$\boldsymbol{R_f}$ = 0.38 (Et_2O:hexane, 7:3]; **m.p.** = 57–63 °C (from Et_2O); $[\alpha]_D^{25.0}$ −81.0 (*c* = 0.50, $CHCl_3$); **IR** (film) ν_{max}/cm^{-1} 3495 (br, OH), 2930, 2857; ^{1}**H NMR** ($CDCl_3$, 600 MHz) δ = 4.67 (1H, s, C1'H), 3.60–3.57 (2H, m overlap, C5'H and C2'H), 3.48 (1H, d, *J* = 9.3 Hz, C4'H), 3.36 (3H, s, OCH_3), 2.73 (1H, d, *J* = 3.5 Hz, C2'OH), 1.29 (3H, s, C7'H_3), 1.25 (3H, d, *J* = 6.1 Hz, C6'H_3), 0.91 (9H, s, C(CH_3)$_3$ of *t*Bu), 0.14 (3H, s, Si(CH_3)$_3$), 0.10 (3H, s, Si(CH_3)$_3$); ^{13}**C NMR** ($CDCl_3$, 150 MHz) δ = 101.0 (C1'H), 76.6 (C4'H), 75.4 (C2'H), 73.4 (C3'), 67.6 (C5'H), 55.1 (OCH_3), 26.0 (3C, C(CH_3)$_3$ of *t*Bu), 19.5 (C7'H_3), 18.5 (C6'H_3), 18.3 (*C*(CH_3)$_3$ of *t*Bu), –3.8 (Si(CH_3)$_2$), –4.6 (Si(CH_3)$_2$); **HRMS** (+ESI) Found $[M+Na]^+$ = 329.1766; $C_{14}H_{30}O_5SiNa$ requires 329.1760, Δ 1.82 ppm. **Elemental Analysis** found C, 54.60; H, 9.70. $C_7H_{12}O_3$ requires C, 54.90; H, 9.90%.

(2*S*,3*S*,4*S*,5*R*,6*R*)-3-(*tert*-Butyldimethylsilyloxy)-5,6-dimethoxy-2,4-dimethyltetrahydro-2*H*-pyran-4-ol 118

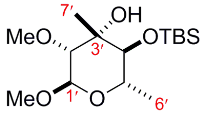

To a stirred solution of **117** (167.0 mg, 0.545 mmol) in DMF (5 mL) was added MeI (85 µL, 1.36 mmol) followed by freshly prepared Ag_2O (315 mg, 1.36 mmol) and the reaction was stirred in the absence of light. After 4.5 h, the suspension was filtered through a pad of Celite® and the residue washed with Et_2O. The filtrate was washed with sat. aq. LiCl (4 × 5 mL) and brine (5 mL), dried (Na_2SO_4) and concentrated *in vacuo*. Purification by column chromatography (SiO_2, hexane:Et_2O, 85:15→3:2) gave the title compound **118** (139.0 mg, 80% over 2 steps) as a colourless oil.

$\boldsymbol{R_f}$ = 0.18 (hexane:Et_2O, 7:3); $[\alpha]_D^{25.0}$ –44.5 (*c* = 0.90, $CHCl_3$); **IR** (film) ν_{max}/cm^{-1} 3548 (OH), 2932, 2900, 2857; **^{1}H NMR** ($CDCl_3$, 600 MHz) δ = 4.71 (1H, s, C1'H), 3.56–3.54 (1H, m, C5'H), 3.46 (3H, s, C2'OCH_3), 3.36 (3H, s, C1'OCH_3), 3.30 (1H, d, *J* = 9.3 Hz, C4'H), 3.08 (1H, s, C2'H), 2.82 (1H, s, C3'OH), 1.25 (3H, s, C7'H_3), 1.24 (3H, d, *J* = 6.3 Hz, C6'H_3), 0.90 (9H, s, $C(CH_3)_3$ of *t*Bu), 0.14 (3H, s, $Si(CH_3)_3$), 0.07 (3H, s, $Si(CH_3)_3$); **^{13}C NMR** ($CDCl_3$, 150 MHz) δ = 98.0 (C1'H), 85.2 (C2'H), 77.8 (C4'H), 72.5 (C3'), 67.6 (C5'H), 59.2 (C2'OCH_3), 54.9 (C1'OCH_3), 26.0 (3C, $C(CH_3)_3$ of *t*Bu), 18.5 (C6'H_3), 18.4 (C7'H_3 or ($C(CH_3)_3$ of *t*Bu)), 18.4 (C7'H_3 or ($C(CH_3)_3$ of *t*Bu)), –3.8 ($Si(CH_3)_2$), –4.8 ($Si(CH_3)_2$); **HRMS** (+ESI) Found $[M+Na]^+$ = 343.1908; $C_{15}H_{32}O_5SiNa$ requires 343.1917, Δ 2.62 ppm.

Phenyl 4-*O*-(*tert*-butyl(dimethyl)silyl)-6-deoxy-3-*C*-methyl-2-*O*-methyl-1-thio-3-*O*-(trimethylsilyl)-α-L-mannopyranoside 119

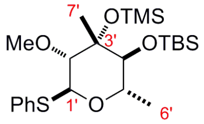

To a 20 mL Biotage® microwave vial was added **118** (103.7 mg, 0.324 mmol), 1,2-dichloroethane (4.2 mL), ZnI_2 (310 mg, 0.972 mmol), Bu_4NI (183 mg, 0.496 mmol), and

TMSSPh (0.307 mL, 1.62 mmol). The reaction was heated to 65 °C and maintained at this temperature for 1 h, after which it was cooled to RT. The reaction was quenched with sat. aq. $Ba(OH)_2{\bullet}8H_2O$ (8 mL). The mixture was extracted with CH_2Cl_2 (3 × 15 mL) and the combined organic layers washed with brine (20 mL), dried ($MgSO_4$) and concentrated *in vacuo*. Purification by column chromatography (SiO_2, dry load, hexane→hexane:Et_2O, 99:1→98:2) gave the title compound **119** (98 mg, 64%) as a white solid.

***R*f** = 0.31 (hexane:Et_2O, 95:5); **m.p.** = 59–62 °C (CH_2Cl_2); $[\alpha]_D^{26.9}$ = –76.6 (*c* = 1.03, $CHCl_3$); **IR** (film) ν_{max}/cm^{-1} 2955, 2931, 2857, 1585, 1473, 1463, 1439, 1382, 1361, 1248, 1172, 1097, 1086, 1011; **^{1}H NMR** ($CDCl_3$, 400 MHz) δ = 7.53 (2H, d, *J* = 7.2 Hz, *ortho* Ph), 7.31–7.21 (3H, m, *meta* and *para* Ph), 5.40 (1H, d, *J* = 4.4 Hz, C1′H), 3.98 (1H, m, appar. quintet *J* = 6.5 Hz, C5′H), 3.50 (1H, d, *J* = 6.4 Hz, C4′H), 3.42 (3H, s, OCH_3), 3.38 (1H, d, *J* = 4.4 Hz, C2′H), 1.42 (3H, s, C7′H_3), 1.32 (3H, d, *J* = 6.6 Hz, C6′H_3), 0.92 (9H, s, C(CH_3)$_3$ of *t*Bu), 0.15 (9H, s, $Si(CH_3)_3$), 0.12 (3H, s, Si(C*H*$_3$)$_2$), 0.09 (3H, s, Si(C*H*$_3$)$_2$); **^{13}C NMR** ($CDCl_3$, 100 MHz) δ = 136.0 (*ipso* Ph), 130.9 (2C, *ortho* Ph), 128.9 (2C, *meta* Ph), 126.9 (*para* Ph), 84.5 (C2′H), 83.0 (C1′H), 77.8 (C4′H), 77.6 (C3′), 71.8 (C5′H), 58.7 (OCH_3), 26.0 (3C, C(*C*H$_3$)$_3$ of *t*Bu), 22.4 (C7′H_3), 18.6 (*C*(CH$_3$)$_3$ of *t*Bu), 18.2 (C6′H_3), 2.7 (3C, $Si(CH_3)_3$), –3.7 (Si(*C*H$_3$)$_2$), –4.3 (Si(*C*H$_3$)$_2$); **HRMS** (+ESI) Found $[M+Na]^+$ = 493.2216; $C_{23}H_{42}O_4Si_2SNa$ requires 493.2235, Δ 3.85 ppm; **Elemental Analysis** found C, 58.77; H, 9.04. $C_{23}H_{42}O_4Si_2S$ requires C, 58.67; H, 8.99%.

The structure and absolute stereochemistry was confirmed by X-ray crystallographic analysis after crystallisation from analytical grade CH_2Cl_2.

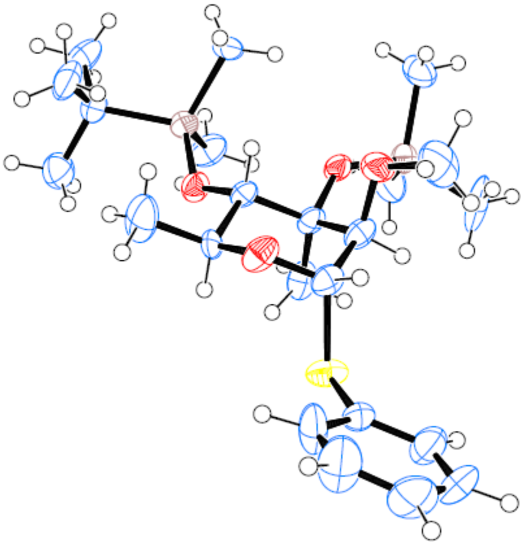

CCDC 882401 contains the supplementary crystallographic data for this thesis. This data can be obtained free of charge from the Cambridge Crystallographic Data Centre *via* www.ccdc.cam.ac.uk/data_request/cif.

Phenyl 6--deoxy-3-*C*-methyl-2-*O*-methyl-1-thio-α-L-mannopyranoside 122

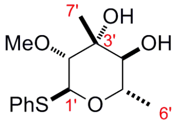

To a solution of **119** (70.1 mg, 0.15 mmol) in THF (2.7 mL) at RT was added TBAF (1 M in THF, 0.60 mL, 0.60 mmol) dropwise. The reaction mixture was stirred for 6 h, and additional TBAF (1 M in THF, 0.20 mL, 0.20 mmol) was added. After a further 12 h TLC analysis indicated that the starting material had been consumed and the reaction mixture was concentrated *in vacuo*. Purification by column chromatography (SiO_2, hexane→hexane:EtOAc, 1:1→EtOAc) gave the title compound **122** (38 mg, 89%) as a white solid.

***R*$_f$** = 0.29 (hexane:EtOAc, 1:1); **m.p.** = 102–106 °C; $[\alpha]_D^{25.0}$ = –120.3 (*c* = 1.02, $CHCl_3$); **IR** (film) ν_{max}/cm^{-1} 3492, 3407, 2972, 2924, 2897, 2853, 1478, 1453, 1439, 1405, 1373, 1256, 1177,

1157, 1111, 1086, 1064, 1027; **^{1}H NMR** ($CDCl_3$, 400 MHz) δ = 7.50 (2H, d, *J* = 7.1 Hz, *ortho* Ph), 7.35–7.27 (3H, m, *meta* and *para* Ph), 5.57 (1H, s, C1′H), 4.11–4.03 (1H, m, C5′H), 3.49 (1H, s, C4′H), 3.47 (3H, s, OCH_3), 3.44 (1H, s, C2′H), 3.10–3.03 (1H, br s, C3′OH), 2.43–2.31 (1H, br s, C4′HO*H*), 1.46 (3H, s, C7′H_3), 1.34 (3H, d, *J* = 6.2 Hz, C6′H_3); **^{13}C NMR** ($CDCl_3$, 100 MHz) δ = 135.8 (*ipso* Ph), 131.1 (2C, *ortho* Ph), 129.1 (2C, *meta* Ph), 127.4 (*para* Ph), 86.8 (C2′H), 84.2 (C1′H), 77.0 (C4′H), 72.7 (C3′), 68.0 (C5′H), 58.5 (OCH_3), 18.4 (C7′H_3), 17.9 (C6′H_3); An accurate mass could not be obtained for this compound.

Phenyl 6--deoxy-3-*C*-methyl-2-*O*-methyl-1-thio-4-*O*-(triethylsilyl)-α-L-mannopyranoside S27

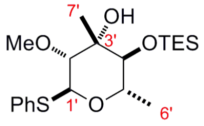

To a solution of **122** (21.8 mg, 0.767 mmol) in pyridine (0.5 mL) at RT was added DMAP (3.75 mg, 39.7 μmol) and TESCl (54 μL, 0.324 mmol) sequentially. After 1 h 45 min at RT the reaction was diluted with CH_2Cl_2 (20 mL) and washed with sat. aq. $NaHCO_3$ (10 mL). The aqueous layer was extracted with CH_2Cl_2 (10 mL) and the combined organic layers dried ($MgSO_4$) and concentrated *in vacuo*. Azeotropic removal of pyridine with hexane (3 × 10 mL) followed by purification by column chromatography (SiO_2, hexane→hexane:EtOAc, 4:1) gave the title compound **S27** (26.1 mg, 85%) as a colourless oil.

***R*$_f$** = 0.63 (hexane:EtOAc, 4:1); $[\alpha]_D^{25.8}$ = –109.1 (*c* = 1.25, $CHCl_3$); **IR** (film) ν_{max}/cm^{-1} 3545, 2934, 2876, 1728, 1584, 1479, 1457, 1440, 1415, 1397, 1373, 1332, 1294, 1265, 1240, 1175, 1162, 1162, 1105, 1085, 1007; **^{1}H NMR** ($CDCl_3$, 400 MHz) δ = 7.50 (2H, d, *J* = 7.1 Hz, *ortho* Ph), 7.34–7.27 (3H, m, *meta* and *para* Ph), 5.54 (1H, s, C1′H), 4.05–3.97 (1H, m, C5′H), 3.46 (3H, s, OCH_3), 3.44 (1H, d, *J* = 9.5 Hz, C4′H), 3.40 (1H, d, *J* = 1.1 Hz, C2′H), 2.94 (1H, s, C3′OH), 1.38 (3H, s, C7′H_3), 1.28 (3H, d, *J* = 6.2 Hz, C6′H_3), 0.99 (9H, t, *J* = 7.9 Hz, Si(CH_2CH_3)$_3$, 0.73–0.65 (6H, m, Si(CH_2CH_3)$_3$); **^{13}C NMR** ($CDCl_3$, 100 MHz) δ = 136.0 (*ipso* Ph), 131.0 (2C, *ortho* Ph), 129.0 (2C, *meta* Ph), 127.3 (*para* Ph), 87.4 (C2′H), 84.4 (C1′H), 78.2

(C4′H), 72.8 (C3′), 69.2 (C5′H), 58.6 (OCH_3), 19.0 (C7′H_3), 18.2 (C6′H_3), 7.0 (3C, Si(CH_2*C*H_3)$_3$), 5.2 (3C, Si(*C*H_2CH_3)$_3$); **HRMS** (+ESI) Found $[M+Na]^+$ = 421.1836; $C_{20}H_{34}O_4SiSNa$ requires 421.1839, Δ 0.71 ppm.

Phenyl 6-deoxy-4-*O*-(diethyl(propyl)silyl)-3-*C*-methyl-2-*O*-methyl-1-thio-3-*O*-(trimethylsilyl)-α-L-mannopyranoside 123

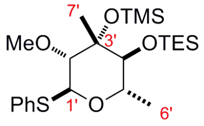

Procedure 1

To a solution of **S27** (8.3 mg, 20.8 µmol) in CH_2Cl_2 (0.70 mL) at –78 °C was added 2,6-lutidine (7 µL, 62.4 µmol) and TMSOTf (6 µL, 30.6 µmol) sequentially. The reaction mixture was stirred for 30 min at –78 °C after which pH 7 phosphate buffer (1 mL) was added and the reaction warmed to RT and diluted with CH_2Cl_2 (10 mL). The layers were separated and the organic layer washed with brine (5 mL). The aqueous layer was then further extracted with EtOAc (10 mL) and the combined organic layers dried ($MgSO_4$) and concentrated *in vacuo*. Purification by column chromatography (SiO_2, hexane→hexane:EtOAc, 4:1) gave the title compound **123** (8.3 mg, 85%) as a colourless oil.

Procedure 2

To a 20 mL Biotage® microwave vial was added **125** (102.8 mg, 0.321 mmol), 1,2-dichloroethane (4.2 mL), ZnI_2 (307.4 mg, 0.963 mmol), Bu_4NI (181.4 mg, 0.491 mmol), and TMSSPh (0.30 mL, 1.60 mmol). The reaction was heated to 65 °C and maintained at this temperature for 2 h 40 min, after which it was cooled to RT. The reaction was quenched with sat. aq. $Ba(OH)_2$•$8H_2O$ (8 mL). The mixture was extracted with CH_2Cl_2 (3 × 15 mL) and the combined organic layers washed with brine (20 mL), dried ($MgSO_4$) and concentrated *in vacuo*. Purification by column chromatography (SiO_2, dry load, hexane→hexane:Et_2O, 99:1→98:2) gave the title compound **123** (130.2 mg, 86%) as a colourless oil.

***R*$_f$** = 0.80 (hexane:EtOAc, 4:1); $[\alpha]_D^{25.1}$ = –38.2 (*c* = 0.83, $CHCl_3$); **IR** (film) ν_{max}/cm^{-1} 2954, 2925, 2876, 1733, 1585, 1479, 1457, 1441, 1414, 1380, 1300, 1248, 1168, 1099, 1011; **^{1}H NMR** ($CDCl_3$, 400 MHz) δ = 7.52 (2H, d, *J* = 7.2 Hz, *ortho* Ph), 7.32–7.20 (3H, m, *meta* and *para* Ph), 5.40 (1H, d, *J* = 4.4. Hz, C1′H), 3.97 (1H, m, appar. quintet *J* = 6.6 Hz, C5′H), 3.53 (1H, d, *J* = 6.6 Hz, C4′H), 3.42 (3H, s, OCH_3), 3.39 (1H, d, *J* = 4.4 Hz, C2′H), 1.41 (3H, s, C7′H_3), 1.31 (3H, d, *J* = 6.6 Hz, C6′H_3), 0.99 (9H, t, *J* = 8.0 Hz, $Si(CH_2CH_3)_3$, 0.65 (6H, q, *J* = 7.8 Hz, $Si(CH_2CH_3)_3$); **^{13}C NMR** ($CDCl_3$, 100 MHz) δ = 136.1 (*ipso* Ph), 130.8 (2C, *ortho* Ph), 128.9 (2C, *meta* Ph), 126.9 (*para* Ph), 84.6 (C2′H), 83.0 (C1′H), 78.0 (C4′H), 77.6 (C3′), 71.8 (C5′H), 58.6 (OCH_3), 22.3 (C7′H_3), 18.3 (C6′H_3), 7.0 (3C, $Si(CH_2CH_3)_3$), 5.3 (3C, $Si(CH_2CH_3)_3$), 2.7 (3C, $Si(CH_3)_3$); **HRMS** (+ESI) Found $[M+Na]^+$ = 493.2229; $C_{23}H_{42}O_4Si_2SNa$ requires 493.2235, Δ 1.22 ppm.

Methyl 6-deoxy-4-*O*-(diethyl(propyl)silyl)-3-*C*-methyl-α-L-mannopyranoside 124

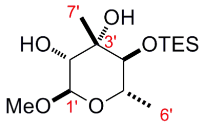

To a solution of **116** (565 mg, 3.92 mmol) in pyridine (27.6 mL) at RT was added DMAP (192 mg, 1.57 mmol) and TESCl (2.10 mL, 12.5 mmol) sequentially. After 1 h at RT the reaction was diluted with CH_2Cl_2 (100 mL) and washed with sat. aq. $NaHCO_3$ (80 mL). The aqueous layer was extracted with CH_2Cl_2 (100 mL) and the combined organic layers dried ($MgSO_4$) and concentrated *in vacuo*. Azeotropic removal of pyridine with hexane (3 × 10 mL) followed by column chromatography (SiO_2, hexane→hexane:EtOAc, 9:1) gave the desired semi-pure mono-protected sugar (assumed quant.). The mixture was used directly in the next step in the reaction sequence.

To a suspension of the mono-protected sugar (assumed quant., 3.92 mmol) and $NaHCO_3$ (0.988 g, 11.76 mmol) in MeOH (15.1 mL) at 0 °C was added *m*-CPBA (70%, 1.16 g, 4.70 mmol). The mixture was maintained at 0 °C for 5 min and then warmed to RT. After 1 h the reaction was quenched with H_2O (60 mL), the layers separated and the aqueous layer further extracted with EtOAc (3 × 100 mL). The combined organic layers were dried ($MgSO_4$) and

concentrated *in vacuo*. Purification by column chromatography (SiO_2, hexane:Et_2O, 7:3) gave the title compound **124** (540 mg, 45% over 2 steps) as a white solid.

***R*$_f$** = 0.20 (hexane:Et_2O, 1:1); **m.p.** = 99–102 °C; $[\alpha]_D^{26.5}$ = –72.8 (*c* = 0.85, $CHCl_3$); **IR** (film) ν_{max}/cm^{-1} 3247, 2956, 2898, 2878, 1448, 1416, 1386, 1352, 1340, 1309, 1284, 1263, 1242, 1195, 1163, 1133, 1116, 1093, 1051, 1018, 1000, 963; **^{1}H NMR** ($CDCl_3$, 400 MHz) δ = 4.68 (1H, s, C1′H), 3.63–3.53 (2H, m, C2′H and C5′H), 3.51 (1H, d, *J* = 9.4 Hz, C4′H), 3.36 (3H, s, C1′HOC*H*$_3$), 2.60 (1H, d, *J* = 3.8 Hz, C2′HO*H*), 2.11 (1H, s, C3′OH), 1.30 (3H, s, C7′H$_3$), 1.26 (3H, d, *J* = 6.1 Hz, C6′H$_3$), 0.98 (9H, t, *J* = 7.9 Hz, Si(CH$_2$C*H*$_3$)$_3$), 0.67 (6H, q, *J* = 7.9 Hz, Si(C*H*$_2$CH$_3$)$_3$); **^{13}C NMR** ($CDCl_3$, 100 MHz) δ = 101.0 (C1′H), 77.3 (C4′H), 75.5 (C2′H), 73.5 (C3′), 67.7 (C5′H), 55.1 (C1′HO*C*H$_3$), 19.5 (C7′H$_3$), 18.3 (C6′H$_3$), 7.0 (3C, Si(CH$_2$*C*H$_3$)$_3$), 5.2 (3C, Si(*C*H$_2$CH$_3$)$_3$); **HRMS** (+ESI) Found $[M+Na]^+$ = 329.1747; $C_{14}H_{30}O_5SiNa$ requires 329.1755, Δ 2.43 ppm; **Elemental Analysis** found C, 55.06; H, 9.85. $C_{14}H_{30}O_5$ requires C, 54.87; H, 9.87%.

The structure and relative stereochemistry was confirmed by X-ray crystallographic analysis after crystallisation from analytical grade $CHCl_3$.

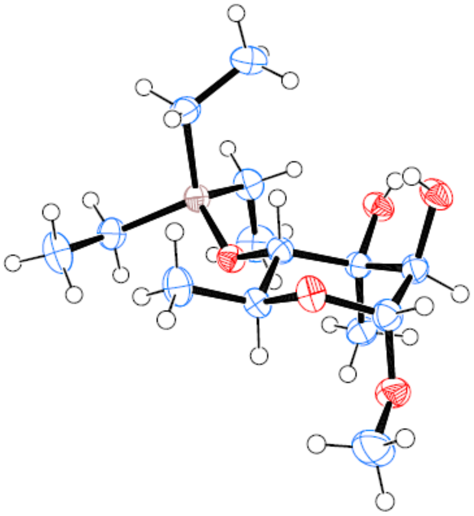

CCDC 882402 contains the supplementary crystallographic data for this thesis. This data can be obtained free of charge from the Cambridge Crystallographic Data Centre *via* www.ccdc.cam.ac.uk/data_request/cif.

Methyl 6-deoxy-4-*O*-(diethyl(propyl)silyl)-3-*C*-methyl-2-*O*-methyl-α-L-mannopyranoside 125

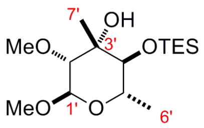

To a mixture of freshly sublimed KO*t*Bu (178.4 mg, 1.59 mmol) in THF (4.2 mL) at 0 °C was added a solution of **124** (440 mg, 1.44 mmol) in THF (5 mL) dropwise. The reaction mixture was stirred for 40 min at 0 °C after which MeI (0.11 mL, 1.73 mmol) was added dropwise to the pale yellow solution and stirred for 1 h. After this time, further KO*t*Bu (16.2 mg, 0.144 mmol) and MeI (18.0 µL, 0.289 mmol) were added and the reaction maintained at 0 °C until TLC analysis indicated full conversion of the starting material. The reaction was quenched with H_2O (9 mL), diluted with CH_2Cl_2 (20 mL) and warmed to RT. The layers were separated, the aqueous layer further extracted with CH_2Cl_2 (3 × 20 mL), and the combined organics dried ($MgSO_4$) and concentrated *in vacuo*. Purification by column chromatography (Florisil, hexane:Et_2O, 7:3) gave the title compound **125** (373 mg, 81%) as a colourless oil.

$\boldsymbol{R_f}$ = 0.52 (hexane:Et_2O, 1:1); $[\alpha]_D^{25.9}$ = –37.1 (*c* = 1.16, $CHCl_3$); **IR** (film) ν_{max}/cm^{-1} 3549, 2952, 2909, 2877, 2830, 1458, 1415, 1389, 1358, 1340, 1308, 1261, 1237, 1182, 1142, 1118, 1099, 1061, 1007; **^{1}H NMR** ($CDCl_3$, 400 MHz) δ = 4.71 (1H, s C1′H), 3.60–3.50 (1H, m, C5′H), 3.47 (3H, s, OCH_3), 3.36 (3H, s, C1′HOCH_3), 3.34 (1H, d, *J* = 9.4 Hz C4′H), 3.09 (1H, d, *J* = 0.8 Hz, C2′H), 2.84 (1H, s, C3′OH), 1.25 (3H, s, C7′H_3), 1.24 (3H, d, *J* = 6.3 Hz, C6′H_3); **^{13}C NMR** ($CDCl_3$, 100 MHz) δ = 98.0 (C1′H), 85.2 (C2′H), 77.9 (C4′H), 72.5 (C3′), 67.7 (C5′H), 59.2 (OCH_3), 54.9 (C1′HOCH_3), 18.4 (C7′H_3), 18.3 (C6′H_3), 7.0 (3C, Si(CH_2CH_3)$_3$), 5.2 (3C, Si(CH_2CH_3)$_3$); **HRMS** (+ESI) Found $[M+Na]^+$ = 343.1903; $C_{15}H_{32}O_5SiNa$ requires 343.1911, Δ 2.33 ppm.

Callipeltoside A (1)

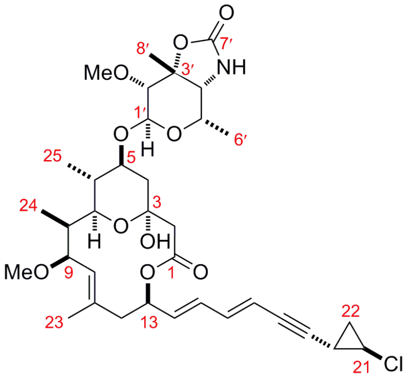

To a solution of aglycon **4** (6.7 mg, 14 μmol) and thioglycoside **112** (13.0 mg, 28 μmol) in CH_2Cl_2 (3.3 mL) at RT was added 4 Å MS followed by DTBMP (7.3 mg, 35.6 μmol) and the mixture stirred for 50 min.[†] The reaction was cooled to –15 °C and NIS (6.3 mg, 28 μmol) and TfOH [(47 μL, 5.3 μmol) of a stock solution of TfOH in CH_2Cl_2 (0.1 mL, 1.125 mmol in 10 mL CH_2Cl_2)] added sequentially. The resulting light pink solution was allowed to warm to RT slowly over 16 h. The reaction was quenched by the addition of sat. aq. $NaHCO_3$ (12 mL) and diluted with EtOAc (10 mL). The layers were separated and the aqueous layer further extracted with EtOAc (3 × 10 mL). The combined organic layers were washed with sat. aq. $Na_2S_2O_3$ (30 mL), dried ($MgSO_4$) and concentrated *in vacuo*. Purification by column chromatography (SiO_2, hexane→hexane:EtOAc, 7:3) gave TIPS-protected callipeltoside A (assumed quant.) as an off-white foam/gum [$\boldsymbol{R}_\mathbf{f}$ = 0.20 (hexane:EtOAc, 7:3)].[26]

To a solution of TIPS-protected callipeltoside A (assumed quant., 14 μmol) in THF (0.88 mL) at RT was added TBAF (1 M in THF, 0.88 mL, 27 μmol). After 10 min, TLC analysis indicated that the reaction was complete and the solvent removed *in vacuo*. Purification by column chromatography (SiO_2, hexane→hexane:EtOAc, 2:3→EtOAc) gave the title compound callipeltoside A **1** (7.9 mg, 83% over 2 steps) as an off-white gum.[27] The sample was further purified using HPLC (see below).

[†] The aglycon **4** and thioglycoside **112** were azeotroped with PhMe (× 3) prior to use.

***R*_f** = 0.30 (EtOAc); $[\alpha]_D^{25.3}$ = –17.5 (*c* = 0.33, MeOH), [lit.[22] $[\alpha]_D^{20}$ = –17.6 (*c* = 0.04, MeOH)]; **IR** (film) ν_{max}/cm^{-1} 3361, 2923, 2854, 1744, 1701, 1667, 1633, 1615, 1606, 1456, 1419, 1375, 1321, 1261, 1227, 1181, 1154, 1095, 1058, 1025, 980; **^{1}H NMR** (CD_3OD, 500 MHz) δ = 6.53 (1H, dd, *J* = 15.4, 10.8 Hz, C16H), 6.36 (1H, dd, *J* = 14.4, 10.8 Hz, C15H), 5.86 (1H, m overlapped, C14H), 5.85 (1H, m overlapped, C13H), 5.68 (1H, dd, *J* = 15.5, 1.9 Hz, C17H), 5.30 (1H, d, *J* = 9.8 Hz, C10H), 4.74 (1H, d, *J* = 6.2 Hz, C1′H), 3.98 (1H, qd, *J* = 6.5, 1.7 Hz, C5′H), 3.91 (1H, dd, *J* = 9.6, 2.4 Hz, C9H), 3.75 (1H, dt, *J* = 10.7, 4.7 Hz, C5H), 3.68 (1H, dd, *J* = 10.4, 2.4 Hz, C7H), 3.62 (3H, s, OCH_3), 3.47 (1H, d, *J* = 1.9 Hz, C4′H), 3.45 (1H, d, *J* = 6.2 Hz, C2′H), 3.28 (1H, m, C21H), 3.24 (3H, s, C9H(OCH_3)), 2.56 (1H, d, *J* = 12.9 Hz, C2H_AH_B), 2.49 (1H, d, *J* = 12.9 Hz, C2H_AH_B), 2.37 (1H, m overlapped, C12H_AH_B), 2.30 (1H, m overlapped, C12H_AH_B), 2.25 (1H, m overlapped, C8H), 2.25 (1H, m overlapped, equatorial C4H_AH_B), 1.85 (1H, m, C20H), 1.77 (3H, s, C23H_3), 1.56 (1H, m, C6H), 1.53 (3H, s, C8′H_3), 1.43 (1H, t, *J* = 11.7 Hz, axial C4H_AH_B), 1.30 (2H, m, C22H_AH_B), 1.12 (3H, d, *J* = 6.5 Hz, C6′H_3), 1.02 (3H, d, *J* = 6.5 Hz, C25H_3), 0.99 (3H, d, *J* = 7.0 Hz, C24H_3); **^{13}C NMR** (CD_3OD, 126 MHz) δ = 172.9 (C1O_2), 161.1 (*C*7′O_2), 141.6 (C16H), 134.4 (C14H), 134.3 (C11), 132.0 (C15H), 128.4 (C10H), 113.5 (C17H), 103.6 (C1′H), 96.6 (C3), 92.8 (C19), 83.9 (C3′), 83.1 (C2′H), 81.4 (C9H), 78.8 (C5H), 78.4 (C18), 76.4 (C7H), 72.7 (C13H), 65.3 (C5′H), 62.7 (C4′H), 62.0 (OCH_3), 55.4 (C9H(O*C*H_3)), 47.8 (C12H_2), 46.1 (C2H_2), 44.5 (C4H_2), 39.9 (C6H), 38.2 (C8H), 35.1 (C21H)*, 23.0 (C8′H_3), 19.8 (C22H_2), 16.3 (C23H_3), 15.9 (C6′H_3), 12.8 (C20H)*, 12.8 (C25H_3), 6.8 (C24H_3); **HRMS** (+ESI) Found $[M+Na]^+$ = 700.2853; $C_{35}H_{48}O_{10}NClNa$ requires 700.2859, Δ 0.86 ppm.[†]

[†] The data is assigned in the same style as originally reported for the natural product.

^{1}H NMR comparison between synthetic and natural callipeltoside A (CD_3OD)

	Natural callipeltoside A[27]	Synthetic callipeltoside A
**Atom**	**δ in ppm (500 MHz)**	**δ in ppm (500 MHz)**
1		
2	2.56 (d, 12.7); 2.50 (d, 12.7)	2.56 (d, 12.9); 2.49 (d, 12.9)
3		
4	2.25, (m, overlapped); 1.43 (t, 11.7)	2.25 (m, overlapped); 1.43 (t, 11.7)
5	3.76 (ddd, 11.7, 5.9, 4.9)	3.75 (dt, 10.7, 4.7)
6	1.58 (m)	1.56 (m)
7	3.68 (dd, 10.5, 2.4)	3.68 (dd, 10.4, 2.4)
8	2.27 (m)	2.25 (m overlapped)
9	3.91 (dd, 9.5, 2.2)	3.91 (dd, 9.6, 2.4)
10	5.30 (br dd, 9.5)	5.30 (d, 9.8)
11		
12	2.37 (dd, overlapped)<br>2.31 (dd, overlapped)	2.37 (dd, overlapped)<br>2.30 (dd, overlapped)
13	5.86 (m, overlapped)	5.85 (m overlapped)
14	5.87 (m, overlapped)	5.86 (m, overlapped)
15	6.37 (dd, 14.2, 10.5)	6.36 (dd, 14.4, 10.8)
16	6.53 (dd, 15.3, 10.5)	6.53 (dd, 15.4, 10.8)
17	5.68 (dd, 15.3, 1.7)	5.68 (dd, 15.5, 1.9)
18		
19		
20	1.85 (m, overlapped)	1.85 (m)
21	3.28 (m)	3.28 (m)
22	1.31 (m)	1.30 (m)
23	1.78 (s)	1.77 (s)
24	0.99 (d, 6.9)	0.99 (d, 7.0)
25	1.03 (d, 6.4)	1.02 (d, 6.5)
C9H(OCH_3)	3.25 (s)	3.24 (s)
3-OH		
1′	4.74 (d, 6.1)	4.74 (d, 6.2)
2′	3.42 (d, 6.1)	3.45 (d, 6.2)
3′		
4′	3.48 (d, 1.7)	3.47 (d, 1.9)
5′	3.99 (dq, 6.5, 1.7)	3.98 (qd, 6.5, 1.7)
6′	1.12 (d, 6.5)	1.12 (d, 6.5)
7′		
8′	1.54 (s)	1.53 (s)
OCH_3	3.63 (s)	3.62 (s)
NH		

^{13}C NMR comparison between synthetic and natural callipeltoside A (CD_3OD)

	Natural callipeltoside A[27]	Synthetic callipeltoside A
**Atom**	**δ in ppm (500 MHz)**	**δ in ppm (500 MHz)**
1	172.4	172.9
2	46.0	46.1
3	97.0	96.6
4	44.5	44.5
5	78.7	78.8
6	39.8	39.9
7	76.4	76.4
8	38.2	38.2
9	81.4	81.4
10	128.3	128.4
11	134.1	134.3
12	47.8	47.8
13	72.7	72.7
14	134.2	134.4
15	132.0	132.0
16	141.5	141.6
17	113.4	113.5
18	78.3	78.4
19	92.4	92.8
20	(34.7)*	12.8
21	(55.4)*	35.1
22	19.3	19.8
23	16.3	16.3
24	6.8	6.8
25	12.8	12.8
C9H(OCH_3)	55.4	55.4
3-OH		
1′	103.6	103.6
2′	83.2	83.1
3′	83.8	83.9
4′	62.7	62.7
5′	65.3	65.3
6′	15.9	15.9
7′	161.2	161.1
8′	23.0	23.0
OCH_3	62.0	62.0
NH		

*Carbons C20 and C21 have been reassigned following work conducted by Paterson.[28]

A further NMR experiment was conducted in order to elucidate the configuration at the anomeric carbon. Measurement of the $^{1}J_{C\text{-}H}$ value from a HSQC (Heteronuclear Single Quantum Coherence) experiment without ^{13}C decoupling is known to be diagnostic of the configuration of the glycosidic linkage. A value of ~170 Hz suggests an equatorial proton at C1′H, whilst ~160 Hz indicates an axial proton.The measurement gave $\boldsymbol{^{1}J_{C\text{-}H} = 174.2}$ **Hz**, indicating an **equatorial proton** at the anomeric carbon.[29]

HPLC conditions

Preparative HPLC purification was performed on an Agilent HP 1100 series chromatograph equipped with a Waters μBondapak C18 column (column length 150 mm, internal diameter of column 3.9 mm, particle size 10 μm, temperature 25 °C). Elution was carried out at a flow rate of 1.0 mL/min using MeOH:H_2O (75:25) and detection was with diode array detection (λ= 250, 272 and 286). The sample was made up to a concentration of 0.015mg/μL with purification carried out using 25 μL injections which saturated the detector at λ= 250, 272 and 286. t_R = 11.8 min.

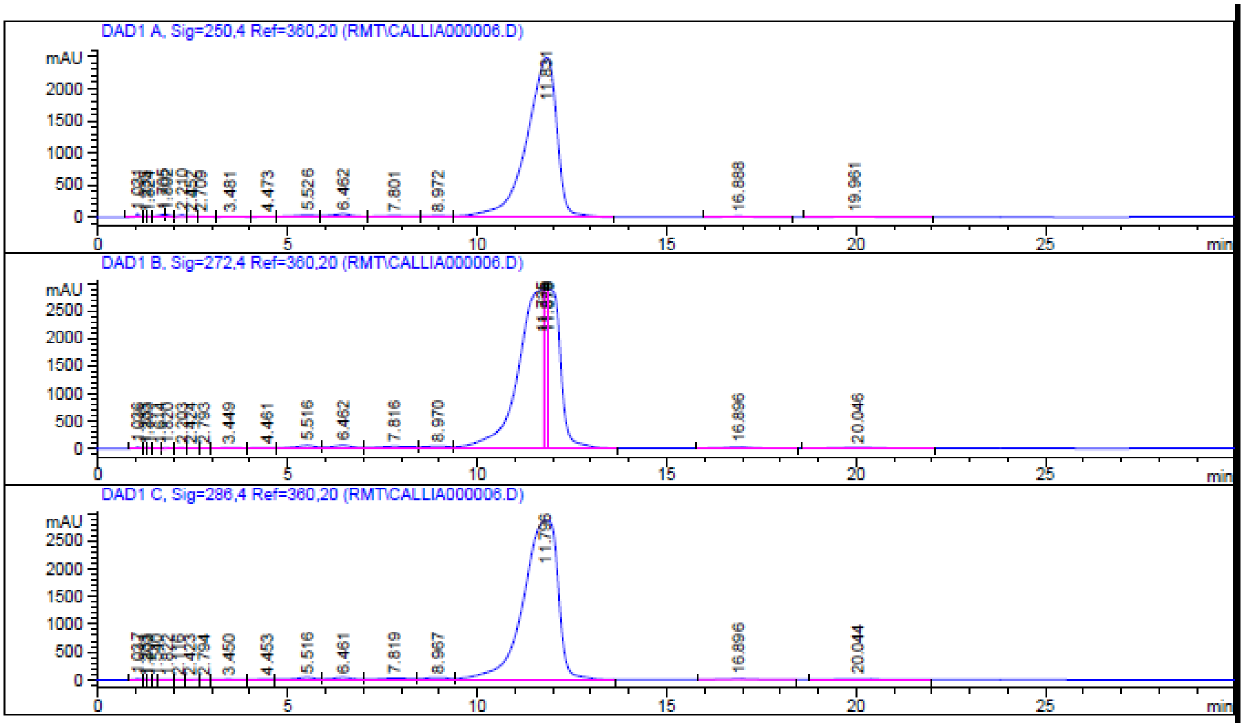

(1*S*,5*R*,7*E*,9*R*,10*R*,11*R*,12*R*,13*S*)-5-{(1*E*,3*E*)-6-[(1*S*,2*R*)-2-chlorocyclopropyl]hexa-1,3-dien-5-yn-1-yl}-1-hydroxy-9-methoxy-7,10,12-trimethyl-3-oxo-4,15-dioxabicyclo[9.3.1]pentadec-7-en-13-yl 4-azido-4,6-dideoxy-3-*C*-methyl-2-*O*-methyl-3-*O*-(trimethylsilyl)-α-L-talopyranoside 126

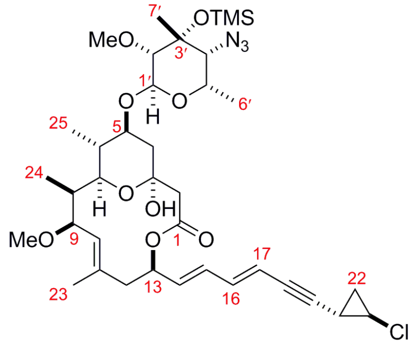

To a solution of aglycon **4** (6.1 mg, 12.7 μmol) and thioglycoside **115** (9.7 mg, 25.4 μmol) in CH_2Cl_2 (3.2 mL) at RT was added 4 Å MS followed by DTBMP (6.62 mg, 32.3 μmol) and the mixture stirred for 50 min.[†] The reaction was cooled to –15 °C and NIS (5.6 mg, 25.4 μmol) and TfOH [(43 μL, 4.8 μmol) of a stock solution of TfOH in CH_2Cl_2 (0.1 mL, 1.125 mmol in 10 mL CH_2Cl_2)] added sequentially. The resulting light pink solution was allowed to warm to RT slowly over 16 h. The reaction was quenched by the addition of sat. aq. $NaHCO_3$ (8 mL) and diluted with EtOAc (15 mL). The layers were separated and the aqueous layer further extracted with EtOAc (3 × 20 mL). The combined organic layers were washed with sat. aq. $Na_2S_2O_3$ (40 mL), dried ($MgSO_4$) and concentrated *in vacuo*. Purification by column chromatography (SiO_2, hexane→hexane:EtOAc, 9:1→4:1→1:1) gave title compound **126** (5.3 mg, 56%) as a pale yellow oil [$\boldsymbol{R}_f$ = 0.37 (hexane:EtOAc, 4:1)].[26]

The 5.3 mg of product was divided into 2 portions and processed using the following 3 step procedure. After this, the 2 batches were combined and purified by column chromatography. The yield (of **2***) quoted is based on the 5.3 mg isolated from the aforementioned glycosidation step.*

[†] The aglycon **4** and thioglycoside **115** were azeotroped with PhMe (× 3) prior to use.

Callipeltoside B (2)

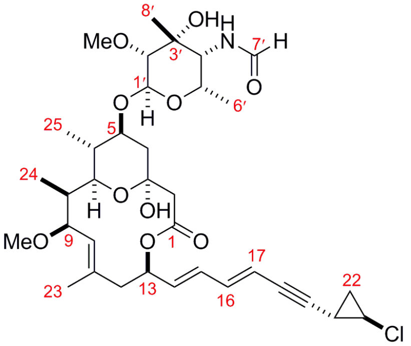

To a solution of **126** (2.2 mg, 2.9 μmol) in pyridine (95 μL) and H_2O (9.5 μL) (10:1) at RT was added Et_3N (5.9 μL) and 1,3-propanedithiol (5.9 μL, 58.7 μmol) sequentially. The reaction was stirred for 2 h 30 min and further Et_3N (5.9 μL) and 1,3-propanedithiol (5.9 μL, 58.7 μmol) added. After 90 min TLC analysis indicated complete consumption of the starting material. Azeotropic removal of the solvents with PhMe (3 × 10 mL) gave the requisite amine (assumed quant.) as an off-white residue. The isolated amine was used immediately in the next step of the reaction without further purification.

To a solution of crude amine (assumed quant., 2.9 μmol) in $CHCl_3$ (0.2 mL) was added freshly prepared **113**[24] (31.1 mg, 0.147 mmol). The reaction was stirred at RT for 30 min and additional **113** (31.1 mg, 0.147 mmol) was added and stirred for 30 min after which the mixture was concentrated *in vacuo*. Purification by column chromatography (SiO_2, hexane→hexane:EtOAc, 9:1→4:1→7:3→3:2→1:1→2:3→EtOAc) gave TMS-protected callipeltoside B (assumed quant.) as a clear oil [$\boldsymbol{R}_\mathbf{f}$ = 0.45 (hexane:EtOAc, 1:1)]. The isolated TMS-protected callipeltoside B was used immediately in the next step of the reaction without characterisation.

To a solution of TMS-protected callipeltoside B (assumed quant., 2.9 μmol) in DMF (0.5 mL) at RT was added TASF (4 mg, 14.5 μmol) and the mixture heated to 40 °C. After 30 min the reaction was cooled to RT, quenched by the addition of pH 7 phosphate buffer (1 mL) and diluted with EtOAc (2 mL). The layers were separated and the aqueous layer further extracted with EtOAc (3 × 5 mL). The combined organic layers were washed with 10% aq. LiCl (2 × 8 mL), dried ($MgSO_4$) and concentrated *in vacuo*. Purification by column chromatography (SiO_2,

hexane→hexane:EtOAc, 7:3→1:1→3:7→EtOAc→CH_2Cl_2:MeOH, 9:1) gave the title compound callipeltoside B **2** (2.5 mg, 52% over 3 steps) as an off-white gum consisting of 2 rotamers (4:1 by ^{1}H NMR).[30] The sample was further purified by HPLC (see below).

Rotameric ratio ascertained by ^{1}H NMR spectroscopy of the purified mixture; δ_H (**$CDCl_3$**, 500 MHz) 8.36 (1H, d, *J* = 1.4 Hz, NHC7′*H*O major), 7.93 (1H, d, *J* = 11.9 Hz, NHC7′*H*O minor).

R

$\boldsymbol{R_f}$ = 0.18 (minor), 0.14 (major) (EtOAc); $[\alpha]_D^{26.2}$ = –21.0 (*c* = 0.10, $CDCl_3$); **IR** (film) ν_{max}/cm^{-1} 3433, 2976, 2925, 2857, 1675, 1512, 1457, 1416, 1380, 1347, 1324, 1310, 1255, 1226, 1179, 1098, 1086, 1058, 1023, 980.

Callipeltoside B ^{1}H and ^{13}C NMR in $CDCl_3$

^{1}H NMR (**$CDCl_3$**, 500 MHz)† δ = 8.36 (1H, d, *J* = 1.4 Hz, NHC7′*H*O major rotamer), 7.93 (1H, d, *J* = 11.9 Hz, NHC7′*H*O minor rotamer), 6.48 (1H, dd, *J* = 15.4, 10.6 Hz, C16H), 6.31 (1H, d, *J* = 10.4 Hz, N*H*C7′HO major rotamer), 6.26 (1H, dd, *J* = 15.2, 10.9 Hz, C15H), 6.13 (1H, t, *J* = 11.4 Hz, N*H*C7′HO minor rotamer), 5.82 (1H, m, C13H), 5.76 (1H, dd, *J* = 15.2, 6.7 Hz, C14H), 5.57 (1H, dd, *J* = 15.5, 1.8 Hz, C17H), 5.29 (1H, d, *J* = 9.4 Hz, C10H), 5.03 (1H, d, *J* = 2.3 Hz, C3OH), 4.97 (1H, s, C1′H), 4.11 (1H, qd, *J* = 6.6, 1.4 Hz, C5′H major and minor rotamers), 3.97 (1H, d, *J* = 10.4 Hz, C4′H major rotamer), 3.81 (1H, dd, *J* = 9.4, 2.2 Hz, C9H), 3.71 (1H, td, *J* = 10.9, 4.5 Hz, C5H), 3.65 (1H, dd, *J* = 10.4, 2.3 Hz, C7H), 3.47 (3H, s, OCH_3 major rotamer), 3.43 (3H, s, OCH_3 minor rotamer), 3.28 (1H, d, *J* = 0.7 Hz, C3′OH minor rotamer), 3.23 (3H, s, C9H(OCH_3)), 3.18 (1H, m, C21H)*, 3.08 (1H, d, *J* = 0.8 Hz, C3′OH major rotamer), 2.97 (1H, br s, C2′H major rotamer), 2.96 (1H, br s, C2′H minor rotamer), 2.96 (1H, d overlapped, C4′H minor rotamer), 2.55 (1H, appar. dd, *J* = 12.9, 3.9 Hz, C2H_AH_B), 2.43 (1H, d, *J* = 13.0 Hz, C2H_AH_B), 2.30 (1H, appar. d, *J* = 3.7 Hz, C12H_AH_B), 2.29 (1H, m overlapped, C12H_AH_B)*, 2.21 (1H, dd, *J* = 11.5, 4.7 Hz, equatorial C4H_AH_B), 2.20 (1H, m overlapped, C8H), 1.80 (1H, m, C20H), 1.73 (3H, s, C23H_3), 1.47 (1H, m, C6H), 1.45 (3H, d, *J* = 0.7 Hz, C8′H_3 major rotamer), 1.43 (3H, d, *J* = 0.9 Hz, C8′H_3 minor rotamer), 1.36 (1H, td, *J* = 11.7, 2.5 Hz, axial C4H_AH_B),

† Only the rotameric peaks attributed to the sugar are distinguishable in the ^{1}H NMR.

1.29 (2H, m, C22H_AH_B)*, 1.14 (3H, d, *J* = 6.4 Hz, C6′H_3 minor rotamer), 1.12 (3H, d, *J* = 6.4 Hz, C6′H_3 major rotamer), 0.98 (3H, d, *J* = 7.0 Hz, C24H_3), 0.93 (3H, d, *J* = 6.5 Hz, C25H_3); **^{13}C NMR** (**$CDCl_3$**, 126 MHz) δ = 171.7 (C1O_2), 164.8 (NH*C*′7HO minor rotamer), 161.5 (NH*C*′7HO major rotamer), 140.1 (C16H), 132.6 (C14H), 132.4 (C11), 131.0 (C15H), 127.7 (C10H), 112.6 (C17H), 99.2 (C1′H major rotamer), 98.8 (C1′H minor rotamer), 95.2 (C3), 92.3 (C19), 83.4 (C2′H major rotamer), 82.7 (C2′H minor rotamer), 79.7 (C9H), 79.6 (C5H), 77.4 (C18), 74.8 (C7H), 71.6 (C13H), 68.0 (C3′ major rotamer), 68.0 (C3′ minor rotamer), 64.9 (C5′H major rotamer), 64.4 (C5′H minor rotamer), 61.0 (C4′H minor rotamer), 59.3 (OCH_3 major rotamer), 59.0 (OCH_3 minor rotamer), 55.5 (C4′H major rotamer), 55.2 (C9H(O*C*H_3)), 46.9 (C12H_2), 44.7 (C2H_2), 42.4 (C4H_2), 38.4 (C6H), 37.0 (C8H), 34.3 (C21H), 23.8 (C8′H_3 major rotamer), 23.5 (C8′H_3 minor rotamer), 19.3 (C22H_2), 17.7 (C6′H_3 minor rotamer), 17.3 (C6′H_3 major rotamer), 16.1 (C23H_3), 12.4 (C20H), 12.1 (C25H_3), 6.5 (C24H_3).

Callipeltoside B ^{1}H and ^{13}C NMR in CD_3OD

^{1}H NMR (**CD_3OD**, 500 MHz) δ = 8.24 (1H, s, NHC7′*H*O major rotamer), 7.90 (1H, s, NHC7′*H*O minor rotamer), 6.53 (1H, dd, *J* = 15.4, 10.9 Hz, C16H), 6.36 (1H, dd, *J* = 14.3, 11.0 Hz, C15H), 5.86 (1H, m overlapped, C14H), 5.84 (1H, m overlapped, C13H), 5.67 (1H, dd, *J* = 15.4, 1.9 Hz, C17H), 5.30 (1H, d, *J* = 9.7 Hz, C10H), 5.0 (1H, d, *J* = 1.1 Hz, C1′H), 4.16 (1H, qd, *J* = 6.5, 1.7 Hz, C5′H), 3.90 (1H, dd, *J* = 9.7, 2.4 Hz, C9H), 3.86 (1H, s, C4′H), 3.73 (1H, td, *J* = 10.6, 4.6 Hz, C5H), 3.68 (1H, dd, *J* = 10.4, 2.5 Hz, C7H), 3.49 (3H, s, OCH_3 major rotamer), 3.49 (3H, s, OCH_3 minor rotamer), 3.27 (1H, m, C21H), 3.24 (3H, s, C9H(OCH_3)), 3.04 (1H, t, *J* = 1.1 Hz, C2′H minor rotamer), 3.01 (1H, t, *J* = 1.1 Hz, C2′H major rotamer), 2.58 (1H, d, *J* = 13.0 Hz, C2H_AH_B), 2.50 (1H, d, *J* = 12.9 Hz, C2H_AH_B), 2.37 (1H, m overlapped, C12H_AH_B), 2.30 (1H, m overlapped, C12H_AH_B), 2.24 (1H, m overlapped, C8H), 2.24 (1H, m overlapped, equatorial C4H_AH_B), 1.83 (1H, m, C20H), 1.77 (3H, s, C23H_3), 1.51 (1H, m, C6H), 1.46 (1H, t, *J* = 11.4 Hz, axial C4H_AH_B), 1.44 (3H, s, C8′H_3), 1.30 (2H, m, C22H_AH_B), 1.13 (3H, d, *J* = 6.3 Hz, C6′H_3 minor rotamer), 1.10 (3H, d, *J* = 6.4 Hz, C6′H_3 major rotamer), 0.99 (6H, appar. d, *J* = 6.5 Hz, C24H_3 and C25H_3)†; **^{13}C NMR** (**CD_3OD**, 126 MHz) δ = 172.9 (C1O_2), 164.6 (NH*C*′7HO), 141.6 (C16H), 134.4 (C11), 134.2 (C14H), 132.1 (C15H), 128.3 (C10H), 113.5 (C17H), 101.2 (C1′H), 96.6 (C3), 92.8 (C19), 84.5 (C2′H), 81.3 (C9H), 80.8 (C5H), 78.4

(C18), 76.3 (C7H), 72.7 (C13H), 69.7 (C3′), 66.2 (C5′H), 59.8 (OCH_3), 57.1 (C4′H), 55.4 (C9H(OCH_3)), 47.8 (C12H_2), 45.9 (C2H_2), 43.8 (C4H_2), 39.8 (C6H), 38.3 (C8H), 35.1 (C21H), 24.8 (C8′H_3), 19.8 (C22H_2), 17.6 (C6′H_3), 16.3 (C23H_3), 12.8 (C20H), 12.7 (C25H_3), 6.8 (C24H_3); **HRMS** (+ESI) Found $[M+Na]^+$ = 702.3018; $C_{35}H_{50}O_{10}NClNa$ requires 702.3015, Δ 0.30 ppm.†

* Protons and carbons reassigned based on 2D COSY, HSQC and HMBC data recorded for the synthetic sample.

† Following a personal communication with Professor Zampella, a ^{13}C NMR (CD_3OD) derived from the HSQC/HMBC was provided. Hence, the data was also recorded in CD_3OD and found to match the data provided for the natural isolate.

^{1}H NMR comparison between synthetic and natural callipeltoside B ($CDCl_3$)

	**Natural callipeltoside B[30]**	**Synthetic callipeltoside B**
**Atom**	**δ in ppm (500 MHz)**	**δ in ppm (500 MHz)**
1		
2	2.53 (d, 13.5), 2.43 (d, 13.5)	2.55 (appar. dd, 12.9, 3.9), 2.43 (d, 13.0)
3		
4	2.21 (dd, 11.2, 4.4), 1.31 (dt, 11.2, 2.0)	2.21 (dd, 11.5, 4.7), 1.36 (td, 11.7, 2.5)
5	3.71 (dt, 11.7, 4.4)	3.71 (td, 10.9, 4.5)
6	1.46 (m)	1.47 (m)
7	3.64 (dd, 10.5, 2.0)	3.65 (dd, 10.4, 2.3)
8	2.20 (m)	2.20 (m, overlapped)
9	3.81 (dd, 9.1, 1.7)	3.81 (dd, 9.4, 2.2)
10	5.29 (br dd, 9.1)	5.29 (d, 9.4)
11		
12	2.31 (dd, overlapped)<br>2.09 (dd, overlapped)]*	2.30 (appar. d, 3.7)<br>2.29 (m, overlapped)
13	5.82 (m)	5.82 (m)
14	5.76 (dd, 15.3, 7.1)	5.76 (dd, 15.2, 6.7)
15	6.26 (dd, 15.3, 10.8)	6.26 (dd, 15.2, 10.9)
16	6.48 (dd, 15.7, 10.8)	6.48 (15.4, 10.6)
17	5.57 (dd, 15.7, 1.7)	5.57 (dd, 15.5, 1.8)
18		
19		
20	1.80 (m, overlapped)	1.80 (m)
21	[1.29 (m)]*	3.18 (m)
22	[3.18 (m)]*	1.29 (m)
23	1.73 (s)	1.73 (s)
24	0.98 (d, 6.9)	0.98 (d, 7.0)
25	0.94 (d, 6.4)	0.93 (d, 6.5)
C9H(OC*H*$_3$)	3.23 (s)	3.23 (s)
3-OH	5.03 (d, 2.0)	5.03 (d, 2.3)
1′	4.97 (br s)	4.97 (s)
2′	2.97 (br s)	2.97 (br s), 2.96 (br s)
3′		
4′	3.97 (d, 10.5)	3.97 (d, 10.4), 2.96 (d, overlapped)
5′	4.11 (q, 6.5)	4.11 (qd, 6.6, 1.4)
6′	[1.20 (d, 6.5), 1.22 (d, 6.5)]†	1.14 (d, 6.4), 1.12 (d, 6.4)
7′	8.36 (br s), 7.92 (d, 10.5)	8.36 (d, 1.4), 7.93 (d, 11.9)
8′	1.44 (s), 1.42 (s)	1.45 (d, 0.7), 1.43 (d, 0.9)
OCH_3	3.47 (s), 3.43 (s)	3.47 (s), 3.43 (s)
NH	6.32† (d, 10.5), 6.13 (t, 10.5)	7.93 (d, 11.9), 6.13 (t, 11.4)
C3′-OH		3.28 (d, 0.7), 3.08 (d, 0.8)

† The tabulated data differs from the spectrum supplied by Professor Zampella. We attribute this to a typographical error.

A further NMR experiment ($CDCl_3$) was conducted in order to elucidate the configuration at the anomeric carbon. Measurement of the $^{1}J_{C\text{-}H}$ value from a HSQC (Heteronuclear Single Quantum Coherence) experiment without ^{13}C decoupling is known to be diagnostic of the configuration of the glycosidic linkage. A value of ~170 Hz suggests an equatorial proton at C1′H, whilst ~160 Hz indicates an axial proton. The measurement gave $\mathbf{^{1}J_{C\text{-}H} = 165.6}$ **Hz**, giving an inconclusive result.[29]

Key selected observed nOe's from NOESY ($CDCl_3$)

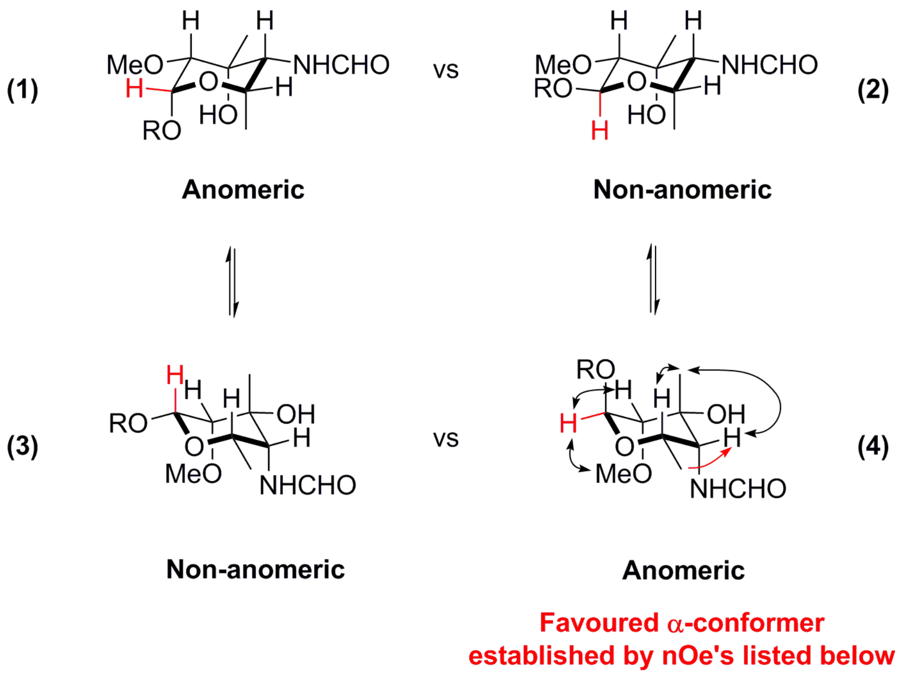

- An nOe was observed between C1′H and C2′H suggesting that **conformer 2** is **disfavoured**.
- An nOe was observed between C1′H and OMe suggesting that **conformer 3** is **disfavoured**.
- An nOe between C6′H_3 and C4′H suggesting that **conformers 1** and **2** are **disfavoured**.
- **α-conformer 4** accounts for all observed nOe interactions indicating that **C1′H** is **equatorial**.

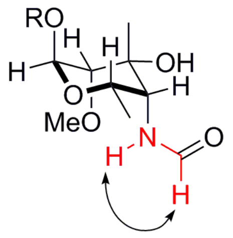

cis-rotamer

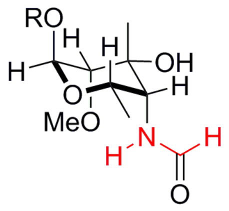

trans-rotamer

- An nOe ($CDCl_3$) was observed between the formyl and NH protons of the **major rotamer** suggesting that the predominant form is ***cis***.
- As expected, **no nOe** ($CDCl_3$) between the formyl and NH protons was observed for the ***trans*** (minor) rotamer.

HPLC conditions

Preparative HPLC purification was performed on an Agilent HP 1100 series chromatograph equipped with a Waters µBondapak C18 column (column length 150 mm, internal diameter of column 3.9 mm, particle size 10 µm, temperature 25 °C). Elution was carried out at a flow rate of 2.0 mL/min using MeOH:H_2O (80:20) and detection was with diode array detection (λ= 250, 272 and 286). The sample was made up to a concentration of 0.003 mg/µL with purification carried out using 30 µL injections which saturated the detector at λ= 250, 272 and 286. t_R = 3.6 min.

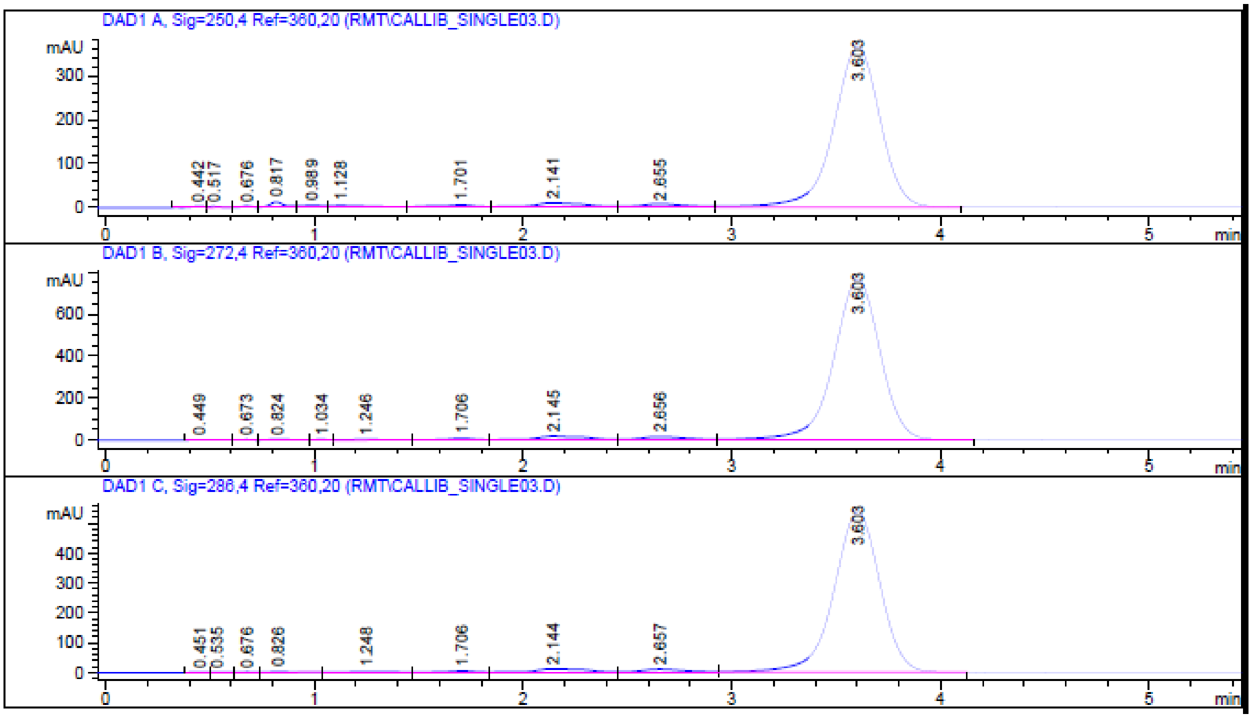

(1*S*,5*R*,7*E*,9*R*,10*R*,11*R*,12*R*,13*S*)-5-{(1*E*,3*E*)-6-[(1*S*,2*R*)-2-chlorocyclopropyl]hexa-1,3-dien-5-yn-1-yl}-1-hydroxy-9-methoxy-7,10,12-trimethyl-3-oxo-4,15-dioxabicyclo[9.3.1]pentadec-7-en-13-yl 4-azido-4,6-dideoxy-3-*C*-methyl-2-*O*-methyl-3-*O*-(trimethylsilyl)-α-D-talopyranoside 127

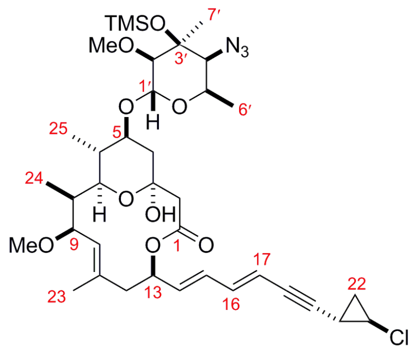

To a solution of aglycon **4** (4.5 mg, 9.4 μmol) and thioglycoside *ent*-**115** (7.2 mg, 18.8 μmol) in CH_2Cl_2 (2.4 mL) at RT was added 4 Å MS followed by DTBMP (4.9 mg, 23.9 μmol) and the mixture stirred for 50 min.† The reaction was cooled to –15 °C and NIS (4.2 mg, 18.8 μmol) and TfOH [(32 μL, 3.6 μmol) of a stock solution of TfOH in CH_2Cl_2 (0.1 mL, 1.125 mmol in 10 mL CH_2Cl_2)] added sequentially. The resulting light pink solution was allowed to warm to RT slowly over 16 h. The reaction was quenched by the addition of sat. aq. $NaHCO_3$ (6 mL) and diluted with EtOAc (10 mL). The layers were separated and the aqueous layer further extracted with EtOAc (3 × 15 mL). The combined organic layers were washed with sat. aq. $Na_2S_2O_3$ (30 mL), dried ($MgSO_4$) and concentrated *in vacuo*. Purification by column chromatography (SiO_2, hexane→hexane:EtOAc, 9:1→4:1→1:1) gave title compound **127** (2.9 mg, 41%) as a pale yellow oil [$\boldsymbol{R}_f$ = 0.39 (hexane:EtOAc, 4:1)].[26]

† The aglycon **4** and thioglycoside *ent*-**115** were azeotroped with PhMe (× 3) prior to use.

Callipeltoside B diastereomer 128

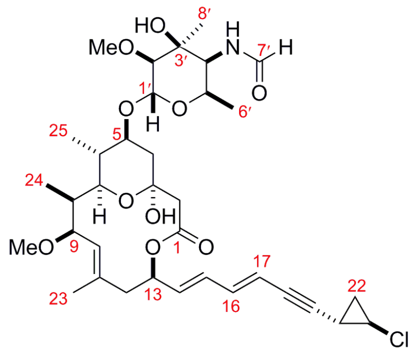

To a solution of **127** (2.9 mg, 3.9 μmol) in pyridine (95 μL) and H_2O (9.5 μL) (10:1) at RT was added Et_3N (7.8 μL) and 1,3-propanedithiol (7.8 μL, 77.0 μmol) sequentially. The reaction was stirred for 2 h 30 min and further Et_3N (7.8 μL) and 1,3-propanedithiol (7.8 μL, 77.0 μmol) added. After 90 min TLC analysis indicated complete consumption of the starting material. Azeotropic removal of the solvents with PhMe (3 × 10 mL) gave the requisite amine (assumed quant.) as an off-white residue. The isolated amine was used immediately in the next step of the reaction without further purification.

To a solution of crude amine (assumed quant. 3.9 μmol) in $CHCl_3$ (0.2 mL) was added freshly prepared **113**[24] (164 mg, 0.77 mmol). The reaction was stirred at RT for 1 h after which the mixture was concentrated *in vacuo*. Purification by column chromatography (SiO_2, hexane→hexane:EtOAc, 9:1→4:1→7:3→3:2→1:1→2:3→EtOAc) gave TMS-protected callipeltoside B (assumed quant.) as a clear oil [$\boldsymbol{R}_\mathbf{f}$ = 0.34 (hexane:EtOAc, 1:1)]. The isolated TMS-protected callipeltoside B was used immediately in the next step of the reaction without characterisation.

To a solution of TMS-protected callipeltoside B (assumed quant., 3.9 μmol) in DMF (0.5 mL) at RT was added TASF (5.3 mg, 19.5 μmol) and the mixture heated to 40 °C. After 30 min the reaction was cooled to RT, quenched by the addition of pH 7 phosphate buffer (1 mL) and diluted with EtOAc (2 mL). The layers were separated and the aqueous layer further extracted with EtOAc (3 × 5 mL). The combined organic layers were washed with 10% aq. LiCl (2 × 8 mL), dried ($MgSO_4$) and concentrated *in vacuo*. Purification by column chromatography (SiO_2,

hexane→hexane:EtOAc, 7:3→1:1→3:7→EtOAc→CH_2Cl_2:MeOH, 9:1) gave the title compound **128** (1.5 mg, 57% over 3 steps) as an off-white gum consisting of 2 rotamers (4:1 by ^{1}H NMR). The sample was further purified by HPLC (see below).

Rotameric ratio ascertained by ^{1}H NMR spectroscopy of the purified mixture; δ_H ($CDCl_3$, 500 MHz) 8.36 (1H, d, *J* = 1.4 Hz, NHC7′*H*O major), 7.93 (1H, d, *J* = 11.9 Hz, NHC7′*H*O minor).

***R*$_f$** = 0.18 (minor), 0.14 (major) (EtOAc); $[\alpha]_D^{27.8}$ = +3.0 (*c* = 0.10, $CDCl_3$); **IR** (film) ν_{max}/cm^{-1} 3404, 2962, 2917, 2849, 1693, 1512, 1463, 1441, 1415, 1386, 1346, 1325, 1310, 1260, 1228, 1174, 1153, 1094, 1084, 1045, 1018, 983; **^{1}H NMR** ($CDCl_3$, 500 MHz)† δ = 8.37 (1H, d, *J* = 1.4 Hz, NHC7′*H*O major rotamer), 7.94 (1H, d, *J* = 11.7 Hz, NHC7′*H*O minor rotamer), 6.48 (1H, dd, *J* = 15.8, 10.9 Hz, C16H), 6.30 (1H, d, *J* = 9.5 Hz, N*H*C7′HO major rotamer), 6.27 (1H, dd, *J* = 15.5, 11.0 Hz, C15H), 6.14 (1H, t, *J* = 11.7 Hz, N*H*C7′HO minor rotamer), 5.83 (1H, m, C13H), 5.76 (1H, dd, *J* = 15.2, 6.4 Hz, C14H), 5.58 (1H, d, *J* = 14.0 Hz, C17H), 5.30 (1H, d, *J* = 8.3 Hz, C10H), 5.06 (1H, dd, *J* = 7.0, 2.0 Hz, C3OH), 5.01 (1H, s, C1′H), 4.16 (1H, m, C5′H major and minor rotamers), 4.00 (1H, d, *J* = 10.5 Hz, C4′H major rotamer), 3.85 (1H, td, *J* = 10.7, 4.5 Hz, C5H), 3.81 (1H, dd, *J* = 9.7, 2.2 Hz, C9H), 3.66 (1H, dd, *J* = 10.4, 2.2 Hz, C7H), 3.47 (3H, s, OCH_3 major rotamer), 3.43 (3H, s, OCH_3 minor rotamer), 3.29 (1H, s, C3′OH minor rotamer), 3.24 (3H, s, C9H(OCH_3)), 3.18 (1H, m, C21H), 3.09 (1H, s, C3′OH major rotamer), 2.99 (1H, d, *J* = 10.5 Hz, C4′H minor rotamer), 2.89 (1H, s, C2′H major rotamer), 2.88 (1H, s, C2′H minor rotamer), 2.54 (1H, d, *J* = 12.9 Hz, C2H_AH$_B$) 2.44 (1H, appar. dd, *J* = 12.9, 2.8 Hz, C2H$_A$$H_B$), 2.31 (1H, appar. d, *J* = 4.3 Hz, C12H_AH$_B$), 2.30 (1H, m overlapped, C12H$_A$$H_B$), 2.24 (1H, m, C8H), 2.19 (1H, dd, *J* = 11.7, 4.4 Hz, equatorial C4H_AH$_B$), 1.80 (1H, m, C20H), 1.73 (3H, s, C23H_3), 1.54 (1H, m overlapped, C6H), 1.47 (3H, s, C8′H_3 major rotamer), 1.45 (3H, s, C8′H_3 minor rotamer), 1.40 (1H, m overlapped, axial C4H$_A$$H_B$), 1.30 (2H, m, C22H$_AH_B$), 1.17 (3H, d, *J* = 6.4 Hz, C6′H_3 minor rotamer), 1.14 (3H, d, *J* = 6.4 Hz, C6′H_3 major rotamer), 0.99 (3H, d, *J* = 7.0 Hz, C24H_3), 0.96 (3H, d, *J* = 6.4 Hz, C25H_3); **^{13}C NMR** ($CDCl_3$, 126 MHz)† δ =

† Only the rotameric peaks attributed to the sugar are distinguishable in the ^{1}H NMR.

† The ^{13}C NMR peaks corresponding to the minor rotamer could not be observed due to the small amount of sample isolated.

171.7 ($C1O_2$), 161.5 (NH*C′*7HO), 140.1 (C16H), 132.5 (C14H), 132.4 (C11), 131.0 (C15H), 127.7 (C10H), 112.6 (C17H), 95.2 (C3), 93.7 (C1′H), 92.4 (C19), 83.8 (C2′H), 79.7 (C9H), 77.4 (C18 overlapped), 75.0 (C7H), 74.7 (C5H), 71.7 (C13H), 68.1 (C3′), 65.2 (C5′H), 59.4 (OCH_3), 55.5 (C4′H), 55.2 (C9H(O*C*H_3)), 46.9 ($C12H_2$), 44.8 ($C2H_2$), 39.6 ($C4H_2$), 37.7 (C6H), 37.0 (C8H), 34.3 (C21H), 23.7 ($C8'H_3$), 19.3 ($C22H_2$), 17.3 ($C6'H_3$), 16.1 ($C23H_3$), 12.7 (C20H), 12.1 ($C25H_3$), 6.5 ($C24H_3$); **HRMS** (+ESI) Found $[M+Na]^+$ = 702.3004; $C_{35}H_{50}O_{10}NClNa$ requires 702.3015, Δ 1.58 ppm.

A further NMR experiment was conducted in order to elucidate the configuration at the anomeric carbon. Measurement of the $^{1}J_{C\text{-}H}$ value from a HSQC (Heteronuclear Single Quantum Coherence) experiment without ^{13}C decoupling is known to be diagnostic of the configuration of the glycosidic linkage. A value of ~170 Hz suggests an equatorial proton at C1′H, whilst ~160 Hz indicates an axial proton. The measurement gave $\mathbf{^{1}J_{C\text{-}H} = 166.3\ Hz}$, giving an inconclusive result.[29]

Key selected observed nOe's from NOESY

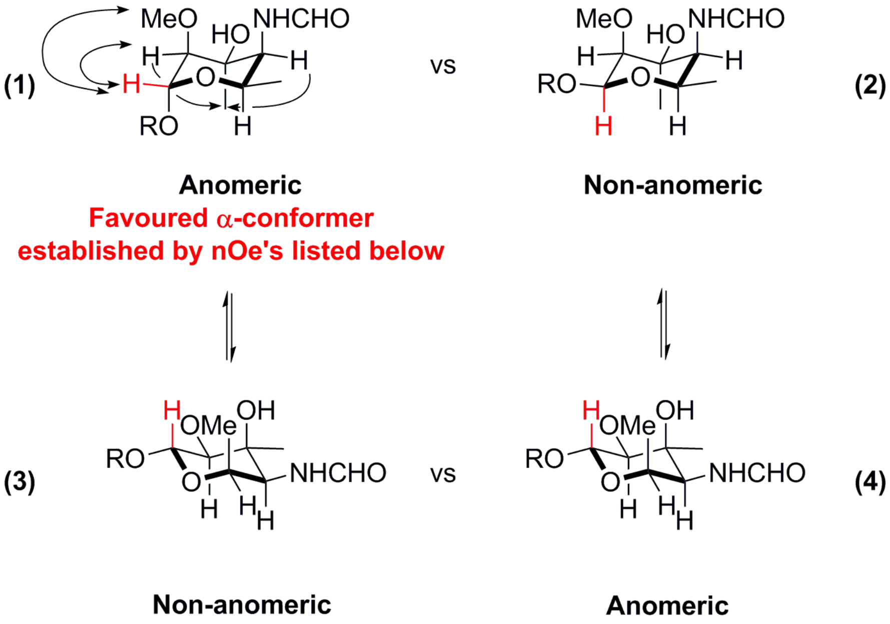

- An nOe was observed between C1′H and C2′H suggesting that **conformers 3** and **4** are **disfavoured**.
- An nOe was observed between C1′H and OMe suggesting that **conformer 2** is **disfavoured**.
- **α-conformer 1** accounts for all observed nOe interactions indicating that **C1′H** is **equatorial**.

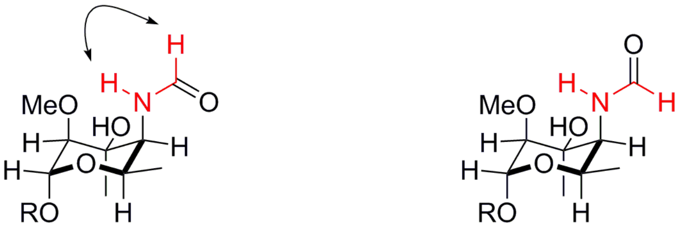

cis-rotamer   *trans*-rotamer

- As in the case for the natural product, an nOe was observed between the formyl and NH protons of the **major rotamer** suggesting that the predominant form is ***cis***.
- As expected, **no nOe** between the formyl and NH protons was observed for the ***trans*** (minor) rotamer.

HPLC conditions

Preparative HPLC purification was performed on an Agilent HP 1100 series chromatograph equipped with a Waters µBondapak C18 column (column length 150 mm, internal diameter of column 3.9 mm, particle size 10 µm, temperature 25 °C). Elution was carried out at a flow rate of 2.0 mL/min using MeOH:H_2O (80:20) and detection was with diode array detection (λ= 250, 272 and 286). The sample was made up to a concentration of 0.003 mg/µL with purification carried out using 20 µL injections which saturated the detector at λ= 250, 272 and 286. t_R = 3.0 min.

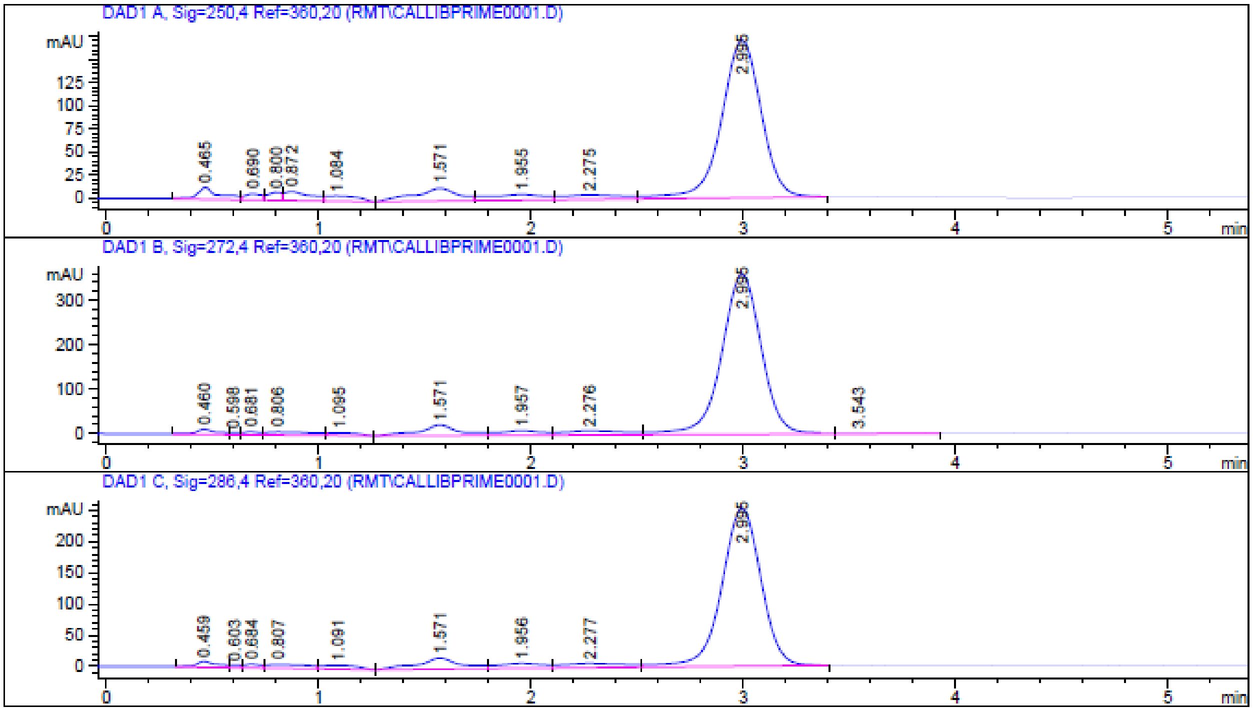

Callipeltoside C (3)

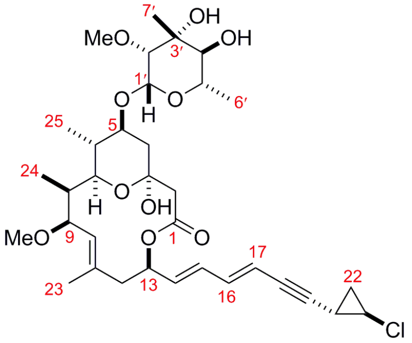

To a solution of aglycon **4** (6.4 mg, 13.4 µmol) and thioglycoside **123** (12.6 mg, 26.7 µmol) in CH_2Cl_2 (3.4 mL) at RT was added 4 Å MS followed by DTBMP (7.0 mg, 34 µmol) and the mixture stirred for 50 min.† The reaction was cooled to –15 °C and NIS (6.0 mg, 26.7 µmol) and TfOH [(45 µL, 5.1 µmol) of a stock solution of TfOH in CH_2Cl_2 (0.1 mL, 1.125 mmol in 10 mL CH_2Cl_2)] added sequentially resulting in the formation of a light pink colour. The reaction was allowed to warm to RT slowly over 16 h. The reaction was quenched by the addition of sat. aq. $NaHCO_3$ (10 mL) and diluted with EtOAc (15 mL). The layers were separated and the aqueous layer further extracted with EtOAc (3 × 15 mL). The combined organic layers were washed with sat. aq. $Na_2S_2O_3$ (30 mL), dried ($MgSO_4$) and concentrated *in vacuo*. Purification by column chromatography (SiO_2, hexane→hexane:EtOAc, 9:1→4:1→1:1) gave *bis*-protected callipeltoside C (assumed quant.) as a pale yellow oil/foam [$\boldsymbol{R_f}$ = 0.37 (hexane:EtOAc, 4:1)].[26]

To a solution of *bis*-protected callipeltoside C (assumed quant., 13.4 µmol) in DMF (1.8 mL) at RT was added TASF (23.3 mg, 85 µmol) and the mixture heated to 40 °C. After 1 h, further TASF (14.6 mg, 53 µmol) was added, and the reaction maintained at 40 °C for an additional 1 h. The reaction was cooled to RT, quenched by the addition of pH 7 phosphate buffer (10 mL) and diluted with EtOAc (10 mL). The layers were separated and the aqueous layer further extracted with EtOAc (3 × 15 mL). The combined organic layers were washed with 10% aq. LiCl (2 × 15 mL), dried ($MgSO_4$) and concentrated *in vacuo*. Purification by column chromatography (SiO_2, hexane→hexane:EtOAc, 4:1→1:1→EtOAc) gave the title compound callipeltoside C **3**

† The aglycon **4** and thioglycoside **123** were azeotroped with PhMe (× 3) prior to use.

(5.0 mg, 57% over 2 steps) as an off-white gum.[30] The sample was further purified using HPLC (see below).

R$_{\mathbf{f}}$ = 0.13 (hexane:EtOAc, 1:1); $[\alpha]_D^{25.0}$ = –32.0 (*c* = 0.30, $CDCl_3$), [lit.[7] $[\alpha]_D^{23}$ = –23.1 (*c* = 0.18, $CDCl_3$)]; **IR** (film) ν_{max}/cm^{-1} 3360, 3187, 2923, 2852, 1703, 1660, 1632, 1468, 1423, 1411, 1378, 1342, 1319, 1260, 1226, 1180, 1154, 1136, 1086, 1052, 1023, 979; **^{1}H NMR** ($CDCl_3$, 500 MHz) δ = 6.48 (1H, dd, *J* = 15.4, 10.9 Hz, C16H), 6.27 (1H,dd, *J* = 15.1, 10.9 Hz, C15H), 5.82 (1H, m, C13H), 5.76 (1H, dd, *J* = 15.1, 6.4 Hz, C14H), 5.58 (1H, dd, *J* = 15.3, 1.7 Hz, C17H), 5.30 (1H, d, *J* = 9.2 Hz, C10H), 4.99 (1H, d, *J* = 2.3 Hz, C3OH), 4.94 (1H, s, C1′H), 3.81 (1H, dd, *J* = 9.5, 2.4 Hz, C9H), 3.70 (1H, td, *J* = 10.3, 4.5 Hz, C5H), 3.65 (1H, m overlapped, C7H), 3.65 (1H, dq overlapped, C5′H), 3.47 (3H, s, OCH_3), 3.36 (1H, dd, *J* = 9.6, 2.3 Hz, C4′H), 3.23 (3H, s, C9H(OCH_3)), 3.18 (1H, m, C21H)*, 3.12 (1H, d, *J* = 1.0 Hz, C2′H), 2.88 (1H, s, C3′OH)*, 2.52 (1H, d, *J* = 12.9 Hz, C2$H_A$$H_B$), 2.43 (1H, d, *J* = 12.9 Hz, C2$H_A$$H_B$), 2.30 (1H, appar. d, *J* = 4.0 Hz, C12$H_A$$H_B$), 2.28 (1H, m overlapped, C12$H_A$$H_B$)*, 2.25 (1H, m overlapped, equatorial C4$H_A$$H_B$), 2.20 (1H, m overlapped, C8H)*, 2.03 (1H, s, C4′HO*H*)*, 1.80 (1H, m, C20H), 1.74 (3H, d, *J* = 0.9 Hz, C23H_3), 1.47 (1H, m, C6H), 1.35 (1H, td, *J* = 11.2, 2.0 Hz, axial C4$H_A$$H_B$), 1.31 (3H, s, C7′$H_3$), 1.29 (2H, m, C22$H_A$$H_B$)*, 1.26 (3H, d, *J* = 6.1 Hz, C6′H_3), 0.98 (3H, d, *J* = 7.0 Hz, C24H_3), 0.92 (3H, d, *J* = 6.5 Hz, C25H_3); **^{13}C NMR** ($CDCl_3$, 126 MHz) δ = 171.8 (C1O_2), 140.1 (C16H), 132.7 (C14H), 132.4 (C11), 130.9 (C15H), 127.7 (C10H), 112.5 (C17H), 98.7 (C1′H), 95.2 (C3), 92.3 (C19), 84.7 (C2′H), 79.7 (C9H), 79.1 (C5H), 77.5 (C18), 76.7 (C4′H), 74.8 (C7H), 72.3 (C3′), 71.5 (C13H), 67.0 (C5′H), 59.0 (OCH_3), 55.2 (C9H(OCH_3)), 46.9 (C12H_2), 44.7 (C2H_2), 42.4 (C4H_2), 38.5 (C6H), 37.0 (C8H), 34.3 (C21H)*, 19.3 (C22H_2), 17.8 (C6′H_3), 17.8 (C7′H_3), 16.1 (C23H_3), 12.4 (C20H)*, 12.1 (C25H_3), 6.5 (C24H_3); **HRMS** (+ESI) Found $[M+Na]^+$ = 675.2904; $C_{34}H_{49}O_{10}ClNa$ requires 675.2906, Δ 0.35 ppm.[†]

* Protons and carbons reassigned based on 2D COSY, HSQC and HMBC data recorded for the synthetic sample.

[†] The data is assigned in the same style as originally reported for the natural product.

^{1}H NMR comparison between synthetic, natural and previously synthesised callipeltoside C ($CDCl_3$)

	**Natural callipeltoside C[30]**	**MacMillan callipeltoside C[7]**	**Synthetic callipeltoside C**
**Atom**	**δ in ppm (500 MHz)**	**δ in ppm (500 MHz)**	**δ in ppm (500 MHz)**
1			
2	2.53 (d, 12.8)<br>2.43 (d, 12.8)	2.52 (d, 12.5)<br>2.43 (d, 12.5)	2.52 (d, 12.9)<br>2.43 (d, 12.9)
3			
4	2.25 (dd, 11.2, 4.4),<br>1.36 (dt, 11.2, 1.30)	2.25 (dd, 11.0, 5.0),<br>1.36 (dt, 11.0, 1.30)	2.25 (m, overlapped)<br>1.35 (td, 11.2, 2.0)
5	3.70 (dt, 10.2, 4.4)	3.70 (dt, 10.0, 4.5)	3.70 (td, 10.3, 4.5)
6	1.50 (m)	1.47 (m)	1.47 (m)
7	3.65 (dd, 10.5, 2.0)	3.65 (dd, 10.0, 2.5)	3.65 (m, overlapped)
8	[2.29 (m)]*	[2.29 (m)]*	2.20 (m, overlapped)
9	3.81 (dd, 9.4, 2.0)	3.81 (dd, 9.5, 2.5)	3.81 (dd, 9.5, 2.4)
10	5.30 (br dd, 9.4)	5.30 (appar. d, 9.0)	5.30 (d, 9.2)
11			
12	2.30 (dd, overlapped)<br>[2.09 (dd, overlapped)]*	2.30 (appar. d, 4.0)<br>[2.02 (m)]*	2.30 (appar. d, 4.0)<br>2.28 (m, overlapped)
13	5.82 (m)	5.82 (m)	5.82 (m)
14	5.77 (dd, 14.8, 6.6)	5.76 (dd, 15.0, 6.5)	5.76 (dd, 15.1, 6.4)
15	6.27 (dd, 14.8, 10.6)	6.27 (dd, 15.0, 11.0)	6.27 (dd, 15.1, 10.9)
16	6.48 (dd, 15.0, 10.6)	6.48 (dd, 15.5, 11.0)	6.48 (dd, 15.4, 10.9)
17	5.58 (dd, 15.0, 1.7)	5.58 (dd, 15.5, 1.5)	5.58 (dd, 15.3, 1.7)
18			
19			
20	1.80 (m,overlapped)	1.79 (m)	1.80 (m)
21	[1.29 (m, overlapped)]*	[1.29 (m)]*	3.18 (m)
22	[3.18 (m)]*	[3.18 (ddd, 5.5, 3.5, 2.0)]*	1.29 (m)
23	1.74 (s)	1.74 (d, 1.0)	1.74 (d, 0.9)
24	0.98 (d, 6.9)	0.98 (d, 7.0)	0.98 (d, 7.0)
25	0.93 (d, 6.4)	0.92 (d, 6.5)	0.92 (d, 6.5)
C9H(OCH_3)	3.23 (s)	3.23 (s)	3.23 (s)
3-OH	4.99 (d, 1.30)	5.00 (d, 2.5)	4.99 (d, 2.3)
1′	4.94 (br s)	4.94 (s)	4.94 (s)
2′	3.12 (br s)	3.12 (d, 1.5)	3.12 (d, 1.0)
3′			
4′	3.36 (dd, 10.3, 1.20)	3.36 (br d, 9.5)	3.36 (dd, 9.6, 2.3)
5′	3.65 (dq, overlapped)	3.65 (dq, overlapped)	3.65 (dq, overlapped)
6′	1.25 (d, 6.5)	1.26 (d, 6.0)	1.26 (d, 6.1)
7′	1.31 (s)	1.31 (s)	1.31 (s)
OCH_3	3.47 (s)	3.47 (s)	3.47 (s)
3′-OH	[3.52 (d, 1.20)]*	[3.49 (s)]*	2.88 (s)
4′-OH	[2.88 (s)]*	[2.89 (br s)]*	2.03 (s)

^{13}C NMR comparison between synthetic, natural and previously synthesised callipeltoside C ($CDCl_3$)

	Natural callipeltoside C[30†]	MacMillan callipeltoside C[7]	Synthetic callipeltoside C
**Atom**	**δ in ppm (500 MHz)**	**δ in ppm (500 MHz)**	**δ in ppm (500 MHz)**
1		171.8	171.8
2	46.0	44.7	44.7
3		95.2	95.2
4	43.8	42.4	42.4
5	79.0	79.1	79.1
6	39.8	38.5	38.5
7	74.8	74.8	74.8
8	38.1	37.0	37.0
9	79.5	79.7	79.7
10	128.0	127.7	127.7
11		132.7	132.4
12	46.7	46.9	46.9
13	71.4	71.5	71.5
14	132.5	132.5	132.7
15	130.6	130.0	130.9
16	140.0	140.2	140.1
17	112.1	112.5	112.5
18		77.5	77.5
19		92.3	92.3
20	(34.0)*	12.4	12.4
21	(19.0)*	34.2	34.3
22	(55.4)*	19.3	19.3
23	15.7	14.2	16.1
24	6.1	6.5	6.5
25	(12.0)*	12.1	12.1
C9H(OCH_3)	55.2	55.2	55.2
3-OH			
1′	98.9	98.7	98.7
2′	84.7	84.7	84.7
3′		72.3	72.3
4′	76.7	76.7	76.7
5′	66.7	67.0	67.0
6′	17.5	17.8	17.8
7′	(17.4)*	16.2	17.8
OCH_3	62.0	60.4	59.0
3′-OH			
4′-OH			

[†] Due to the small sample of natural product, the carbon signals were detected in the HMQC spectrum.

*Protons and carbons reassigned based on COSY, HSQC and HMBC data recorded for the synthetic sample.

A further NMR experiment was conducted in order to elucidate the configuration at the anomeric carbon. Measurement of the $^{1}J_{C\text{-}H}$ value from a HSQC (Heteronuclear Single Quantum Coherence) experiment without ^{13}C decoupling is known to be diagnostic of the configuration of the glycosidic linkage. A value of ~170 Hz suggests an equatorial proton at C1′H, whilst ~160 Hz indicates an axial proton. The measurement gave $\mathbf{^{1}J_{C\text{-}H}}$ **= 166.5 Hz**, giving an inconclusive result.[29]

Key selected observed nOe's from NOESY

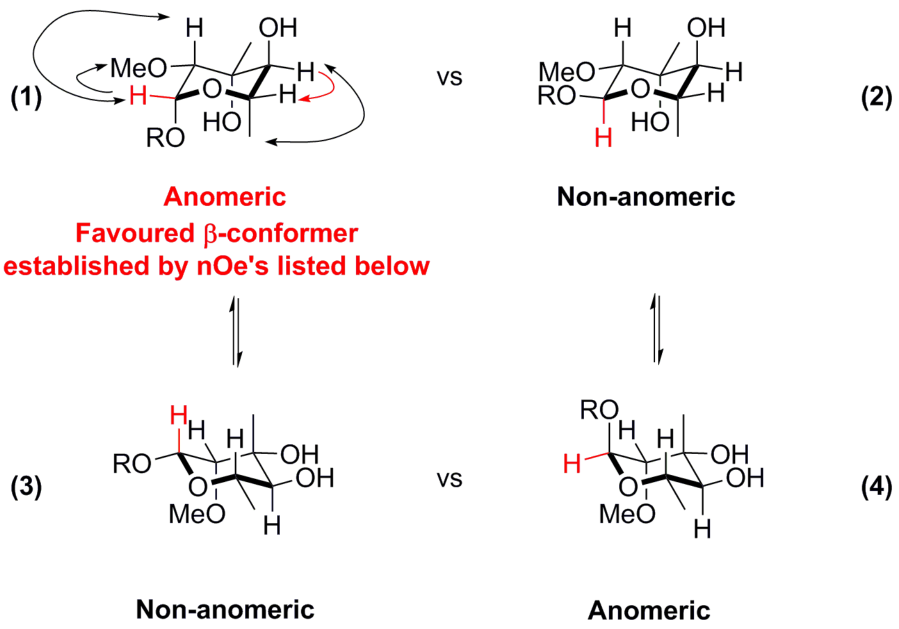

- An nOe was observed between C1′H and C2′H suggesting that **conformer 2** is **disfavoured**.
- An nOe was observed between C1′H and OMe suggesting that **conformer 3** is **disfavoured**.
- An nOe between C4′H and C5′H suggesting that **conformers 3** and **4** are **disfavoured**.
- **β-conformer 1** accounts for all observed nOe interactions indicating that **C1′H** is **equatorial**.

HPLC conditions

Preparative HPLC purification was performed on an Agilent HP 1100 series chromatograph equipped with a Waters μBondapak C18 column (column length 150 mm, internal diameter of column 3.9 mm, particle size 10 μm, temperature 25 °C). Elution was carried out at a flow rate of 2.0 mL/min using MeOH:H_2O (70:30) and detection was with diode array detection (λ= 250, 272 and 286). The sample was made up to a concentration of 0.015 mg/μL with purification carried out using 25 μL injections which saturated the detector at λ= 250, 272 and 286. t_R = 6.1 min.

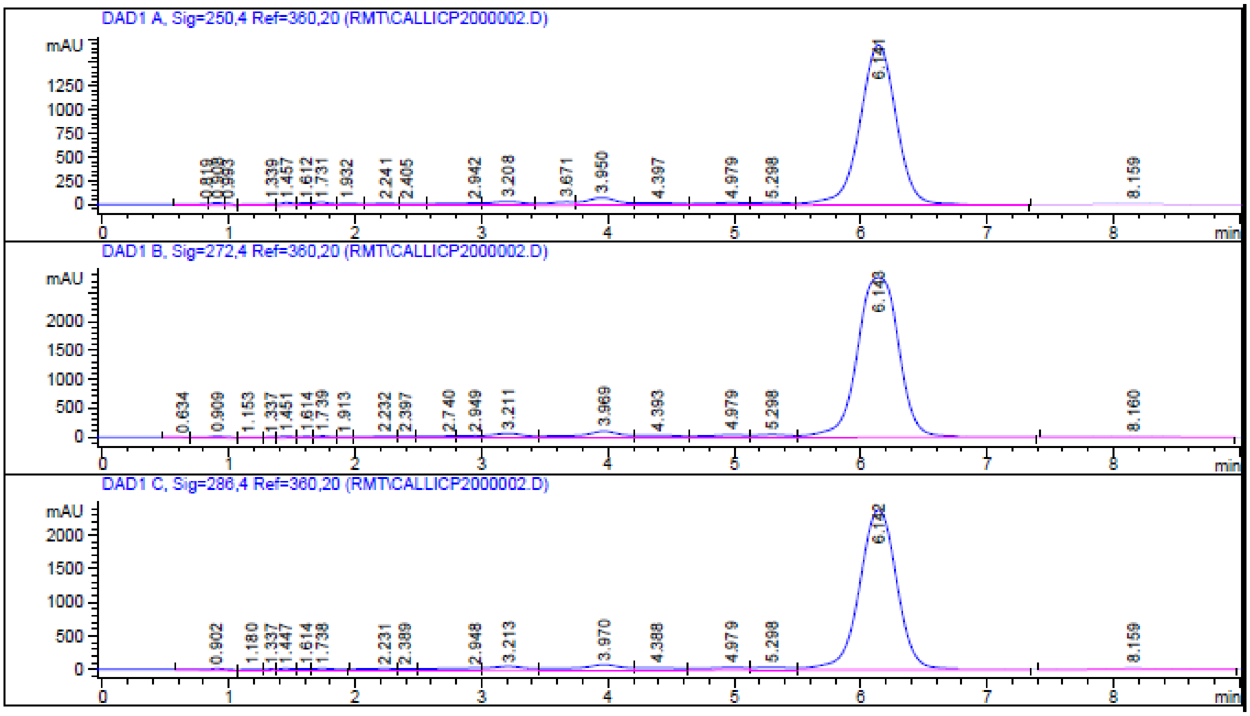

III) Selected NMR Spectra

^{1}H NMR: Dimethyl (7*S*)-5-*O*-(*tert*-butyl(dimethyl)silyl)-7-((2*R*,3*R*,4*E*,7*R*,8*E*,10*E*)-7-((*tert*-butyl(dimethyl)silyl)oxy)-13-((1*S*,2*R*)-2-chlorocyclopropyl)-3-hydroxy-5-methyltrideca-4,8,10-trien-12-yn-2-yl)-2,4,6-trideoxy-6-methyl-α-L-*threo*-hept-3-ulopyranosidonate S20

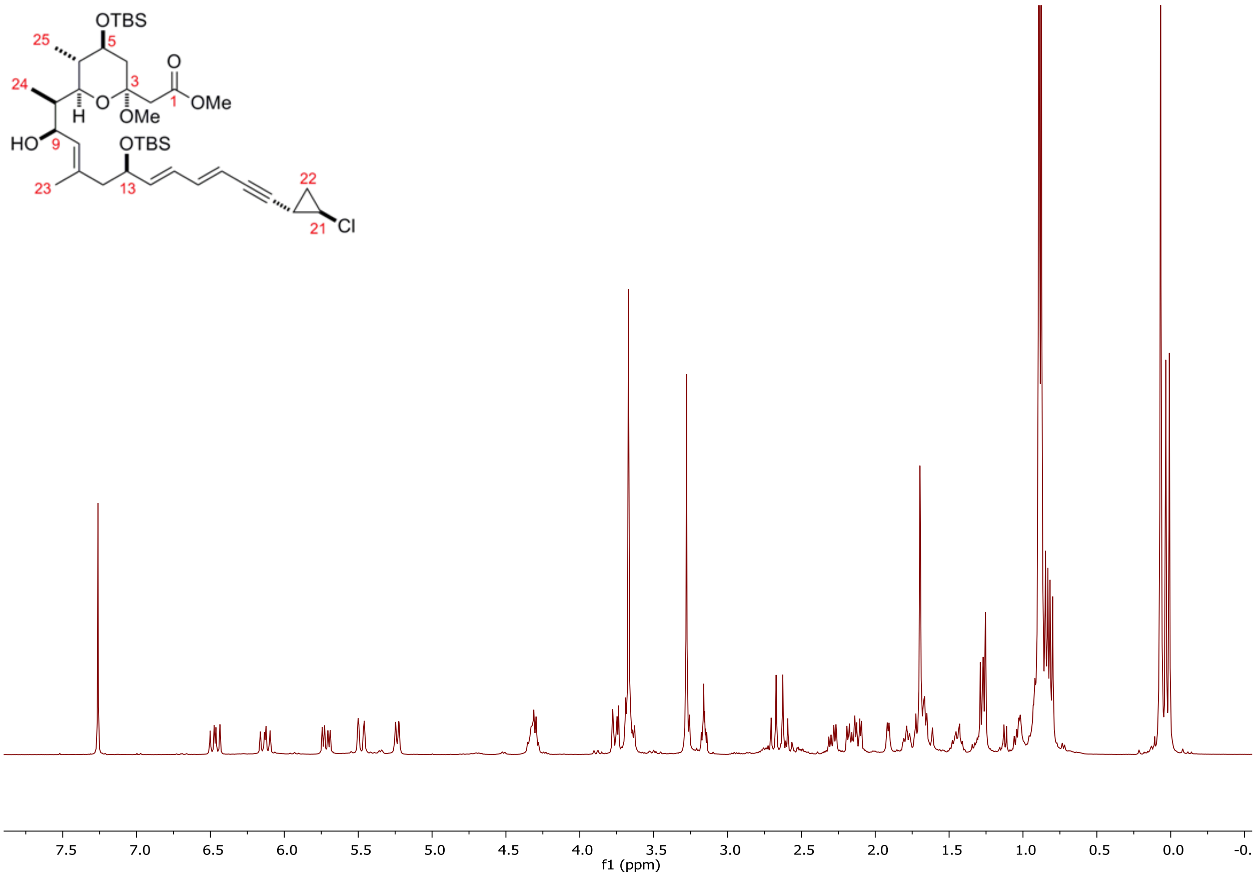

^{13}C NMR: Dimethyl (7*S*)-5-*O*-(*tert*-butyl(dimethyl)silyl)-7-((2*R*,3*R*,4*E*,7*R*,8*E*,10*E*)-7-((*tert*-butyl(dimethyl)silyl)oxy)-13-((1*S*,2*R*)-2-chlorocyclopropyl)-3-hydroxy-5-methyltrideca-4,8,10-trien-12-yn-2-yl)-2,4,6-trideoxy-6-methyl-α-L-*threo*-hept-3-ulopyranosidonate S20

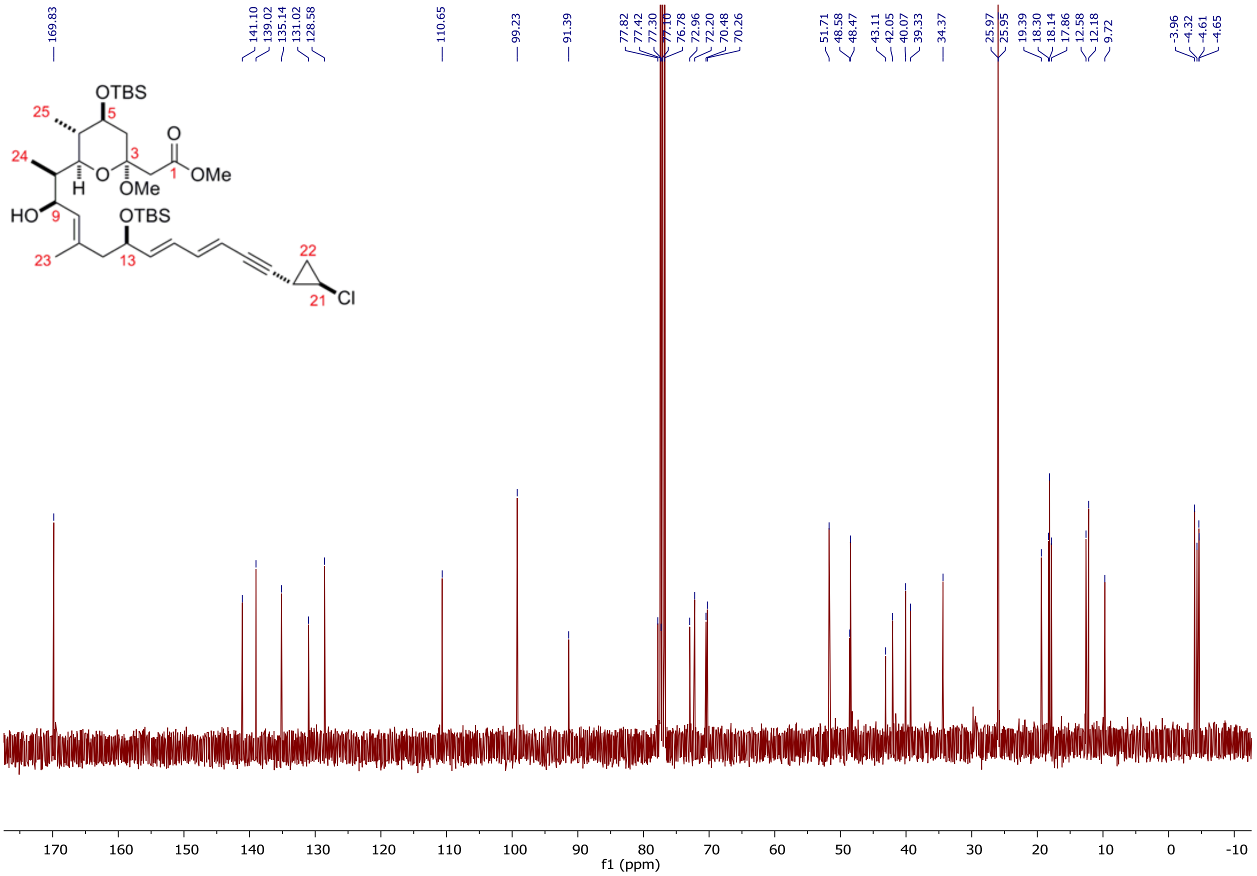

^{1}H NMR: Dimethyl (7*S*)-5-*O*-(*tert*-butyl(dimethyl)silyl)-7-((2*R*,3*R*,4*E*,7*R*,8*E*,10*E*)-7-((*tert*-butyl(dimethyl)silyl)oxy)-13-((1*S*,2*R*)-2-chlorocyclopropyl)-3-methoxy-5-methyltrideca-4,8,10-trien-12-yn-2-yl)-2,4,6-trideoxy-6-methyl-α-L-*threo*-hept-3-ulopyranosidonate 95

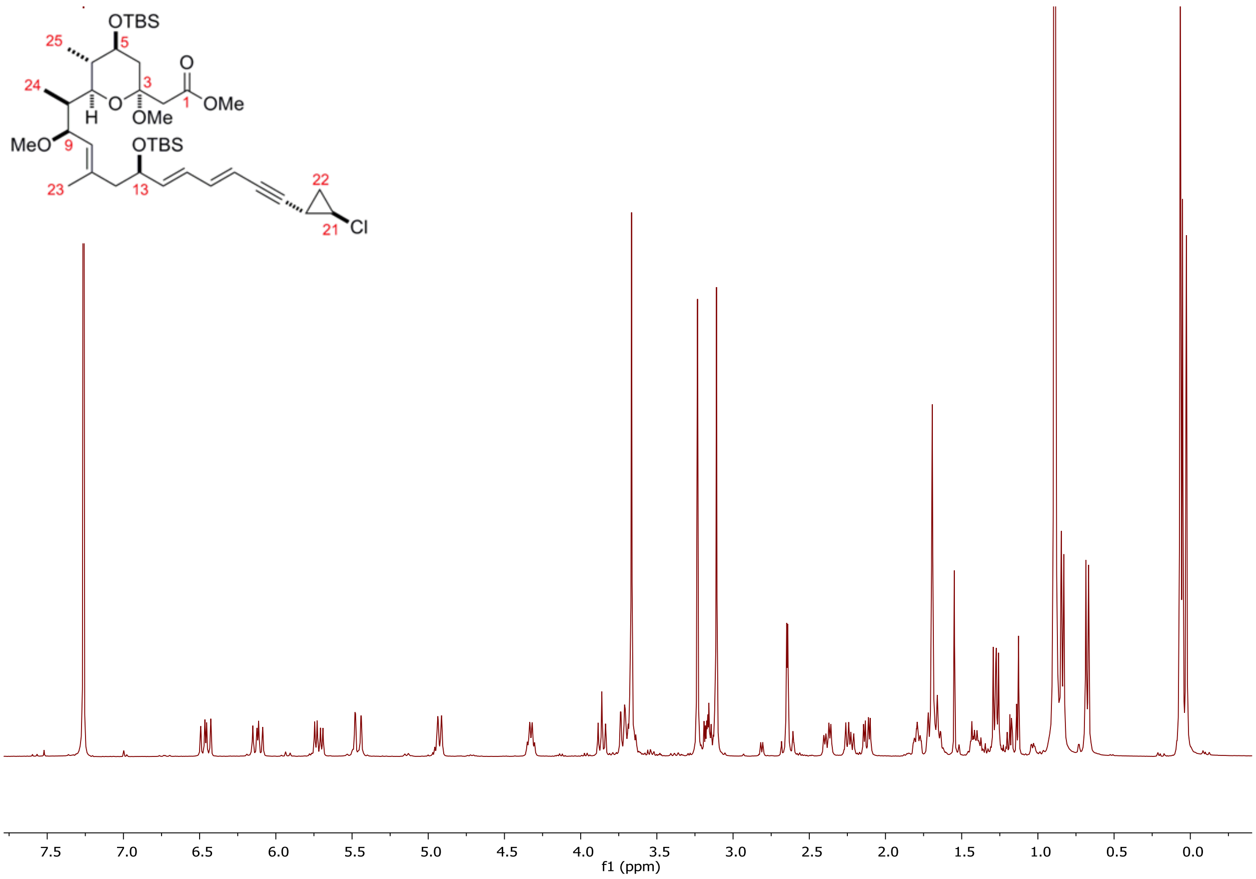

^{13}C NMR: Dimethyl (7*S*)-5-*O*-(*tert*-butyl(dimethyl)silyl)-7-((2*R*,3*R*,4*E*,7*R*,8*E*,10*E*)-7-((*tert*-butyl(dimethyl)silyl)oxy)-13-((1*S*,2*R*)-2-chlorocyclopropyl)-3-methoxy-5-methyltrideca-4,8,10-trien-12-yn-2-yl)-2,4,6-trideoxy-6-methyl-α-L-*threo*-hept-3-ulopyranosidonate 95

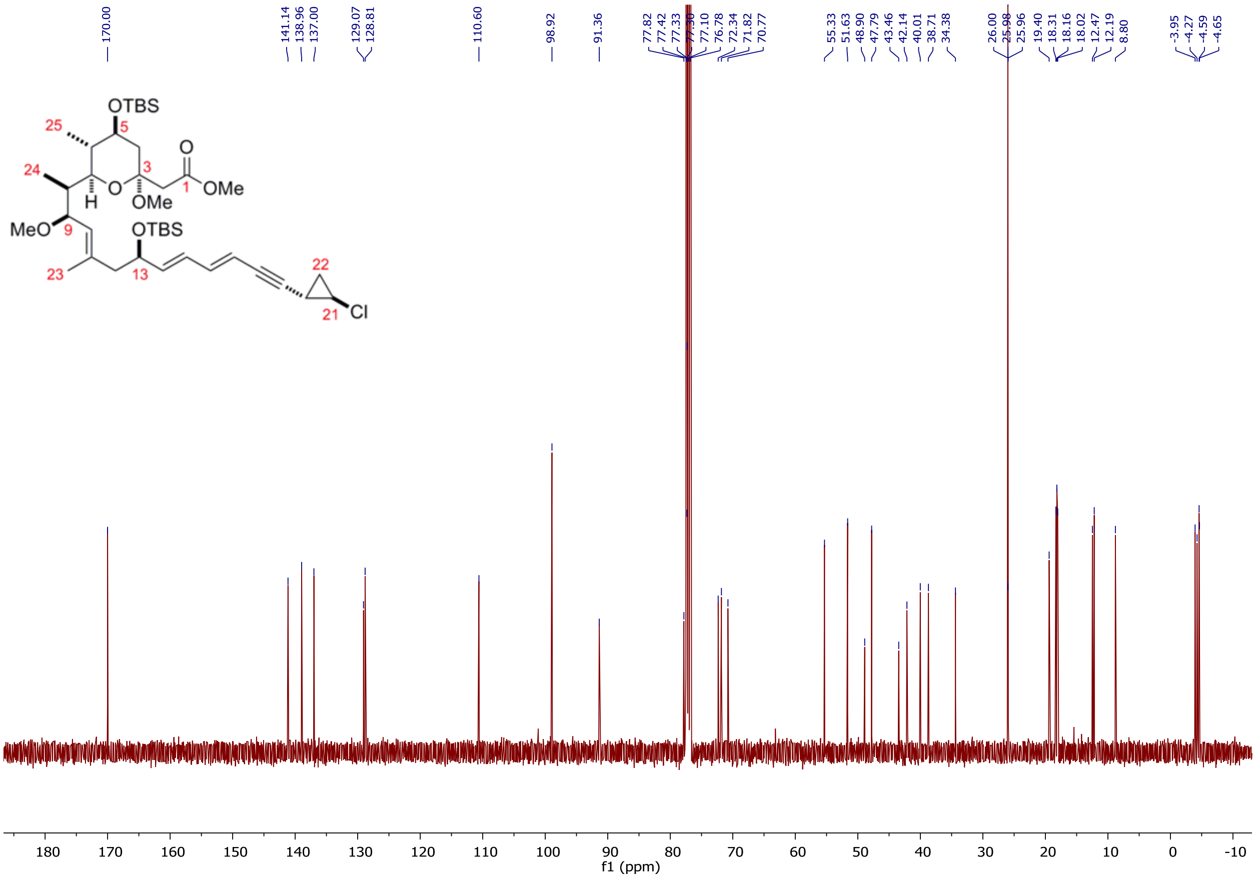

^{1}H NMR: Dimethyl (7*S*)-5-*O*-(*tert*-butyl(dimethyl)silyl)-7-((2*R*,3*R*,4*E*,7*R*,8*E*,10*E*)-13-((1*S*,2*R*)-2-chlorocyclopropyl)-7-hydroxy-3-methoxy-5-methyltrideca-4,8,10-trien-12-yn-2-yl)-2,4,6-trideoxy-6-methyl-α-L-*threo*-hept-3-ulopyranosidonate 99

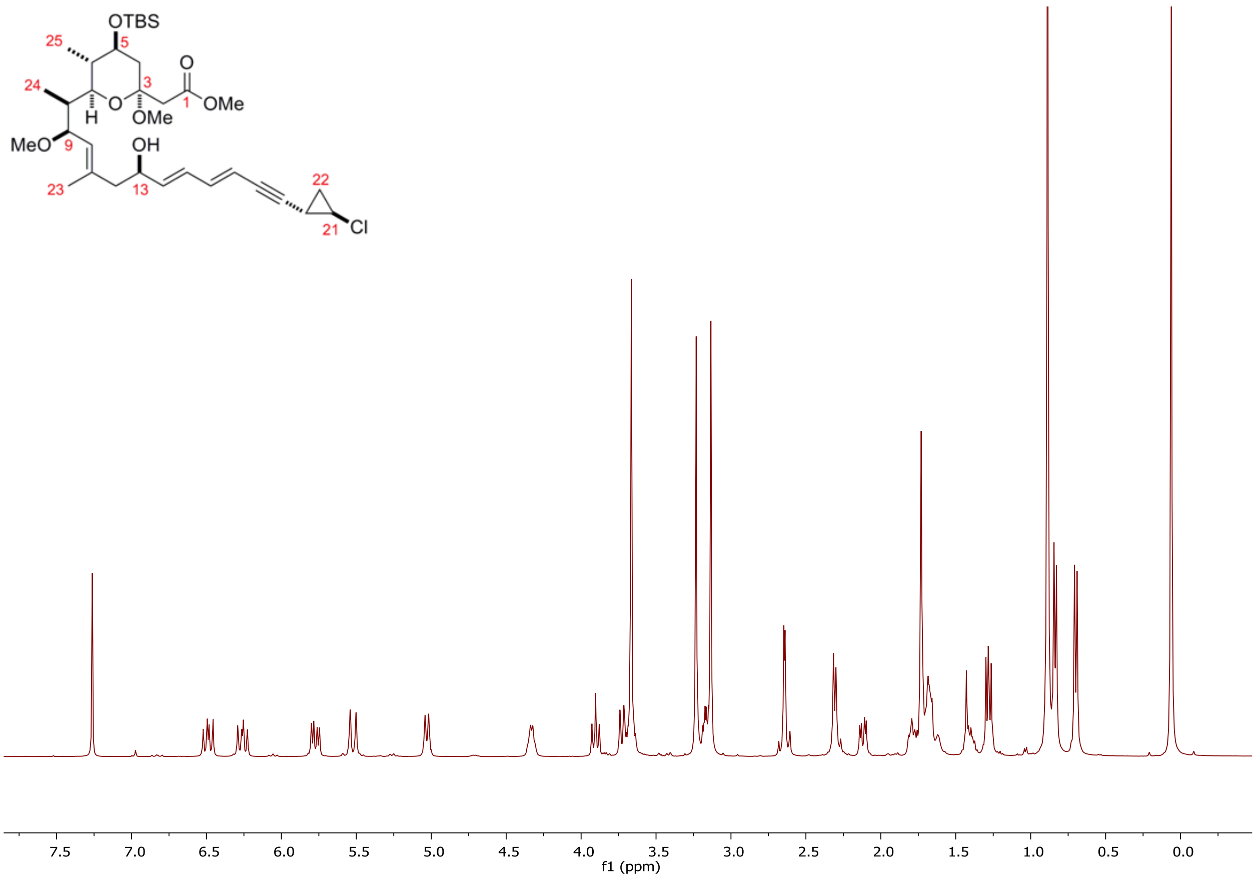

^{13}C NMR: Dimethyl (7*S*)-5-*O*-(*tert*-butyl(dimethyl)silyl)-7-((2*R*,3*R*,4*E*,7*R*,8*E*,10*E*)-13-((1*S*,2*R*)-2-chlorocyclopropyl)-7-hydroxy-3-methoxy-5-methyltrideca-4,8,10-trien-12-yn-2-yl)-2,4,6-trideoxy-6-methyl-α-L-*threo*-hept-3-ulopyranosidonate 99

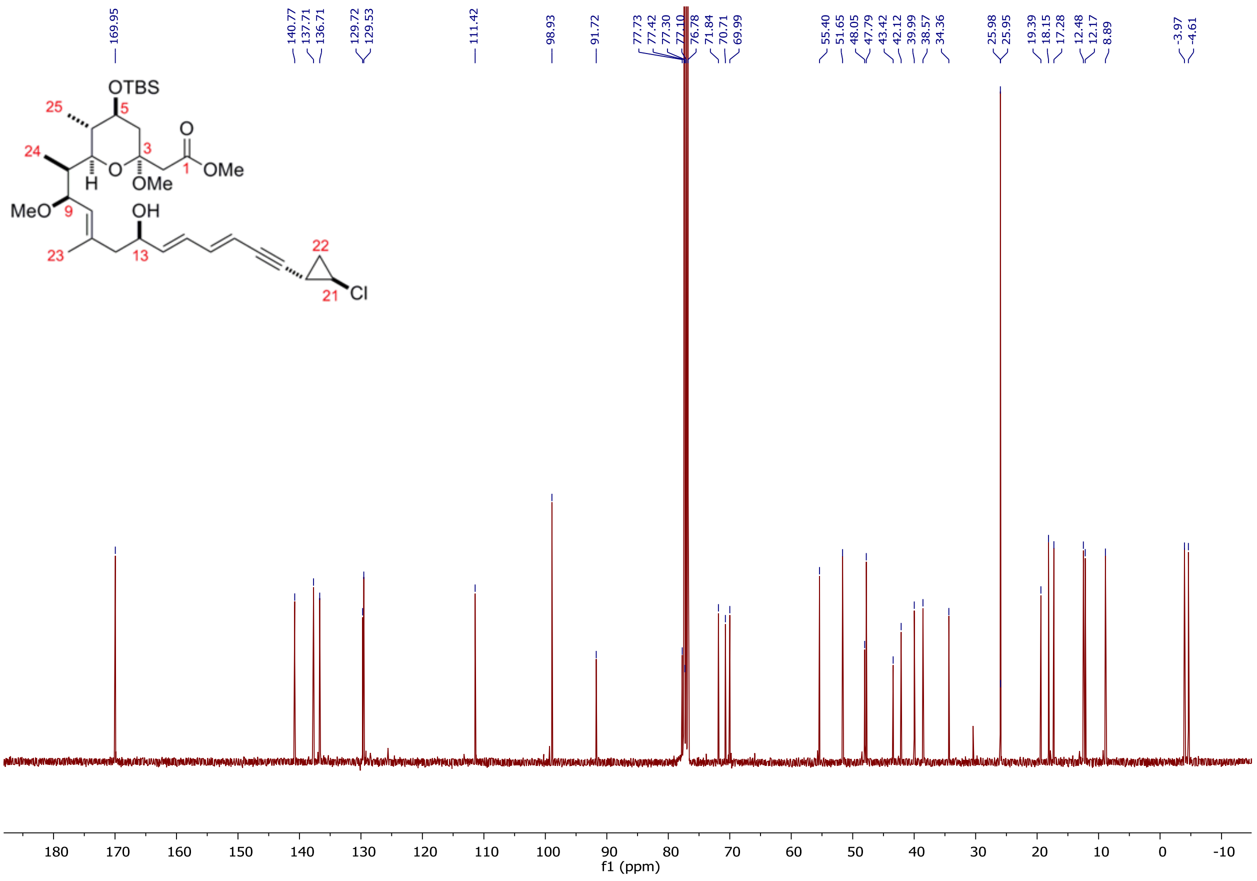

^{1}H NMR: Methyl (7*S*)-5-*O*-(*tert*-butyl(dimethyl)silyl)-7-((2*R*,3*R*,4*E*,7*R*,8*E*,10*E*)-13-((1*S*,2*R*)-2-chlorocyclopropyl)-7-hydroxy-3-methoxy-5-methyltrideca-4,8,10-trien-12-yn-2-yl)-2,4,6-trideoxy-6-methyl-α-L-*threo*-hept-3-ulopyranosidonic acid 100

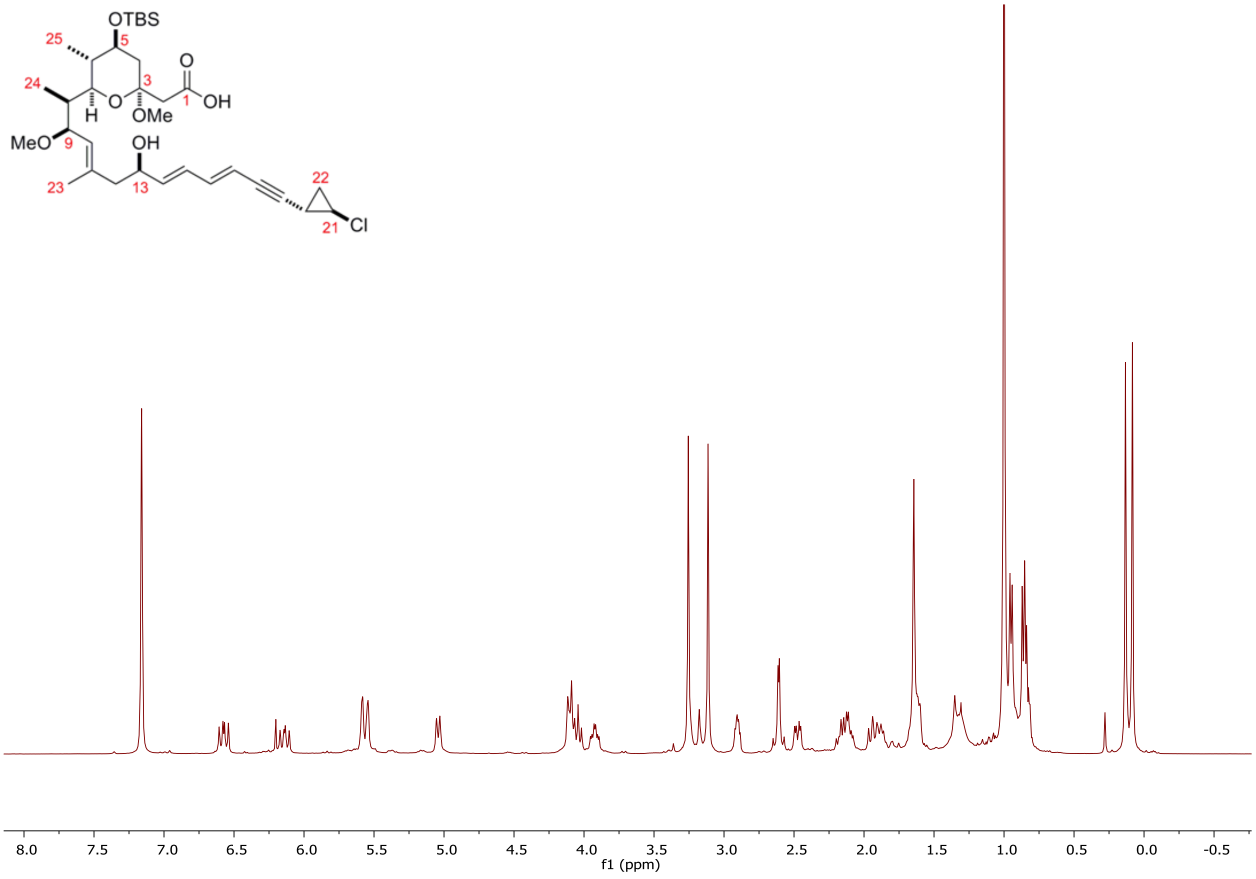

^{13}C NMR: Methyl (7*S*)-5-*O*-(*tert*-butyl(dimethyl)silyl)-7-((2*R*,3*R*,4*E*,7*R*,8*E*,10*E*)-13-((1*S*,2*R*)-2-chlorocyclopropyl)-7-hydroxy-3-methoxy-5-methyltrideca-4,8,10-trien-12-yn-2-yl)-2,4,6-trideoxy-6-methyl-α-L-*threo*-hept-3-ulopyranosidonic acid 100

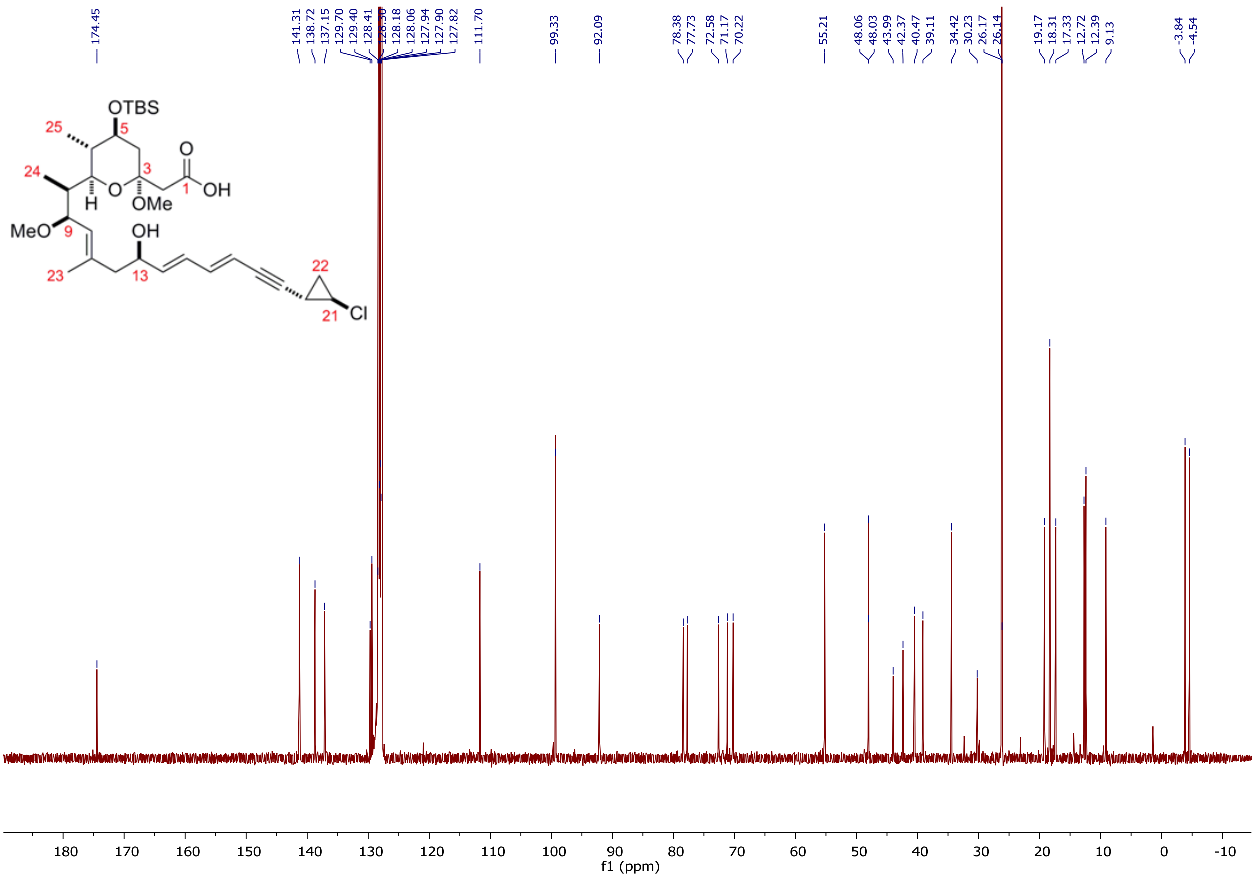

^{1}H NMR: (1*R*,6*R*,8*E*,10*R*,11*R*,12*R*,13*R*,14*S*)-6-((1*E*,3*E*)-6-((1*S*,2*R*)-2-Chlorocyclopropyl)hexa-1,3-dien-5-yn-1-yl)-1,14-dihydroxy-10-methoxy-8,11,13-trimethyl-5,16-dioxabicyclo[10.3.1]hexadec-8-en-4-one 4

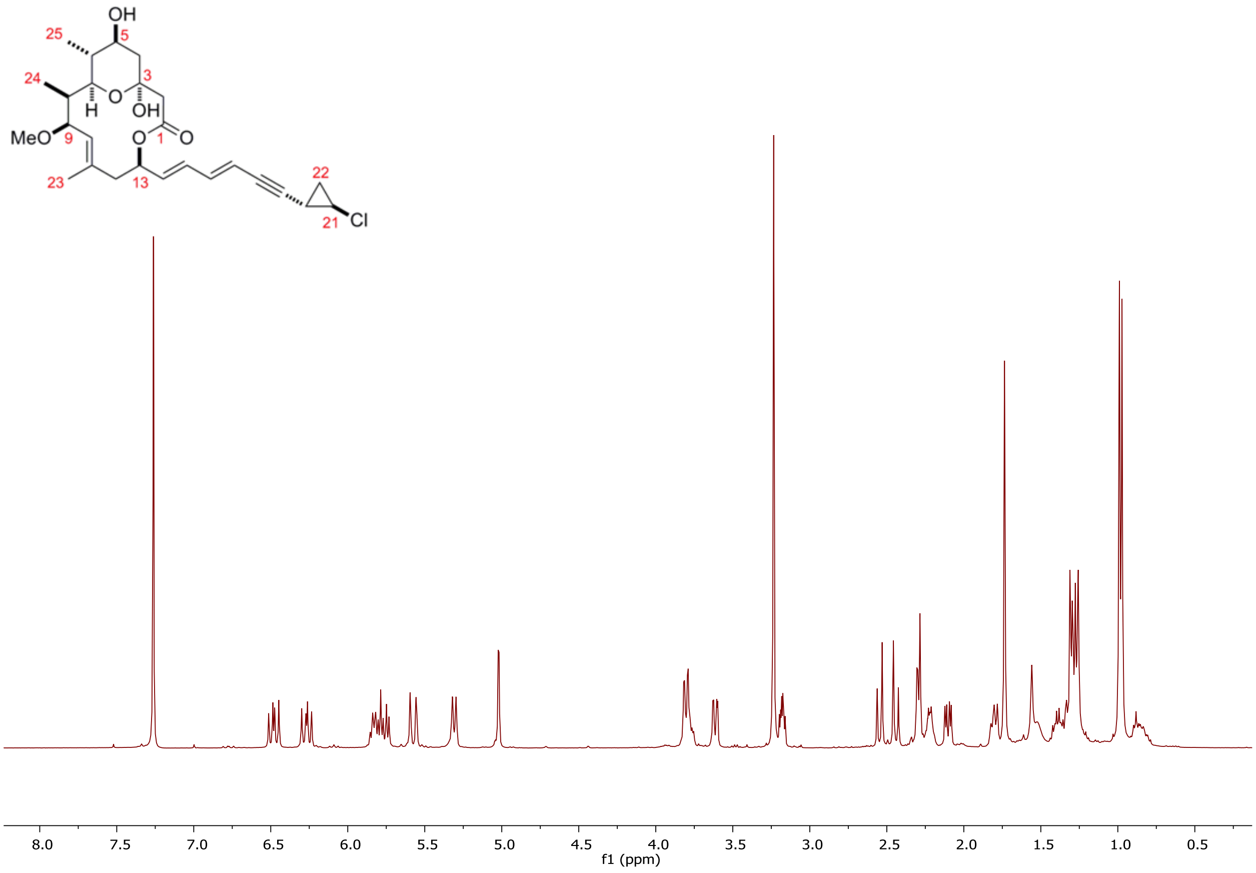

^{13}C NMR: (1*R*,6*R*,8*E*,10*R*,11*R*,12*R*,13*R*,14*S*)-6-((1*E*,3*E*)-6-((1*S*,2*R*)-2-Chlorocyclopropyl)hexa-1,3-dien-5-yn-1-yl)-1,14-dihydroxy-10-methoxy-8,11,13-trimethyl-5,16-dioxabicyclo[10.3.1]hexadec-8-en-4-one 4

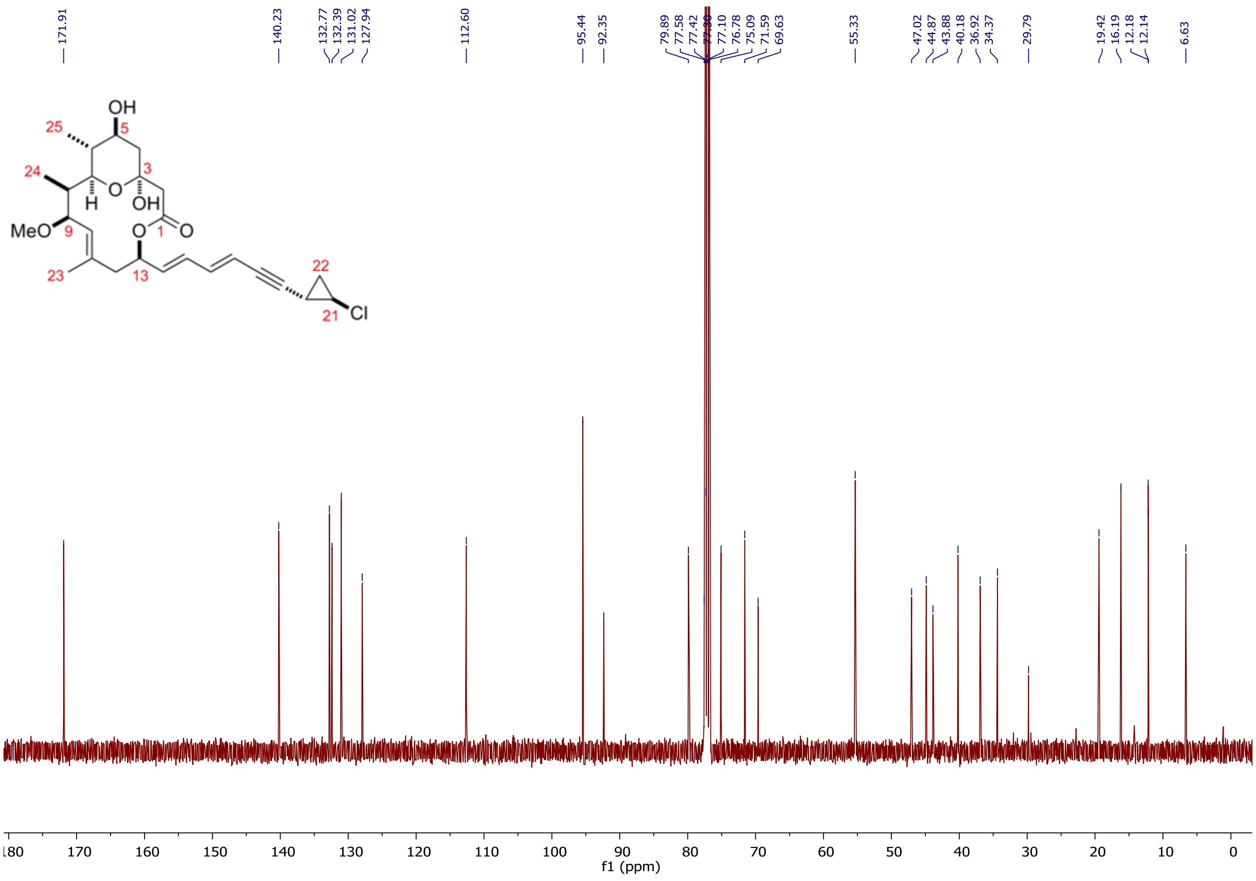

^{1}H NMR: Callipeltoside A (1)

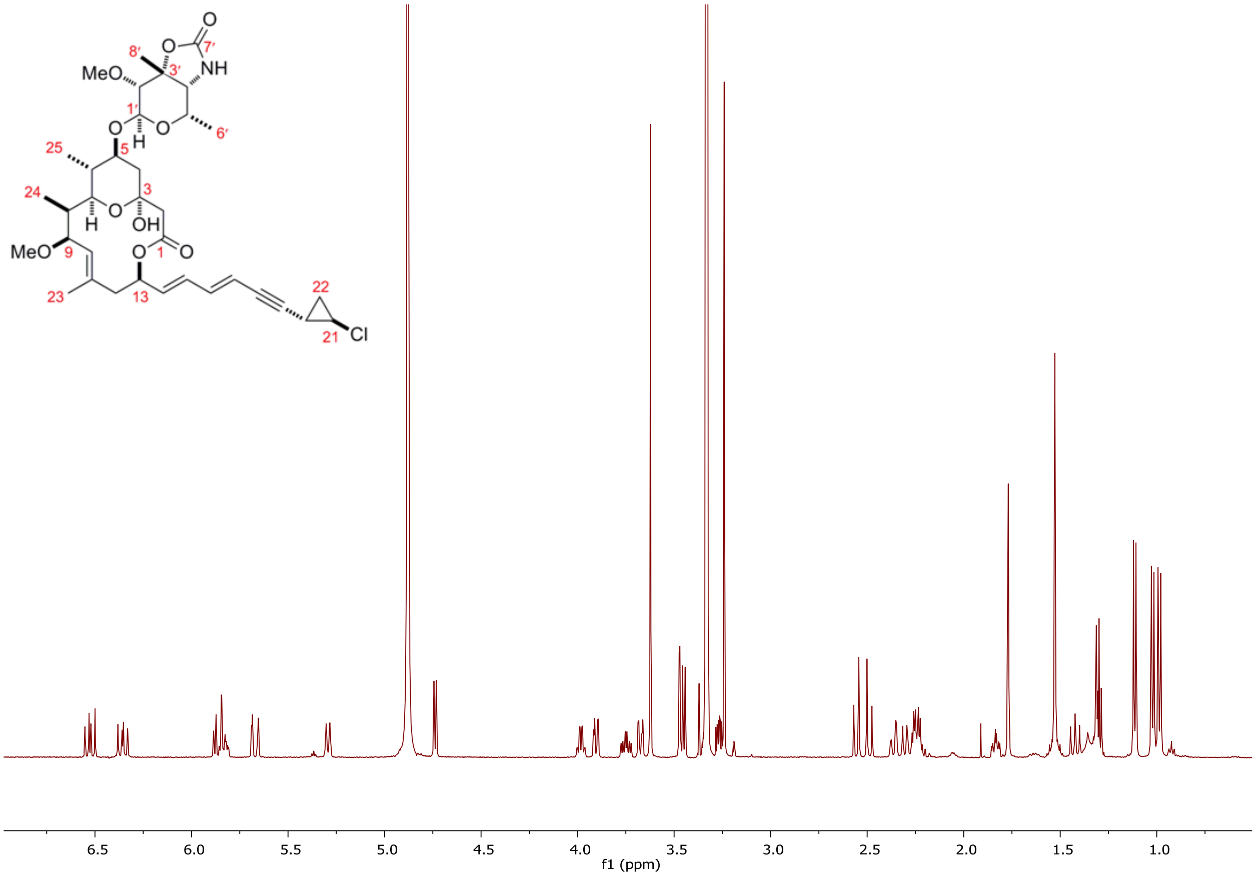

^{13}C NMR: Callipeltoside A (1)

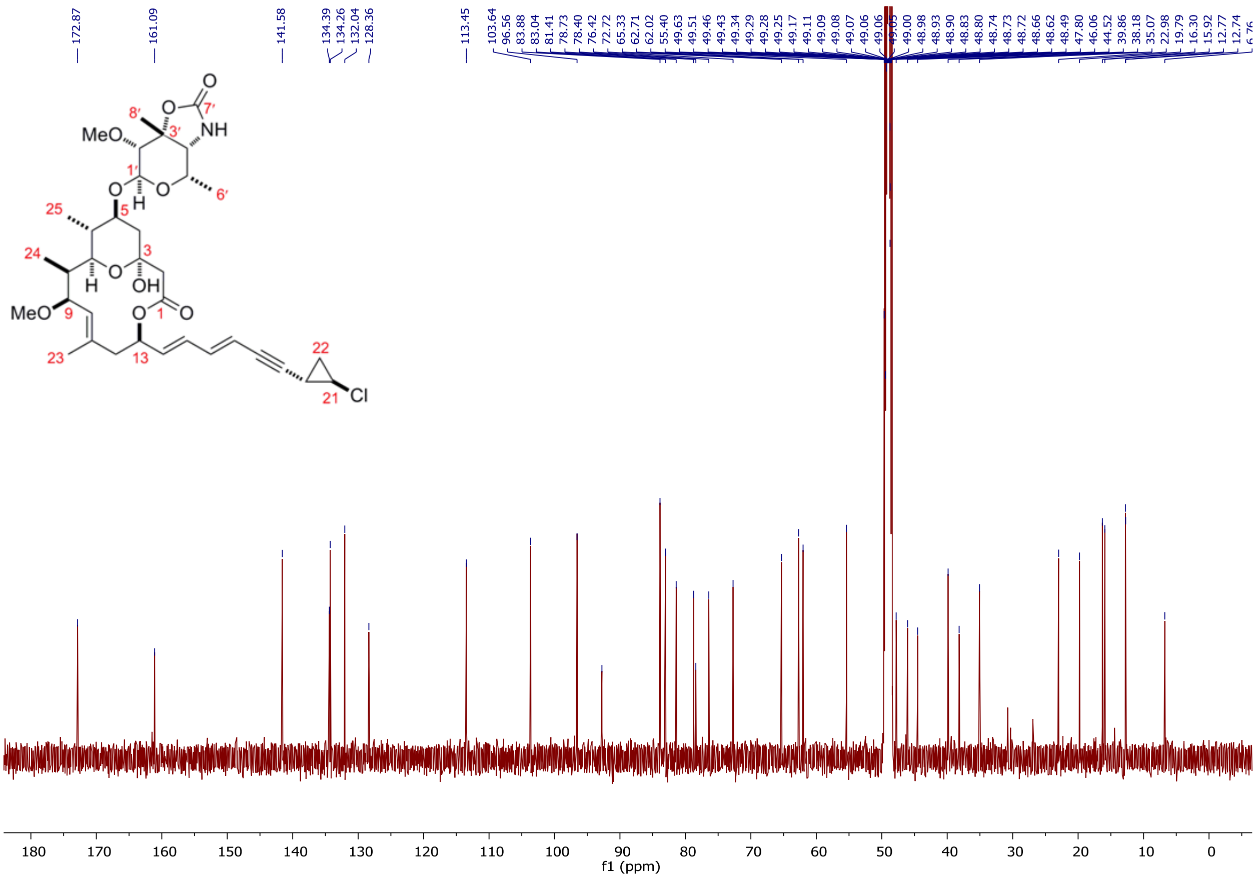

^{1}H NMR: Callipeltoside B (2), $CDCl_3$

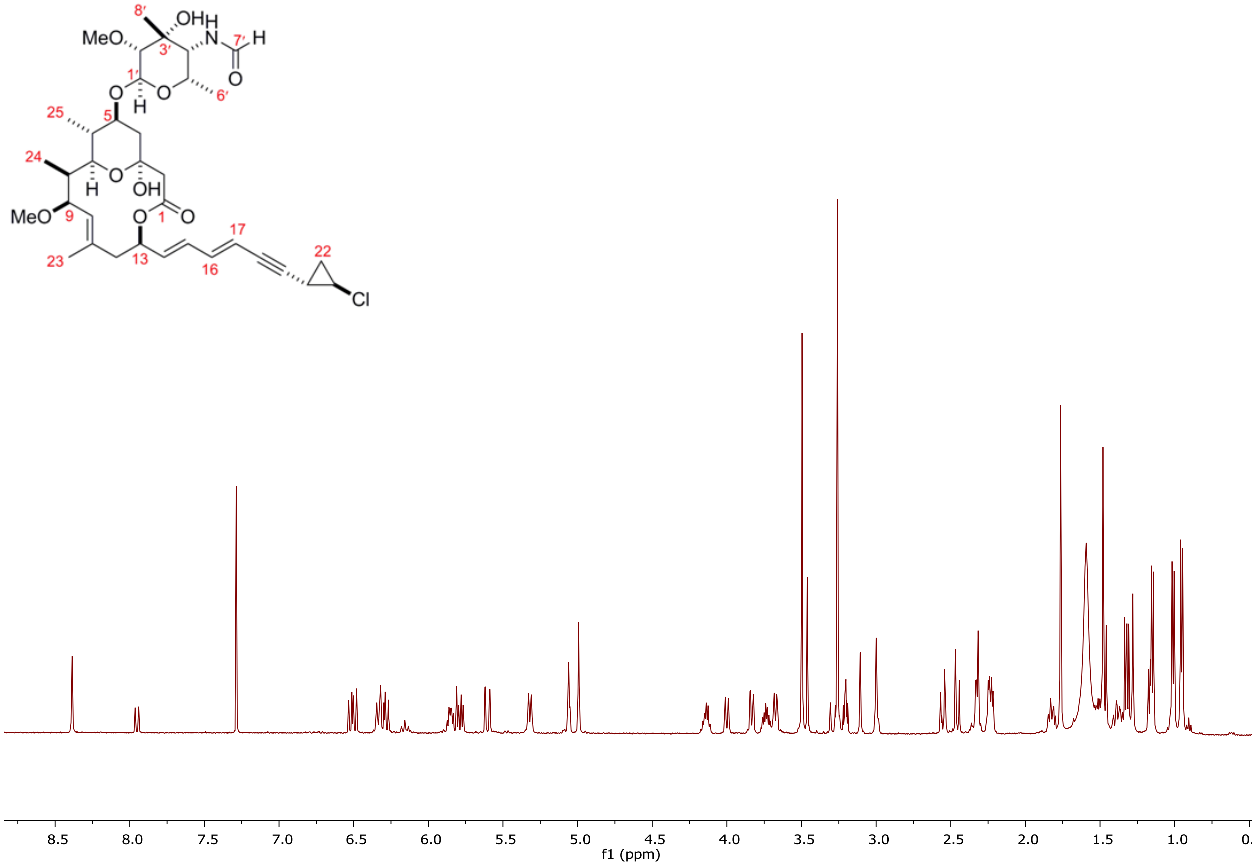

^{13}C NMR: Callipeltoside B (2), $CDCl_3$

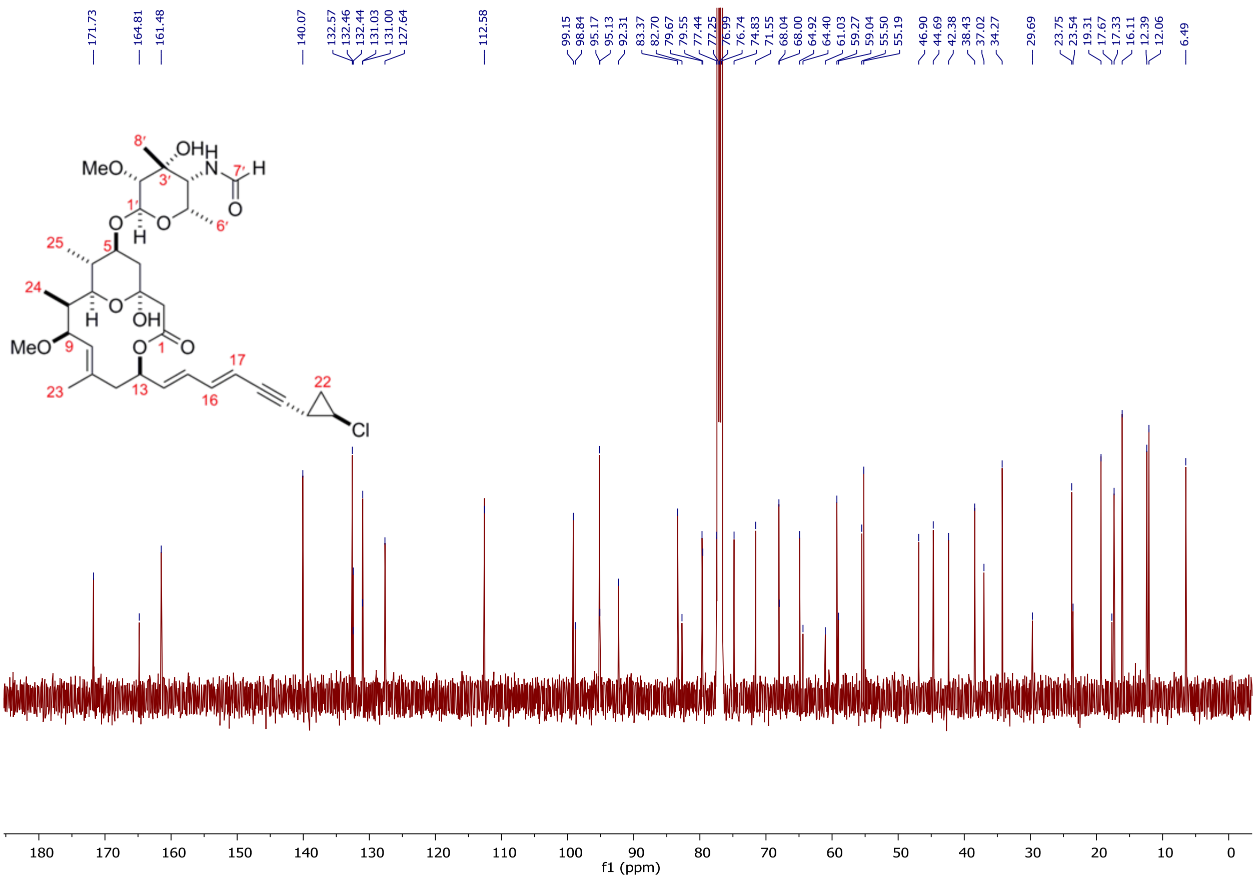

^{1}H NMR: Callipeltoside B (2), MeOD

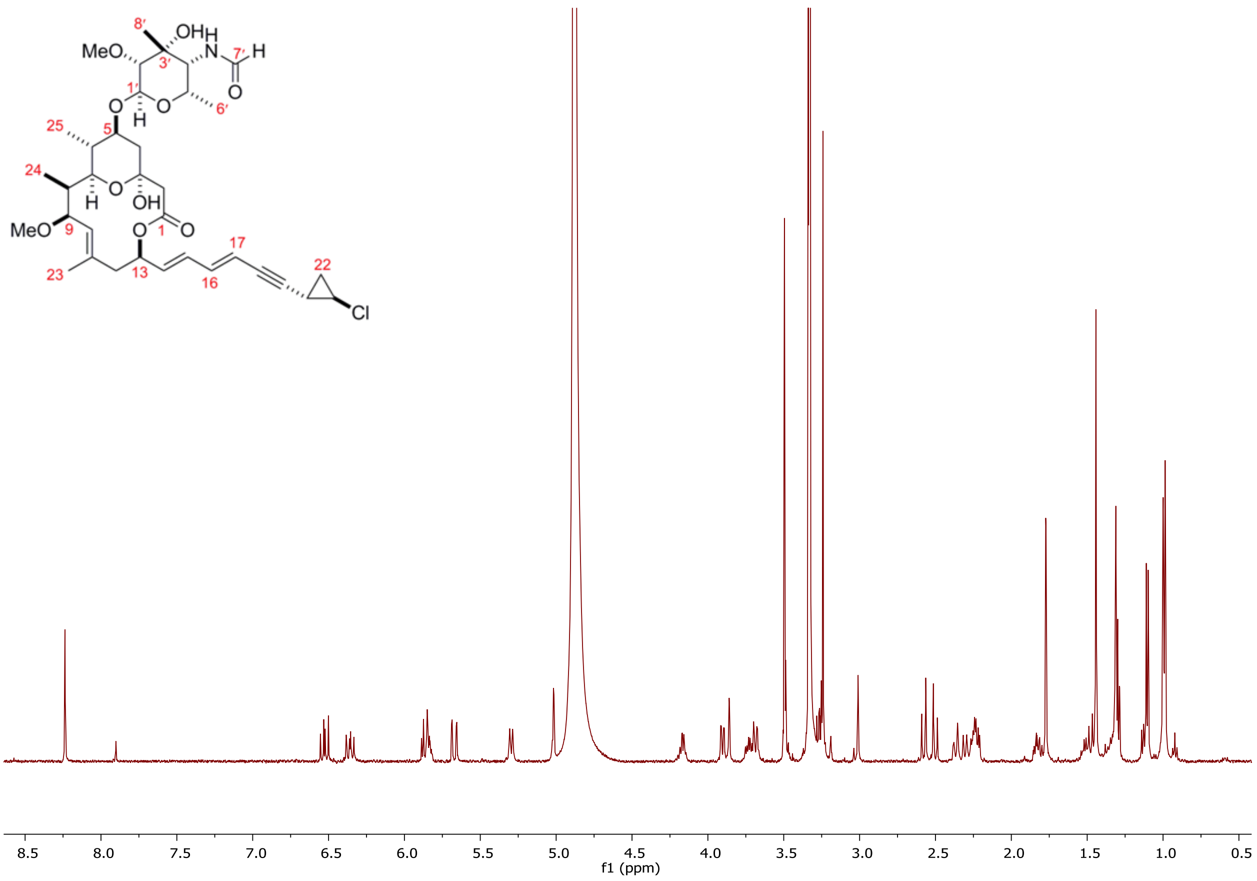

^{13}C NMR: Callipeltoside B (2), MeOD

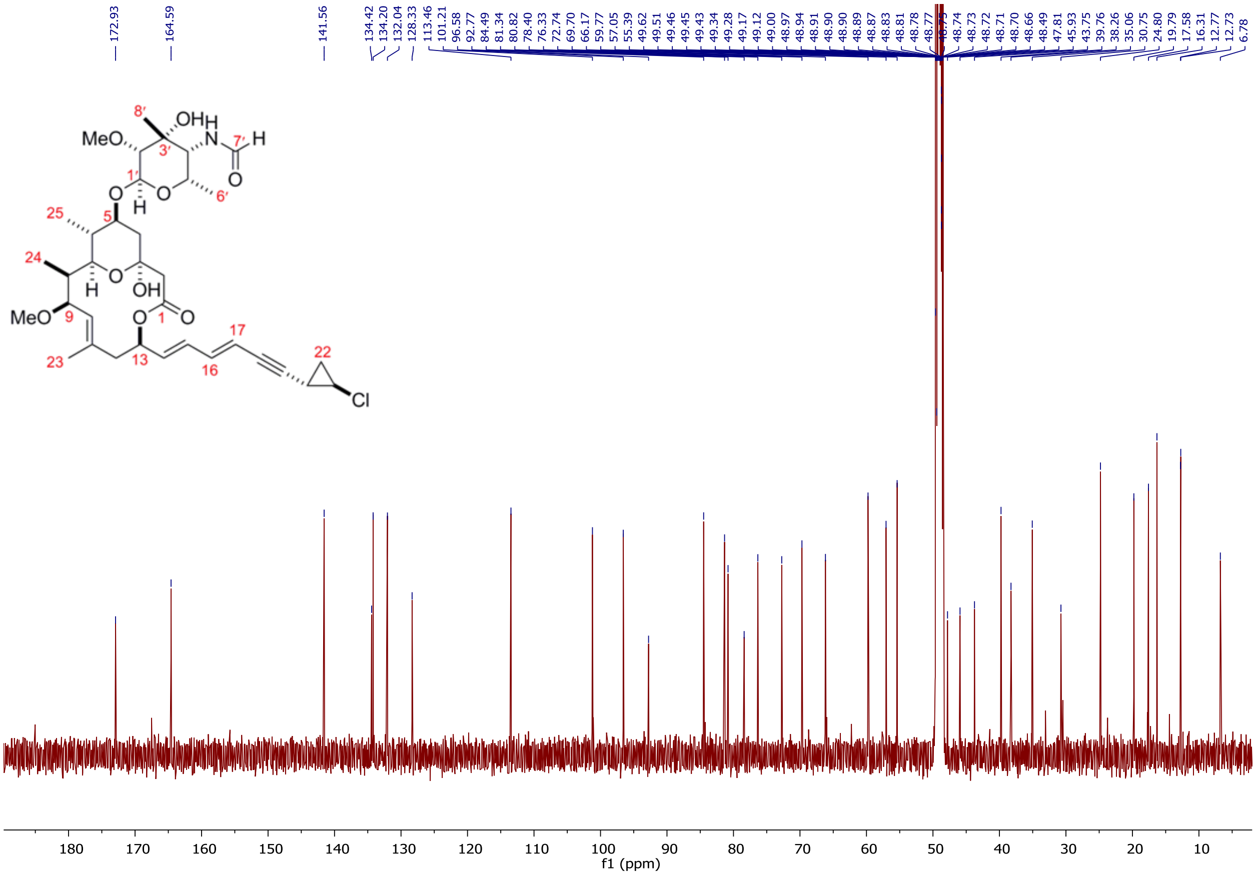

^{1}H NMR: Callipeltoside B diastereomer 128

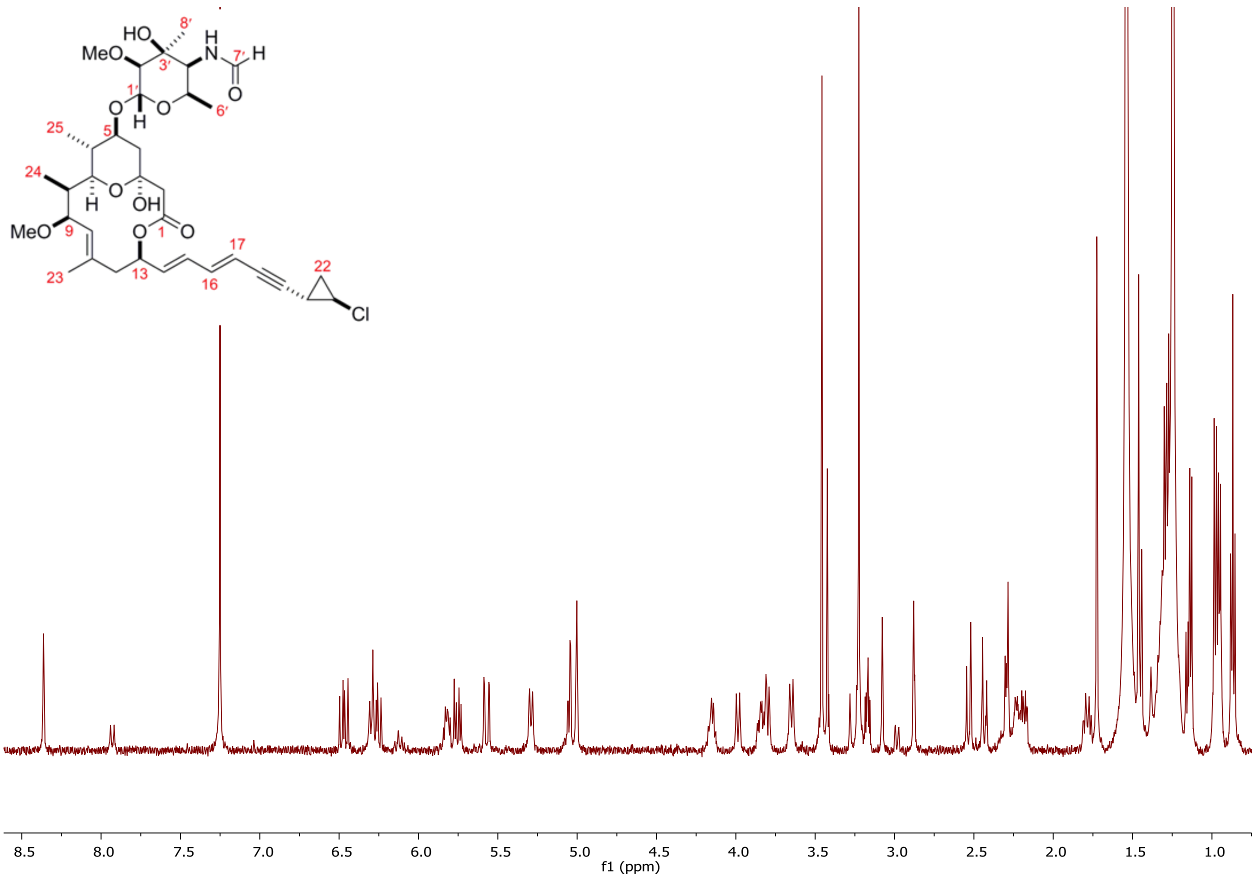

^{13}C NMR: Callipeltoside B diastereomer 128

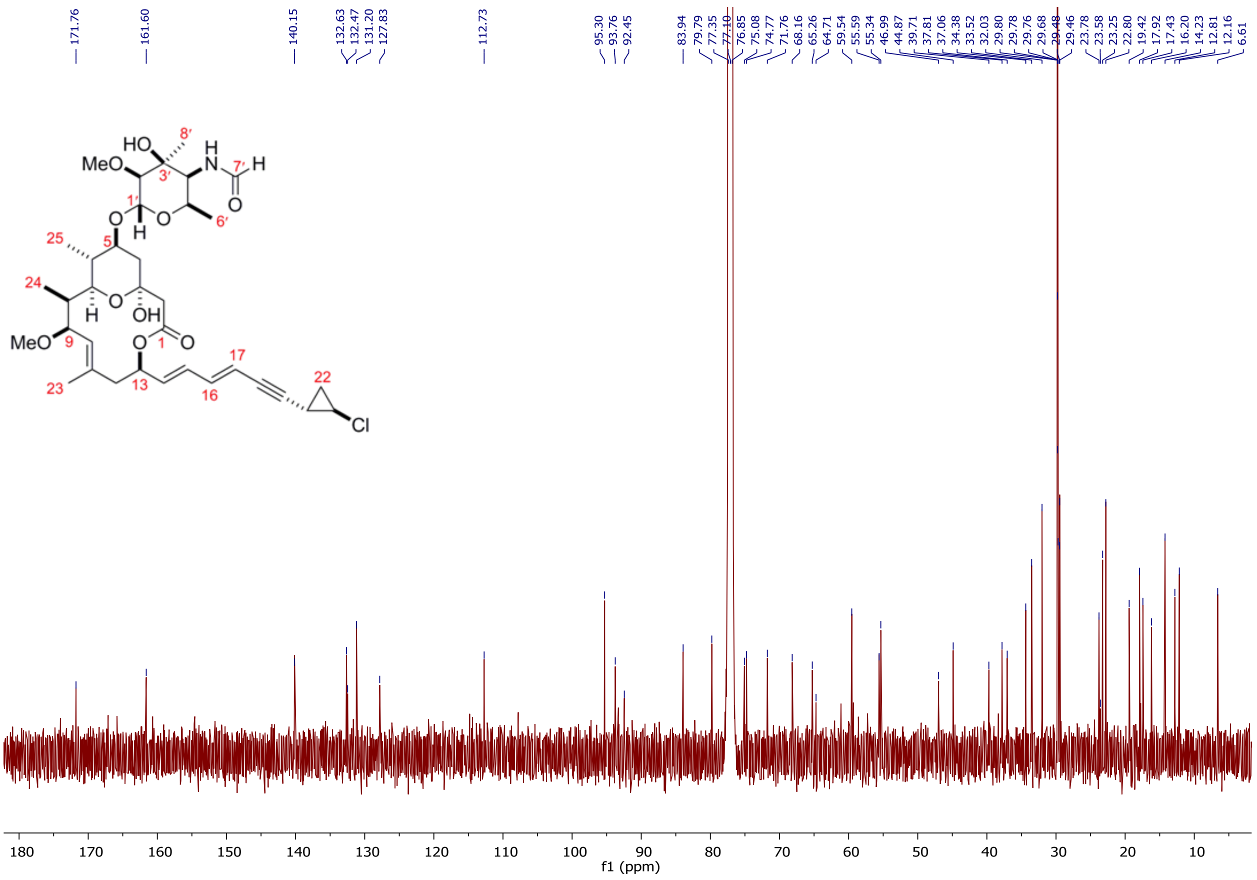

^{1}H NMR: Callipeltoside C (3)

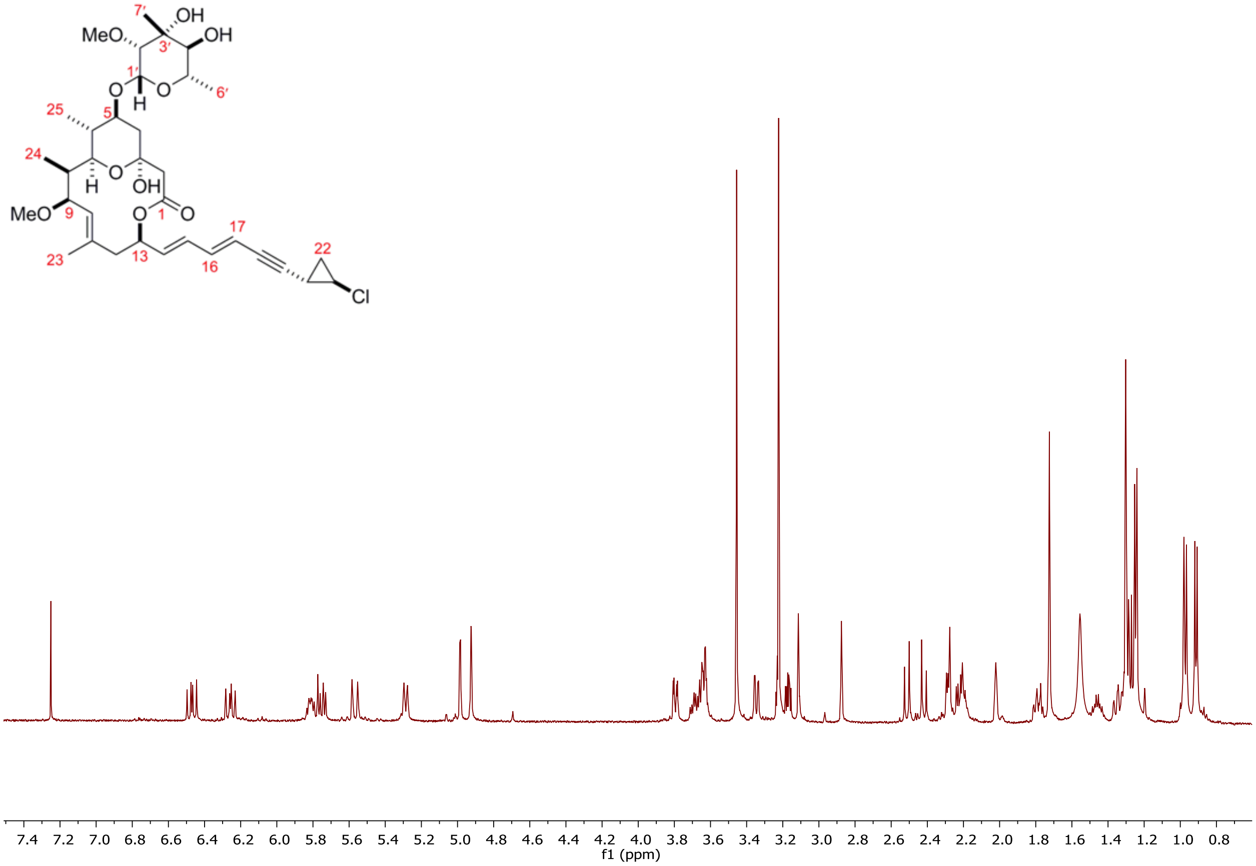

^{13}C NMR: Callipeltoside C (3)

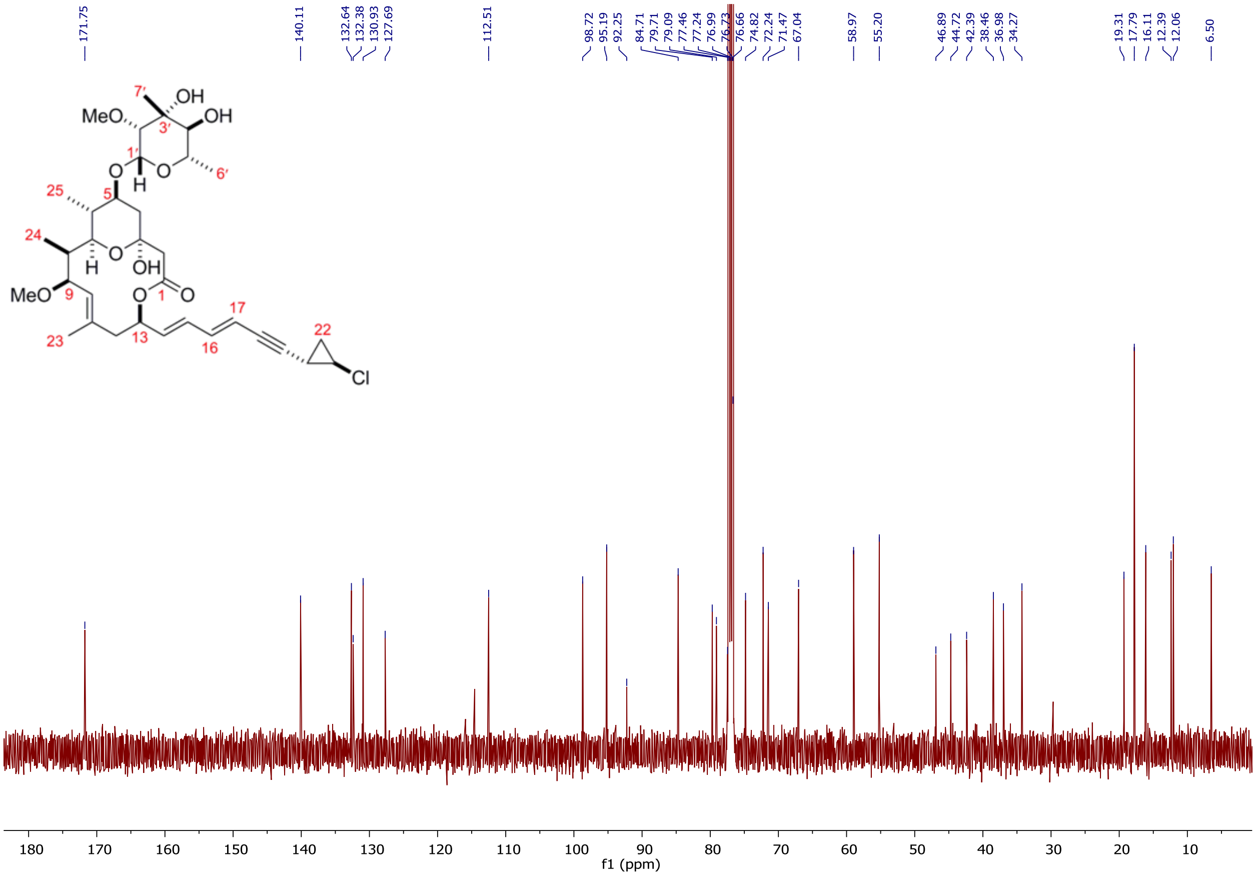

IV) References

1. a) J. A. Marshall, H. R. Chobanian, *J. Org. Chem.* **2000**, *65*, 8357–8360; b) J. A. Marshall, G. M. Schaaf, *J. Org. Chem.* **2001**, *66*, 7825–7831.
2. a) S. D. Burke, J. E. Cobb, K. Takeuchi, *J. Org. Chem.* **1990**, *55*, 2138–2151; b) W. R. Roush, A. D. Palkowitz, K. Ando, *J. Am. Chem. Soc.* **1990**, *112*, 6348–6359.
3. A. Zampella, M. Sorgente, M. V. D'Auria, *Tetrahedron: Asymmetry* **2002**, *13*, 681–685.
4. D. A. Evans, R. L. Dow, T. L. Shih, J. M. Takacs, R. Zahler, *J. Am. Chem. Soc.* **1990**, *112*, 5290–5313.
5. a) W. R. Roush, A. D. Palkowitz, M. J. Palmer, *J. Org. Chem.* **1987**, *52*, 316–318; b) W. R. Roush, K. Ando, D. B. Powers, A. D. Palkowitz, R. L. Halterman, *J. Am. Chem. Soc.* **1990**, *112*, 6339–6348; c) W. R. Roush, A. D. Palkowitz, K. Ando, *J. Am. Chem. Soc.* **1990**, *112*, 6348–6359.
6. .a) J. A. Dale, H. S. Mosher, *J. Am. Chem. Soc.* **1973**, *95*, 512–519; b) T. R. Hoye, C. S. Jeffrey, F. Shao, *Nature Protoc.* **2007**, *2*, 2451–2458.
7. J. Carpenter, A. B. Northrup, D. Chung, J. J. M. Wiener, S. Kim, D. W. C. MacMillan, *Angew. Chem.* **2008**, *120*, 3624–3628; *Angew. Chem. Int. Ed.* **2008**, *47*, 3568–3572.
8. a) M. Frigerio, M. Santagostino, S. Sputore, *J. Org. Chem.* **1999**, *64*, 4537–4538; b) R. E. Ireland, L. Liu, *J. Org. Chem.* **1993**, *58*, 2899–2899.
9. T. Ichige, Y. Okano, N. Kanoh, M. Nakata, *J. Org. Chem.* **2009**, *74*, 230–243.
10. C. D. Papageorgiou, M. A. Cubillo de Dios, S. V. Ley, M. J. Gaunt, *Angew. Chem.* **2004**, *116*, 4741–4744; *Angew. Chem. Int. Ed.* **2004**, *43*, 4641–4644.
11. a) A. B. Charette, H. Juteau, *J. Am. Chem. Soc.* **1994**, *116*, 2651–2652; b) A. B. Charette, S. Prescott, C. Brochu, *J. Org. Chem.* **1995**, *60*, 1081–1083; c) For DFT computational studies: T. Wang, Y. Liang, Z.-X. Yu, *J. Am. Chem. Soc.* **2011**, *133*, 9343–9353.
12. H. Huang, J. S. Panek, *Org. Lett.* **2004**, *6*, 4383–4385.
13. a) S. R. Landor, E. S. Pepper, *J. Chem. Soc. C.* **1966**, 2283–2285; b) R. B. Bates, W. A. Beavers, B. Gordon III, N. S. Mills, *J. Org. Chem.* **1979**, *44*, 3800–3803.
14. J. D. White, S. Jana, *J. Org. Chem.* **2014**, *79*, 700–710.
15. P. Li, J. Li, F. Arikan, W. Ahlbrecht, M. Dieckmann, D. Menche, *J. Am. Chem. Soc.* **2009**, *131*, 11678–11679.

16. a) K. C. Nicolaou, T. K. Chakraborty, A. D. Piscopio, N. Minowa, P. Bertinato, *J. Am. Chem. Soc*. **1993**, *115*, 4419–4420; b) K. C. Nicolaou, A. D. Piscopio, P. Bertinato, T. K. Chakraborty, N. Minowa, K. Koide, *Chem. Eur. J*. **1995**, *1*, 318–333.
17. a) H. X. Zhang, F. Guibé, G. Balavoine, *J. Org. Chem*. **1990**, *55*, 1857–1867; b) H. T. Dieck, H. Friedel, *J. Organomet. Chem*. **1968**, *14*, 375–385.
18. J. D. White, C. M. Lincoln, J. Yang, W. H. C. Martin, D. B. Chan, *J. Org. Chem*. **2008**, *73*, 4139–4150.
19. P. Prinz, A. Lansky, B. Knieriem, A. de Meijere, T. Haumann, R. Boese, M. Noltemeyer, *Angew. Chem*. **1997**, *109*, 1343–1346; *Angew. Chem. Int. Ed*. **1997**, *36*, 1289–1292.
20. A. F. Renaldo, J. W. Labadie, J. K. Stille, *Org. Synth. Coll*. *8*, 268.
21. C. M. Hettrick, W. J. Scott, *J. Am. Chem. Soc*. **1991**, *113*, 4903–4910.
22. I. Paterson, R. D. M. Davies, A. C. Heimann, R. Marquez, A. Meyer, *Org. Lett.* **2003**, *5*, 4477–4480.
23. A. J. Pihko, K. C. Nicolaou, A. M. P. Koskinen, *Tetrahedron: Asymmetry* **2001**, *12,* 937–942.
24. L. Kisfaludy, L. Ötvös, *Synthesis* **1987**, 510.
25. M. Langner, S. Laschat, J. Grunenberg, *Eur. J. Org. Chem.* **2003**, 1494–1499.
26. D. A. Evans, J. D. Burch, E. Hu, G. Jaeschke, *Tetrahedron* **2008**, *64*, 4671–4699.
27. A. Zampella, M. V. D'Auria, L. Minale, C. Debitus, C. Roussakis, *J. Am. Chem. Soc*. **1996**, *118*, 11085–11088.
28. I. Paterson, R. D. M. Davies, R. Marquez, *Angew Chem*. **2001**, *113*, 623–627; *Angew. Chem. Int. Ed*. **2001**, *40*, 603–607.
29. a) N. Muller, D. E. Pritchard, *J. Chem. Phys*. **1959**, *31*, 768–771; b) G. E. Maciel, J. W. McIver, Jr, N. S. Ostlund, J. A. Pople, *J. Am. Chem. Soc*. **1970**, *92*, 1–11; c) K. Bock, I. Lundt, C. Pedersen, *Tetrahedron Lett*. **1973**, *14*, 1037–1040; d) K. Bock, L. Wiebe, *Acta Chem. Scand*. **1973**, *27*, 2676–2678; e) K. Bock, C. Pedersen, *J. Chem. Soc., Perkin Trans*. **1974**, 293–299.
30. A. Zampella, M. V. D'Auria, L. Minale, *Tetrahedron*, **1997**, *53*, 3243–3248.
